# Asymmetric Synthesis of Functionalizable Type II β-Turn-Inducing α-Amino Acid Building Blocks

**DOI:** 10.1021/acs.orglett.3c02376

**Published:** 2023-08-29

**Authors:** Wenzheng Gao, Jiaxin Han, Sophie Greaves, Joseph P. A. Harrity

**Affiliations:** Department of Chemistry, University of Sheffield, Sheffield S3 7HF, United Kingdom

## Abstract

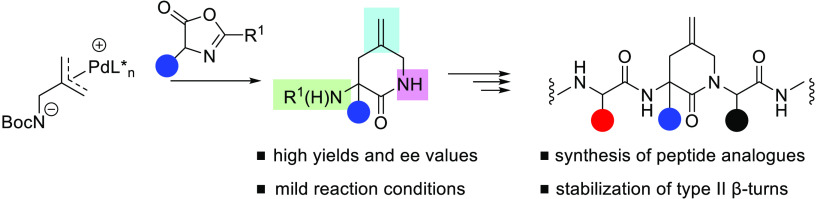

Peptidomimetics are emerging as a promising class of potent and selective therapeutics. Among the current approaches to these compounds, the utilization of constrained lactams is a key element in enforcing the active peptide conformation, and the development of efficient and stereocontrolled methods for generating such lactam building blocks is an important objective. Current methods typically rely on the elaboration of existing α-amino acids, and in so doing, the side chain is sacrificed during the ring-forming process. We report a new asymmetric approach to lactam-constrained α-amino acid building blocks bearing a range of polar and hydrophobic side chains. The chemistry is amenable to rapidly generating di- and tripeptides, and the potential for these lactams to stabilize type II β-turns is demonstrated in the synthesis of the melanocyte-inhibiting factor peptidomimetic.

Despite mediating a plethora of vital biological processes in living organisms, peptides remain a challenging class of drug targets due to their poor cell penetration, proteolytic instability, and unfavorable pharmacokinetics. Among the approaches employed to address these shortcomings,^1^ the incorporation of conformational constraints has the potential to enhance stability and, if the constraint can mimic a bioactive conformation, improve potency.^2^ With these goals in mind, lactam-bridged peptides were introduced by Freidinger, and these have been proven to deliver an effective class of peptidomimetics as they stabilize type II β-turns.^3^ For example, and as shown in Figure 1a, an analogue of the luteinizing hormone-releasing hormone containing a γ-lactam as a conformational constraint showed improved agonist activity as compared to the parent hormone due to the stabilization of a bioactive conformation containing a β-turn. There are several strategies for the synthesis of Freidinger lactams, including cyclocondensation^3,4^ and ring-closing metathesis,^5^ but in general, they provide motifs that lack functionality, which limits their further elaboration downstream (Figure 1b). In addition, current approaches rely almost exclusively on the stereospecific elaboration of available α-amino acids, and de novo asymmetric routes to functionalizable constrained amino acid building blocks are almost unknown. Indeed, in many of these cases, the side chain is sacrificed during the ring-forming process. We envisaged that the allylation of azlactones using an in situ generated Pd zwitterion reported by our group^6^ and others^7^ would allow the ready assembly of lactam monomers that were appropriately armed for incorporation into peptide motifs. As shown in Figure 1c, these compounds would be amenable to N to C homologation by lactam alkylation, peptide coupling, and tagging at the olefin moiety. We report herein our progress toward the enantioselective synthesis of these constrained amino acids and their incorporation into small peptide arrays.

**Figure 1:**
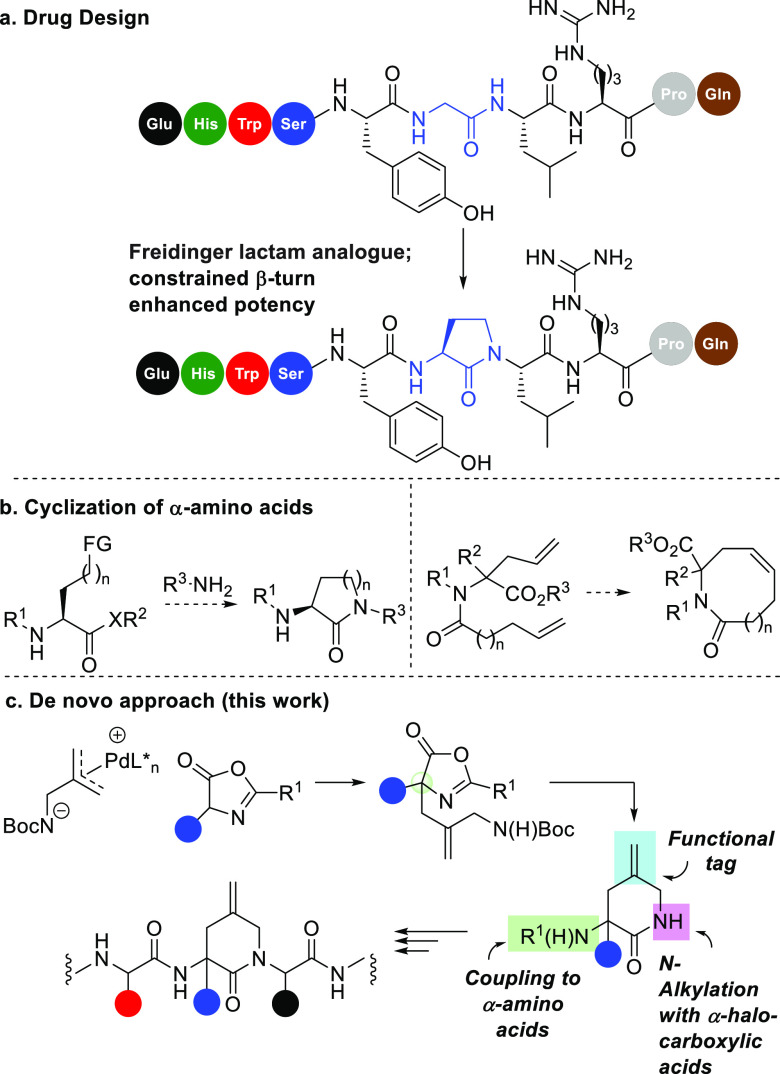
Synthetic approaches to Freidinger lactams.

We began our studies by investigating the key lactam-forming transformation, and our results are shown in Figure 2a. Pleasingly, carbamate **2a** and 6-substituted analogue **2b** were efficiently transformed in to lactams **3** and **4a**, respectively. Notably, **4a** is formed at the expense of isomer **4b** with excellent *E*/*Z* selectivity, an observation we attribute to a combination of minimization of steric control and allylic strain, as highlighted in **I**. At the outset of this work, we recognized the importance of establishing a method that would access the constrained amino acid building blocks with synthetically useful enantiomeric ratios. On the basis that the Pd-catalyzed asymmetric allylation and benzylation of azlactones are successful using the Trost ligand series,^8^ we used these ligands to screen and optimize the enantioselectivity of this transformation. As shown in Figure 2b, we identified **L4** as providing the most selective catalyst system, and further optimization led to conditions that generated **3** in both high yield and good enantiocontrol. Unfortunately, compound **4a** could not be accessed with useful levels of enantiocontrol, despite a range of chiral ligands having been screened.

**Figure 2:**
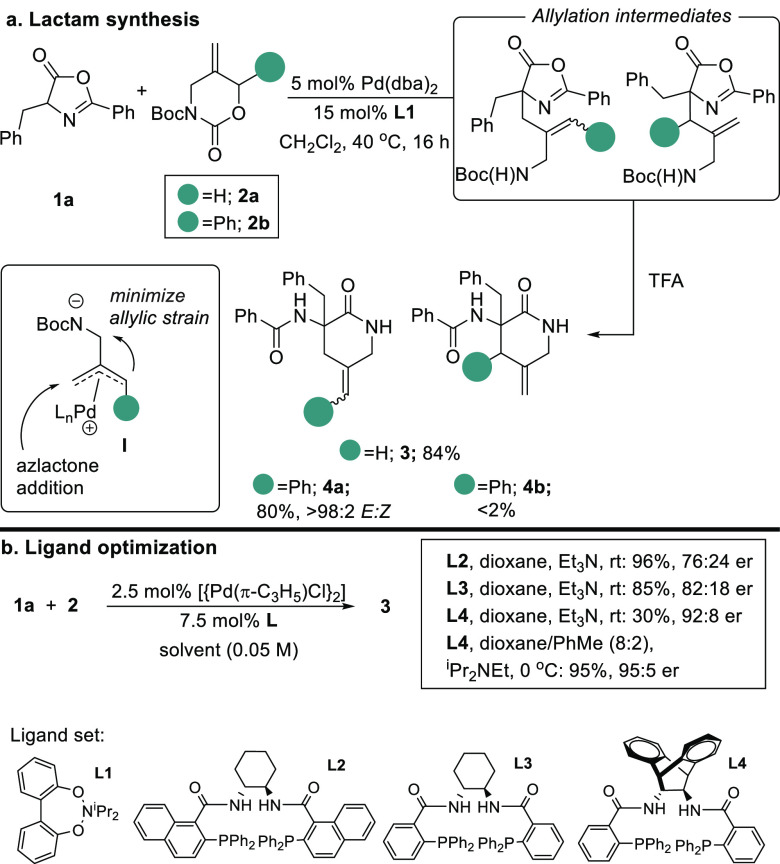
Development of the lactam-forming transformation and optimization of the reaction enantioselectivity.

We next set out to explore the scope of the method for generating simple lactam-constrained mimics of representative natural α-amino acids, and our results are summarized in Table 1. In general, we were able to incorporate the key 2-aminomethyl allyl fragment with enantiomeric ratios of 90:10 or better, and these could be smoothly transformed into the corresponding lactams after removal of the Boc group with TFA. The methodology showed good generality, delivering lactam mimics bearing both polar and hydrophobic side chains. The exception was **12**, which underwent the asymmetric allylation step with both a poor yield and poor enantioselectivity. In addition, we were able to crystallize lactams **3** and **5**, and both showed the *R* configuration at the newly generated stereogenic center. The remaining compounds were assigned by inference. In addition, we recognized that this methodology offered the opportunity to directly prepare FL_x_-Gly (FL, Freidinger lactam; x, α-amino acid mimic label) dipeptides by replacing the azlactone Ph substituent at C2 with an aminomethyl group. Pleasingly, subjecting azlactones **13**–**15** to our optimized conditions generated FL_Phe_-Gly dipeptides with or without a Cbz protecting group and Cbz-protected FL_Leu_-Gly, both with high enantiomeric ratios.^9^

**Table 1:**
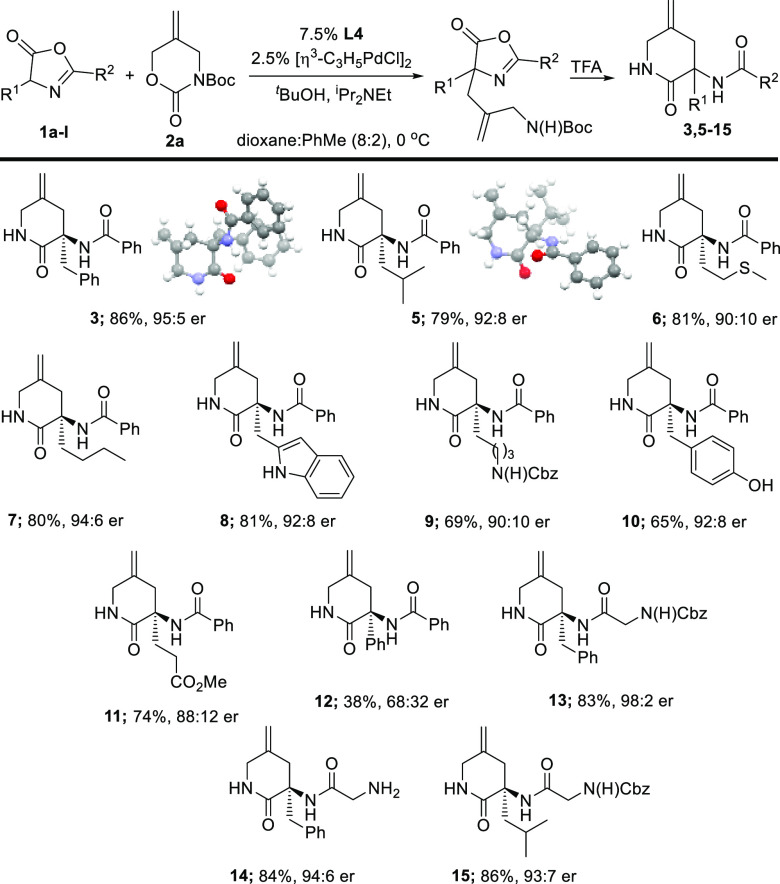
Scope of the Enantioselective Lactam Synthesis^a^

a X-ray structures show thermal ellipsoids drawn at the 50% probability level.

As shown in Figure 3, we have applied the model developed by Lloyd-Jones and Norrby^10^ to put forward a rationale for the observed stereochemical outcome of the allylic alkylation process. The enantioselective step is mediated by a H-bond interaction between the azlactone enolate and the ligand N–H that allows the addition to take place with the aromatic substituent oriented away from the catalyst “roof”. The alternative mode of addition places both substituents at C2 and C4 in the proximity of the ligand structure.

**Figure 3:**
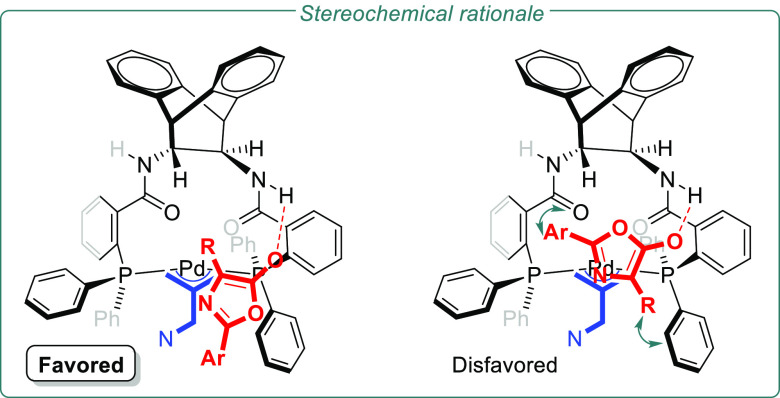
Proposed origin of the reaction enantioselectivity.

While the substrates prepared in Table 1 verified that the basic methodology could allow access to enantiomerically enriched constrained amino acid building blocks, we were concerned that the generation of the products as benzamides would hamper their further elaboration due to the difficulties associated with hydrolyzing this group. In this context, Connon and co-workers designed a family of azlactones that offer mild hydrolysis protocols via formation of phthalimide intermediates and set out to investigate whether this chemistry could offer a useful solution to this problem.^11^ Pleasingly, as shown in Table 2, we were able to transform azlactones **1m**–**1q** into the corresponding free amine building blocks **16**–**20**, respectively, through a simple three-step sequence, generating the products in good yield and enantiocontrol. Interestingly, the product derived from glutamic acid-based azlactone **1r** underwent further cyclization when subjected to this sequence, generating functionalized spirolactam **21**.

**Table 2:**
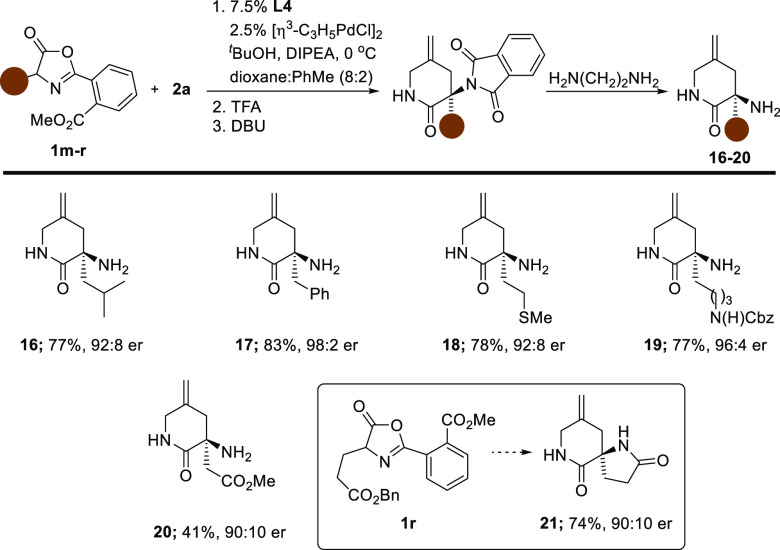
Scope of Free 3-Aminolactam Scaffolds

We next took the opportunity to explore the functionalization of the alkene. As shown in Figure 4, we protected the amino group in **16** and carried out a homologation to generate ^*t*^Bu-Gly-FL_Leu_-Boc **22**. Oxidative cleavage of the alkene followed by diastereoselective reduction^12^ provided an alcohol unit that was readily elaborated to the corresponding propargyl ether **24**, offering a means for these lactams to be easily tagged to other molecules through “click” chemistry. In addition, **22** also underwent efficient epoxidation and hydrogenation reactions, albeit with modest diastereocontrol.

**Figure 4:**
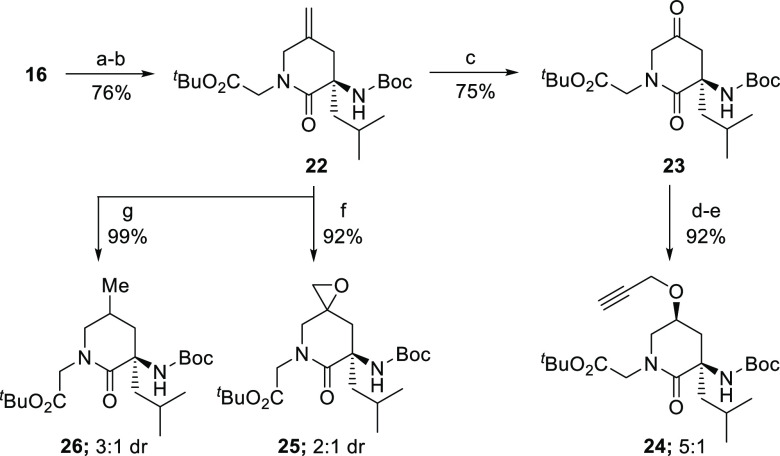
Reaction conditions: ^*a*^Boc_2_O, Et_3_N, THF/H_2_O (1:2); ^*b*^(i) LHMDS; (ii) BrCH_2_CO_2_^*t*^Bu; ^*c*^RuCl_3_ (30 mol %), NaIO_4_, MeCN/CH_2_Cl_2_/H_2_O (1:1:2); ^*d*^K-selectride, THF; ^*e*^HCCCH_2_Br, ^*n*^Bu_4_NSO_4_, NaOH, toluene; ^*f*^Oxone, NaHCO_3_, acetone/H_2_O; ^*g*^H_2_/Pd/C.

Finally, to demonstrate the applicability of this chemistry to the generation of peptidomimetics, we targeted the synthesis of a constrained analogue of melanocyte-inhibiting factor (MIF-1).^13^ MIF-1 is a hypothalamic neuropeptide derived endogenously by cleavage of the hormone oxytocin. This tripeptide displays a range of bioactivities and has been studied for the treatment of Parkinson’s disease as well as for its antidepressant and nootropic activities. As shown in Figure 5, we derivatized FL_Leu_**5** toward MIF-1 analogue **28** within a short synthetic sequence. During this synthesis, we were able to grow suitable crystals of **27** for X-ray crystallographic analysis, and this compound showed the 10-membered glycinamide–proline hydrogen bond and dihedral angles that are consistent with type II β-turns.^14^ Notably, the H-bond interaction between the C-terminal glycinamide hydrogen and the prolyl carbonyl oxygen has been observed in MIF-1 both in solution and in the solid state,^15^ highlighting that these lactam mimics can deliver peptides that maintain key secondary structural features.

**Figure 5:**
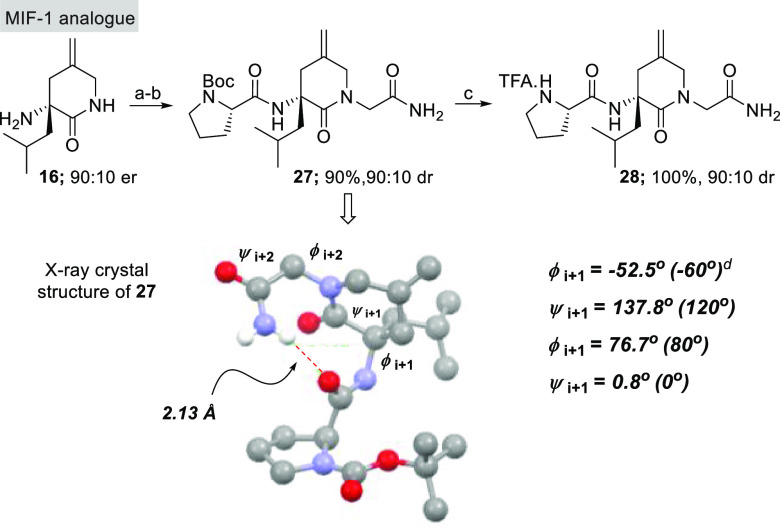
*^a^*EDCI, HOAt, ^i^Pr_2_NEt, Boc-l-Pro-OH, CH_2_Cl_2_; ^*b*^LHMDS, ICH_2_C(O)NH_2_, DMF; ^*c*^TFA. ^*d*^Torsional angles in parentheses are those for an ideal type II β-turn. The X-ray structure shows thermal ellipsoids drawn at the 50% probability level.

We developed a de novo asymmetric route to lactam-constrained α-amino acid building blocks bearing a range of polar and hydrophobic side chains. As well as enforcing type II β-turn conformations, these intermediates are armed with an exocyclic alkene that provides a handle for ligation via “click” chemistry providing a platform for a range of tailored applications. Studies in this area are ongoing and will be reported in due course.

## Data Availability

The data underlying this study are available in the published article and its Supporting Information.

## Supporting Information Available

Synthesis and characterization details, ^1^H and ^13^C NMR spectra, FTIR and HRMS data, HPLC traces, and X-ray crystal structure data (PDF)

## References

[ref1] Muttenthaler M.; King G. F.; Adams D. J.; Alewood P. F. Trends in peptide drug discovery. Nat. Rev. Drug Discovery 2021, 20, 309–325. 10.1038/s41573-020-00135-8. 33536635

[ref2] aJwad R.; Weissberger D.; Hunter L. Strategies for fine-tuning the conformations of cyclic peptides. Chem. Rev. 2020, 120, 9743–9789. 10.1021/acs.chemrev.0c00013. 32786420

[ref3] aFreidinger R. M.; Veber D. F.; Perlow D. S.; Brooks J. R.; Saperstein R. Bioactive conformation of luteinizing hormone-releasing hormone: evidence from a conformationally constrained analog. Science. 1980, 210, 656–658. 10.1126/science.7001627. 7001627

[ref4] aRao M. H. V.; Pinyol E.; Lubell W. D. Rigid Dipeptide Mimics: Synthesis of Enantiopure C6-Functionalized Pyrrolizidinone Amino Acids. J. Org. Chem. 2007, 72, 736–743. 10.1021/jo0616761. 17253788

[ref5] Hoffmann T.; Lanig H.; Waibel R.; Gmeiner P. Rational Molecular Design and EPC Synthesis of a Type VI β-Turn Inducing Peptide Mimetic. Angew. Chem., Int. Ed. 2001, 40, 3361–3364. 10.1002/1521-3773(20010917)40:18<3361::AID-ANIE3361>3.0.CO;2-9. 11592138

[ref6] aAllen B. D. W.; Connolly M. J.; Harrity J. P. A. A Pd-Catalyzed Synthesis of Functionalized Piperidines. Chem. - Eur. J. 2016, 22, 13000–13003. 10.1002/chem.201602586. 27273202

[ref7] aLi K.; Zhen S.; Wang W.; Du J.; Yu S.; Wu Y.; Guo H. Fungicide-inspired precursors of π-allylpalladium intermediates for palladium-catalyzed decarboxylative cycloadditions. Chem. Sci. 2023, 14, 3024–3029. 10.1039/D3SC00112A. 36937593 PMC10016346

[ref8] aTrost B. M.; Ariza X. Enantioselective allylations of azlactones with unsymmetrical acyclic allyl esters. J. Am. Chem. Soc. 1999, 121, 10727–10737. 10.1021/ja992754n.

[ref9] Enantiomeric ratios of lactams were based on the values measured for the allylation precursors. We validated this assumption in the cases of **3a/4a** and **3b/4b** in which the enantiomeric purities were recorded for the allylation product and lactam, respectively, and were found to be unchanged.

[ref10] Butts C. P.; Filali E.; Lloyd-Jones G. C.; Norrby P. O.; Sale D. A.; Schramm Y. Structure-Based Rationale for Selectivity in the Asymmetric Allylic Alkylation of Cycloalkenyl Esters Employing the Trost ‘Standard Ligand’ (TSL): Isolation, Analysis and Alkylation of the Monomeric form of the Cationic η^3^-Cyclohexenyl Complex [(η^3^-c-C_6_H_9_)Pd(TSL)]^+^. J. Am. Chem. Soc. 2009, 131, 9945–9957. 10.1021/ja8099757. 19435358

[ref11] Tallon S.; Manoni F.; Connon S. J. A Practical Aryl Unit for Azlactone Dynamic Kinetic Resolution: Orthogonally Protected Products and A Ligation-Inspired Coupling Process. Angew. Chem., Int. Ed. 2015, 54, 813–817. 10.1002/anie.201406857. 25425156

[ref12] Tentative assignent of the stereochemistry of **9** has been made on the basis of NOE spectroscopy. See the Supporting Information for further details.

[ref13] aNair R. M. G.; Kastin A. J.; Schally A. V. Isolation and structure of hypothalamic MSH release-inhibiting hormone. Biochem. Biophys. Res. Commun. 1971, 43, 1376–1381. 10.1016/S0006-291X(71)80026-8. 4398196

[ref14] Deposition Numbers 2224755 (**3**), 2284781 (**5**), and 2224762 (**27**) contain the supplementary crystallographic data for this paper. These data are provided free of charge by the joint Cambridge Crystallographic Data Centre and Fachinformationszentrum Karlsruhe Access Structures service www.ccdc.cam.ac.uk/structures.

[ref15] aWalter R.; Bernal I.; Johnson L. F. Chemistry and Biology of Peptides; Ann Arbor Science Publishers: Ann Arbor, MI, 1972; pp 131–135.

