# Supplementary material for: Asymmetric Synthesis of Functionalizable Type II β-Turn-Inducing α-Amino Acid Building Blocks
Source: Org Lett. 2023 Aug 29;25(35):6555–9. doi: 10.1021/acs.orglett.3c02376 (PMC10496131; doi:10.1021/acs.orglett.3c02376)
Supplement: Supplementary file 1 — ol3c02376_si_001.pdf [file ol3c02376_si_001.pdf]

# Asymmetric Synthesis of Functionalizable Type II β-Turn Inducing α-Amino Acid Building Blocks

Wenzheng Gao, Jiaxin Han, Sophie Greaves and Joseph P. A. Harrity*

Department of Chemistry, University of Sheffield, Sheffield, S3 7HF, United Kingdom

## Table of Contents

General Considerations ........ 1
Experimental Procedures ........ 2
Synthesis of Azlactones ........ 2
Allylation Products ........ 18
Synthesis of Lactams ........ 27
Synthesis of benzamide-free lactam building blocks ........ 45
Synthesis of 2b and 4a ........ 49
Synthesis of Alkyne Tagged Lactam ........ 53
Synthesis of MIF-1 analogue ........ 58
Gram scale synthesis ........ 60
NMR Spectra ........ 61
nOe Analysis of compound 9 and 12 ........ 127
HPLC traces of enantioenriched products ........ 131
X-ray crystallography details ........ 151
References ........ 172

## General Considerations

All reactions were carried out in flame-dried glassware under high vacuum, unless stated otherwise. For reactions carried out under an inert atmosphere, solvents were purified using a PureSolv MD purification system and transferred under nitrogen. A DrySyn block combined with a temperature probe was used as the heating source, where required. Infrared (IR) spectra were recorded on a Perkin Elmer Paragon FTIR spectrometer ($\nu_{max}$/cm$^{-1}$). Samples were recorded neat as thin films. $^{1}$H NMR spectra were recorded on a Bruker AVIII HD 400 (400 MHz), Bruker AVI 400 (400 MHz) or Bruker AMX400 (400 MHz). Chemical shifts are reported in parts per million (ppm) from tetramethylsilane, using the residual protic solvent resonance as the internal reference: ($CHCl_3$: δ 7.26 ppm, MeOH: δ 3.31 ppm) unless otherwise stated. Data are reported as follows: chemical shift, multiplicity (s = singlet, d = doublet, t = triplet, q = quartet, br = broad, m = multiplet), coupling constant (Hz), integration). $^{13}$C NMR spectra were recorded on a Bruker AVIII HD 400 (101 MHz), Bruker AVI 400 (101 MHz) or Bruker AMX-400 (101 MHz) with broadband proton decoupling. Structural assignments were made with additional information from $^{13}$C DEPTQ experiments. Chemical shifts are reported in ppm from tetramethylsilane with the solvent as the internal reference ($CDCl_3$: δ 77.16 ppm, $CD_3OD$: δ 49.00 ppm). $^{19}$F NMR spectra were recorded on a Bruker AVIII HD 400 (128 MHz). High-resolution mass spectra (HRMS) recorded for accurate mass analysis, were performed on a Micromass LCT operating in electrospray mode (TOF, ESI+, ESI–). Thin layer chromatography (TLC) was performed on aluminium-backed plates pre-coated with silica (0.2 mm, Merck 60 F254) which were developed using standard visualizing agents: UV light or potassium permanganate. Flash chromatography was performed on silica gel (Merck 40-63 µm) or Florisil® (60-100 mesh). Melting points were recorded on Gallenkamp melting point apparatus and are uncorrected.

Carbamate **2a** and ligand **L1** was prepared according to procedures previously described.[1]

Optical rotations were recorded for enantioenriched compounds of 95:5 er or better.

**Experimental Procedures**

**Synthesis of Azlactones**

**General Procedure 1**

The amino acid (1 eq) was dissolved in 1 M NaOH (aq) and to this solution was added the acid chloride (1.2 eq) in 1,4-dioxane slowly over a period of 5 min. The reaction mixture was allowed to stir at 75 °C (DrySyn block) for 30 min, then acidified to pH~2 using conc. HCl and extracted using EtOAc. The solvent was removed under reduced pressure to give the *N*-acylated amino acid which was used directly in the next step as crude material.

**General Procedure 2**

The amino acid (1 eq) was dissolved in THF and the acid chloride (1.2 eq) was added dropwise. The reaction mixture was stirred at 80 °C (DrySyn block) overnight then cooled to room temperature. This mixture was extracted with EtOAc and the solvent removed under reduced pressure to give the *N*-acylated amino acid which was used directly in the next step as crude material.

**General Procedure 3**

The *N*-acylated amino acid (1 eq) was dissolved in dry DCM under an inert atmosphere and cooled to 0 °C. To this solution, EDC.HCl (1.2 eq) was added in one portion. The reaction mixture was allowed to warm to room temperature and stirred overnight. The reaction was quenched with sat. aq. $NaHCO_3$ and extracted with DCM, and the solvent removed under reduced pressure. The resulting residue was then purified by flash column chromatography to give the azlactone.

**General Procedure 4**

The *N*-acylated amino acid was dissolved in $Ac_2O$ (10 mL) under nitrogen atmosphere and stirred at 90 °C (DrySyn block) for 30 minutes. After cooling to room temperature, the reaction mixture was quenched with sat. aq. $NaHCO_3$ and the product extracted with $Et_2O$. The solvent was removed under reduced pressure and the crude residue was purified by flash column chromatography to give the azlactone.

**2-Phenyl-4-benzyl-2-oxazolin-5-one, 1a**[2]

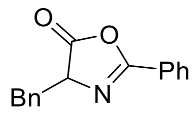

Phenylalanine (1.07 g, 6.5 mmol) in 1 M NaOH (aq) (8 mL) and PhCOCl (0.77 mL, 6.6 mmol) were subjected to general procedure 1. The crude *N*-acylated amino acid and EDC.HCl (1.42 g, 7.4 mmol) in dry DCM (20 mL), were subjected to general procedure 3 and the crude residue was purified by flash column chromatography eluting with 2% EtOAc in petrol to give the azlactone 2-phenyl-4-benzyl-2-oxazolin-5-one (**1a**) (696 mg, 43%) as a white amorphous solid.

**$^1$H NMR (400 MHz, $CDCl_3$)** δ 7.98-7.91 (m, 2H, $CH_{Ar}$), 7.63-7.53 (m, 1H, $CH_{Ar}$), 7.52-7.44 (m, 2H, $CH_{Ar}$), 7.34-7.19 (m, 5H, $CH_{Ar}$), 3.72 (dd, J = 6.5, 5.0 Hz, 1H, CH), 3.40 (dd, J = 14.0, 5.0 Hz, 1H, $CH_2$), 3.22 (dd, J = 14.0, 6.5 Hz, 1H, $CH_2$); **$^{13}$C NMR (101 MHz, $CDCl_3$)** δ 177.6, 161.7, 135.3, 132.7, 129.6, 128.7, 128.4, 127.9, 127.2, 125.8, 66.6, 37.3. Data is consistent with literature.

### 2-Phenyl-4-isobutyl-2-oxazolin-5-one, 1b[2]

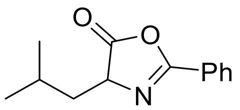


Leucine (2.0 g, 15.25 mmol) in 1 M NaOH (aq) (20 mL) and PhCOCl (2.1 mL, 18.3 mmol) were subjected to general procedure 1. The crude *N*-acylamino acid was dissolved in $Ac_2O$ (28 mL, 0.23 mol) and subjected to general procedure 4 and the crude residue was purified by flash column chromatography eluting with 6% $Et_2O$ in petrol to give the azlactone 2-phenyl-4-isobutyl-2-oxazolin-5-one (**1b**) (1.8 g, 55%) as an amorphous solid.

**$^1$H NMR (400 MHz, $CDCl_3$)** δ 8.05-7.98 (m, 2H, $CH_{Ar}$), 7.62-7.56 (m, 1H, $CH_{Ar}$), 7.54-7.47 (m, 2H, $CH_{Ar}$), 4.43 (dd, 1H, J = 9.0, 6.0 Hz, CH), 2.15-2.02 (m, 1H, CH), 1.86 (ddd, J = 13.5, 8.0, 6.0 Hz, 1H, $CH_2$), 1.70 (ddd, J = 13.5, 9.0, 6.0 Hz, 1H, $CH_2$), 1.06 (d, *J* = 6.5 Hz, 3H, $CH_3$), 1.03 (d, *J* = 6.5 Hz, 1H, $CH_3$); **$^{13}$C NMR (101 MHz, $CDCl_3$)** δ 179.0, 161.4, 132.7, 128.8, 127.9, 126.1, 64.0, 40.8, 25.2, 22.8, 22.1. Data is consistent with literature.

### 2-Phenyl-4-[2-(methylthio)ethyl)]-2-oxazolin-5-one, 1c[2]

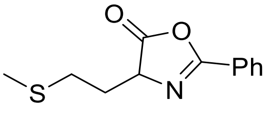


Methionine (1.05 g, 7.0 mmol) in 1 M NaOH (aq) (10 mL) and PhCOCl (1 mL, 8.6 mmol) were subjected to general procedure 1. The crude *N*-acylamino acid in $Ac_2O$ (10 mL, 0.11 mmol) was subjected to general procedure 4 and the crude residue was purified by flash column

chromatography eluting with 5% EtOAc in petrol to give the azlactone 2-phenyl-4-[2-(methylthio)ethyl)]-2-oxazolin-5-one (**1c**) (836 mg, 50%) as a colourless oil.

**$^1$H NMR (400 MHz, $CDCl_3$)** δ 8.04-7.96 (m, 2H, $CH_{Ar}$), 7.65-7.53 (m, 1H, $CH_{Ar}$), 7.48 (t, J = 7.5 Hz, 2H, $CH_{Ar}$), 4.61 (dd, J = 7.0, 6.0 Hz, 1H, CH), 2.73 (t, J = 7.0 Hz, 2H, $SCH_2$), 2.41-2.25 (m, 1H, $CH_2CH$), 2.24-2.06 (m, 1H, $CH_2CH$), 2.11 (s, 3H, $CH_3$); **$^{13}$C NMR (101 MHz, $CDCl_3$)** δ 175.7, 162.3, 128.9, 128.0, 127.2, 125.8, 63.7, 30.4, 30.0, 15.1. Data is consistent with literature.

**2-Phenyl-4-butyl-2-oxazolin-5-one, 1d**[2]

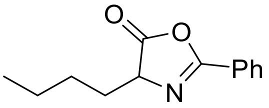


Norleucine (922 mg, 7.0 mmol) in 1 M NaOH (aq) (10 mL) then PhCOCl (0.96 mL, 1.2 eq, 8.2 mmol) were subjected to general procedure 1. The crude *N*-acylamino acid was dissolved in $Ac_2O$ (10 mL, 15 eq, 0.11 mol) and subjected to general procedure 4 and the crude residue was purified by flash column chromatography eluting with 5% EtOAc in petrol to give the azlactone 2-phenyl-4-butyl-2-oxazolin-5-one (**1d**) (802 mg, 53%) as a white amorphous solid.

**$^1$H NMR (400 MHz, $CDCl_3$)** δ 8.02 (dd, J = 7.0, 1.5 Hz, 2H, $CH_{Ar}$), 7.65-7.56 (m, 1H, $CH_{Ar}$), 7.55-7.46 (m, 2H $CH_{Ar}$), 4.47-4.37 (m, 1H, CH), 2.16-1.97 (m, 1H, $CH_2CH$), 1.97-1.77 (m, 1H, $CH_2CH$), 1.58- 1.34 (m, 4H, $CH_2$), 0.94 (t, J = 7.0 Hz, 3H, $CH_3$); **$^{13}$C NMR (101 MHz, $CDCl_3$)** δ 176.2, 167.7, 133.6, 132.9, 132.0, 128.9, 65.3, 31.3, 27.2, 22.3, 13.8. Data is consistent with literature.

**2-Phenyl-4-((1H-indol-2-yl)methyl)-2-oxazolin-5-one, 1e**[3]

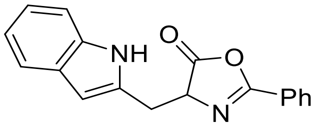


Tryptophan (2.5 g, 12.0 mmol) in 1 M NaOH (aq) (20 mL) then PhCOCl (1.67 mL, 14.4 mmol) were subjected to general procedure 2. The crude *N*-acylamino acid was dissolved in $Ac_2O$ (23 mL, 0.18 mol) and subjected to general procedure 4 and the crude residue was purified by flash column chromatography eluting with 20% EtOAc in petrol to give the azlactone 2-phenyl-4-((1H-indol-2-yl)methyl)-2-oxazolin-5-one (**1e**) (2.6 g, 74%) as a yellow amorphous solid.

$^{1}$**H NMR (400 MHz, CDCl$_3$)** δ 8.04 (s, 1H, NH), 7.95-7.87 (m, 2H, $CH_{Ar}$), 7.78-7.79 (m, 1H, $CH_{Ar}$), 7.54 (t, J = 7.5 Hz, 1H, $CH_{Ar}$), 7.44 (t, J = 7.5 Hz, 2H, $CH_{Ar}$), 7.34-7.26 (m, 1H, $CH_{Ar}$), 7.21-7.10 (m, 3H, $CH_{Ar}$), 4.78 (dd, J = 6.0, 5.0 Hz, 1H, CH), 3.56 (dd, J = 15.0, 5.0 Hz, 1H, $CH_2$), 3.43 (dd, J = 15.0, 6.0 Hz, 1H, $CH_2$); $^{13}$**C NMR (101 MHz, CDCl$_3$)** δ 178.0, 161.9, 136.0, 132.7, 128.7, 127.9, 127.4, 125.8, 123.5, 122.1, 119.6, 119.2, 111.1, 109.6, 66.6, 27.3. Data is consistent with literature.

**Benzyl (4-(5-oxo-2-phenyl-4,5-dihydrooxazol-4-yl)butyl)carbamate, 1f**

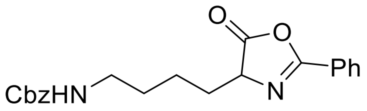


H-Lys(Z)-OH (2.8 g, 10 mmol) in THF (10 mL) then PhCOCl (1.4 mL, 12 mmol) were subjected to general procedure 2. The crude *N*-acylamino acid and EDC.HCl (2.3 g, 12 mmol) in dry DCM (40 mL) were subjected to general procedure 3. The crude residue was purified by flash column chromatography eluting with 30% EtOAc in petrol to give the azlactone benzyl (4-(5-oxo-2-phenyl-4,5-dihydrooxazol-4-yl)butyl)carbamate (**1f**) (3.0 g, 80%) as a colourless oil.

**FTIR** $\nu_{max}$ (thin film/cm$^{-1}$ ) 3334, 3062, 1821, 1702, 1652; $^{1}$**H NMR (400 MHz, CDCl$_3$)** δ 8.05-7.99 (m, 2H, $CH_{Ar}$), 7.60 (t, J = 7.5 Hz, 1H, $CH_{Ar}$), 7.51 (t, J = 7.5 Hz, 2H, $CH_{Ar}$), 7.41-7.29 (m, 5H, $CH_{Ar}$), 5.11 (s, 2H, $CH_2$), 4.86 (s, 1H, NH), 4.42 (t, 1H, J = 6.5 Hz, CH), 3.31-3.09 (m, 2H, $CH_2$), 2.17-1.97 (m, 1H, $CH_2$), 1.96-1.77 (m, 1H, $CH_2$), 1.71-1.42 (m, 4H, $CH_2CH_2$); $^{13}$**C NMR (101 MHz, CDCl$_3$)** δ 178.3, 161.7, 156.5, 136.7, 132.8, 128.8, 128.5, 128.1 (×2 C), 127.9, 125.9, 66.6, 66.2, 40.7, 31.2, 29.5, 22.6. **MS (ESI$^+$)** 367 (100%, M+H$^+$); **HRMS (ESI-TOF)** m/z: [M + H]$^+$ Calcd for $C_{21}H_{23}N_2O_4$ 367.1652; Found 367.1659.

**2-Phenyl-4-(4-hydroxybenzyl)-2-oxazolin-5-one, 1g**[3]

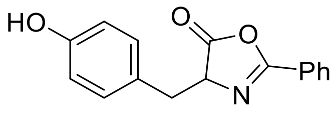


Tyrosine (1.8 g, 10 mmol) in THF (10 mL) then PhCOCl (1.4 mL, 12 mmol) were subjected to general procedure 2. The crude *N*-acylamino acid and EDC.HCl (2.3 g, 12 mmol) in dry DCM (40 mL) were subjected to general procedure 3. The crude residue was purified by flash

column chromatography eluting with 40% EtOAc in petrol to give the azlactone 2-phenyl-4-(4-hydroxybenzyl)-2-oxazolin-5-one (**1g**) (1.6 g, 60%) as a colourless oil.

**$^1$H NMR (400 MHz, CDCl$_3$)** δ 7.97-7.90 (m, 2H, $CH_{Ar}$), 7.62-7.53 (m, 1H, $CH_{Ar}$), 7.50-7.41 (m, 2H, $CH_{Ar}$), 7.15-7.07 (m, 2H, $CH_{Ar}$), 6.75-6.65 (m, 2H, $CH_{Ar}$), 5.77 (s, 1H, OH), 4.69 (dd, J = 6.5, 5.0 Hz, 1H, CH), 3.33 (dd, J = 14.0, 5.0 Hz, 1H, $CH_2$), 3.14 (dd, J = 14.0, 6.5 Hz, 1H, $CH_2$); **$^{13}$C NMR (101 MHz, CDCl$_3$)** δ 177.5, 162.1, 155.0, 132.9, 130.8, 128.8, 128.0, 126.9, 125.6, 115.4, 66.7, 36.5. Data is consistent with literature.

**2-Phenyl-4-methyl propanoate-2-oxazolin-5-one, 1h**[2]

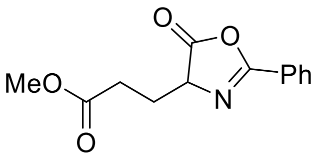


H-Glu(OMe)-OH (1.6 g, 10 mmol) in THF (10 mL) then PhCOCl (1.4 mL, 12 mmol) were subjected to general procedure 2. The crude *N*-acylamino acid and EDC.HCl (2.3 g, 1.2 eq, 12 mmol) in dry DCM (40 mL) were subjected to general procedure 3. The crude residue was purified by flash column chromatography eluting with 30% EtOAc in petrol to give the azlactone 2-phenyl-4-methyl propanoate-2-oxazolin-5-one (**1h**) (870 mg, 35%) as a colourless oil.

**$^1$H NMR (400 MHz, CDCl$_3$)** δ 8.05-7.98 (m, 2H, $CH_{Ar}$), 7.65-7.56 (m, 1H, $CH_{Ar}$), 7.55-7.47 (m, 2H, $CH_{Ar}$), 4.53 (dd, J= 8.0, 6.0 Hz, 1H, CH), 3.70 (s, 3H, $CH_3$), 2.62 (t, J = 7.5 Hz, 2H, $CH_2$), 2.46-2.33 (m, 1H, $CH_2$), 2.17 (dq, J = 15.0, 7.5 Hz, 1H, $CH_2$); **$^{13}$C NMR (101 MHz, CDCl$_3$)** δ 177.9, 172.7, 162.1, 132.9, 128.8, 128.0, 125.7, 64.2, 51.8, 29.8, 26.7. Data is consistent with literature.

**2-Phenyl-4-phenyl-2-oxazolin-5-one, 1i**[3]

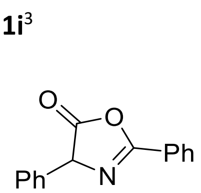


Phenylglycine (2.06 g, 1.0 eq, 14 mmol) in 1 M NaOH(aq) (15 mL) and PhCOCl (1.7 mL, 1.1 eq, 15 mmol) in 1,4-dioxane (15 mL) were subjected to general procedure 1. The crude N-acylated amino acid and EDC.HCl (2.81 g, 1.1 eq, 15 mmol) in dry DCM (40 mL) were subjected to general procedure D and the crude residue was purified by flash column chromatography

eluting with a gradient of 2-5% EtOAc in petrol to give the 2-Phenyl-4-phenyl-2-oxazolin-5-one (**1i**) (679 mg, 21%) as a yellow amorphous solid.

**$^1$H NMR (400 MHz, CDCl$_3$)** δ 8.13-8.08 (2H, m, $CH_{Ar}$), 7.68-7.36 (8H, m, $CH_{Ar}$), 5.83 (1H, m, CH); **$^{13}$C NMR (101 MHz, CDCl$_3$)** δ 176.3, 162.6, 133.5, 133.1, 129.2, 129.1, 128.9, 128.2, 127.4, 125.7, 68.2. Data is consistent with literature.

**Benzyl (4-benzyl-5-oxo-4,5-dihydro-oxazol-2-ylmethyl)-carbamic acid, 1j**[2]

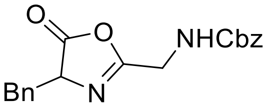


Z-Gly-Phe-OH (500 mg, 1.4 mmol) and EDC.HCl (351 mg, 1.8 mmol) in dry DCM (20 mL) were subjected to general procedure 3. The crude residue was purified by flash column chromatography eluting with a gradient of 20-40% EtOAc in petrol to give the azlactone benzyl (4-benzyl-5-oxo-4,5-dihydro-oxazol-2-ylmethyl)-carbamic acid (**1j**) (139 mg, 29%) as a colourless oil.

**$^1$H NMR (400 MHz, CDCl$_3$)** δ 7.45-7.22 (m, 10H, $CH_{Ar}$), 5.97 (s, 1H, NH), 5.16-5.05 (m, 2H, $CH_2$), 4.95 (t, J = 5.5 Hz, 1H, $CH_2$), 3.99-3.87 (m, 2H, $CH_2$), 3.83- 3.68 (m, 1H, $PhCH_2CH$) 3.68-3.47 (m, 1H, $PhCH_2CH$); **$^{13}$C NMR (101 MHz, CDCl$_3$)** δ 164.9, 164.3, 156.1, 136.1, 133.7, 129.3, 128.9, 128.6, 128.3, 128.2, 127.5, 98.0, 67.2, 43.2, 34.3. Data is consistent with literature.

***tert*-Butyl (4-benzyl-5-oxo-4,5-dihydro-oxazol-2-ylmethyl)-carbamic acid, 1k**[2]

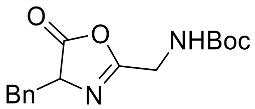


Glycylphenylalanine (1.04 g, 4.7 mmol) and $NaHCO_3$ (1.18 g, 14 mmol) were dissolved in water (14 mL) and THF (14 mL). To this solution, $Boc_2O$ (1.20 g, 5.5 mmol) was added and the reaction was stirred at room temperature overnight. The reaction mixture was washed with $Et_2O$ (20 mL), then acidified to pH~2 with 1 M HCl (aq). The acidified crude mixture was extracted with EtOAc (2x20 mL), the organic phases were combined and dried with $MgSO_4$, then filtered and the solvent removed under reduced pressure to give crude Boc-glycylphenylalanine (600 mg, 57%).

Boc-Glycylphenylalanine (207 mg, 0.64 mmol) in dry DCM (10 mL) under a nitrogen atmosphere was cooled to 0 °C, and DCC (146 mg, 0.71 mmol) was added. The mixture was stirred for 4 hours, then filtered to remove the urea by-product. The crude residue was concentrated and a minimal amount of DCM was added to re-dissolve the residue. This was then stored in the freezer overnight to precipitate the remaining starting materials, and filtered once more. The solvent was removed to give the azlactone *tert*-butyl (4-benzyl-5-oxo-4,5-dihydro-oxazol-2-ylmethyl)-carbamic acid (**1k**) (161 mg, 83%) as a white cloudy oil.

**$^1$H NMR (400 MHz, CDCl$_3$)** δ 7.35-7.17 (m, 5H, $CH_{Ar}$), 5.05 (s, 1H, NH), 4.48 (m, 1H, CH), 4.02 (d, J = 3.5 Hz, 2H, $CH_2$), 3.27 (dd, J = 14.0, 5.0 Hz, 1H, $PhCH_2$), 3.09 (dd, J = 14.0, 6.5 Hz, 1H, $PhCH_2CH$), 1.47 (s, 9H, $C(CH_3)_3$); **$^{13}$C-NMR (101 MHz, CDCl$_3$)** 176.9, 162.9, 155.4, 134.8, 129.5, 128.5, 127.4, 80.4, 65.5, 38.3, 36.8, 28.3. Data is consistent with literature.

**Benzyl (4-isobutyl-5-oxo-4,5-dihydro-oxazol-2-ylmethyl)-carbamic acid, 1l**[4]

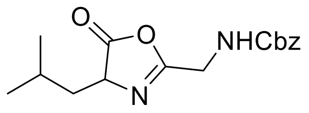


Z-Gly-Lys-OH (3.22 g, 10 mmol) and EDC.HCl (2.30 g, 12 mmol) in dry DCM (20 mL) were subjected to general procedure 3. The crude residue was purified by flash column chromatography eluting with a gradient of 70% $Et_2O$ in petrol to give the azlactone benzyl (4-isobutyl-5-oxo-4,5-dihydro-oxazol-2-ylmethyl)-carbamic acid (**1l**) (1.10 g, 37%) as a colourless oil.

**$^1$H NMR (400 MHz, CDCl$_3$)** δ 7.45-7.29 (m, 5H, $CH_{Ar}$), 5.96 (s, 1H, NH), 5.23-4.97 (m, 3H, $CH_2$ & CH), 3.81-3.68 (m, 1H, $CH_2$), 3.69-3.57 (m, 1H, $CH_2$), 2.49 (d, J = 2.0 Hz, 1H, $CH_2$), 2.47 (d, J = 2.0 Hz, 1H, $CH_2$), 2.18 (dp, J = 13.5 & 7.0 Hz, 1H, CH), 0.98 (d, J = 6.5 Hz, 6H, 2×$CH_3$); **$^{13}$C NMR (101 MHz, CDCl$_3$)** δ 165.5, 165.2, 156.2, 136.1, 128.6, 128.3, 128.2, 98.0, 67.3, 43.3, 36.6, 26.1, 22.4. Data is consistent with literature.

**Methyl 2-((1-(*tert*-butoxy)-4-methyl-1-oxopentan-2-yl)carbamoyl)benzoate**

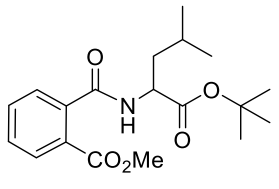

Monomethyl phthalate (1.80 g, 10.0 mmol), N,N'-diisopropylcarbondiimide (1.40 g, 10 mmol), 4-Dimethylaminopyridine (122 mg, 1 mmol) were dissolved in dry DCM (100 ml). The reaction mixture was stirred at room temperature for 30 minutes. Then H-Leu-OtBu.HCl (2.30 g, 10.0 mmol) and $NEt_3$ (1.4 mL, 10 mmol) were added in the mixture. The reaction mixture was stirred at room temperature overnight. The solvent was removed under reduced pressure and residue purified by flash column chromatography eluting with 20% EtOAc in petrol to give methyl 2-((1-(*tert*-butoxy)-4-methyl-1-oxopentan-2-yl)carbamoyl)benzoate (3.40 g, 97%) as a white solid.

**FTIR** $\nu_{max}$ (thin film/cm$^{-1}$ ) 3319, 2957, 1727, 1651; **$^1$H NMR (400 MHz, $CDCl_3$)** δ 7.88 (dd, J = 8.0, 1.5 Hz, 1H, $CH_{Ar}$), 7.61 – 7.42 (m, 3H, $CH_{Ar}$), 6.28 (d, J = 8.5 Hz, 1H, NH), 4.74 (dt, J = 8.5, 5.5 Hz, 1H, CH), 3.87 (s, 3H, $CH_3$), 1.88 – 1.59 (m, 3H, CH & $CH_2$), 1.51 (s, 9H, $C(CH_3)_3$), 1.04 (d, J = 6.5 Hz, 3H, $CH_3$), 1.01 (d, J = 6.5 Hz, 3H, $CH_3$); **$^{13}$C NMR (101 MHz, $CDCl_3$)** δ 172.1, 168.6, 167.2, 137.8, 131.8, 130.1, 129.8, 129.6, 127.6, 82.0, 52.5, 51.8, 42.0, 28.0, 25.0, 22.8, 22.2; **MS (ESI$^+$)** 372 (100%, M+Na$^+$); **HRMS (ESI-TOF)** m/z: $[M + Na]^+$ Calcd for $C_{19}H_{27}NO_5Na$ 372.1787; Found 372.1787.

**Methyl 2-(4-isobutyl-5-oxo-4,5-dihydrooxazol-2-yl)benzoate, 1m**

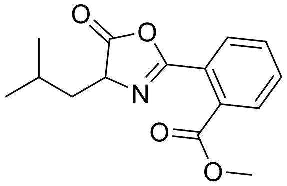


To a solution of methyl 2-((1-(*tert*-butoxy)-4-methyl-1-oxopentan-2-yl)carbamoyl)benzoate (3.4 g, 10 mmol) dissolved in dry DCM (20 mL) was added TFA (20 mL) and the reaction was stirred at room temperature overnight. The solvent was removed under reduced pressure. The residue and TFAA (4.5 mL, 13 mmol) were dissolved in dry DCM (30 mL) and the reaction was stirred at room temperature for 2 hours. The solvent was removed under reduced pressure and purified by flash column chromatography eluting with 20% $Et_2O$ in petrol to give methyl 2-(4-isobutyl-5-oxo-4,5-dihydrooxazol-2-yl)benzoate (**1m**) (1.6 g, 60%) as a yellow oil.

**FTIR** $\nu_{max}$ (thin film/cm$^{-1}$ ) 2957, 2872, 1824, 1731, 1657; **$^1$H NMR (400 MHz, $CDCl_3$)** δ 7.88 – 7.73 (m, 2H, $CH_{Ar}$), 7.66 – 7.57 (m, 2H, $CH_{Ar}$), 4.42 (dd, J = 9.5, 5.5 Hz, 1H, CH), 3.91 (s, 3H, $OCH_3$), 2.13-2.02 (m, 1H, CH), 1.86 (ddd, J = 13.5, 8.0, 5.5 Hz, 1H, $CH_2$), 1.72 (ddd, J = 13.5, 9.5, 6.0 Hz, 1H, $CH_2$), 1.06 (d, J = 6.5 Hz, 3H, $CH_3$), 1.04 (d, J = 6.5 Hz, 3H, $CH_3$); **$^{13}$C NMR (101 MHz, $CDCl_3$)** δ 178.6, 167.3, 161.7, 131.9, 131.7, 131.5, 129.8, 129.5, 129.4, 64.0, 52.7, 40.5, 25.3,

22.8, 21.9; **MS (ESI$^+$)** 276 (100%, M+H$^+$); **HRMS (ESI-TOF)** m/z: [M + H]$^+$ Calcd for $C_{15}H_{18}NO_4$ 276.1158; Found 276.1321.

**Methyl 2-((1-(*tert*-butoxy)-1-oxo-3-phenylpropan-2-yl)carbamoyl)benzoate**

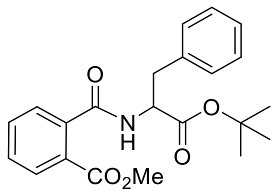


Monomethyl phthalate (1.80 g, 10.0 mmol), N,N'-diisopropylcarbondiimide (1.40 g, 10 mmol), 4-Dimethylaminopyridine (122 mg, 1 mmol) were dissolved in dry DCM (100 ml). The reaction mixture was stirred at room temperature for 30 minutes. Then H-Phe-OtBu.HCl (3.60 g, 10.0 mmol) and $NEt_3$ (1.4 mL, 10 mmol) were added in the mixture. The reaction mixture was stirred at room temperature overnight. The solvent was removed under reduced pressure and residue purified by flash column chromatography eluting with 20% EtOAc in petrol to give methyl 2-((1-(*tert*-butoxy)-1-oxo-3-phenylpropan-2-yl)carbamoyl)benzoate (3.60 g, 95%) as a yellow oil.

**FTIR** $\nu_{max}$ (thin film/cm$^{-1}$ ) 3317, 2978, 1724, 1651; **$^1$H NMR (400 MHz, $CDCl_3$)** δ 7.92 – 7.80 (m, 1H, $CH_{Ar}$), 7.54 – 7.43 (m, 2H, $CH_{Ar}$), 7.45 – 7.34 (m, 1H, $CH_{Ar}$), 7.34 – 7.19 (m, 5H, $CH_{Ar}$), 6.47 (d, J = 7.5 Hz, 1H, NH), 4.98 (dt, J = 7.5, 6.0 Hz, 1H, CH), 3.84 (s, 3H, $CH_3$), 3.29 – 3.18 (m, 2H, $CH_2$), 1.42 (s, 9H, $C(CH_3)_3$); **$^{13}$C NMR (101 MHz, $CDCl_3$)** δ 170.5, 168.4, 167.1, 137.6, 136.3, 131.8, 130.1, 129.8, 129.7, 129.6, 128.4, 127.5, 126.9, 82.5, 54.0, 52.5, 38.0, 28.0; **MS (ESI$^+$)** 406 (100%, M+Na$^+$); **HRMS (ESI-TOF)** m/z: [M + H]$^+$ Calcd for $C_{22}H_{26}NO_5$ 406.1630; Found 406.1624.

**Methyl 2-(4-benzyl-5-oxo-4,5-dihydrooxazol-2-yl)benzoate, 1n**

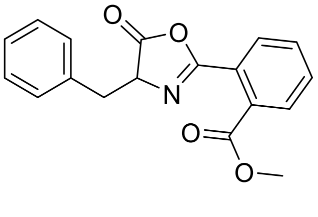


To a solution of GP-13-1 (3.6 g, 10 mmol) dissolved in dry DCM (20 mL) was added TFA (20 mL) and the reaction was stirred at room temperature overnight. The solvent was removed under reduced pressure. The residue and TFAA (4.5 mL, 13 mmol) was dissolved in dry DCM (30 mL)

and the reaction was stirred at room temperature for 2 hours. The solvent was removed under reduced pressure and purified by flash column chromatography eluting with 40% $Et_2O$ in petrol to give methyl 2-(4-benzyl-5-oxo-4,5-dihydrooxazol-2-yl)benzoate (**1n**) (1.4 g, 45%) as a yellow oil.

**FTIR** $\nu_{max}$ (thin film/cm$^{-1}$ ) 3031, 2952, 1817, 1726, 1655; **$^1$H NMR (400 MHz, $CDCl_3$)** δ 7.84 – 7.74 (m, 1H, $CH_{Ar}$), 7.61 – 7.49 (m, 3H, $CH_{Ar}$), 7.36 – 7.21 (m, 5H, $CH_{Ar}$), 4.70 (dd, J = 7.0, 5.0 Hz, 1H, CH), 3.80 (s, 3H, $CH_3$), 3.40 (dd, J = 14.0, 5.0 Hz, 1H, $CH_2$), 3.21 (dd, J = 14.0, 7.0 Hz, 1H, $CH_2$); **$^{13}$C NMR (101 MHz, $CDCl_3$)** δ 177.3, 167.1, 162.0, 135.5 (×2C), 131.7, 131.5, 129.9, 129.7, 129.5, 128.5, 127.2, 126.4, 66.5, 52.7, 37.0; **MS (ESI$^+$)** 310 (100%, M+H$^+$); **HRMS (ESI-TOF)** m/z: $[M + H]^+$ Calcd for $C_{18}H_{16}NO_4$ 310.1079; Found 310.1080.

**Methyl 2-((1-(*tert*-butoxy)-4-(methylthio)-1-oxobutan-2-yl)carbamoyl)benzoate**

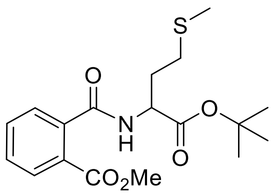


Monomethyl phthalate (1.80 g, 10.0 mmol), N,N'-diisopropylcarbondiimide (1.40 g, 10 mmol), 4-Dimethylaminopyridine (122 mg, 1 mmol) were dissolved in dry DCM (100 ml). The reaction mixture was stirred at room temperature for 30 minutes. Then H-Met-OtBu.HCl (2.40 g, 10.0 mmol) and $NEt_3$ (1.4 mL, 10 mmol) were added in the mixture. The reaction mixture was stirred at room temperature overnight. The solvent was removed under reduced pressure and residue purified by flash column chromatography eluting with 20% EtOAc in petrol to give methyl 2-((1-(*tert*-butoxy)-4-(methylthio)-1-oxobutan-2-yl)carbamoyl)benzoate (3.30 g, 90%) as a yellow oil.

**FTIR** $\nu_{max}$ (thin film/cm$^{-1}$ ) 3300, 2976, 1725, 1650; **$^1$H NMR (400 MHz, $CDCl_3$)** δ 7.87 – 7.80 (m, 1H, $CH_{Ar}$), 7.55 – 7.40 (m, 3H, $CH_{Ar}$), 6.63 (d, J = 7.5 Hz, 1H, NH), 4.76 (td, J = 7.0, 5.0 Hz, 1H, CH), 3.83 (s, 3H, $CH_3$), 2.68 – 2.51 (m, 2H, $CH_2$), 2.31 – 2.18 (m, 1H, $CH_2$), 2.10 (s, 3H, $CH_3$), 2.14 – 1.96 (m, 1H, $CH_2$), 1.47 (s, 9H, $C(CH_3)_3$); **$^{13}$C NMR (101 MHz, $CDCl_3$)** δ 170.9, 168.7, 166.9, 137.7, 131.9, 130.1, 129.8, 129.3, 127.5, 82.5, 52.6, 52.5, 32.1, 29.8, 28.0, 15.4; **MS (ESI$^+$)** 368 (100%, M+H$^+$); **HRMS (ESI-TOF)** m/z: $[M + H]^+$ Calcd for $C_{18}H_{26}NO_5S$ 368.1532; Found 368.1534.

**Methyl 2-(4-(2-(methylthio)ethyl)-5-oxo-4,5-dihydrooxazol-2-yl)benzoate, 1o**

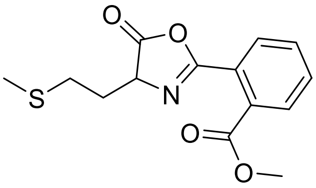

To a solution of 2-((1-(*tert*-butoxy)-4-(methylthio)-1-oxobutan-2-yl)carbamoyl)benzoate (3.4 g, 9 mmol) dissolved in dry DCM (20 mL) was added TFA (20 mL) and the reaction was stirred at room temperature overnight. The solvent was removed under reduced pressure. The residue and TFAA (4.5 mL, 13 mmol) were dissolved in dry DCM (30 mL) and the reaction was stirred at room temperature for 2 hours. The solvent was removed under reduced pressure and purified by flash column chromatography eluting with 40% $Et_2O$ in petrol to give methyl 2-(4-(2-(methylthio)ethyl)-5-oxo-4,5-dihydrooxazol-2-yl)benzoate (**1o**) (1.1 g, 40%) as a yellow oil.

**FTIR** $\nu_{max}$ (thin film/cm$^{-1}$ ) 3264, 2924, 1719, 1649; **$^1$H NMR (400 MHz, $CDCl_3$)** δ 7.89 – 7.81 (m, 1H, $CH_{Ar}$), 7.83 – 7.76 (m, 1H, $CH_{Ar}$), 7.69 – 7.57 (m, 2H, $CH_{Ar}$), 4.64 (dd, J = 7.5, 6.0 Hz, 1H, CH), 3.92 (s, 3H, $CH_3$), 2.77 (t, J = 7.0 Hz, 2H, $CH_2$), 2.42 – 2.26 (m, 1H, $CH_2$), 2.24 – 2.13 (m, 4H, $CH_2$, $CH_3$); **$^{13}$C NMR (101 MHz, $CDCl_3$)** δ 178.0, 162.5, 132.8, 131.8, 131.6, 129.8, 129.6, 126.4, 63.8, 52.8, 52.7, 30.3, 30.1, 15.2; **MS (ESI$^+$)** 294 (100%, M+H$^+$); **HRMS (ESI-TOF)** m/z: [M + H]$^+$ Calcd for $C_{14}H_{16}NO_4S$ 294.0800; Found 294.0806.

**Methyl 2-((6-(((benzyloxy)carbonyl)amino)-1-(tert-butoxy)-1-oxohexan-2-yl)-carbamoyl)-benzoate**

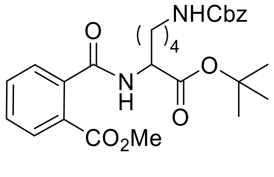

Monomethyl phthalate (1.80 g, 10.0 mmol), N,N'-diisopropylcarbondiimide (1.40 g, 10 mmol), 4-Dimethylaminopyridine (122 mg, 1 mmol) were dissolved in dry DCM (100 ml). The reaction mixture was stirred at room temperature for 30 minutes. Then H-Lys(Z)-OtBu.HCl (3.8 g, 10.0 mmol) and $NEt_3$ (1.4 mL, 10 mmol) were added in the mixture. The reaction mixture was stirred at room temperature overnight. The solvent was removed under reduced pressure and residue purified by flash column chromatography eluting with 40% EtOAc in petrol to give

methyl 2-((6-(((benzyloxy)carbonyl)amino)-1-(tert-butoxy)-1-oxohexan-2-yl)-carbamoyl)-benzoate (4.6 g, 92%) as a colorless oil.

**FTIR** ν*max* (thin film/cm$^{-1}$ ) 3318, 2949, 1722, 1650; **$^1$H NMR (400 MHz, CDCl$_3$)** δ 7.88 - 7.86 (m, 1H, $CH_{Ar}$), 7.57 – 7.42 (m, 3H, $CH_{Ar}$), 7.39 – 7.28 (m, 5H, $CH_{Ar}$), 6.53 (d, J = 8.0 Hz, 1H, NH), 5.28 – 5.23 (br, 1H, NH), 5.05 (d, J = 12.0 Hz, 1H, $CH_2$), 4.99 (d, J = 12.0 Hz, 1H, $CH_2$), 4.75 – 4.66 (m, 1H, $CH_2$), 3.83 – 3.78 (s, 3H, $CH_3$), 3.32 – 3.14 (m, 2H, $CH_2$), 2.09 – 1.96 (m, 1H, $CH_2$), 1.83 – 1.73 (m, 1H, $CH_2$), 1.68 – 1.52 (m, 3H, $CH_2$), 1.50 – 1.39 (m, 10H, $CH_2$, $C(CH_3)_3$); **$^{13}$C NMR (101 MHz, CDCl$_3$)** δ 171.5, 168.8, 167.0, 156.6, 138.1, 136.7, 132.0, 130.1, 129.7, 129.2, 128.5, 128.1, 128.0, 127.7, 82.3, 66.5, 52.9, 52.5, 40.5, 32.0, 29.3, 28.0, 22.0; **MS (ESI$^+$)** 499 (100%, M+H$^+$); **HRMS (ESI-TOF)** m/z: [M + H]$^+$ Calcd for $C_{27}H_{35}N_2O_7$ 499.2444; Found 499.2449.

**Methyl 2-(4-(4-(((benzyloxy)carbonyl)amino)butyl)-5-oxo-4,5-dihydrooxazol-2-yl)benzoate, 1p**

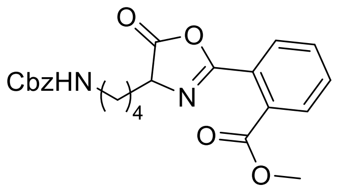


To a solution of methyl 2-((6-(((benzyloxy)carbonyl)amino)-1-(tert-butoxy)-1-oxohexan-2-yl)-carbamoyl)-benzoate (4.5 g, 9 mmol) dissolved in dry DCM (25 mL) was added TFA (25 mL) and the reaction was stirred at room temperature overnight. The solvent was removed under reduced pressure. The residue and TFAA (4.5 mL, 13 mmol) were dissolved in dry DCM (30 mL) and the reaction was stirred at room temperature for 2 hours. The solvent was removed under reduced pressure and purified by flash column chromatography eluting with 30% EtOAc in petrol to give methyl 2-(4-(4-(((benzyloxy)carbonyl)amino)butyl)-5-oxo-4,5-dihydrooxazol-2-yl)benzoate (**1p**) (2.2 g, 56%) as a yellow oil.

**FTIR** ν*max* (thin film/cm$^{-1}$ ) 3331, 2952, 1709, 1661; **$^1$H NMR (400 MHz, CDCl$_3$)** δ 7.88 – 7.81 (m, 1H, $CH_{Ar}$), 7.81 – 7.72 (m, 1H, $CH_{Ar}$), 7.66 – 7.57 (m, 2H, $CH_{Ar}$), 7.43 – 7.27 (m, 5H, $CH_{Ar}$), 5.09 – 4.96 (m, 3H, $CH_2$, NH), 4.44 – 4.36 (m, 1H, CH), 3.87 (s, 3H, $CH_3$), 3.26 – 3.20 (m, 2H, $CH_2$), 2.10 – 2.02 (m, 1H, $CH_2$), 1.97 – 1.81 (m, 1H, $CH_2$), 1.67 – 1.45 (m, 4H, 2$CH_2$); **$^{13}$C NMR (101 MHz, CDCl$_3$)** δ 178.0, 167.1, 162.2, 156.5, 136.6, 136.5, 131.8, 131.6, 129.9, 129.6, 128.5, 128.1, 128.1, 126.5, 66.6, 65.3, 52.8, 40.7, 30.9, 29.5, 22.7; **MS (ESI$^+$)** 425 (100%, M+H$^+$); **HRMS (ESI-TOF)** m/z: [M + H]$^+$ Calcd for $C_{23}H_{24}N_2O_6$ 425.1713; Found 425.1714.

**1-(tert-butyl) 4-methyl (2-(methoxycarbonyl)benzoyl)aspartate**

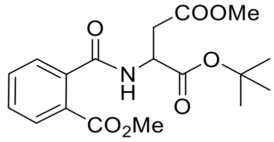


Monomethyl phthalate (1.80 g, 10.0 mmol), N,N'-diisopropylcarbondiimide (1.40 g, 10 mmol), 4-Dimethylaminopyridine (122 mg, 1 mmol) were dissolved in dry DCM (100 ml). The reaction mixture was stirred at room temperature for 30 minutes. Then (S)-1-tert-Butyl 4-methyl 2-aminosuccinate hydrochloride (2.4 g, 10.0 mmol) and $NEt_3$ (1.4 mL, 10 mmol) were added in the mixture. The reaction mixture was stirred at room temperature overnight. The solvent was removed under reduced pressure and residue purified by flash column chromatography eluting with 40% EtOAc in petrol to give 1-(tert-butyl) 4-methyl (2-(methoxycarbonyl)benzoyl)aspartate (3.1 g, 85%) as a colorless oil.

**FTIR** $\nu_{max}$ (thin film/$cm^{-1}$ )3346, 2981, 1725, 1655; **$^1$H NMR (400 MHz, $CDCl_3$)** δ 7.82 – 7.78 (m, 1H, $CH_{Ar}$), 7.49 – 7.39 (m, 3H, $CH_{Ar}$), 6.82 (d, J = 8.0 Hz, 1H, NH), 4.87- 4.82 (m, 1H, CH), 3.78 (s, 3H, $CH_3$), 3.63(s, 3H, $CH_3$), 3.05 - 2.95 (m, 2H, $CH_2$), 1.41 (s, 9H, $C(CH_3)_3$); **$^{13}$C NMR (101 MHz, $CDCl_3$)** δ 171.4, 169.4, 168.7, 166.7, 137.7, 132.0, 130.0, 129.7, 129.1, 127.5, 82.6, 52.3, 51.7, 49.5, 36.0, 27.8; **MS (ESI$^+$)** 366 (100%, M+H$^+$); **HRMS (ESI-TOF)** m/z: $[M + H]^+$ Calcd for $C_{18}H_{24}NO_7$ 366.1552; Found 366.1552.

**Methyl 2-(4-(2-methoxy-2-oxoethyl)-5-oxo-4,5-dihydrooxazol-2-yl)benzoate, 1q**

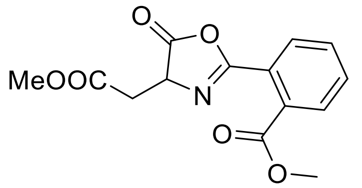


To a solution of 1-(tert-butyl) 4-methyl (2-(methoxycarbonyl)benzoyl)aspartate (2.9 g, 8 mmol) dissolved in dry DCM (20 mL) was added TFA (20 mL) and the reaction was stirred at room temperature overnight. The solvent was removed under reduced pressure. The residue and TFAA (4.5 mL, 13 mmol) were dissolved in dry DCM (30 mL) and the reaction was stirred at room temperature for 2 hours. The solvent was removed under reduced pressure and purified by flash column chromatography eluting with 40% EtOAc in petrol to give methyl methyl 2-(4-(2-methoxy-2-oxoethyl)-5-oxo-4,5-dihydrooxazol-2-yl)benzoate (**1q**) (1.5 g, 65%) as a yellow oil.

**FTIR** $\nu_{max}$ (thin film/cm$^{-1}$ ) 3004, 2954, 1822, 1727, 1653; **$^1$H NMR (400 MHz, $CDCl_3$)** δ 7.87 – 7.73 (m, 2H, $CH_{Ar}$), 7.63 – 7.54 (m, 2H, $CH_{Ar}$), 4.64 (d, J = 5.0 Hz, 0.5H, CH), 4.63 (d, J = 5.0 Hz, 0.5H, CH), 3.87 (s, 3H, $CH_3$), 3.70 (s, 3H, $CH_3$), 3.12 (dd, J = 17.0, 5.0 Hz, 1H, $CH_2$), 3.02 (dd, *J* = 17.0, 5.0 Hz, 1H, $CH_2$); **$^{13}$C NMR (101 MHz, $CDCl_3$)** δ 177.1, 169.7, 167.3, 163.2, 131.9, 131.8, 131.5, 129.9, 129.5, 126.3, 61.8, 52.7, 52.3, 34.8; **MS (ESI$^+$)** 292 (100%, M+H$^+$); **HRMS (ESI-TOF)** m/z: [M + H]$^+$ Calcd for $C_{14}H_{14}NO_6$ 292.0821; Found 292.0815.

**5-benzyl 1-(tert-butyl) (2-(methoxycarbonyl)benzoyl)glutamate**

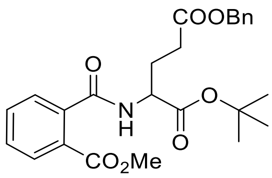


Monomethyl phthalate (1.80 g, 10.0 mmol), N,N'-diisopropylcarbondiimide (1.40 g, 10 mmol), 4-Dimethylaminopyridine (122 mg, 1 mmol) were dissolved in dry DCM (100 ml). The reaction mixture was stirred at room temperature for 30 minutes. Then H-Glu(OBn)-OtBu.HCl (4 g, 12.0 mmol) and $NEt_3$ (1.4 mL, 10 mmol) were added in the mixture. The reaction mixture was stirred at room temperature overnight. The solvent was removed under reduced pressure and residue purified by flash column chromatography eluting with 20% EtOAc in petrol to give methyl 5-benzyl 1-(tert-butyl) (2-(methoxycarbonyl)benzoyl)glutamate (4.0 g, 85%) as a colorless oil.

**FTIR** $\nu_{max}$ (thin film/cm$^{-1}$ ) 3345, 2977, 1725, 1651; **$^1$H NMR (400 MHz, $CDCl_3$)** δ 7.94 – 7.84 (m, 1H, $CH_{Ar}$), 7.60 – 7.45 (m, 3H, $CH_{Ar}$), 7.41 – 7.31 (m, 5H, $CH_{Ar}$), 6.54 (d, J = 8.0 Hz, 1H, NH), 5.18 – 5.06 (s, 2H, $CH_2$), 3.86 – 3.77 (s, 3H, $CH_3$), 2.70 – 2.48 (m, 2H, $CH_2$), 2.46 – 2.29 (m, 1H, $CH_2$), 2.18 – 2.01 (m, 1H, $CH_2$), 1.57 – 1.46 (s, 9H, $C(CH_3)_3$); **$^{13}$C NMR (101 MHz, $CDCl_3$)** δ 172.8, 170.9, 168.9, 167.0, 137.6, 135.8, 131.9, 130.1, 129.9, 129.4, 128.6, 128.3, 128.2, 127.5, 82.7, 66.5, 52.5, 52.5, 30.3, 28.0, 27.8; **MS (ESI$^+$)** 456 (100%, M+H$^+$); **HRMS (ESI-TOF)** m/z: [M + H]$^+$ Calcd for $C_{25}H_{31}NO_7$ 456.2022; Found 456.2020.

**Methyl 2-(4-(3-(benzyloxy)-3-oxopropyl)-5-oxo-4,5-dihydrooxazol-2-yl)benzoate, 1r**

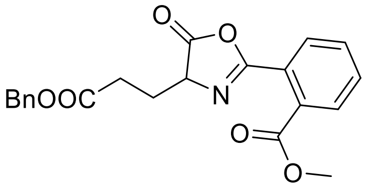


To a solution of 5-benzyl 1-(tert-butyl) (2-(methoxycarbonyl)benzoyl)glutamate (3.6 g, 8 mmol) dissolved in dry DCM (20 mL) was added TFA (20 mL) and the reaction was stirred at room temperature overnight. The solvent was removed under reduced pressure. The residue and TFAA (4.5 mL, 13 mmol) were dissolved in dry DCM (30 mL) and the reaction was stirred at room temperature for 2 hours. The solvent was removed under reduced pressure and purified by flash column chromatography eluting with 30% EtOAc in petrol to give methyl 2-(4-(3-(benzyloxy)-3-oxopropyl)-5-oxo-4,5-dihydrooxazol-2-yl)benzoate (**1r**) (1.8 g, 60%) as a yellow oil.

**FTIR** $\nu_{max}$ (thin film/cm$^{-1}$ ) 3036, 2952, 1821, 1726, 1657; **$^1$H NMR (400 MHz, $CDCl_3$)** δ 7.91 – 7.81 (m, 1H, $CH_{Ar}$), 7.81 – 7.74 (m, 1H, $CH_{Ar}$), 7.69 – 7.57 (m, 2H, $CH_{Ar}$), 7.46 – 7.29 (m, 5H, $CH_{Ar}$), 5.24 – 5.10 (s, 2H, $CH_2$), 4.57 (d, J = 6.0 Hz, 0.5H, CH), 4.53 – 4.45 (d, J = 6.0 Hz, 0.5H, CH), 3.97 – 3.81 (s, 3H, $CH_3$), 2.79 – 2.59 (m, 2H, $CH_2$), 2.50 – 2.35 (m, 1H, $CH_2$), 2.30 – 2.06 (m, 1H, $CH_2$); **$^{13}$C NMR (101 MHz, $CDCl_3$)** δ 177.5, 172.1, 167.1, 162.4, 135.7, 131.9, 131.8, 131.6, 129.8, 129.6, 128.6, 128.3, 128.3, 126.3, 66.6, 64.3, 52.7, 29.9, 26.4.; **MS (ESI$^+$)** 382 (100%, M+H$^+$); **HRMS (ESI-TOF)** m/z: [M + H]$^+$ Calcd for $C_{21}H_{20}NO_6$ 382.1291; Found 382.1298.

## Screening process

**Table 1. Chiral ligand screening and optimization.** [a] Concentration with respect to cyclic carbamate. [b] 2.0 equiv. base and 5 equiv. of $^t$BuOH were used. [c] Isolated yield after chromatography. [d] Determined by chiral HPLC with a constant flow rate of 1 mL/min using hexane/isopropyl alcohol 95:5 as the mobile phase.

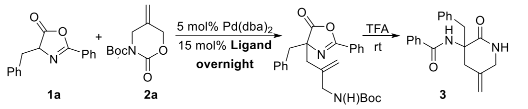


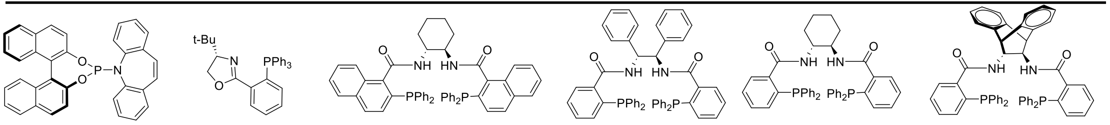


| Entry | Ligand | Conc.[a] (mol/L) | Additives[b] | Solvent | Temp(℃) | yield[c] | ee[d] |
|---|---|---|---|---|---|---|---|
| **1** | **1** | 0.05 | TEA, $^t$BuOH | DCM | rt | 98 | 8 |
| **2** | **2** | 0.05 | TEA, $^t$BuOH | DCM | rt | 95 | 14 |
| **3** | **3** | 0.05 | TEA, $^t$BuOH | DCM | rt | 93 | 25 |
| **4** | **3** | 0.05 | TEA, $^t$BuOH | DCM | 0 | 93 | 35 |
| **5** | **3** | 0.05 | TEA, $^t$BuOH | DCM | -20 | 83 | 25 |
| **6** | **3** | 0.05 | TEA, $^t$BuOH | Toluene | 0 | 10 | 50 |
| **7** | **3** | 0.05 | TEA, $^t$BuOH | Dioxane | rt | 96 | 53 |
| **8** | **3** | 0.05 | TEA, $^t$BuOH | Acetonitrile | rt | 95 | 10 |
| **9** | **4** | 0.05 | TEA, $^t$BuOH | Dioxane | rt | 95 | 50 |
| **10** | **5** | 0.05 | TEA, $^t$BuOH | Dioxane | rt | 65 | 63 |
| **11** | **6** | 0.05 | TEA, $^t$BuOH | Dioxane | rt | 30 | 81 |
| **12** | **6** | 0.05 | TEA, $^t$BuOH | 20% toluene in dioxane | 0 | 20 | 91 |
| **13** | **6** | 0.05 | DIPEA, $^t$BuOH | 20% toluene in dioxane | 0 | 50 | 91 |
| **14** | **6** | 0.1 | DIPEA, $^t$BuOH | 20% toluene in dioxane | 0 | 90 | 90 |
| **15** | **6** | 0.1 | - | 20% toluene in dioxane | 0 | No reaction | - |
| **16** | **6** | 0.1 | $^t$BuOH | 20% toluene in dioxane | 0 | No reaction | - |
| **17** | **6** | 0.1 | DIPEA | 20% toluene in dioxane | 0 | 70 | 90 |

**Allylation Products**

***tert*-butyl (*R*)-(2-((4-benzyl-5-oxo-2-phenyl-4,5-dihydrooxazol-4-yl)methyl)allyl)carbamate**

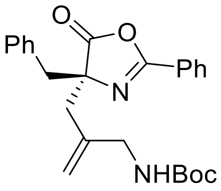


*N*-Boc-5-methylenecyclohexacarbamate (**2a**) (21 mg, 0.1 mmol), (*R,R*)-ANDEN-phenyl Trost ligand (6 mg, 0.0075 mmol), {η$^3$-$C_3H_5PdCl$}$_2$ (1 mg, 0.0025 mmol), $^t$BuOH (0.045 ml, 0.5 mmol) and DIPEA (0.025 ml, 0.2 mmol) were dissolved in dry 20% toluene in dioxane (1 mL) at 0 °C for 20 mins. Then 2-phenyl-4-benzyl-2-oxazolin-5-one (**1a**) (75 mg, 0.3 mmol) was added. The reaction mixture was stirred at 0 °C overnight. The solvent was removed under reduced pressure and residue purified by flash column chromatography eluting with 20% EtOAc in petrol to give *tert*-butyl (*R*)-(2-((4-benzyl-5-oxo-2-phenyl-4,5-dihydrooxazol-4-yl)methyl)allyl)carbamate (38 mg, 90%) as a colourless oil.

**FTIR** $\nu_{max}$ (thin film/cm$^{-1}$ ) 3381, 2978, 2928, 1816, 1712, 1651; **$^1$H NMR (400 MHz, CDCl$_3$)** δ 7.82-7.83 (m, 2H, $CH_{Ar}$), 7.51-7.54 (m, 1H, $CH_{Ar}$), 7.40-7.43 (m, 2H, $CH_{Ar}$), 7.12-7.16 (m, 5H, $CH_{Ar}$), 5.12 (s, 1H, NH), 5.07 (s, 1H, C=CH), 5.03 (s, 1H, C=CH), 3.72 (br, 2H, $CH_2$), 3.26 (d, J = 12.0 Hz, 1H, $CH_2$), 3.17 (d, J = 12.0 Hz, 1H, $CH_2$), 2.75 (s, 2H, $CH_2$), 1.43 (s, 9H, $C(CH_3)_3$); **$^{13}$C NMR (101 MHz, CDCl$_3$)** δ 179.3, 160.1, 155.8, 140.6, 134.1, 132.7, 130.2, 128.7, 128.2, 127.8, 127.3, 125.5, 116.3, 79.3, 75.3, 46.2, 43.5, 41.0, 28.4; **MS (ESI$^+$)** 421 (100%, M+H$^+$); **HRMS (ESI-TOF)** m/z: [M + H]$^+$ Calcd for $C_{25}H_{29}N_2O_4$ 421.2122; Found 421.2134; **HPLC** (Cellulose-2, hexane:$^i$PrOH 95:5, flow rate 1.0 mL/min, λ = 254 nm, 22 °C) $t_R$(major) = 9.090, $t_R$(minor) = 10.323, er = 95:5. $[\alpha]_D^{22} = -10$ (*c* 1.0, $CHCl_3$).

***tert*-butyl-(*R*)-(2-((4-isobutyl-5-oxo-2-phenyl-4,5-dihydrooxazol-4-yl)methyl)allyl)-carbamate**

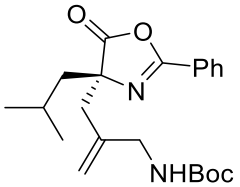

*N*-Boc-5-methylenecyclohexacarbamate (**2a**) (21 mg, 0.1 mmol), (*R,R*)-ANDEN-phenyl Trost ligand (6 mg, 0.0075 mmol), {η$^3$-$C_3H_5PdCl$}$_2$ (1 mg, 0.0025 mmol), $^t$BuOH (0.045 ml, 0.5 mmol) and DIPEA (0.025 ml, 0.2 mmol) were dissolved in dry 20% toluene in dioxane (1 mL) at 0 °C for 20 mins. Then 2-phenyl-4-isobutyl-2-oxazolin-5-one (**1b**) (65 mg, 0.3 mmol) was added. The reaction mixture was stirred at 0 °C overnight. The solvent was removed under reduced pressure and residue purified by flash column chromatography eluting with 20% EtOAc in petrol to give *tert*-butyl *tert*-butyl-(*R*)-(2-((4-isobutyl-5-oxo-2-phenyl-4,5-dihydrooxazol-4-yl)methyl)allyl)-carbamate (32 mg, 83%) as a white solid.

**FTIR** $\nu_{max}$ (thin film/cm$^{-1}$) 3346, 2960, 1815, 1713, 1651; **$^1$H NMR (400 MHz, CDCl$_3$)** δ 8.02 (d, *J* = 7.5 Hz, 2H, $CH_{Ar}$), 7.64 – 7.57 (m, 1H, $CH_{Ar}$), 7.51 (t, J = 7.5 Hz, 2H, $CH_{Ar}$), 5.04 (s, 1H, C=$CH_2$), 4.98 (br, 2H, NH, C=$CH_2$), 3.67 (br, 2H, $CH_2$), 2.70 – 2.55 (m, 2H, $CH_2$), 1.97 (dd, J = 14.0, 5.5 Hz, 1H, $CH_2$), 1.83 (dd, J = 14.0, 7.0 Hz, 1H, $CH_2$), 1.67 – 1.55 (m, 1H, CH), 1.43 (s, 9H, $C(CH_3)_3$), 0.90 (d, J = 6.5 Hz, 3H, $CH_3$), 0.88 (d, J = 6.5 Hz, 3H, $CH_3$); **$^{13}$C NMR (101 MHz, CDCl$_3$)** δ 180.6, 159.9, 156.8, 140.4, 132.8, 128.9, 127.9, 125.7, 116.0, 79.2, 73.9, 46.2, 46.1, 42.3, 28.4, 24.9, 24.1, 23.2; **MS (ESI$^+$)** 387 (100%, M+H$^+$); **HRMS (ESI-TOF)** m/z: $[M + H]^+$ Calcd for $C_{22}H_{31}N_2O_4$ 387.2278; Found 387.2276; **HPLC** (Cellulose-1, hexane:$^i$PrOH 99:1, flow rate 1.0 mL/min, λ = 254 nm, 22 °C) $t_R$(major) = 12.643, $t_R$(minor) = 10.993, er = 8.5:91.5.

***tert*-butyl-(*S*)-(2-((4-(2-(methylthio)ethyl)-5-oxo-2-phenyl-4,5-dihydrooxazol-4-yl)methyl)allyl)carbamate**

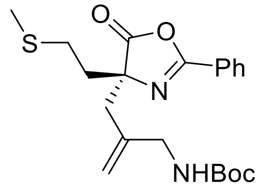


*N*-Boc-5-methylenecyclohexacarbamate (**2a**) (21 mg, 0.1 mmol), (*R,R*)-ANDEN-phenyl Trost ligand (6 mg, 0.0075 mmol), {η$^3$-$C_3H_5PdCl$}$_2$ (1 mg, 0.0025 mmol), $^t$BuOH (0.045 ml, 0.5 mmol) and DIPEA (0.025 ml, 0.2 mmol) were dissolved in dry 20% toluene in dioxane (1 mL) at 0 °C for 20 mins. Then 2-phenyl-4-[2-(methylthio)ethyl)]-2-oxazolin-5-one (**1c**) (71 mg, 0.3 mmol) was added. The reaction mixture was stirred at 0 °C overnight. The solvent was removed under reduced pressure and residue purified by flash column chromatography eluting with 15% EtOAc in petrol to give *tert*-butyl-(*S*)-(2-((4-(2-(methylthio)ethyl)-5-oxo-2-phenyl-4,5-dihydrooxazol-4-yl)methyl)allyl)carbamate (35 mg, 87%) as a colourless oil.

**FTIR** $\nu_{max}$ (thin film/cm$^{-1}$) 3355, 2976, 2920, 1815, 1713, 1650; **$^{1}$H NMR (400 MHz, CDCl$_3$)** δ 8.00 (d, 2H, J = 7.5 Hz, $CH_{Ar}$), 7.58 (t, 1H, J = 7.5 Hz, $CH_{Ar}$), 7.49 (t, 2H, J = 7.5 Hz, $CH_{Ar}$), 5.05 (s, 1H, C=$CH_2$), 5.01 (s, 1H, NH), 4.99 (s, 1H, C=$CH_2$), 3.67 (br, 2H, $CH_2$), 2.65 (d, J = 14.0 Hz, 1H), 2.60 (d, J = 14.0 Hz, 1H), 2.50-2.40 (m, 1H, $CH_2$), 2.39-2.29 (m, 1H, $CH_2$), 2.27-2.17 (m, 2H, $CH_2$), 2.04 (s, 3H, $CH_3$), 1.41 (s, 9H, $C(CH_3)_3$); **$^{13}$C NMR (101 MHz, CDCl$_3$)** δ. 179.8, 160.8, 156.8, 140.3, 133.0, 128.9, 128.0, 125.5, 116.3, 79.3, 73.2, 46.1, 41.4, 36.2, 28.6, 28.4, 15.2; **MS (ESI$^+$)** 404 (100%, M+H$^+$); **HRMS (ESI-TOF)** m/z: [M + H]$^+$ Calcd for $C_{21}H_{29}N_2O_4S$ 405.1848; **HPLC** (Cellulose-2, hexane:$^i$PrOH 97.5:2.5, flow rate 1.0 mL/min, λ = 254 nm, 22 °C) $t_R$(major) = 18.903, $t_R$(minor) = 11.037, er = 90:10.

***tert*-butyl-(*R*)-(2-((4-butyl-5-oxo-2-phenyl-4,5-dihydrooxazol-4-yl)methyl)allyl)carbamate**

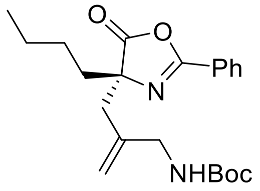


*N*-Boc-5-methylenecyclohexacarbamate (**2a**) (21 mg, 0.1 mmol), (*R*,*R*)-ANDEN-phenyl Trost ligand (6 mg, 0.0075 mmol), {η$^3$-$C_3H_5PdCl$}$_2$ (1 mg, 0.0025 mmol), $^t$BuOH (0.045 ml, 0.5 mmol) and DIPEA (0.025 ml, 0.2 mmol) were dissolved in dry 20% toluene in dioxane (1 mL) at 0 °C for 20 mins. Then 2-phenyl-4-butyl-2-oxazolin-5-one (**1d**) (65 mg, 0.3 mmol) was added. The reaction mixture was stirred at 0 °C overnight. The solvent was removed under reduced pressure and residue purified by flash column chromatography eluting with 10% EtOAc in petrol to give *tert*-butyl-(*R*)-(2-((4-butyl-5-oxo-2-phenyl-4,5-dihydrooxazol-4-yl)methyl)allyl)carbamate (33 mg, 85%) as a colourless oil.

**FTIR** $\nu_{max}$ (thin film/cm$^{-1}$) 3346, 2960, 2930, 1817, 1717, 1654; **$^{1}$H NMR (400 MHz, CDCl$_3$)** δ 8.01 (d, J = 7.5 Hz, 2H, $CH_{Ar}$), 7.59 (t, J = 7.5 Hz, 1H, $CH_{Ar}$), 7.50 (t, J = 7.5 Hz, 2H, $CH_{Ar}$), 5.04 (br, 2H, C=$CH_2$, NH), 4.99 (s, 1H, C=$CH_2$), 3.69 (br, 2H, $CH_2$), 2.64 (s, 2H, $CH_2$), 1.97 – 1.84 (m, 2H, $CH_2$), 1.97 – 1.84 (m, 9H, $C(CH_3)_3$), 1.35 – 1.06 (m, 4H, $CH_2CH_2$), 0.86 (t, J = 7.0 Hz, 3H, $CH_3$); **$^{13}$C NMR (101 MHz, CDCl$_3$)** δ 180.1, 160.2, 155.8, 140.7, 132.8, 128.9, 128.0, 125.6, 115.8, 79.2, 74.3, 46.2, 41.1, 37.2, 28.4, 25.8, 22.5, 13.8; **MS (ESI$^+$)** 387 (100%, M+H$^+$); **HRMS (ESI-TOF)** m/z: [M + H]$^+$ Calcd for $C_{22}H_{31}N_2O_4$ 387.2278; Found 387.2297; **HPLC** (Cellulose-1, hexane:$^i$PrOH 99:1, flow rate 1.0 mL/min, λ = 254 nm, 22 °C) $t_R$(major) = 13.663, $t_R$(minor) = 11.960, er = 6:94.

**_tert_-butyl (_S_)-(2-((4-((1_H_-indol-2-yl)methyl)-5-oxo-2-phenyl-4,5-dihydrooxazol-4-yl)methyl)allyl)carbamate**

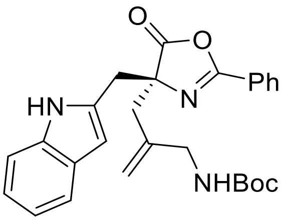


*N*-Boc-5-methylenecyclohexacarbamate (**2a**) (21 mg, 0.1 mmol), (*R*,*R*)-ANDEN-phenyl Trost ligand (6 mg, 0.0075 mmol), {η$^3$-$C_3H_5PdCl$}$_2$ (1 mg, 0.0025 mmol), $^t$BuOH (0.045 ml, 0.5 mmol) and DIPEA (0.025 ml, 0.2 mmol) were dissolved in dry 20% toluene in dioxane (1 mL) at 0 °C for 20 mins. Then 2-phenyl-4-((1*H*-indol-2-yl)methyl)-2-oxazolin-5-one (**1e**) (87 mg, 0.3 mmol) was added. The reaction mixture was stirred at 0 °C overnight. The solvent was removed under reduced pressure and residue purified by flash column chromatography eluting with 20% EtOAc in petrol to give *tert*-butyl (*S*)-(2-((4-((1*H*-indol-2-yl)methyl)-5-oxo-2-phenyl-4,5-dihydrooxazol-4-yl)methyl)allyl)carbamate (39 mg, 85%) as a colourless oil.

**FTIR** $\nu_{max}$ (thin film/cm$^{-1}$) 3319, 2978, 2921, 1814, 1693, 1653; **$^1$H NMR (400 MHz, $CDCl_3$)** δ 8.11 (br, 1H, PhNH), 7.84 – 7.76 (m, 2H, $CH_{Ar}$), 7.74 – 7.67 (m, 1H, $CH_{Ar}$), 7.53 – 7.46 (m, 1H, $CH_{Ar}$), 7.38 (t, *J* = 7.5 Hz, 2H, $CH_{Ar}$), 7.26 – 7.20 (m, 1H, $CH_{Ar}$), 7.13 – 7.04 (m, 2H, $CH_{Ar}$), 7.02 (d, *J* = 2.5 Hz, 1H, $CH_{Ar}$), 5.24 (br, 1H, NH), 5.09 (s, 1H, C=$CH_2$), 5.07 (s, 1H, C=$CH_2$), 3.84 – 3.66 (m, 2H, $CH_2$), 3.42 (d, J = 14.0 Hz, 1H, $CH_2$), 3.37 (d, *J* = 14.0 Hz, 1H, $CH_2$), 2.90 – 2.77 (m, 2H, $CH_2$), 1.47 (s, 9H, $C(CH_3)_3$); **$^{13}$C NMR (101 MHz, $CDCl_3$)** δ 179.8, 160.3, 155.9, 140.8, 135.7, 132.5, 128.6, 127.9, 127.6, 125.5, 123.8, 121.9, 119.6, 119.5, 116.1, 110.9, 108.6, 79.3, 75.8, 46.3, 40.8, 33.4, 28.4; **MS (ESI$^+$)** 460 (100%, M+H$^+$); **HRMS (ESI-TOF)** m/z: [M + H]$^+$ Calcd for $C_{27}H_{30}N_3O_4$ 460.2231; Found 460.2238; **HPLC** (Cellulose-2, hexane:$^i$PrOH 90:10, flow rate 1.0 mL/min, λ = 254 nm, 22 °C) $t_R$(major) = 15.463, $t_R$(minor) = 23.853, er = 91.5:8.5.

**_tert_-butyl (_R_)-(2-((4-(4-(((benzyloxy)carbonyl)amino)butyl)-5-oxo-2-phenyl-4,5-dihydrooxazol-4-yl)methyl)allyl)carbamate**

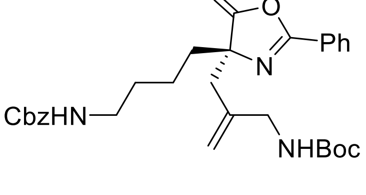


*N*-Boc-5-methylenecyclohexacarbamate (**2a**) (21 mg, 0.1 mmol), (*R*,*R*)-ANDEN-phenyl Trost ligand (6 mg, 0.0075 mmol), {η$^3$-$C_3H_5PdCl$}$_2$ (1 mg, 0.0025 mmol), $^t$BuOH (0.045 ml, 0.5 mmol)

and DIPEA (0.025 ml, 2 eq, 0.2 mmol) were dissolved in dry 20% toluene in dioxane (1 mL) at 0 °C for 20 mins. Then benzyl (4-(5-oxo-2-phenyl-4,5-dihydrooxazol-4-yl)butyl)carbamate (**1f**) (110 mg, 0.3 mmol) was added. The reaction mixture was stirred at 0 °C overnight. The solvent was removed under reduced pressure and residue purified by flash column chromatography eluting with 20% EtOAc in petrol to give *tert*-butyl (*R*)-(2-((4-(4-(((benzyloxy)carbonyl)amino)butyl)-5-oxo-2-phenyl-4,5-dihydrooxazol-4-yl)methyl)allyl)-carbamate (40 mg, 75%) as a colourless oil.

**FTIR** $\nu_{max}$ (thin film/cm$^{-1}$) 3334, 2929, 1816, 1695, 1654; **$^1$H NMR (400 MHz, CDCl$_3$)** δ 8.01 (d, J = 7.5 Hz, 2H, $CH_{Ar}$), 7.61 (t, J = 7.5 Hz, 1H, $CH_{Ar}$), 7.51 (t, J = 7.5 Hz, 2H, $CH_{Ar}$), 7.40 – 7.29 (m, 5H, $CH_{Ar}$), 5.07 (s, 2H, C=$CH_2$, NH), 5.05 (s, 1H, C=$CH_2$), 4.99 (br, 2H, $CH_2$), 4.81 (br, 1H, NH), 3.80 – 3.59 (m, 2H, $CH_2$), 3.17 (d, J = 7.0 Hz, 1H), 3.13 (d, J = 7.0 Hz, 1H), 2.67 – 2.57 (m, 2H, $CH_2$), 1.99 – 1.87 (m, 2H, $CH_2$), 1.62 – 1.47 (m, 2H, $CH_2$), 1.44 (s, 9H, $C(CH_3)_3$), 1.36 – 1.11 (m, 2H, $CH_2$); **$^{13}$C NMR (101 MHz, CDCl$_3$)** δ 179.9, 160.4, 156.3, 155.8, 140.5, 136.5, 132.9, 128.9, 128.5, 128.1, 128.0, 127.2, 125.5, 115.8, 79.3, 74.1, 66.6, 46.2, 41.1, 40.6, 36.8, 29.6, 28.4, 21.0; **MS (ESI$^+$)** 536 (100%, M+H$^+$); **HRMS (ESI-TOF)** m/z: $[M + H]^+$ Calcd for $C_{30}H_{38}N_3O_6$ 536.2755; Found 536.2768; **HPLC** (Cellulose-2, hexane:$^i$PrOH 80:20, flow rate 1.0 mL/min, λ = 254 nm, 22 °C) $t_R$(major) = 18.570, $t_R$(minor) = 14.300, er = 10:90.

**_tert_-butyl (_R_)-(2-((4-(4-hydroxybenzyl)-5-oxo-2-phenyl-4,5-dihydrooxazol-4-yl)methyl)-allyl)carbamate**

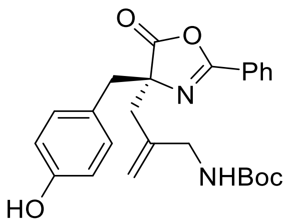


*N*-Boc-5-methylenecyclohexacarbamate (**2a**) (21 mg, 0.1 mmol), (*R*,*R*)-ANDEN-phenyl Trost ligand (6 mg, 0.0075 mmol), {η$^3$-$C_3H_5PdCl$}$_2$ (1 mg, 0.025 eq, 0.0025 mmol), $^t$BuOH (0.045 ml, 0.5 mmol) and DIPEA (0.025 ml, 0.2 mmol) were dissolved in dry 20% toluene in dioxane (1 mL) at 0 °C for 20 mins. Then 2-phenyl-4-(4-hydroxybenzyl)-2-oxazolin-5-one (**1g**) (80 mg, 0.3 mmol) was added. The reaction mixture was stirred at 0 °C overnight. The solvent was removed under reduced pressure and residue purified by flash column chromatography eluting with 20% EtOAc in petrol to give *tert*-butyl (*R*)-(2-((4-(4-hydroxybenzyl)-5-oxo-2-phenyl-4,5-dihydrooxazol-4-yl)methyl)-allyl)carbamate (31 mg, 70%) as a colourless oil.

**FTIR** $\nu_{max}$ (thin film/cm$^{-1}$) 3332, 2978, 1816, 1689, 1653; **$^1$H NMR (400 MHz, CDCl$_3$)** δ 7.92 – 7.79 (m, 2H, CH$_{Ar}$), 7.58 – 7.49 (m, 1H, CH$_{Ar}$), 7.46 – 7.38 (m, 2H, CH$_{Ar}$), 7.00 (d, J = 8.5 Hz, 2H, CH$_{Ar}$), 6.64 (d, J = 8.5 Hz, 2H, CH$_{Ar}$), 6.17 (s, 1H, OH), 5.17 (s, 1H, NH), 5.05 (s, 1H, C=CH$_2$), 5.02 (s, 1H, C=CH$_2$), 3.84 – 3.56 (m, 2H, CH$_2$), 3.16 (d, J = 13.5 Hz, CH$_2$), 3.07 (d, J = 13.5 Hz, 1H, CH$_2$), 2.73 (s, 2H, CH$_2$), 1.44 (s, 9H, C(CH$_3$)$_3$); **$^{13}$C NMR (101 MHz, CDCl$_3$)** δ 179.4, 160.2, 156.1, 155.3, 140.4, 132.7, 131.3, 128.8, 127.9, 125.6, 125.4, 116.3, 115.2, 79.1, 75.5, 46.2, 42.7, 40.8, 28.4; **MS (ESI$^+$)** 437 (100%, M+H$^+$); **HRMS (ESI-TOF)** m/z: [M + H]$^+$ Calcd for $C_{25}H_{29}N_2O_5$ 437.2071; Found 437.2088; **HPLC** (Cellulose-2, hexane:$^i$PrOH 95:5, flow rate 1.0 mL/min, λ = 254 nm, 22 °C) t$_R$(major) = 49.013, t$_R$(minor) = 44.447, er = 8.5:91.5.

**Methyl (*R*)-3-(4-(2-(((*tert*-butoxycarbonyl)amino)methyl)allyl)-5-oxo-2-phenyl-4,5-dihydrooxazol-4-yl)propanoate**

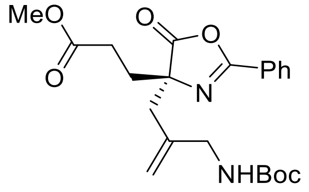


*N*-Boc-5-methylenecyclohexacarbamate (**2a**) (21 mg, 0.1 mmol), (*R*,*R*)-ANDEN-phenyl Trost ligand (6 mg, 0.0075 mmol), {η$^3$-C$_3$H$_5$PdCl}$_2$ (1 mg, 0.0025 mmol), $^t$BuOH (0.045 ml, 0.5 mmol) and DIPEA (0.025 ml, 0.2 mmol) were dissolved in dry 20% toluene in dioxane (1 mL) at 0 °C for 20 mins. Then 2-phenyl-4-methyl propanoate-2-oxazolin-5-one (**1h**) (74 mg, 0.3 mmol) was added. The reaction mixture was stirred at 0 °C overnight. The solvent was removed under reduced pressure and residue purified by flash column chromatography eluting with 20% EtOAc in petrol to give methyl (*R*)-3-(4-(2-(((*tert*-butoxycarbonyl)amino)-methyl)allyl)-5-oxo-2-phenyl-4,5-dihydrooxazol-4-yl)propanoate (33 mg, 80%) as a colourless oil.

**FTIR** $\nu_{max}$ (thin film/cm$^{-1}$) 3384, 2977, 1817, 1738, 1714, 1654; **$^1$H NMR (400 MHz, CDCl$_3$)** δ 8.03 – 7.96 (m, 2H, CH$_{Ar}$), 7.64 – 7.56 (m, 1H, CH$_{Ar}$), 7.56 – 7.45 (m, 2H, CH$_{Ar}$), 5.07 (s, 1H, C=CH$_2$), 5.01 (s, 1H, C=CH$_2$), 4.96 (s, 1H, NH), 3.74 – 3.65 (m, 2H, CH$_2$), 3.62 (s, 3H, CH$_3$), 2.66 (s, 2H CH$_2$), 2.38 – 2.20 (m, 4H, CH$_2$CH$_2$), 1.43 (s, 9H, C(CH$_3$)$_3$); **$^{13}$C NMR (101 MHz, CDCl$_3$)** δ 179.4, 172.4, 160.7, 156.8, 140.3, 133.1, 128.9, 128.0, 125.3, 116.1, 79.3, 73.2, 51.8, 46.1, 40.8, 32.1, 28.8, 28.4; **MS (ESI$^+$)** 417 (100%, M+H$^+$); **HRMS (ESI-TOF)** m/z: [M + H]$^+$ Calcd for $C_{22}H_{29}N_2O_6$ 417.2026; Found 417.2024; **HPLC** (Cellulose-1, hexane:$^i$PrOH 90:10, flow rate 1.0 mL/min, λ = 254 nm, 22 °C) t$_R$(major) = 16.587, t$_R$(minor) = 14.743, er = 12:88.

**tert-butyl (S)-(2-((5-oxo-2,4-diphenyl-4,5-dihydrooxazol-4-yl)methyl)allyl)carbamate**

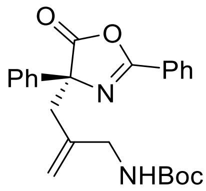


*N*-Boc-5-methylenecyclohexacarbamate (**2a**) (21 mg, 0.1 mmol), (*R*,*R*)-ANDEN-phenyl Trost ligand (6 mg, 0.0075 mmol), {$\eta^3$-$C_3H_5PdCl$}$_2$ (1 mg, 0.0025 mmol), $^t$BuOH (0.045 ml, 0.5 mmol) and DIPEA (0.025 ml, 0.2 mmol) were dissolved in dry 20% toluene in dioxane (1 mL) at 0 °C for 20 mins. Then 2-Phenyl-4-phenyl-2-oxazolin-5-one (**1i**) (72 mg, 0.3 mmol) was added. The reaction mixture was stirred at 0 °C overnight. The solvent was removed under reduced pressure and residue purified by flash column chromatography eluting with 20% EtOAc in petrol to give tert-butyl (S)-(2-((5-oxo-2,4-diphenyl-4,5-dihydrooxazol-4-yl)methyl)allyl)-carbamate (16 mg, 40%) as a colourless oil.

**FTIR** $\nu_{max}$ (thin film/cm$^{-1}$) 3350, 2971, 1816, 1717, 1654; **$^1$H NMR (400 MHz, CDCl$_3$)** δ 8.15 – 8.07 (m, 2H, $CH_{Ar}$), 7.77 – 7.66 (m, 2H, $CH_{Ar}$), 7.66 – 7.59 (m, 1H, $CH_{Ar}$), 7.59 – 7.49 (m, 2H, $CH_{Ar}$), 7.47 – 7.32 (m, 3H, $CH_{Ar}$), 5.05 (br, 2H, C=$CH_2$, NH), 5.00 – 4.96 (s, 1H, C=$CH_2$), 3.72 (br, 2H, $CH_2$), 3.09 – 2.98 (d, J = 14.0Hz, 1H, $CH_2$), 2.98 – 2.89 (d, J = 14.0 Hz, 1H, $CH_2$), 1.51 – 1.36 (s, 9H, 3$CH_3$); **$^{13}$C NMR (101 MHz, CDCl$_3$)** δ 178.3, 160.5, 155.8, 140.3, 137.8, 133.1, 128.9, 128.7, 128.4, 128.1, 125.7, 125.6, 116.3, 79.3, 75.0, 46.3, 44.3, 28.4; **MS (ESI$^+$)** 407 (100%, M+H$^+$); **HRMS (ESI-TOF)** m/z: [M + H]$^+$ Calcd for $C_{22}H_{29}N_2O_6$ 407.1965; Found 407.1983; **HPLC** (Cellulose-2, hexane:$^i$PrOH 99:1, flow rate 1.0 mL/min, λ = 254 nm, 22 °C) $t_R$(major) = 28.593, $t_R$(minor) = 32.263, er = 68:32.

***tert*-butyl (*R*)-(2-((4-benzyl-2-((((benzyloxy)carbonyl)amino)methyl)-5-oxo-4,5-dihydrooxazol-4-yl)methyl)allyl)carbamate**

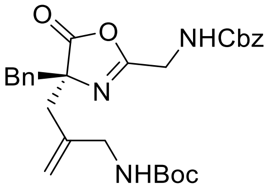


*N*-Boc-5-methylenecyclohexacarbamate (**2a**) (21 mg, 0.1 mmol), (*R*,*R*)-ANDEN-phenyl Trost ligand (6 mg, 0.0075 mmol), {$\eta^3$-$C_3H_5PdCl$}$_2$ (1 mg, 0.0025 mmol), $^t$BuOH (0.045 ml, 0.5 mmol) and DIPEA (0.025 ml, 0.2 mmol) were dissolved in dry 20% toluene in dioxane (1 mL) at 0 °C for 20 mins. Then benzyl (4-benzyl-5-oxo-4,5-dihydro-oxazol-2-ylmethyl)-carbamic acid (**1j**)

(102 mg, 3 eq, 0.3 mmol) was added. The reaction mixture was stirred at 0 °C overnight. The solvent was removed under reduced pressure and residue purified by flash column chromatography eluting with 30% EtOAc in petrol to give *tert*-butyl (*R*)-(2-((4-benzyl-2-((((benzyloxy)carbonyl)amino)methyl)-5-oxo-4,5-dihydrooxazol-4-yl)methyl)allyl)-carbamate (45 mg, 90%) as a colorless oil.

**FTIR** $\nu_{max}$ (thin film/cm$^{-1}$) 3332, 2978, 1821, 1693, 1516; **$^1$H NMR (400 MHz, $CDCl_3$)** δ 7.44 – 7.30 (m, 5H, $CH_{Ar}$), 7.30 – 7.21 (m, 3H, $CH_{Ar}$), 7.17 – 7.07 (m, 2H, $CH_{Ar}$), 6.30 (s, 1H, NH), 5.24 – 5.09 (m, 2H, $CH_2$), 5.09 – 4.98 (m, 1H, NH), 4.94 (s, 1H, C=$CH_2$), 4.93 (s, 1H, C=$CH_2$), 4.08 – 3.86 (m, 3H, $CH_2$), 3.34 (dd, J = 17.5 & 4.0 Hz, 1H, $CH_2$), 2.79 (d, J = 13.5 Hz, 1H, $CH_2$), 3.10 (s, 2H, $CH_2$), 2.43 (d, J = 13.5 Hz, 1H, $CH_2$), 1.39 (s, 9H, $C(CH_3)_3$); **$^{13}$C NMR (101 MHz, $CDCl_3$)** δ 178.7, 161.5, 156.6, 155.9, 140.9, 136.5, 133.7, 130.2, 128.4, 128.3, 128.13, 128.08, 127.5, 112.7, 79.7, 75.3, 67.0, 45.6, 43.1, 40.3, 38.7, 28.3; **MS (ESI$^+$)** 508 (100%, M+H$^+$); **HRMS (ESI-TOF)** m/z: [M + H]$^+$ Calcd for $C_{28}H_{34}N_3O_6$ 530.2262; Found 530.2284; **HPLC** (Cellulose-1, hexane:$^i$PrOH 90:10, flow rate 1.0 mL/min, λ = 254 nm, 22 °C) $t_R$(major) = 15.243, $t_R$(minor) = 17.807, er = 98.5:1.5; $[\alpha]_D^{22} = -10$ (*c* 1.0, $CHCl_3$).

**_tert_-butyl (_R_)-(2-((4-benzyl-2-(((tert-butoxycarbonyl)amino)methyl)-5-oxo-4,5-dihydrooxazol-4-yl)methyl)allyl)carbamate**

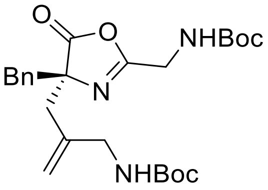


*N*-Boc-5-methylenecyclohexacarbamate (**2a**) (21 mg, 0.1 mmol), (*R*,*R*)-ANDEN-phenyl Trost ligand (6 mg, 0.0075 mmol), {η$^3$-$C_3H_5PdCl$}$_2$ (1 mg, 0.0025 mmol), $^t$BuOH (0.045 ml, 0.5 mmol) and DIPEA (0.025 ml, 0.2 mmol) were dissolved in dry 20% toluene in dioxane (1 mL) at 0 °C for 20 mins. Then *tert*-butyl (4-benzyl-5-oxo-4,5-dihydro-oxazol-2-ylmethyl)-carbamic acid (**1k**) (91 mg, 0.3 mmol) was added. The reaction mixture was stirred at 0 °C overnight. The solvent was removed under reduced pressure and residue purified by flash column chromatography eluting with 60% $Et_2O$ in petrol to give *tert*-butyl (*R*)-(2-((4-benzyl-2-(((*tert*-butoxycarbonyl)amino)methyl)-5-oxo-4,5-dihydrooxazol-4-yl)methyl)allyl)-carbamate (44 mg, 93%) as a colorless oil.

**FTIR** $\nu_{max}$ (thin film/cm$^{-1}$) 3347, 2978, 2928, 1823, 1693, 1516; **$^1$H NMR (400 MHz, $CDCl_3$)** δ 7.29 – 7.19 (m, 3H, $CH_{Ar}$), 7.17 – 7.08 (m, 2H, $CH_{Ar}$), 5.73 (s, 1H, NH), 5.21 (s, 1H, NH), 4.96 (s,

1H, $C=CH_2$), 4.92 (s, 1H, $C=CH_2$), 4.08 – 3.86 (m, 3H, $CH_2$), 3.50 – 3.37 (m, 1H, $CH_2$), 3.09 (s, 2H), 2.77 (d, J = 13.4 Hz, 1H, $CH_2$), 2.44 (d, J = 13.4 Hz, 1H, $CH_2$), 1.46 (s, 9H, $C(CH_3)_3$), 1.44 (s, 9H, $C(CH_3)_3$); **$^{13}$C NMR (101 MHz, $CDCl_3$)** δ 178.8, 161.6, 156.0, 155.9, 141.0, 133.8, 130.1, 128.2, 127.4, 112.9, 80.0, 79.4, 75.4, 45.5, 43.2, 40.3, 38.4, 28.4, 28.4; **MS (ESI$^+$)** 474 (100%, $M+H^+$); **HRMS (ESI-TOF)** m/z: $[M + H]^+$ Calcd for $C_{25}H_{36}N_3O_6$ 474.2604; Found 474.2590; **HPLC** (Cellulose-1, hexane:$^i$PrOH 97.5:2.5, flow rate 1.0 mL/min, λ = 254 nm, 22 °C) $t_R$(major) = 18.007, $t_R$(minor) = 27.023, er = 94:6.

**Benzyl-(*R*)-((4-(2-(((*tert*-butoxycarbonyl)amino)methyl)allyl)-4-isobutyl-5-oxo-4,5-dihydrooxazol-2-yl)methyl)carbamate**

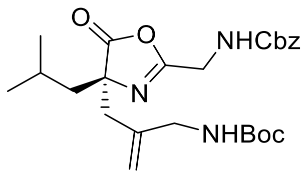


*N*-Boc-5-methylenecyclohexacarbamate (**2**) (21 mg, 0.1 mmol), (*R*,*R*)-ANDEN-phenyl Trost ligand (6 mg, 0.0075 mmol), {$\eta^3$-$C_3H_5PdCl$}$_2$ (1 mg, 0.0025 mmol), $^t$BuOH (0.045 ml, 0.5 mmol) and DIPEA (0.025 ml, 0.2 mmol) were dissolved in dry 20% toluene in dioxane (1 mL) at 0 °C for 20 mins. Then benzyl (4-isobutyl-5-oxo-4,5-dihydro-oxazol-2-ylmethyl)-carbamic acid (**1l**) (91 mg, 0.3 mmol) was added. The reaction mixture was stirred at 0 °C overnight. The solvent was removed under reduced pressure and residue purified by flash column chromatography eluting with 50% $Et_2O$ in petrol to give benzyl-(*R*)-((4-(2-(((*tert*-butoxycarbonyl)amino)methyl)allyl)-4-isobutyl-5-oxo-4,5-dihydrooxazol-2-yl)methyl)-carbamate (43 mg, 90%) as a colorless oil.

**FTIR** $\nu_{max}$ (thin film/cm$^{-1}$) 3356, 2960, 1825, 1692, 1513; **$^1$H NMR (400 MHz, $CDCl_3$)** δ 7.41 – 7.27 (m, 5H, $CH_{Ar}$), 6.50 (t, *J* = 6.0 Hz, 1H, NH), 5.14 (s, 2H, $CH_2$), 5.07 – 4.99 (m, 1H, NH), 4.91 (s, 1H, $C=CH_2$), 4.86 (s, 1H, $C=CH_2$), 4.32 – 4.15 (m, 2H, $CH_2$), 3.96 (dd, J = 18.0, 8.0 Hz, 1H, $CH_2$), 3.26 (dd, J = 18.0, 5.0 Hz, 1H), 2.61 (d, J = 13.5 Hz, 1H, $CH_2$), 2.29 (d, J = 13.5 Hz, 1H, $CH_2$), 1.83 (dd, J = 14.0, 5.5 Hz, 1H, $CH_2$), 1.74 (dd, J = 14.0, 7.0 Hz, 1H, $CH_2$), 1.55 (dp, J = 13.5, 6.5 Hz, 1H, CH), 1.39 (s, 9H, $C(CH_3)_3$), 0.89 (d, J = 6.5 Hz, 3H, $CH_3$), 0.85 (d, J = 6.5 Hz, 3H, $CH_3$) ; **$^{13}$C NMR (101 MHz, $CDCl_3$)** δ 180.2, 161.4, 156.8, 156.0, 140.7, 136.5, 128.4, 128.1, 128.0, 112.5, 79.7, 73.9, 67.0, 45.9, 45.8, 41.6, 40.0, 28.3, 24.7, 24.1, 23.1; **MS (ESI$^+$)** 474 (100%, $M+H^+$); **HRMS (ESI-TOF)** m/z: $[M + H]^+$ Calcd for $C_{25}H_{36}N_3O_6$ 474.2604; Found 474.2587; **HPLC** (Cellulose-1,

hexane:$^{i}$PrOH 97.5:2.5, flow rate 1.0 mL/min, λ = 254 nm, 22 °C) $t_R$(major) = 40.313, $t_R$(minor) = 50.510, er = 93:7.

## Synthesis of Lactams

### (*R*)-*N*-(3-Benzyl-5-methylene-2-oxopiperidin-3-yl)benzamide, 3

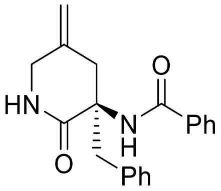


To a solution of *tert*-butyl (*R*)-(2-((4-benzyl-5-oxo-2-phenyl-4,5-dihydrooxazol-4-yl)methyl)-allyl)carbamate (84 mg, 0.2 mmol) dissolved in dry DCM (2 mL) was added TFA (1.2 mL, 15 mmol) and the reaction stirred at room temperature. After 1 hour, EtOAc was added and the reaction was quenched with sat. aq. $NaHCO_3$. The layers were separated, and the aqueous layer was further extracted with EtOAc. The solvent was removed under reduced pressure and residue purified by flash column chromatography eluting with 60% EtOAc in petrol to give (*R*)-*N*-(3-benzyl-5-methylene-2-oxopiperidin-3-yl)benzamide **(3)** (61 mg, 95%) as a white solid.

Crystallization Method: A sample of product (20 mg) was dissolved in acetone (1 mL). Hexane (9 mL) was added and the sample transferred to a glass tube. Slow evaporation of the solvent gave crystals of **3** for X-ray analysis.

**m.p.:** 63.4 – 64.0 ℃ (After recrystallisation from DCM and hexane); **FTIR** $\nu_{max}$ (thin film/cm$^{-1}$) 3279, 2927, 1667, 1629; **$^1$H NMR (400 MHz, $CDCl_3$)** δ 7.75 – 7.65 (m, 2H, $CH_{Ar}$), 7.57 – 7.45 (m, 1H, $CH_{Ar}$), 7.45 – 7.35 (m, 2H, $CH_{Ar}$), 7.29 – 7.21 (m, 4H, $CH_{Ar}$, NH), 7.20 – 7.10 (m, 2H, $CH_{Ar}$), 6.17 (s, 1H, NH), 5.25 (s, 1H, C=$CH_2$), 5.18 (s, 1H, C=$CH_2$), 4.23 (d, J = 15.0 Hz, 1H, $CH_2$), 4.12 (d, J = 15.0 Hz, 1H, $CH_2$), 3.75 (d, J = 14.0 Hz, 1H, $CH_2$), 3.66 (d, J = 14.0 Hz, 1H, $CH_2$), 3.07 (d, J = 14.0 Hz, 1H, $CH_2$), 2.92 (d, J = 14.0 Hz, 1H, $CH_2$); **$^{13}$C NMR (101 MHz, $CDCl_3$)** δ 172.5, 166.9, 136.5, 135.7, 134.9, 131.5, 130.2, 128.5, 128.3, 127.1, 126.9, 114.1, 59.5, 47.2, 40.0, 39.2; **MS (ESI$^+$)** 321 (100%, M+H$^+$); **HRMS (ESI-TOF)** m/z: [M + H]$^+$ Calcd for $C_{20}H_{21}N_2O_2$ 321.1603; Found 321.1598. **HPLC** (Cellulose-2, hexane:$^{i}$PrOH 90:10, flow rate 1.0 mL/min, λ = 254 nm, 22 °C) $t_R$(major) = 29.823, $t_R$(minor) = 27.390, er = 95:5. $[\alpha]_D^{22} = +30$ (*c* 1.0, $CHCl_3$).

**(*R*)-*N*-(3-Isobutyl-5-methylene-2-oxopiperidin-3-yl)benzamide, 5**

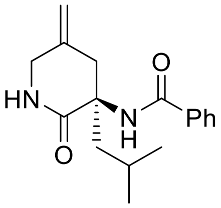


To a solution of *tert*-butyl-(*R*)-(2-((4-isobutyl-5-oxo-2-phenyl-4,5-dihydrooxazol-4-yl)methyl)-allyl)carbamate (**3b**) (77 mg, 0.2 mmol) dissolved in dry DCM (2 mL) was added TFA (1.2 mL, 15 mmol) and the reaction stirred at room temperature. After 1 hour, EtOAc was added and the reaction was quenched with sat. aq. $NaHCO_3$. The layers were separated, and the aqueous layer was further extracted with EtOAc. The solvent was removed under reduced pressure and residue purified by flash column chromatography eluting with 60% EtOAc in petrol to give (*R*)-*N*-(3-isobutyl-5-methylene-2-oxopiperidin-3-yl)benzamide (**5**) (53 mg, 95%) as a white solid.

Crystallization Method: A sample of product (20 mg) was dissolved in acetone (1 mL). Hexane (9 mL) was added and the sample transferred to a glass tube. Slow evaporation of the solvent gave crystals of **5** for X-ray analysis.

**m.p.:** 163.7 – 166.4 ℃ (After recrystallisation from toluene); **FTIR** $\nu_{max}$ (thin film/cm$^{-1}$) 3229, 2971, 1739, 1649; **$^1$H NMR (400 MHz, $CDCl_3$)** δ 7.88 – 7.79 (m, 2H, $CH_{Ar}$), 7.64 (s, 1H, NH), 7.55 – 7.49 (m, 1H, $CH_{Ar}$), 7.50 – 7.41 (m, 2H, $CH_{Ar}$), 6.14 (s, 1H, NH), 5.15 (s, 1H, C=$CH_2$), 5.07 (s, 1H, C=$CH_2$), 4.16 (d, *J* = 15.5 Hz, 1H, $CH_2$), 4.05 (d, J = 15.5 Hz, 1H, $CH_2$), 3.69 (dd, J = 14.0, 3.5 Hz, 1H, $CH_2$), 2.78 (d, J = 14.0 Hz, 1H, $CH_2$), 2.38 (ddd, J = 14.5, 5.5, 1.5 Hz, 1H, $CH_2$), 1.89 – 1.75 (m, 1H, CH), 1.64 (dd, J = 14.5, 6.5 Hz, 1H, $CH_2$), 0.94 (d, J = 6.5 Hz, 3H, $CH_3$), 0.92 (d, J = 6.5 Hz, 3H, $CH_3$); **$^{13}$C NMR (101 MHz, $CDCl_3$)** δ 173.9, 166.3, 136.7, 135.0, 131.4, 128.5, 126.9, 113.4, 58.9, 46.7, 42.3, 40.0, 24.23, 24.20, 23.8; **MS (ESI$^+$)** 287 (100%, M+H$^+$); **HRMS (ESI-TOF)** m/z: [M + H]$^+$ Calcd for $C_{17}H_{23}N_2O_2$ 287.1754; Found 287.1752. **HPLC** (Cellulose-2, hexane:$^i$PrOH 80:20, flow rate 1.0 mL/min, λ = 254 nm, 22 °C) $t_R$(major) = 10.203, $t_R$(minor) = 11.650, er = 90:10.

**(*S*)-*N*-(5-Methylene-3-(2-(methylthio)ethyl)-2-oxopiperidin-3-yl)benzamide, 6**

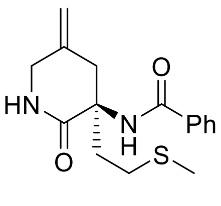


To a solution of *tert*-butyl-(*S*)-(2-((4-(2-(methylthio)ethyl)-5-oxo-2-phenyl-4,5-dihydrooxazol-4-yl)methyl)allyl)carbamate (**3c**) (80 mg, 0.2 mmol) dissolved in dry DCM (2 mL) was added

TFA (1.2 mL, 15 mmol) and the reaction stirred at room temperature. After 1 hour, EtOAc was added and the reaction was quenched with sat. aq. $NaHCO_3$. The layers were separated, and the aqueous layer was further extracted with EtOAc. The solvent was removed under reduced pressure and residue purified by flash column chromatography eluting with 60% EtOAc in petrol to give (*S*)-*N*-(5-methylene-3-(2-(methylthio)ethyl)-2-oxopiperidin-3-yl)benzamide, (**6**) (56 mg, 93%) as a white solid.

**FTIR** $\nu_{max}$ (thin film/cm$^{-1}$) 3257, 2966, 1662, 1652; **$^1$H NMR (400 MHz, $CDCl_3$)** δ 7.89 – 7.79 (m, 2H, $CH_{Ar}$), 7.72 (s, 1H, NH), 7.56 – 7.50 (m, 1H, $CH_{Ar}$), 7.48 – 7.44 (m, 2H, $CH_{Ar}$), 6.15 (s, 1H, NH), 5.17 (s, 1H, C=$CH_2$), 5.11 (s, 1H, C=$CH_2$), 4.18 (d, J = 15.0 Hz, 1H, $CH_2$), 4.00 (d, J = 15.0 Hz, 1H, $CH_2$), 3.57 (d, J = 14.0 Hz, 1H, $CH_2$), 2.92 (d, J = 14.0 Hz, 1H, $CH_2$), 2.73 – 2.46 (m, 3H, $CH_2$), 2.10 (s, 3H, $CH_3$), 2.08 – 1.96 (m, 1H, $CH_2$); **$^{13}$C NMR (101 MHz, $CDCl_3$)** δ 172.7, 166.6, 136.1, 134.5, 131.7, 128.6, 127.0, 114.1, 59.0, 47.2, 39.5, 34.3, 28.5, 15.7; **MS (ESI$^+$)** 305 (100%, M+H$^+$); **HRMS (ESI-TOF)** m/z: [M + H]$^+$ Calcd for $C_{21}H_{29}N_2O_4S$ 305.1328; Found 321.1318.

**(*R*)-*N*-(3-butyl-5-methylene-2-oxopiperidin-3-yl)benzamide, 7**

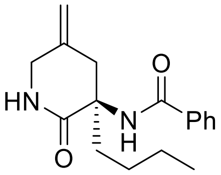


To a solution of *tert*-butyl-(*R*)-(2-((4-butyl-5-oxo-2-phenyl-4,5-dihydrooxazol-4-yl)methyl)-allyl)carbamate (**3d**) (77 mg, 0.2 mmol) dissolved in dry DCM (2 mL) was added TFA (1.2 mL, 15 mmol) and the reaction stirred at room temperature. After 1 hour, EtOAc was added and the reaction was quenched with sat. aq. $NaHCO_3$. The layers were separated, and the aqueous layer was further extracted with EtOAc. The solvent was removed under reduced pressure and residue purified by flash column chromatography eluting with 80% EtOAc in petrol to give (*R*)-*N*-(3-butyl-5-methylene-2-oxopiperidin-3-yl)benzamide (**7**) (52 mg, 94%) as a white solid.

**FTIR** $\nu_{max}$ (thin film/cm$^{-1}$) 3275, 2961, 1659, 1629; **$^1$H NMR (400 MHz, $CDCl_3$)** δ 7.85 – 7.79 (m, 2H, $CH_{Ar}$), 7.54 – 7.46 (m, 1H, $CH_{Ar}$), 7.47 – 7.39 (m, 3H, $CH_{Ar}$, NH), 6.52 (s, 1H, NH), 5.12 (s, 1H, C=$CH_2$), 5.05 (s, 1H, C=$CH_2$), 4.12 (d, J = 15.0 Hz, 1H, $CH_2$), 3.95 (d, J = 15.0 Hz, 1H, $CH_2$), 3.50 (d, J = 14.0Hz, 1H, $CH_2$), 2.90 (d, J = 14.0 Hz, 1H, $CH_2$), 2.29 – 2.21 (m, 1H, $CH_2$), 1.76 – 1.64 (m, 1H, $CH_2$), 1.42 – 1.21 (m, 4H, 2$CH_2$), 0.86 (t, J = 7.0 Hz, 3H, $CH_3$); **$^{13}$C NMR (101 MHz, $CDCl_3$)** δ 173.8, 166.8, 136.5, 134.7, 131.5, 128.5, 127.0, 113.5, 58.9, 46.9, 39.1, 34.6, 25.7, 22.7, 13.9;

**MS (ESI$^+$)** 287 (100%, M+H$^+$); **HRMS (ESI-TOF)** m/z: [M + H]$^+$ Calcd for $C_{17}H_{23}N_2O_2$ 287.1765; Found 287.1754.

**(*S*)-*N*-(3-((1*H*-Indol-2-yl)methyl)-5-methylene-2-oxopiperidin-3-yl)benzamide, 8**

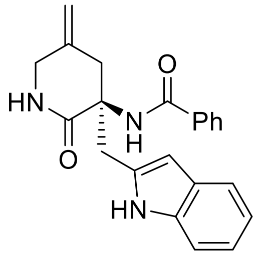


To a solution of *tert*-butyl (*S*)-(2-((4-((1*H*-indol-2-yl)methyl)-5-oxo-2-phenyl-4,5-dihydrooxazol-4-yl)methyl)allyl)carbamate (**3e**) (92 mg, 0.2 mmol) dissolved in dry DCM (4 mL) was added TFA (0.13 mL, 1.6 mmol) and the reaction stirred at room temperature. After 2 hours, EtOAc was added and the reaction was quenched with sat. aq. $NaHCO_3$. The layers were separated, and the aqueous layer was further extracted with EtOAc. The solvent was removed under reduced pressure and residue purified by flash column chromatography eluting with 80% EtOAc in petrol to give (*S*)-*N*-(3-((1*H*-indol-2-yl)methyl)-5-methylene-2-oxopiperidin-3-yl)benzamide (**8**) (68 mg, 95%) as a white solid.

**FTIR** $\nu_{max}$ (thin film/cm$^{-1}$) 3288, 2923, 1653; **$^1$H NMR (400 MHz, $CDCl_3$)** δ 10.97 (s, 1H, NH), 7.82 (s, 1H, NH), 7.78 – 7.62 (m, 3H, $CH_{Ar}$), 7.62 – 7.48 (m, 2H, $CH_{Ar}$), 7.35-7.41 (m, 2H, $CH_{Ar}$), 7.35 (d, J = 8.0 Hz, 1H, $CH_{Ar}$), 7.20 (s, 1H, NH), 7.06 (t, J = 7.5 Hz, 1H, $CH_{Ar}$), 6.97 (t, J = 7.4 Hz, 1H, $CH_{Ar}$), 5.01 (s, 1H, C=$CH_2$), 4.91 (s, 1H, C=$CH_2$), 4.01 (d, J = 15.0 Hz, 1H, $CH_2$), 3.88 (d, J = 15.0 Hz, 1H, $CH_2$), 3.41 (d, J = 14.0 Hz, 1H, $CH_2$), 3.24 (d, J = 14.0 Hz, 1H, $CH_2$), 3.04 (d, J = 14.0 Hz, 1H, $CH_2$), 2.98 (d, J = 14.0 Hz, 1H, $CH_2$);**$^{13}$C NMR (101 MHz, $CDCl_3$)** δ 171.8, 166.3, 138.8, 136.3, 135.2, 131.8, 128.8, 128.7, 127.4, 125.3, 121.3, 118.9, 118.7, 112.5, 111.9, 108.5, 59.5, 46.7, 38.7, 31.1; **MS (ESI$^+$)** 360 (100%, M+H$^+$); **HRMS (ESI-TOF)** m/z: [M + H]$^+$ Calcd for $C_{22}H_{22}N_3O_2$ 360.1712; Found 360.1716.

**Benzyl (*S*)-(2-(3-benzamido-5-methylene-2-oxopiperidin-3-yl)ethyl)carbamate, 9**

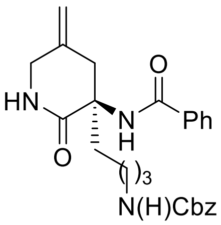


To a solution of *tert*-butyl (*R*)-(2-((4-(4-(((benzyloxy)carbonyl)amino)butyl)-5-oxo-2-phenyl-4,5-dihydrooxazol-4-yl)methyl)allyl)-carbamate (**3f**) (107 mg, 0.2 mmol) dissolved in dry DCM (2 mL) was added TFA (1.2 mL, 15 mmol) and the reaction stirred at room temperature. After 1 hour, EtOAc was added and the reaction was quenched with sat. aq. $NaHCO_3$. The layers were separated, and the aqueous layer was further extracted with EtOAc. The solvent was removed under reduced pressure and residue purified by flash column chromatography eluting with 90% EtOAc in petrol to give benzyl (*S*)-(2-(3-benzamido-5-methylene-2-oxopiperidin-3-yl)ethyl)carbamate (**9**) (80 mg, 92%) as a white solid.

**FTIR** $\nu_{max}$ (thin film/cm$^{-1}$) 3318, 2950, 1670, 1645; **$^1$H NMR (400 MHz, $CDCl_3$)** δ 7.83 – 7.79 (m, 2H, $CH_{Ar}$), 7.62 (s, 1H, NH), 7.44 (t, J = 7.5 Hz, 1H, $CH_{Ar}$), 7.40 – 7.17 (m, 7H, $CH_{Ar}$), 7.06 (s, 1H, NH), 5.31 (s, 1H, NH), 5.14 – 4.82 (m, 4H, C=$CH_2$, $CH_2$), 4.03 (d, J = 15.0 Hz, 1H, $CH_2$), 3.82 (d, J = 15.0 Hz, 1H, $CH_2$), 3.37 – 3.04 (m, 3H, $CH_2$), 2.97 (d, J = 14.0 Hz, 1H, $CH_2$), 2.16 – 2.09 (m, 1H, $CH_2$), 1.85 – 1.56 (m, 1H, $CH_2$), 1.53 – 1.14 (m, 4H, 2$CH_2$);**$^{13}$C NMR (101 MHz, $CDCl_3$)** δ 173.8, 167.4, 156.9, 136.7, 136.6, 134.5, 131.5, 128.5, 128.4, 128.0, 128.0, 127.2, 113.3, 66.5, 58.7, 46.9, 40.0, 38.7, 34.4, 29.6, 20.1; **MS (ESI$^+$)** 436 (100%, M+H$^+$); **HRMS (ESI-TOF)** m/z: $[M + H]^+$ Calcd for $C_{23}H_{26}N_3O_4$ 436.2236; Found 436.2239.

**(*R*)-*N*-(3-(4-Hydroxybenzyl)-5-methylene-2-oxopiperidin-3-yl)benzamide, 10**

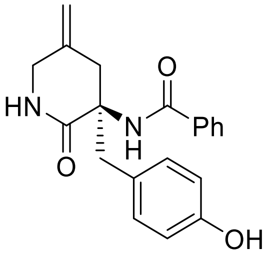


To a solution of *tert*-butyl (*R*)-(2-((4-(4-hydroxybenzyl)-5-oxo-2-phenyl-4,5-dihydrooxazol-4-yl)methyl)-allyl)carbamate (**3g**) (87 mg, 0.2 mmol) dissolved in dry DCM (2 mL) was added TFA (1.2 mL, 15 mmol) and the reaction stirred at room temperature. After 1 hour, EtOAc was added and the reaction was quenched with sat. aq. $NaHCO_3$. The layers were separated, and

the aqueous layer was further extracted with EtOAc. The solvent was removed under reduced pressure and residue purified by flash column chromatography eluting with EtOAc to give (*R*)-*N*-(3-(4-hydroxybenzyl)-5-methylene-2-oxopiperidin-3-yl)benzamide (**10**) (63 mg, 93%) as a white solid.

**FTIR** $\nu_{max}$ (thin film/cm$^{-1}$) 3296, 2942, 2831, 1645; **$^1$H NMR (400 MHz, MeOD)** δ 7.78 – 7.66 (m, 2H, $CH_{Ar}$), 7.59 – 7.48 (m, 1H, $CH_{Ar}$), 7.51 – 7.37 (m, 2H, $CH_{Ar}$), 7.12 – 6.97 (m, 2H, $CH_{Ar}$), 6.78 – 6.63 (m, 2H, $CH_{Ar}$), 5.08 (s, 1H, C=$CH_2$), 5.04 (s, 1H, C=$CH_2$), 4.14 (d, J = 15.0 Hz, 1H, $CH_2$), 3.92 (d, J = 15.0 Hz, 1H, $CH_2$), 3.33 (d, J = 13.5 Hz, 1H, $CH_2$), 3.15 (d, J = 14.0 Hz, 1H, $CH_2$), 3.07 (d, J = 13.5 Hz, 1H, $CH_2$), 3.02 (d, J = 14.0 Hz, 1H, $CH_2$);**$^{13}$C NMR (101 MHz, MeOD)** δ 172.9, 168.0, 156.4, 137.2, 134.5, 131.5, 131.2, 128.3, 126.7, 125.8, 114.7, 112.2, 59.3, 46.4, 40.1, 38.3; **MS (ESI$^+$)** 337 (100%, M+H$^+$); **HRMS (ESI-TOF)** m/z: [M + H]$^+$ Calcd for $C_{20}H_{21}N_2O_3$ 337.1552; Found 337.1557.

**Methyl (*R*)-3-(3-benzamido-5-methylene-2-oxopiperidin-3-yl)propanoate, 11**

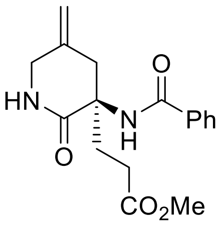


To a solution of methyl (*R*)-3-(4-(2-(((*tert*-butoxycarbonyl)amino)-methyl)allyl)-5-oxo-2-phenyl-4,5-dihydrooxazol-4-yl)propanoate (**3h**) (107 mg, 0.2 mmol) dissolved in dry DCM (2 mL) was added TFA (1.2 mL, 15 mmol) and the reaction stirred at room temperature. After 1 hour, EtOAc was added and the reaction was quenched with sat. aq. $NaHCO_3$. The layers were separated, and the aqueous layer was further extracted with EtOAc. The solvent was removed under reduced pressure and residue purified by flash column chromatography eluting with 90% EtOAc in petrol to give methyl (*R*)-3-(3-benzamido-5-methylene-2-oxopiperidin-3-yl)propanoate (**11**) (80 mg, 92%) as a white solid.

**FTIR** $\nu_{max}$ (thin film/cm$^{-1}$) 3363, 2958, 1667, 1643; **$^1$H NMR (400 MHz, CDCl$_3$)** δ 8.05 (s, 1H, NH), 7.81 (d, J = 7.5 Hz, 2H, $CH_{Ar}$), 7.51 – 7.42 (m, 1H, $CH_{Ar}$), 7.42 – 7.32 (m, 2H, $CH_{Ar}$), 7.12 (s, 1H, NH), 5.05 (s, 1H, C=$CH_2$), 5.07 (s, 1H, C=$CH_2$), 4.09 (d, J = 15.0 Hz, 1H, $CH_2$), 3.82 (d, J = 15.0 Hz, 1H, $CH_2$), 3.63 (s, 3H, $CH_3$), 3.19 (br, 1H, $CH_2$), 2.98 (br, 1H, $CH_2$), 2.61 (br, 1H, $CH_2$), 2.51 – 2.20 (m, 2H, $CH_2$), 2.21 – 1.96 (m, 1H, $CH_2$);**$^{13}$C NMR (101 MHz, CDCl$_3$)** δ 178.5, 176.3, 172.3, 141.5,

138.2, 135.4, 132.1, 130.9, 116.4, 62.0, 54.9, 50.8, 43.1, 34.7, 31.9.; **MS (ESI$^+$)** 317 (100%, M+H$^+$); **HRMS (ESI-TOF)** m/z: [M + H]$^+$ Calcd for $C_{17}H_{21}N_2O_4$ 317.1501; Found 317.1500.

**(S)-N-(5-methylene-2-oxo-3-phenylpiperidin-3-yl)benzamide, 12**

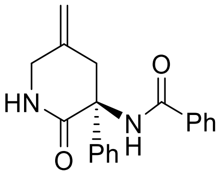


To a solution of tert-butyl (S)-(2-((5-oxo-2,4-diphenyl-4,5-dihydrooxazol-4-yl)methyl)allyl)-carbamate (**3i**) (41 mg, 0.1 mmol) dissolved in dry DCM (1 mL) was added TFA (0.6 mL, 7.5 mmol) and the reaction stirred at room temperature. After 1 hour, EtOAc was added and the reaction was quenched with sat. aq. $NaHCO_3$. The layers were separated, and the aqueous layer was further extracted with EtOAc. The solvent was removed under reduced pressure and residue purified by flash column chromatography eluting with 90% EtOAc in petrol to give (S)-N-(5-methylene-2-oxo-3-phenylpiperidin-3-yl)benzamide (**12**) (29 mg, 95%) as a white solid.

**FTIR** $\nu_{max}$ (thin film/cm$^{-1}$) 3251, 3062, 1685, 1650; **$^1$H NMR (400 MHz, CDCl$_3$)** δ 8.18 – 8.06 (s, 1H, NH), 7.87 – 7.76 (m, 2H, $CH_{Ar}$), 7.68 – 7.58 (m, 2H, $CH_{Ar}$), 7.56 – 7.45 (m, 1H, $CH_{Ar}$), 7.47 – 7.38 (m, 2H, $CH_{Ar}$), 7.39 – 7.26 (m, 3H, $CH_{Ar}$), 6.85 – 6.65 (s, 1H, NH), 5.17 – 5.00 (s, 1H, C=$CH_2$), 5.00 – 4.87 (s, 1H, C=$CH_2$), 4.38 – 4.19 (d, J = 16.0 Hz, 1H, $CH_2$), 4.08 – 3.87 (d, J = 15.5 Hz, 1H, $CH_2$), 3.76 – 3.54 (d, J = 15.5 Hz, 1H, $CH_2$), 3.36 – 3.06 (d, J = 16.0 Hz, 1H, $CH_2$);**$^{13}$C NMR (101 MHz, CDCl$_3$)** δ 172.5, 166.1, 138.0, 137.2, 134.5, 131.6, 128.6, 128.5, 128.2, 127.4, 127.1, 112.2, 61.1, 45.9, 37.8; **MS (ESI$^+$)** 307 (100%, M+H$^+$); **HRMS (ESI-TOF)** m/z: [M + H]$^+$ Calcd for $C_{19}H_{20}N_2O_2$ 307.1447; Found 307.1452.

**Benzyl-(*R*)-(2-((3-benzyl-5-methylene-2-oxopiperidin-3-yl)amino)-2-oxoethyl)carbamate, 13**

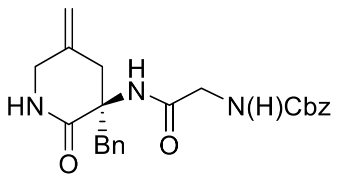


To a solution of *tert*-butyl (*R*)-(2-((4-benzyl-2-((((benzyloxy)carbonyl)amino)methyl)-5-oxo-4,5-dihydrooxazol-4-yl)methyl)allyl)-carbamate (**3j**) (102 mg, 0.2 mmol) dissolved in dry DCM

(2 mL) was added TFA (1.2 mL, 15 mmol) and the reaction stirred at room temperature. After 1 hour, EtOAc was added and the reaction was quenched with sat. aq. $NaHCO_3$. The layers were separated, and the aqueous layer was further extracted with EtOAc. The solvent was removed under reduced pressure and residue purified by flash column chromatography eluting with 90% EtOAc in petrol to give benzyl-(*R*)-(2-((3-benzyl-5-methylene-2-oxopiperidin-3-yl)amino)-2-oxoethyl)-carbamate (**13**) (75 mg, 92%) as a white solid.

**FTIR** $\nu_{max}$ (thin film/cm$^{-1}$) 3315, 2952, 1723, 1667; **$^1$H NMR (400 MHz, CDCl$_3$)** δ 7.49 – 7.19 (m, 8H, $CH_{Ar}$), 7.18 – 7.00 (m, 4H, $CH_{Ar,}$ NH), 6.13 (s, 1H, NH), 5.09 (s, 4H, C=$CH_2$, $CH_2$), 4.07 (d, J = 15.0 Hz, 1H, $CH_2$), 3.97 – 3.80 (m, 2H, $CH_2$), 3.71 (d, J = 13.0 Hz, 1H, $CH_2$), 3.25 (d, J = 13.5 Hz, 1H, $CH_2$), 3.13 – 2.78 (m, 3H, $CH_2$);**$^{13}$C NMR (101 MHz, CDCl$_3$)** δ 172.8, 169.4, 156.6, 136.4, 136.3, 134.9, 130.4, 128.5 (×2C), 128.2, 128.1, 127.3, 114.1, 67.0, 58.9, 47.2, 44.7, 40.6, 37.9; **MS (ESI$^+$)** 408 (100%, M+H$^+$); **HRMS (ESI-TOF)** m/z: $[M + H]^+$ Calcd for $C_{23}H_{26}N_3O_4$ 408.1936; Found 408.1918. $[\alpha]_D^{22} = +40$ (*c* 1.0, $CHCl_3$).

**(*R*)-2-Amino-*N*-(3-benzyl-5-methylene-2-oxopiperidin-3-yl)acetamide, 14**

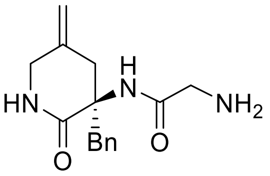


To a solution of *tert*-butyl (*R*)-(2-((4-benzyl-2-(((tert-butoxycarbonyl)amino)methyl)-5-oxo-4,5-dihydrooxazol-4-yl)methyl)allyl)-carbamate (**3k**) (95 mg, 0.2 mmol) dissolved in dry DCM (2 mL) was added TFA (1.2 mL, 15 mmol) and the reaction stirred at room temperature. After 1 hour, EtOAc was added and the reaction was quenched with sat. aq. $NaHCO_3$. The layers were separated, and the aqueous layer was further extracted with EtOAc. The solvent was removed under reduced pressure and residue purified by flash column chromatography eluting with 90% EtOAc in petrol to give (*R*)-2-amino-*N*-(3-benzyl-5-methylene-2-oxopiperidin-3-yl)acetamide (**14**) (50 mg, 90%) as a colorless oil.

**FTIR** $\nu_{max}$ (thin film/cm$^{-1}$) 3298, 2986, 1672; **$^1$H NMR (400 MHz, MeOD)** δ 7.36 – 7.11 (m, 5H, $CH_{Ar}$), 4.99 (s, 1H, C= $CH_2$), 4.92 (s, 1H, C= $CH_2$), 4.06 (d, J = 15.0 Hz, 1H, $CH_2$), 3.81 (d, J = 15.0 Hz, 1H, $CH_2$), 3.77 – 3.57 (m, 2H, $CH_2$), 3.26 (d, J = 13.5 Hz, 1H, $CH_2$), 3.11 – 2.99 (m, 2H, $CH_2$), 2.92 (d, J = 14.0 Hz, 1H, $CH_2$);**$^{13}$C NMR (101 MHz, MeOD)** δ 172.6, 166.0, 136.8, 135.1, 130.4,

127.8, 126.8, 112.3, 59.4, 46.3, 41.4, 40.6, 38.5; **MS (ESI$^+$)** 274 (100%, M+H$^+$); **HRMS (ESI-TOF)** m/z: [M + H]$^+$ Calcd for $C_{15}H_{20}N_3O_2$ 274.1563; Found 274.1556.

**Benzyl-(*R*)-(2-((3-isobutyl-5-methylene-2-oxopiperidin-3-yl)amino)-2-oxoethyl)carbamate, 15**

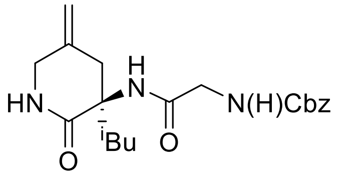


To a solution of benzyl-(*R*)-((4-(2-(((*tert*-butoxycarbonyl)amino)methyl)allyl)-4-isobutyl-5-oxo-4,5-dihydrooxazol-2-yl)methyl)-carbamate (**3l**) (95 mg, 0.2 mmol) dissolved in dry DCM (2 mL) was added TFA (1.2 mL, 15 mmol) and the reaction stirred at room temperature. After 1 hour, EtOAc was added and the reaction was quenched with sat. aq. $NaHCO_3$. The layers were separated, and the aqueous layer was further extracted with EtOAc. The solvent was removed under reduced pressure and residue purified by flash column chromatography eluting with 5% methanol in DCM to give benzyl-(*R*)-(2-((3-isobutyl-5-methylene-2-oxopiperidin-3-yl)amino)-2-oxoethyl)-carbamate (**15**) (71 mg, 95%) as a colorless oil.

**FTIR** $\nu_{max}$ (thin film/cm$^{-1}$) 3287, 2956, 1708, 1651; **$^1$H NMR (400 MHz, $CDCl_3$)** δ 7.38 – 7.26 (m, 6H, $CH_{Ar}$, NH), 6.62 (s, 1H, NH), 5.89 (s, 1H, NH), 5.12 (s, 2H, $CH_2$), 5.05 (s, 1H, C=$CH_2$), 5.01 (s, 1H, C=$CH_2$), 4.05 (d, J = 15.0 Hz, 1H, $CH_2$), 3.98 – 3.77 (m, 3H, $CH_2$), 3.25 (d, J = 14.0 Hz, 1H, $CH_2$), 2.82 (d, J = 14.0 Hz, 1H, $CH_2$), 2.05 (dd, J = 14.5, 5.0 Hz, 1H, $CH_2$), 1.81 – 1.69 (m, 1H, CH), 1.55 (dd, J = 14.5, 5.0 Hz, 1H, $CH_2$), 0.90 (d, J = 6.5 Hz, 3H, $CH_3$), 0.87 (d, J = 6.5 Hz, 3H, $CH_3$);**$^{13}$C NMR (101 MHz, $CDCl_3$)** δ 173.4, 168.6, 156.6, 136.7, 136.3, 128.5, 128.2, 128.1, 113.3, 67.0, 58.8, 46.7, 44.8, 42.8, 39.4, 24.2, 24.0, 23.7; **MS (ESI$^+$)** 374 (100%, M+H$^+$); **HRMS (ESI-TOF)** m/z: [M + H]$^+$ Calcd for $C_{20}H_{28}N_3O_4$ 374.2080; Found 374.2084.

**Methyl-(*R*)-2-((3-isobutyl-5-methylene-2-oxopiperidin-3-yl)carbamoyl)benzoate**

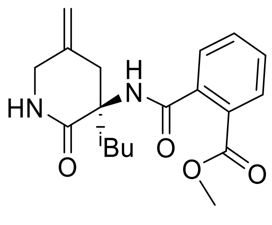

*N*-Boc-5-Methylenecyclohexacarbamate (**2a**) (42 mg, 0.2 mmol), (*R*,*R*)-ANDEN-phenyl Trost ligand (12 mg, 0.015 mmol), {η$^3$-$C_3H_5PdCl$}$_2$ (2 mg, 0.005 mmol), $^t$BuOH (0.09 ml, 1.0 mmol) and DIPEA (0.03 ml, 0.4 mmol) were dissolved in dry 20% toluene in dioxane (2 mL) at 0 °C for 20 mins. Then methyl 2-(4-isobutyl-5-oxo-4,5-dihydrooxazol-2-yl)benzoate (**1m**) (164 mg, 0.6 mmol) was added. The reaction mixture was stirred at 0 °C overnight. The solvent was removed under reduced pressure give the crude product which was dissolved in dry DCM (2 mL). TFA (1.2 mL, 15 mmol) was added and the reaction stirred at room temperature. After 1 hour, EtOAc was added and the reaction was quenched with sat. aq. $NaHCO_3$. The layers were separated, and the aqueous layer was further extracted with EtOAc. The solvent was removed under reduced pressure and residue purified by flash column chromatography eluting with EtOAc to give methyl (*R*)-2-((3-isobutyl-5-methylene-2-oxopiperidin-3-yl)carbamoyl)benzoate (64 mg, 93%) as a colorless oil.

**FTIR** $v_{max}$ (thin film/cm$^{-1}$) 3262, 2953, 1727, 1650; **$^1$H NMR (400 MHz, $CDCl_3$)** δ 7.89 – 7.82 (m, 1H, $CH_{Ar}$), 7.59 – 7.38 (m, 3H, $CH_{Ar}$), 7.16 (s, 1H, NH), 6.51 (s, 1H, NH), 5.13 (s, 1H, C=$CH_2$), 5.05 (s, 1H, C=$CH_2$), 4.12 (d, J = 15.5 Hz, 1H, $CH_2$), 4.00 (d, J = 15.5 Hz, 1H, $CH_2$), 3.88 (s, 3H, $CH_3$), 3.66 (d, J = 14.0 Hz, 1H, $CH_2$), 2.89 (d, J = 14.0 Hz, 1H, $CH_2$), 2.34 (dd, J = 14.5, 5.0 Hz, 1H), 1.87 (dp, J = 13.0, 6.5 Hz, 1H, CH), 1.62 (dd, J = 14.5, 5.0 Hz, 1H, $CH_2$), 1.01 (d, J = 6.5 Hz, 3H, $CH_3$), 0.90 (d, J = 6.5 Hz, 3H, $CH_3$);**$^{13}$C NMR (101 MHz, $CDCl_3$)** δ 173.7, 168.0, 167.2, 138.6, 136.8, 131.8, 130.0, 129.6, 129.4, 127.4, 113.3, 59.2, 52.4, 46.6, 42.7, 39.3, 24.4, 24.2, 23.6; **MS (ESI$^+$)** 345 (100%, M+H$^+$); **HRMS (ESI-TOF)** m/z: [M + H]$^+$ Calcd for $C_{19}H_{23}N_2O_4$ 345.1814; Found 345.1810.

**Methyl-(*R*)-2-((3-benzyl-5-methylene-2-oxopiperidin-3-yl)carbamoyl)benzoate**

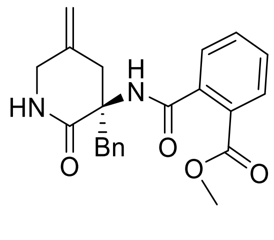


*N*-Boc-5-Methylenecyclohexacarbamate (**2a**) (42 mg, 0.2 mmol), (*R*,*R*)-ANDEN-phenyl Trost ligand (12 mg, 0.015 mmol), {η$^3$-$C_3H_5PdCl$}$_2$ (2 mg, 0.005 mmol), $^t$BuOH (0.09 ml, 1.0 mmol) and DIPEA (0.03 ml, 0.4 mmol) were dissolved in dry 20% toluene in dioxane (2 mL) at 0 °C for 20 mins. Then methyl 2-(4-benzyl-5-oxo-4,5-dihydrooxazol-2-yl)benzoate (**1n**) (186 mg, 0.6 mmol) was added. The reaction mixture was stirred at 0 °C overnight. The solvent was

removed under reduced pressure give the crude product which was dissolved in dry DCM (2 mL). TFA (1.2 mL, 15 mmol) was added and the reaction stirred at room temperature. After 1 hour, EtOAc was added and the reaction was quenched with sat. aq. $NaHCO_3$. The layers were separated, and the aqueous layer was further extracted with 60% EtOAc. The solvent was removed under reduced pressure and residue purified by flash column chromatography eluting with EtOAc to give methyl (*R*)-2-((3-benzyl-5-methylene-2-oxopiperidin-3-yl)carbamoyl)benzoate (72 mg, 95%) as a colorless oil.

**FTIR** $\nu_{max}$ (thin film/cm$^{-1}$) 3264, 2951, 1726, 1650; **$^1$H NMR (400 MHz, $CDCl_3$)** δ 7.93 – 7.74 (m, 1H, $CH_{Ar}$), 7.55 – 7.38 (m, 2H, $CH_{Ar}$), 7.37 – 7.12 (m, 6H, $CH_{Ar}$), 6.95 (s, 1H, NH), 6.92 (s, 1H, NH), 5.20 (s, 1H, $C=CH_2$), 5.11 (s, 1H, $C=CH_2$), 4.12 (d, J = 14.0 Hz, 1H, $CH_2$), 4.01 (d, J = 14.0 Hz, 1H, $CH_2$), 3.85 (s, 3H, $CH_3$), 3.72 (d, J = 14.0 Hz, 1H, $CH_2$), 3.61 (d, J = 13.0 Hz, 1H, $CH_2$), 3.07 (d, J = 13.0 Hz, 1H, $CH_2$), 2.99 (d, J = 14.0 Hz, 1H, $CH_2$); **$^{13}$C NMR (101 MHz, $CDCl_3$)** δ 168.8, 168.3, 167.3, 138.1, 136.5, 135.9, 131.8, 130.4, 129.9, 129.7, 129.6, 128.2, 127.3, 127.0, 113.8, 59.8, 52.4, 46.8, 40.4, 38.4; **MS (ESI$^+$)** 379 (100%, M+H$^+$); **HRMS (ESI-TOF)** m/z: [M + H]$^+$ Calcd for $C_{22}H_{23}N_2O_4$ 379.1658; Found 379.1656.

**Methyl-(*S*)-2-((5-methylene-3-(2-(methylthio)ethyl)-2-oxopiperidin-3-yl)carbamoyl)-benzoate**

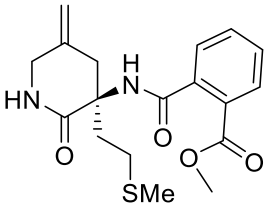


*N*-Boc-5-Methylenecyclohexacarbamate (**2a**) (42 mg, 0.2 mmol), (*R*,*R*)-ANDEN-phenyl Trost ligand (12 mg, 0.015 mmol), {$\eta^3$-$C_3H_5PdCl$}$_2$ (2 mg, 0.005 mmol), $^t$BuOH (0.09 ml, 1.0 mmol) and DIPEA (0.03 ml, 0.4 mmol) were dissolved in dry 20% toluene in dioxane (2 mL) at 0 °C for 20 mins. Then methyl 2-(4-(2-(methylthio)ethyl)-5-oxo-4,5-dihydrooxazol-2-yl)benzoate (**1o**) (176 mg, 0.6 mmol) was added. The reaction mixture was stirred at 0 °C overnight. The solvent was removed under reduced pressure give the crude product which was dissolved in dry DCM (2 mL). TFA (1.2 mL, 15 mmol) was added and the reaction stirred at room temperature. After 1 hour, EtOAc was added and the reaction was quenched with sat. aq. $NaHCO_3$. The layers were separated, and the aqueous layer was further extracted with EtOAc. The solvent was removed under reduced pressure and residue purified by flash column chromatography

eluting with EtOAc to give methyl-(*S*)-2-((5-methylene-3-(2-(methylthio)ethyl)-2-oxopiperidin-3-yl)carbamoyl)-benzoate (67 mg, 93%) as a colorless oil.

**FTIR** $\nu_{max}$ (thin film/cm$^{-1}$) 3279, 2950, 1726, 1652; **$^1$H NMR (400 MHz, CDCl$_3$)** δ 7.93 – 7.77 (m, 1H, $CH_{Ar}$), 7.59 – 7.36 (m, 3H, $CH_{Ar}$), 7.23 (s, 1H, NH), 6.68 (s, 1H, NH), 5.15 (s, 1H, C=$CH_2$), 5.08 (s, 1H, C=$CH_2$), 4.11 (d, J = 15.0 Hz, 1H, $CH_2$), 3.93 (d, J = 15.0 Hz, 1H, $CH_2$), 3.89 (s, 3H, $CH_3$), 3.58 (d, J = 14.0 Hz, 1H, $CH_2$), 2.97 (d, J = 14.0 Hz, 1H, $CH_2$), 2.73 (td, J = 12.0, 5.0 Hz, 1H, $CH_2$), 2.67 – 2.55 (m, 1H, $CH_2$), 2.48 (td, J = 12.0, 5.0 Hz, 1H, $CH_2$), 2.11 (s, 3H, $CH_3$), 2.04 – 1.90 (m, 1H, $CH_2$); **$^{13}$C NMR (101 MHz, CDCl$_3$)** δ 172.7, 168.6, 167.1, 138.3, 136.0, 131.9, 130.0, 129.7, 129.2, 127.6, 114.1, 59.2, 52.5, 47.0, 38.8, 34.7, 28.3, 15.5; **MS (ESI$^+$)** 363 (100%, M+H$^+$); **HRMS (ESI-TOF)** m/z: [M + H]$^+$ Calcd for $C_{18}H_{23}N_2O_4S$ 363.1379; Found 363.1382.

## Methyl (R)-2-((3-(4-(((benzyloxy)carbonyl)amino)butyl)-5-methylene-2-oxopiperidin-3-yl)carbamoyl)benzoate

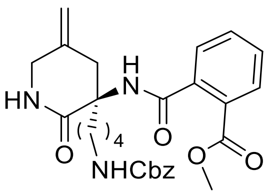


*N*-Boc-5-Methylenecyclohexacarbamate (**2a**) (42 mg, 0.2 mmol), (*R*,*R*)-ANDEN-phenyl Trost ligand (12 mg, 0.015 mmol), {$\eta^3$-$C_3H_5PdCl$}$_2$ (2 mg, 0.005 mmol), $^t$BuOH (0.09 ml, 1.0 mmol) and DIPEA (0.03 ml, 0.4 mmol) were dissolved in dry 20% toluene in dioxane (2 mL) at 0 °C for 20 mins. Then methyl 2-(4-(4-(((benzyloxy)carbonyl)amino)butyl)-5-oxo-4,5-dihydrooxazol-2-yl)benzoate (**1p**) (255 mg, 0.6 mmol) was added. The reaction mixture was stirred at 0 °C overnight. The solvent was removed under reduced pressure give the crude product which was dissolved in dry DCM (2 mL). TFA (1.2 mL, 15 mmol) was added and the reaction stirred at room temperature. After 1 hour, EtOAc was added and the reaction was quenched with sat. aq. $NaHCO_3$. The layers were separated, and the aqueous layer was further extracted with EtOAc. The solvent was removed under reduced pressure and residue purified by flash column chromatography eluting with EtOAc to give methyl (R)-2-((3-(4-(((benzyloxy)-carbonyl)amino)butyl)-5-methylene-2-oxopiperidin-3-yl)carbamoyl)benzoate (92 mg, 93%) as a colorless oil.

**FTIR** $\nu_{max}$ (thin film/cm$^{-1}$) 3322, 2953, 1710, 1652; **$^1$H NMR (400 MHz, CDCl$_3$)** δ 7.86 – 7.84 (m, 1H, $CH_{Ar}$), 7.59 – 7.40 (m, 3H, $CH_{Ar}$), 7.40 – 7.26 (m, 5H, $CH_{Ar}$), 7.19 (s, 1H, NH), 6.19 (s, 1H, NH),

5.30 (s, 1H, NH), 5.13 (s, 1H, C=$CH_2$), 5.06 (s, 1H, C=$CH_2$), 4.98 (s, 2H, $CH_2$), 4.12 (d, J = 15.0 Hz, 1H, $CH_2$), 3.95 (d, J = 15.0 Hz, 1H, $CH_2$), 3.83 (s, 3H, $CH_3$), 3.58 (d, J = 14.0 Hz, 1H, $CH_2$), 3.32 – 3.10 (m, 2H, $CH_2$), 2.98 (d, J = 14.0 Hz, 1H, $CH_2$), 2.44 – 2.26 (m, 1H, $CH_2$), 1.78 – 1.61 (m, 2H, $CH_2$), 1.62 – 1.42 (m, 2H, $CH_2$), 1.40 – 1.16 (m, 1H, $CH_2$); **$^{13}$C NMR (101 MHz, $CDCl_3$)** δ 173.0, 168.6, 167.1, 156.7, 138.7, 136.7, 136.5, 131.9, 130.0, 129.5, 129.1, 128.5, 128.1, 128.0, 127.7, 113.7, 66.5, 59.2, 52.5, 47.0, 40.2, 39.0, 34.0, 29.6, 20.4; **MS (ESI$^+$)** 494 (100%, M+H$^+$); **HRMS (ESI-TOF)** m/z: [M + H]$^+$ Calcd for $C_{27}H_{32}N_3O_6$ 494.2291; Found 494.2300.

**Methyl-(S)-2-((3-(2-methoxy-2-oxoethyl)-5-methylene-2-oxopiperidin-3-yl)carbamoyl)-benzoate**

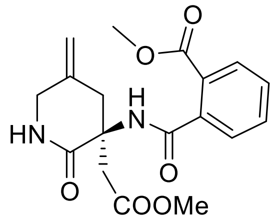


*N*-Boc-5-Methylenecyclohexacarbamate (**2a**) (42 mg, 0.2 mmol), (*R*,*R*)-ANDEN-phenyl Trost ligand (12 mg, 0.015 mmol), {$\eta^3$-$C_3H_5PdCl$}$_2$ (2 mg, 0.005 mmol), $^t$BuOH (0.09 ml, 1.0 mmol) and DIPEA (0.03 ml, 0.4 mmol) were dissolved in dry 20% toluene in dioxane (2 mL) at 0 °C for 20 mins. Then methyl 2-(4-(2-methoxy-2-oxoethyl)-5-oxo-4,5-dihydrooxazol-2-yl)benzoate (**1q**) (175 mg, 0.6 mmol) was added. The reaction mixture was stirred at 0 °C overnight. The solvent was removed under reduced pressure give the crude product which was dissolved in dry DCM (2 mL). TFA (1.2 mL, 15 mmol) was added and the reaction stirred at room temperature. After 1 hour, EtOAc was added and the reaction was quenched with sat. aq. $NaHCO_3$. The layers were separated, and the aqueous layer was further extracted with EtOAc. The solvent was removed under reduced pressure and residue purified by flash column chromatography eluting with EtOAc to give methyl-(S)-2-((3-(2-methoxy-2-oxoethyl)-5-methylene-2-oxopiperidin-3-yl)carbamoyl)-benzoate (65 mg, 90%) as a colorless oil.

**FTIR** $\nu_{max}$ (thin film/cm$^{-1}$) 3265, 2952, 1723, 1651; **$^1$H NMR (400 MHz, $CDCl_3$)** δ 7.88 – 7.86 (m, 1H, $CH_{Ar}$), 7.61 – 7.51 (m, 2H, $CH_{Ar}$), 7.51 – 7.42 (m, 1H, $CH_{Ar}$), 7.32 (s, 1H, NH), 6.71 (s, 1H, NH), 5.10 (s, 2H, $CH_2$), 4.15 (d, J = 14.5 Hz, 1H, $CH_2$), 3.88 (s, 4H, $CH_3$, $CH_2$), 3.69 (s, 3H, $CH_3$), 3.28 (d, J = 14.0 Hz, 1H, $CH_2$), 3.17 (d, J = 14.0 Hz, 1H, $CH_2$), 3.10 (d, J = 14.5 Hz, 1H, $CH_2$), 2.83 (d, J = 14.5 Hz, 1H, $CH_2$); **$^{13}$C NMR (101 MHz, $CDCl_3$)** δ 170.9, 170.8, 168.7, 167.0, 138.1, 136.3, 132.0, 130.0, 129.7, 129.0, 127.7, 114.5, 57.8, 52.4, 52.1, 47.4, 39.2, 38.5; **MS (ESI$^+$)** 361 (100%, M+H$^+$); **HRMS (ESI-TOF)** m/z: [M

+ H]$^+$ Calcd for $C_{18}H_{21}N_2O_6$ 361.1400; Found 361.1408; **HPLC** (SA, hexane:$^i$PrOH 60:40, flow rate 1.0 mL/min, λ = 210 nm, 25 °C) $t_R$(major) = 6.963, $t_R$(minor) = 9.727, er = 90:10.

**Methyl-(R)-2-((3-(3-(benzyloxy)-3-oxopropyl)-5-methylene-2-oxopiperidin-3-yl)carbamoyl)-benzoate**

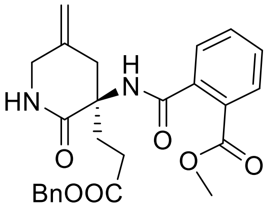


*N*-Boc-5-Methylenecyclohexacarbamate (**2a**) (42 mg, 0.2 mmol), (*R*,*R*)-ANDEN-phenyl Trost ligand (12 mg, 0.015 mmol), {η$^3$-$C_3H_5$PdCl}$_2$ (2 mg, 0.005 mmol), $^t$BuOH (0.09 ml, 1.0 mmol) and DIPEA (0.03 ml, 0.4 mmol) were dissolved in dry 20% toluene in dioxane (2 mL) at 0 °C for 20 mins. Then methyl 2-(4-(3-(benzyloxy)-3-oxopropyl)-5-oxo-4,5-dihydrooxazol-2-yl)benzoate (**1r**) (229 mg, 0.6 mmol) was added. The reaction mixture was stirred at 0 °C overnight. The solvent was removed under reduced pressure give the crude product which was dissolved in dry DCM (2 mL). TFA (1.2 mL, 15 mmol) was added and the reaction stirred at room temperature. After 1 hour, EtOAc was added and the reaction was quenched with sat. aq. $NaHCO_3$. The layers were separated, and the aqueous layer was further extracted with EtOAc. The solvent was removed under reduced pressure and residue purified by flash column chromatography eluting with EtOAc to give methyl-(R)-2-((3-(3-(benzyloxy)-3-oxopropyl)-5-methylene-2-oxopiperidin-3-yl)carbamoyl)- benzoate (86 mg, 95%) as a colorless oil.

**FTIR** $v_{max}$ (thin film/cm$^{-1}$) 3315, 2952, 1727, 1651; **$^1$H NMR (400 MHz, $CDCl_3$)** δ 7.88 – 7.86 (m, 1H, $CH_{Ar}$), 7.64 – 7.43 (m, 3H, $CH_{Ar}$), 7.39 – 7.32 (m, 5H, $CH_{Ar}$), 7.23 (s, 1H, NH), 6.29 (s, 1H, NH), 5.19 – 5.07 (m, 4H, $CH_2$, C=$CH_2$), 4.12 (d, J = 15.0 Hz, 1H, $CH_2$), 3.96 (d, J = 15.0 Hz, 1H, $CH_2$), 3.84 (s, 3H, $CH_3$), 3.61 (d, J = 14.0 Hz, 1H, $CH_2$), 3.01 (d, J = 14.0 Hz, 1H, $CH_2$), 2.75 – 2.58 (m, 2H, $CH_2$), 2.57 – 2.47 (m, 1H, $CH_2$), 2.24 – 2.12 (m, 1H, $CH_2$); **$^{13}$C NMR (101 MHz, $CDCl_3$)** δ 173.3, 172.4, 168.6, 167.1, 138.3, 136.1, 135.9, 131.9, 130.1, 129.7, 129.3, 128.5, 128.3(x2C), 127.5, 114.1, 66.4, 58.5, 52.4, 46.9, 39.1, 29.6, 28.9; **MS (ESI$^+$)** 451 (100%, M+H$^+$); **HRMS (ESI-TOF)** m/z: [M + H]$^+$ Calcd for $C_{25}H_{27}N_2O_6$ 451.1869; Found 451.1973.

**(R)-2-(3-Isobutyl-5-methylene-2-oxopiperidin-3-yl)isoindoline-1,3-dione**

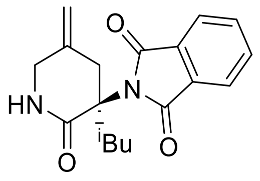

To a solution of methyl (*R*)-2-((3-isobutyl-5-methylene-2-oxopiperidin-3-yl)carbamoyl)-benzoate (62 mg, 0.2 mmol) dissolved in dry THF (2 mL) was added 1,8-diazabicyclo[5.4.0]undec-7-ene (0.03 mL, 0.2 mmol) and the reaction heated at 70 ℃ (DrySyn block) for 24 hours. The solvent was removed under reduced pressure and the residue purified by flash column chromatography eluting with 60% EtOAc in petrol to give (*R*)-2-(3-isobutyl-5-methylene-2-oxopiperidin-3-yl)isoindoline-1,3-dione (56 mg, 90%) as a white solid.

**FTIR** $\nu_{max}$ (thin film/cm$^{-1}$) 3223, 2956, 1708, 1677; **$^1$H NMR (400 MHz, CDCl$_3$)** δ 7.81 (dd, J = 5.5, 3.0 Hz, 2H, $CH_{Ar}$), 7.75 – 7.65 (m, 2H, $CH_{Ar}$), 5.83 (s, 1H, NH), 5.00 (s, 1H, C=$CH_2$), 4.98 (s, 1H, C=$CH_2$), 4.11 (d, J = 15.0 Hz, 1H, $CH_2$), 3.99 (d, J = 15.0 Hz, 1H, $CH_2$), 3.36 (d, J = 14.0 Hz, 1H, $CH_2$), 2.66 (d, J = 14.0 Hz, 1H, $CH_2$), 2.54 (dd, J = 14.5, 4.0 Hz, 1H, $CH_2$), 2.14 (dd, J = 14.5, 6.0 Hz, 1H), 1.95 – 1.81 (m, 1H, CH), 0.97 (d, J = 6.5 Hz, 6H, 2x$CH_3$) **$^{13}$C NMR (101 MHz, CDCl$_3$)** δ 170.4, 168.4, 137.0, 134.0, 131.6, 123.1, 113.1, 63.0, 47.1, 42.3, 40.0, 24.7, 24.4, 24.1; **MS (ESI$^+$)** 313 (100%, M+H$^+$); **HRMS (ESI-TOF)** m/z: [M + H]$^+$ Calcd for $C_{18}H_{21}N_2O_3$ 313.1552; Found 313.1557. **HPLC** (Cellulose-2, hexane:$^i$PrOH 80:20, flow rate 1.0 mL/min, λ = 254 nm, 22 °C) $t_R$(major) = 18.923, $t_R$(minor) = 24.240, er = 92:8.

**(*R*)-2-(3-Benzyl-5-methylene-2-oxopiperidin-3-yl)isoindoline-1,3-dione**

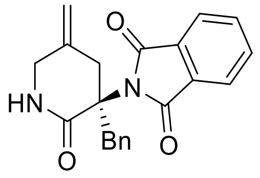

To a solution of methyl (*R*)-2-((3-benzyl-5-methylene-2-oxopiperidin-3-yl)carbamoyl)-benzoate (76 mg, 0.2 mmol) dissolved in dry THF (2 mL) was added 1,8-diazabicyclo[5.4.0]undec-7-ene (0.03 mL, 0.2 mmol) and the reaction heated at 70 ℃ (DrySyn block) for 24 hours. The solvent was removed under reduced pressure and the residue purified by flash column chromatography eluting with 40% EtOAc in petrol to give (*R*)-2-(3-benzyl-5-methylene-2-oxopiperidin-3-yl)isoindoline-1,3-dione (64 mg, 92%) as a white solid.

**FTIR** $\nu_{max}$ (thin film/cm$^{-1}$) 3227, 2933, 1709, 1680; **$^1$H NMR (400 MHz, $CDCl_3$)** δ 7.85 – 7.75 (m, 2H, $CH_{Ar}$), 7.75 – 7.64 (m, 2H, $CH_{Ar}$), 7.47 – 7.36 (m, 2H, $CH_{Ar}$), 7.28 – 7.12 (m, 3H, $CH_{Ar}$), 6.49 (s, 1H, NH), 4.85 (br, 2H, C=$CH_2$), 3.96 – 3.90 (m, 2H, $CH_2$), 3.83 (d, J = 15.0 Hz, 1H, $CH_2$), 3.76 (d, J = 13.0 Hz, 1H, $CH_2$), 3.42 (d, J = 14.5 Hz, 1H, $CH_2$), 2.58 (d, J = 14.5 Hz, 1H, $CH_2$); **$^{13}$C NMR (101 MHz, $CDCl_3$)** δ 169.7, 168.2, 136.8, 136.0, 134.2, 131.5, 131.4, 128.1, 126.9, 123.2, 112.8, 63.9, 46.7, 39.4, 37.8; **MS (ESI$^+$)** 347 (100%, M+H$^+$); **HRMS (ESI-TOF)** m/z: [M + H]$^+$ Calcd for $C_{21}H_{19}N_2O_3$ 347.1396; Found 347.1392. **HPLC** (Cellulose-1, hexane:$^i$PrOH 70:30, flow rate 1.0 mL/min, λ = 254 nm, 22 °C) $t_R$(major) = 11.120, $t_R$(minor) = 21.303, er = 98:2; $[\alpha]_D^{22} = -40$ (*c* 1.0, $CHCl_3$).

**(*S*)-2-(5-Methylene-3-(2-(methylthio)ethyl)-2-oxopiperidin-3-yl)isoindoline-1,3-dione**

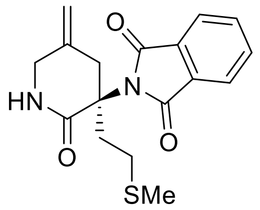


To α solution of methyl (*S*)-2-((5-methylene-3-(2-(methylthio)ethyl)-2-oxopiperidin-3-yl)carbamoyl)-benzoate (73 mg, 0.2 mmol) dissolved in dry THF (2 mL) was added 1,8-diazabicyclo[5.4.0]undec-7-ene (0.03 mL, 0.2 mmol) and the reaction heated at 70 ℃ (DrySyn block) for 24 hours. The solvent was removed under reduced pressure and the residue purified by flash column chromatography eluting with 60% EtOAc in petrol to give (*S*)-2-(5-methylene-3-(2-(methylthio)ethyl)-2-oxopiperidin-3-yl)isoindoline-1,3-dione (58 mg, 88%) as a white solid.

**FTIR** $\nu_{max}$ (thin film/cm$^{-1}$) 3235, 2919, 1712, 1678; **$^1$H NMR (400 MHz, $CDCl_3$)** δ 7.89 – 7.77 (m, 2H, $CH_{Ar}$), 7.78 – 7.62 (m, 2H, $CH_{Ar}$), 6.23 (s, 1H, NH), 5.07 (s, 1H, C=$CH_2$), 5.04 (s, 1H, C=$CH_2$), 4.16 (d, J = 14.5 Hz, 1H, $CH_2$), 3.94 (d, J = 14.5 Hz, 1H, $CH_2$), 3.30 (d, J = 13.5 Hz, 1H, $CH_2$), 3.03 – 2.83 (m, 1H, $CH_2$), 2.80 – 2.58 (m, 3H, $CH_2$), 2.52 – 2.35 (m, 1H, $CH_2$), 2.17 (s, 3H, $CH_3$); **$^{13}$C NMR (101 MHz, $CDCl_3$)** δ 169.9, 168.4, 136.0, 134.2, 131.6, 123.2, 114.2, 62.4, 47.4, 40.6, 35.0, 29.3, 15.3; **MS (ESI$^+$)** 331 (100%, M+H$^+$); **HRMS (ESI-TOF)** m/z: [M + H]$^+$ Calcd for $C_{17}H_{19}N_2O_3S$ 331.1116; Found 331.1113. **HPLC** (Cellulose-1, hexane:$^i$PrOH 70:30, flow rate 1.0 mL/min, λ = 254 nm, 22 °C) $t_R$(major) = 13.273, $t_R$(minor) = 33.117, er = 92:8.

**Benzyl-(R)-(4-(3-(1,3-dioxoisoindolin-2-yl)-5-methylene-2-oxopiperidin-3-yl)butyl)-carbamate**

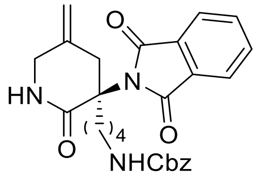


To a solution of methyl (R)-2-((3-(4-(((benzyloxy)carbonyl)amino)butyl)-5-methylene-2-oxopiperidin-3-yl)carbamoyl)benzoate (100 mg, 0.2 mmol) dissolved in dry THF (2 mL) was added 1,8-diazabicyclo[5.4.0]undec-7-ene (0.03 mL, 0.2 mmol) and the reaction heated at 70 ℃ (DrySyn block) for 24 hours. The solvent was removed under reduced pressure and the residue purified by flash column chromatography eluting with 80% EtOAc in petrol to give benzyl-(R)-(4-(3-(1,3-dioxoisoindolin-2-yl)-5-methylene-2-oxopiperidin-3-yl)butyl)-carbamate (83 mg, 90%) as a colorless oil.

**FTIR** $v_{max}$ (thin film/cm$^{-1}$) 3320, 2925, 1708, 1677; **$^1$H NMR (400 MHz, $CDCl_3$)** δ 7.82 – 7.75 (m, 2H, $CH_{Ar}$), 7.74 – 7.66 (m, 2H, $CH_{Ar}$), 7.38 – 7.26 (m, 5H, $CH_{Ar}$), 6.41 (s, 1H, NH), 5.19 (s, 1H, NH), 5.09 (s, 2H, $CH_2$), 5.00 (s, 1H, C= $CH_2$), 4.98 (s, 1H, C= $CH_2$), 4.11 (d, J = 14.5 Hz, 1H, $CH_2$), 3.89 (d, J = 14.5 Hz, 1H, $CH_2$), 3.32 – 3.11 (m, 3H, $CH_2$), 2.65 (d, J = 13.5 Hz, 1H, $CH_2$), 2.56 – 2.47 (m, 1H, $CH_2$), 2.29 – 2.17 (m, 1H, $CH_2$), 1.64 – 1.40 (m, 4H, $CH_2$); **$^{13}$C NMR (101 MHz, $CDCl_3$)** δ 170.4, 168.6, 156.4, 136.8, 136.5, 134.1, 131.7, 128.5, 128.0, 128.0, 123.2, 113.6, 66.4, 62.8, 47.3, 40.4, 40.1, 33.9, 29.5, 21.9; **MS (ESI$^+$)** 462 (100%, M+H$^+$); **HRMS (ESI-TOF)** m/z: $[M + H]^+$ Calcd for $C_{26}H_{28}N_3O_5$ 462.2029; Found 462.2031. **HPLC** (Cellulose-1, hexane:$^i$PrOH 80:20, flow rate 1.0 mL/min, λ = 254 nm, 22 °C) $t_R$(major) = 54.863, $t_R$(minor) = 97.607, er = 96:4; $[\alpha]_D^{22} = -50$ (*c* 1.0, $CHCl_3$).

**Methyl-(S)-2-(3-(1,3-dioxoisoindolin-2-yl)-5-methylene-2-oxopiperidin-3-yl)acetate**

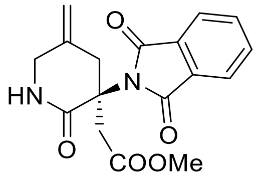


To a solution of methyl-(S)-2-((3-(2-methoxy-2-oxoethyl)-5-methylene-2-oxopiperidin-3-yl)carbamoyl)-benzoate (73 mg, 0.2 mmol) dissolved in dry THF (2 mL) was added 1,8-diazabicyclo[5.4.0]undec-7-ene (0.03 mL, 0.2 mmol) and the reaction heated at 70 ℃ (DrySyn block) for 24 hours. The solvent was removed under reduced pressure and the white solid was filtered. Then, the residue purified by flash column chromatography eluting with 60% EtOAc

in petrol to give methyl-(S)-2-(3-(1,3-dioxoisoindolin-2-yl)-5-methylene-2-oxopiperidin-3-yl)acetate (40 mg, 60%) as a colorless oil.

**FTIR** $\nu_{max}$ (thin film/cm$^{-1}$) 3357, 2951, 1708, 1677; **$^1$H NMR (400 MHz, CDCl$_3$)** δ 7.90 – 7.76 (m, 2H, $CH_{Ar}$), 7.75 – 7.57 (m, 2H, $CH_{Ar}$), 6.48 (s, 1H, NH), 4.98 (s, 1H, C= $CH_2$), 4.96 (s, 1H, C= $CH_2$), 4.07 (d, J = 15.0 Hz, 1H, C= $CH_2$), 4.01 (d, J = 15.0 Hz, 1H, C= $CH_2$), 3.68 (s, 3H, $CH_3$), 3.42 (d, J = 14.0 Hz, 1H, C= $CH_2$), 3.36 (d, J = 16.5 Hz, 1H, C= $CH_2$), 3.25 (d, J = 16.5 Hz, 1H, C= $CH_2$), 3.22 (d, J = 14.0 Hz, 1H, C= $CH_2$); **$^{13}$C NMR (101 MHz, CDCl$_3$)** δ 170.7, 169.7, 168.2, 136.1, 134.2, 131.5, 123.3, 113.9, 60.9, 51.7, 47.0, 37.9, 37.4; **MS (ESI$^+$)** 329 (100%, M+H$^+$); **HRMS (ESI-TOF)** m/z: [M + H]$^+$ Calcd for $C_{17}H_{17}N_2O_5$ 329.1137; Found 329.1130.

**Benzyl-(R)-3-(3-(1,3-dioxoisoindolin-2-yl)-5-methylene-2-oxopiperidin-3-yl)propanoate**

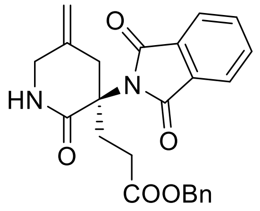


To α solution of methyl-(R)-2-((3-(3-(benzyloxy)-3-oxopropyl)-5-methylene-2-oxopiperidin-3-yl)carbamoyl)- benzoate (90 mg, 0.2 mmol) dissolved in dry THF (2 mL) was added 1,8-diazabicyclo[5.4.0]undec-7-ene (0.03 mL, 0.2 mmol) and the reaction heated at 70 ℃ (DrySyn block) for 24 hours. The solvent was removed under reduced pressure and the residue purified by flash column chromatography eluting with 60% EtOAc in petrol to give benzyl-(R)-3-(3-(1,3-dioxoisoindolin-2-yl)-5-methylene-2-oxopiperidin-3-yl)propanoate (72 mg, 86%) as a colorless oil.

**FTIR** $\nu_{max}$ (thin film/cm$^{-1}$) 3229, 2927, 1712, 1678; **$^1$H NMR (400 MHz, CDCl$_3$)** δ 7.85 – 7.77 (m, 2H, $CH_{Ar}$), 7.77 – 7.69 (m, 2H, $CH_{Ar}$), 7.43 – 7.25 (m, 5H, $CH_{Ar}$), 6.00 (s, 1H, NH), 5.11 (s, 2H, $CH_2$), 5.07 (s, 1H, C=$CH_2$), 5.05 (s, 1H, C=$CH_2$), 4.16 (d, J = 14.5 Hz, 1H, $CH_2$), 3.93 (d, J = 14.5 Hz, 1H, $CH_2$), 3.31 (d, J = 13.5 Hz, 1H, $CH_2$), 3.13 – 2.93 (m, 1H, $CH_2$), 2.90 – 2.72 (m, 1H, $CH_2$), 2.72 – 2.46 (m, 3H, $CH_2$); **$^{13}$C NMR (101 MHz, CDCl$_3$)** δ 173.0, 169.8, 168.4, 136.1, 135.9, 134.2, 131.6, 128.5, 128.1, 128.1, 123.2, 114.2, 66.2, 61.8, 47.4, 40.9, 30.0, 29.5; **MS (ESI$^+$)** 419 (100%, M+H$^+$); **HRMS (ESI-TOF)** m/z: [M + H]$^+$ Calcd for $C_{24}H_{23}N_2O_5$ 419.1607; Found 419.1612. **HPLC** (Cellulose-1, hexane:$^i$PrOH 60:40, flow rate 1.0 mL/min, λ = 254 nm, 22 °C) $t_R$(major) = 21.053, $t_R$(minor) = 38.207, er = 90:10.

**Synthesis of benzamide-free lactam building blocks**

**(*R*)-3-Amino-3-isobutyl-5-methylenepiperidin-2-one, 16**

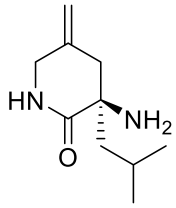


To a solution of (*R*)-2-(3-isobutyl-5-methylene-2-oxopiperidin-3-yl)isoindoline-1,3-dione (66 mg, 0.2 mmol) dissolved in dry $^{i}$PrOH (1 mL) was added ethylenediamine (0.1 mL, 1.6 mmol) and the reaction heated at 80 ℃ (DrySyn block) for 24 hours. The solvent was removed under reduced pressure and residue purified by flash column chromatography eluting with 10% MeOH in DCM to give (*R*)-3-amino-3-isobutyl-5-methylenepiperidin-2-one (**16**) (34 mg, 93%) as a colorless oil.

**FTIR** $\nu_{max}$ (thin film/cm$^{-1}$) 3227, 2953, 1658; **$^{1}$H NMR (400 MHz, $CDCl_3$)** δ 6.19 (s, 1H, NH), 5.08 (s, 1H, $C=CH_2$), 5.06 (s, 1H, $C=CH_2$), 3.94 (s, 2H), 2.60 (d, J = 13.5 Hz, 1H, $CH_2$), 2.38 (d, J = 13.5 Hz, 1H, $CH_2$), 2.05 (s, 2H, $NH_2$), 1.86-1.76 (m, 1H, CH), 1.56 (dd, J = 14.0, 6.0 Hz, 1H, $CH_2$), 1.48 (dd, *J* = 14.5, 6.0 Hz, 1H, $CH_2$), 0.93 (d, *J* = 4.5 Hz, 3H, $CH_3$), 0.91 (d, *J* = 4.5 Hz, 3H, $CH_3$); **$^{13}$C NMR (101 MHz, $CDCl_3$)** δ 177.5, 137.5, 113.0, 56.1, 47.6, 46.8, 41.9, 24.6, 24.5, 23.7; **MS (ESI$^{+}$)** 183 (100%, M+H$^{+}$); **HRMS (ESI-TOF)** m/z: [M + H]$^{+}$ Calcd for $C_{10}H_{19}N_2O$ 183.1492; Found 183.1499.

**(*R*)-3-Amino-3-benzyl-5-methylenepiperidin-2-one, 17**

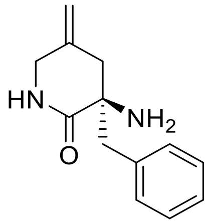


To a solution of (*R*)-2-(3-benzyl-5-methylene-2-oxopiperidin-3-yl)isoindoline-1,3-dione (69 mg, 0.2 mmol) dissolved in dry $^{i}$PrOH (1 mL) was added ethylenediamine (0.1 mL, 1.6 mmol) and the reaction heated at 80 ℃ (DrySyn block) for 24 hours. The solvent was removed under reduced pressure and residue purified by flash column chromatography eluting with 5%

MeOH in DCM to give (*R*)-3-amino-3-benzyl-5-methylenepiperidin-2-one (**17**) (41 mg, 95%) as a colorless oil.

**FTIR** $\nu_{max}$ (thin film/cm$^{-1}$) 3220, 2920, 1657; **$^1$H NMR (400 MHz, $CDCl_3$)** δ 7.39 – 7.19 (m, 5H, $CH_{Ar}$), 6.44 (br, 1H, NH), 5.12 (s, 1H, C=$CH_2$), 5.05 (s, 1H, C=$CH_2$), 4.03 – 3.97 (m, 1H, $CH_2$), 3.98 – 3.89 (m, 1H, $CH_2$), 3.06 (d, J = 13.5 Hz, 1H, $CH_2$), 2.84 (d, J = 13.5 Hz, 1H, $CH_2$), 2.54 (d, J = 13.5 Hz, 1H, $CH_2$), 2.33 (d, J = 13.5 Hz, 1H, $CH_2$), 1.80 (br, 2H, $NH_2$); **$^{13}$C NMR (101 MHz, $CDCl_3$)** δ 176.5, 137.1, 136.2, 130.9, 128.1, 126.8, 113.6, 56.7, 47.9, 44.3, 40.6; **MS (ESI$^+$)** 217 (100%, M+H$^+$); **HRMS (ESI-TOF)** m/z: [M + H]$^+$ Calcd for $C_{13}H_{17}N_2O$ 217.1341; Found 217.1343. $[\alpha]_D^{22} = -40$ (*c* 1.0, $CHCl_3$).

**(*S*)-3-Amino-5-methylene-3-(2-(methylthio)ethyl)piperidin-2-one, 18**

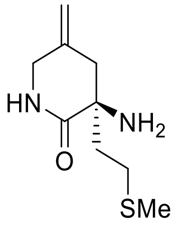


To a solution of (*S*)-2-(5-methylene-3-(2-(methylthio)ethyl)-2-oxopiperidin-3-yl)isoindoline-1,3-dione (69 mg, 0.2 mmol) dissolved in dry $^i$PrOH (1 mL) was added ethylenediamine (0.1 mL, 1.6 mmol) and the reaction heated at 80 ℃ (DrySyn block) for 24 hours. The solvent was removed under reduced pressure and residue purified by flash column chromatography eluting with 5% MeOH in DCM to give (*S*)-3-amino-5-methylene-3-(2-(methylthio)ethyl)piperidin-2-one (**18**) (41 mg, 95%) as a colorless oil.

**FTIR** $\nu_{max}$ (thin film/cm$^{-1}$) 3207, 2916, 1661; **$^1$H NMR (400 MHz, $CDCl_3$)** δ 6.19 (s, 1H, NH), 5.08 (s, 1H, C=$CH_2$), 5.06 (s, 1H, C=$CH_2$), 4.02 (d, *J* = 14.5 Hz, 1H, $CH_2$), 3.96 (d, *J* = 14.5 Hz, 1H, $CH_2$), 2.67 – 2.49 (m, 3H, $CH_2$), 2.42 (d, *J* = 13.5 Hz, 1H, $CH_2$), 2.11 (s, 3H, $CH_3$), 1.95 – 1.84 (m, 2H, $CH_2$), 1.82 (s, 2H, $NH_2$); **$^{13}$C NMR (101 MHz, $CDCl_3$)** δ 176.6, 136.7, 113.6, 55.8, 47.8, 41.4, 38.3, 28.0, 15.6; **MS (ESI$^+$)** 201 (100%, M+H$^+$); **HRMS (ESI-TOF)** m/z: [M + H]$^+$ Calcd for $C_9H_{17}N_2OS$ 201.1062; Found 201.1063.

**Benzyl-(R)-(4-(3-amino-5-methylene-2-oxopiperidin-3-yl)butyl)carbamate, 19**

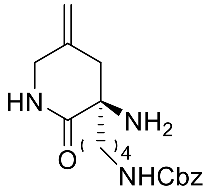


To a solution of benzyl-(R)-(4-(3-(1,3-dioxoisoindolin-2-yl)-5-methylene-2-oxopiperidin-3-yl)butyl)-carbamate (95 mg, 0.2 mmol) dissolved in dry $^{i}$PrOH (1 mL) was added ethylenediamine (0.1 mL, 1.6 mmol) and the reaction heated at 80 ℃ (DrySyn block) for 24 hours. The solvent was removed under reduced pressure and residue purified by flash column chromatography eluting with 10% MeOH in DCM to give (R)-9-methylene-1,7-diazaspiro[4.5]decane-2,6-dione (**19**) (61mg, 92%) as a colorless oil.

**FTIR** $\nu_{max}$ (thin film/cm$^{-1}$) 3287, 2939, 1703, 1650; **$^{1}$H NMR (400 MHz, CDCl3)** δ 7.46 – 7.25 (m, 5H, $CH_{Ar}$), 6.37 (s, 1H, NH), 5.09 (s, 2H, $CH_2$), 5.03 (s, 1H, C= $CH_2$), 5.00 (s, 1H, C= $CH_2$), 3.92 (s, 2H, $CH_2$), 3.32 – 3.09 (m, 2H, $CH_2$), 2.55 (d, *J* = 13.5 Hz, 1H, $CH_2$), 2.36 (d, *J* = 13.5 Hz, 1H, $CH_2$), 1.88 (s, 2H, $NH_2$), 1.65 – 1.53 (m, 2H, $CH_2$), 1.52 – 1.44 (m, 2H, $CH_2$), 1.45 – 1.31 (m, 2H, $CH_2$); **$^{13}$C NMR (101 MHz, CDCl3)** δ 177.1, 156.4, 137.1, 136.7, 128.5, 128.13, 128.07, 113.2, 66.5, 55.8, 47.7, 41.3, 40.7, 38.1, 30.0, 20.2; **MS (ESI$^+$)** 332 (100%, M+H$^+$); **HRMS (ESI-TOF)** m/z: [M + H]$^+$ Calcd for $C_{18}H_{26}N_3O_3$ 332.1974; Found 332.1977. $[\alpha]_D^{22} = -50$ (*c* 1.0, $CHCl_3$).

**Methyl-(S)-2-(3-amino-5-methylene-2-oxopiperidin-3-yl)acetate, 20**

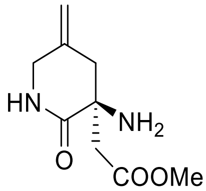


To a solution of methyl-(S)-2-(3-(1,3-dioxoisoindolin-2-yl)-5-methylene-2-oxopiperidin-3-yl)acetate (66 mg, 0.2 mmol) dissolved in dry $^{i}$PrOH (1 mL) was added ethylenediamine (0.1 mL, 1.6 mmol) and the reaction heated at 80 ℃ (DrySyn block) for 24 hours. The solvent was removed under reduced pressure and residue purified by flash column chromatography eluting with 10% MeOH in DCM to give methyl-(S)-2-(3-amino-5-methylene-2-oxopiperidin-3-yl)acetate (**20**) (30 mg, 75%) as a colorless oil.

**FTIR** $\nu_{max}$ (thin film/cm$^{-1}$) 3353, 2954, 1723, 1643; **$^{1}$H NMR (400 MHz, CDCl3)** δ 6.68 (s, 1H, NH), 5.09 (s, 1H, C=$CH_2$), 5.04 (s, 1H, C=$CH_2$), 4.00 (d, J = 14.0 Hz, 1H, $CH_2$), 3.91 (d, J = 14.0 Hz, 1H, $CH_2$), 3.67 (s, 3H, $CH_3$), 2.91 (d, J = 13.5 Hz, 1H, $CH_2$), 2.80 (d, J = 15.5 Hz, 1H, $CH_2$), 2.54 (d, J = 15.5 Hz, 1H, $CH_2$), 2.41 (d, J = 13.5 Hz, 1H, $CH_2$), 2.02 (s, 2H, $NH_2$); **$^{13}$C NMR (101 MHz, CDCl3)**

δ 175.4, 171.4, 136.6, 114.1, 54.8, 51.6, 48.0, 42.5, 41.1; **MS (ESI$^+$)** 199 (100%, M+H$^+$); **HRMS (ESI-TOF)** m/z: $[M + H]^+$ Calcd for $C_9H_{15}N_2O_3$ 199.1083; Found 199.1085.

**(R)-9-methylene-1,7-diazaspiro[4.5]decane-2,6-dione, 21**

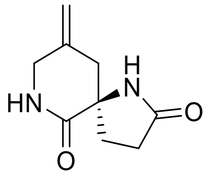

To a solution of benzyl-(R)-3-(3-(1,3-dioxoisoindolin-2-yl)-5-methylene-2-oxopiperidin-3-yl)propanoate (84 mg, 0.2 mmol) dissolved in dry $^i$PrOH (1 mL) was added ethylenediamine (0.1 mL, 1.6 mmol) and the reaction heated at 80 ℃ (DrySyn block) for 24 hours. The solvent was removed under reduced pressure and residue purified by flash column chromatography eluting with 10% MeOH in DCM to give (R)-9-methylene-1,7-diazaspiro[4.5]decane-2,6-dione (**21**) (33mg, 90%) as a colorless oil.

**FTIR** $\nu_{max}$ (thin film/cm$^{-1}$) 3238, 2924, 1678; **$^1$H NMR (400 MHz, MeOD)** δ 5.15 (s, 1H, C=$CH_2$), 5.12 (s, 1H, C=$CH_2$), 4.02 (d, J = 14.5 Hz, 1H, $CH_2$), 3.90 (d, J = 14.5 Hz, 1H, $CH_2$), 2.82 (d, J = 13.0 Hz, 1H, $CH_2$), 2.59 (d, J = 13.0 Hz, 1H, $CH_2$), 2.55 – 2.31 (m, 2H, $CH_2$), 2.30 – 2.15 (m, 1H, $CH_2$), 2.12 – 1.95 (m, 1H, $CH_2$); **$^{13}$C NMR (101 MHz, MeOD)** δ 179.6, 136.4, 112.9, 61.7, 46.9, 41.6, 31.7, 29.4; **MS (ESI$^+$)** 181 (100%, M+H$^+$); **HRMS (ESI-TOF)** m/z: $[M + H]^+$ Calcd for $C_9H_{17}N_2OS$ 181.0977; Found 181.0977.

**Synthesis of 2b and 4a**

**Methyl 2-(hydroxy(phenyl)methyl)acrylate**[5]

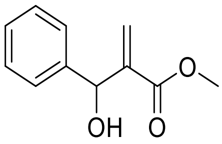

To a flask charged with DABCO (11.22 g, 100 mmol) was added dioxane (4 mL) and water (4 mL). Benzaldehyde (10.20 mL, 100 mmol) was added followed by methyl acrylate (27.02 mL, 300 mmol) and the resulting mixture was left to stir for 3 days. The layers were separated, and the aqueous layer was further extracted with DCM. The solvent was removed under reduced pressure and residue purified by flash column chromatography with 20% EtOAc in petrol to give methyl 2-(hydroxy(phenyl)methyl)acrylate (12.09, 63%) as a colorless oil.

**$^1$H NMR (400 MHz, CDCl3)** δ 7.41 – 7.32 (m, 4H, $CH_{Ar}$), 7.32 – 7.26 (m, 1H, $CH_{Ar}$), 6.34 (s, 1H, C=$CH_2$), 5.84 – 5.83 (m, 1H, C=$CH_2$), 5.57 (d, J = 5.5 Hz, 1H, CH), 3.73 (s, 3H, $CH_3$), 2.99 (d, J = 5.5 Hz, 1H, OH); **$^{13}$C NMR (101 MHz, CDCl3)** δ 166.9, 142.1, 141.4, 128.6, 128.0, 126.7, 126.4, 73.5, 52.1. Data is consistent with literature.

**2-Methylene-1-phenylpropane-1,3-diol**[6]

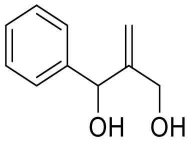

To a flame-dried flask charged with $LIiAlH_4$ (11.39 g, 300 mmol), was added $Et_2O$ (600 mL) and the resulting mixture was cooled to -78 ℃, A solution of methyl 2-(hydroxy(phenyl)-methyl)acrylate (21.42 g, 100 mmol) in Et2O (25 mL) was added dropwise and the resulting mixture was left to stir at room temperature for overnight. The mixture was cooled to 0 ℃ and water (100 mL) was added dropwise. The emulsion was diluted with EtOAc (500 mL) and conc. HCl was added dropwise until the emulsion dispersed and the layers were partitioned. The aqueous layer was further extracted with EtOAc and the organic components were combined. The solvent was removed under reduced pressure and residue purified by flash column chromatography with 40% EtOAc in petrol to give 2-methylene-1-phenylpropane-1,3-diol (7,97 g, 49%) as a colorless oil.

**$^1$H NMR (400 MHz, CDCl3)** δ 7.42 – 7.27 (m, 5H, $CH_{Ar}$), 5.38 (s, 1H, CH), 5.25 – 5.23 (m, 2H, $C=CH_2$), 4.17 (d, J = 13.0 Hz, 1H, $CH_2$), 4.07 (d, J = 13.0 Hz, 1H, $CH_2$), 2.56 (br, 1H, OH), 1.80 (br, 1H, OH); **$^{13}$C NMR (101 MHz, CDCl3)** δ 149.5, 141.9, 128.7, 128.0, 126.4, 113.6, 76.5, 64.3. Data is consistent with literature.

**2-(Iodomethyl)-1-phenylprop-2-wn-1-ol**

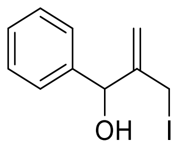


To a flask charged with triphenylphosphine (6.02 g, 21.64 mmol) and imidazole was added DCM (20 mL) and EtOAc (20 mL), followed by 2-methylene-1-phenylpropane-1,3-diol (3.23 g, 19.67 mmol). The resulting mixture was cooled to 0 ℃ and iodine (4.99 g, 19.67 mmol) was added portionwise. The reaction was left to stir at room temperature in the dark for 16 hrs, before dilution with DCM (20 mL) and the addition of water (40 mL). The layers were partitioned and the aqueous layer was further with DCM. The solvent was removed under reduced pressure and residue purified by flash column chromatography with 5% EtOAc in petrol to give 2-(iodomethyl)-1-phenylprop-2-wn-1-ol (1.90 g, 35%) as a colorless oil.

**FTIR** $\nu_{max}$ (thin film/cm$^{-1}$) 3369, 3028, 2874; **$^1$H NMR (400 MHz, CDCl3)** δ 7.41 – 7.30 (m, 5H, $CH_{Ar}$), 5.54 (s, 1H, CH), 5.45 (s, 1H, $C=CH_2$), 5.40 (s, 1H, $C=CH_2$), 4.00 (d, J = 9.5 Hz, 1H, $CH_2$), 3.65 (d, J = 9.5 Hz, 1H, $CH_2$), 2.14 (br, 1H, OH); **$^{13}$C NMR (101 MHz, CDCl3)** δ 148.0, 141.4, 128.8, 128.4, 127.0, 114.6, 74.8, 6.2; **MS (ESI$^+$)** 273 (100%, M+H$^+$); **HRMS (ESI-TOF)** m/z: $[M + H]^+$ Calcd for $C_{10}H_{12}IO$ 273.9849; Found 273.9842.

**5-Methylene-6-phenyl-1, 3-oxazinan-2-one**

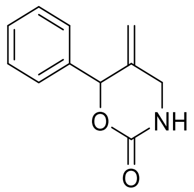


To a solution of 2-(Iodomethyl)-1-phenylprop-2-wn-1-ol (1.41 g, 5.13 mmol) in toluene (25 mL) was added AgOCN (1.15 g, 769 mmol) and the resulting mixture was left to stir at reflux (DrySyn block) overnight. Upon cooling, the resulting black precipitate was removed by

filtration, and washed cold DCM (20 mL). The solvent was removed under reduced pressure and residue purified by flash column chromatography with 70% EtOAc in petrol to give 5-methylene-6-phenyl-1, 3-oxazinan-2-one (533 mg, 55%) as a white solid.

**FTIR** $\nu_{max}$ (thin film/cm$^{-1}$) 1697, 1475, 1274, 1102; **$^1$H NMR (400 MHz, CDCl3)** δ 7.44 – 7.31 (m, 5H, $CH_{Ar}$), 6.19 (br,1H, NH), 5.77 (s, 1H, CH), 5.29 (s, 1H, C=$CH_2$), 5.05 (s, 1H, C=$CH_2$), 3.98 (d, J = 14.0 Hz, 1H, $CH_2$), 3.91 (d, J = 14.0 Hz, 1H, $CH_2$); **$^{13}$C NMR (101 MHz, CDCl3)** δ 154.7, 136.9, 136.5, 128.8, 128.6, 126.2, 115.3, 81.2, 44.6; **MS (ESI$^+$)** 190 (100%, M+H$^+$); **HRMS (ESI-TOF)** m/z: [M + H]$^+$ Calcd for $C_{11}H_{12}NO_2$ 190.0863; Found 190.0863.

### ***tert*-butyl 5-methylene-2-oxo-6-phenyl-1,3-oxazinane-3-carboxylate, 2a**

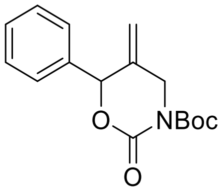


To a flask charged with 5-methylene-6-phenyl-1, 3-oxazinan-2-one (533 mg, 2.82 mmol) and DMAP (69 mg, 0.56 mmol) in DCM (15 mL) was added a solution of $Boc_2O$ (1.23 g, 5.63 mmol) in DCM (5 mL) and resulting mixture was left to stir for 3 hrs at room temperature. Upon completion, the solvent was removed under reduced pressure and residue purified by flash column chromatography with 20% EtOAc in petrol to give *tert*-butyl 5-methylene-2-oxo-6-phenyl-1,3-oxazinane-3-carboxylate (**2a**) (783 mg, 96%) as a white solid.

**FTIR** $\nu_{max}$ (thin film/cm$^{-1}$) 1794, 1727, 1264; **$^1$H NMR (400 MHz, CDCl3)** δ 7.44 – 7.31 (m, 5H, $CH_{Ar}$), 5.76 (s, 1H, CH), 5.30 – 5.38 (m, 1H, C=$CH_2$), 4.89 – 4.87 (m, 1H, C=$CH_2$), 4.44 (d, J = 15.0 Hz, 1H, $CH_2$), 4.26 (d, J = 15.0 Hz, 1H, $CH_2$); **$^{13}$C NMR (101 MHz, CDCl3)** δ 151.9, 150.4, 138.3, 135.7, 129.0, 128.8, 126.9, 114.8, 84.1, 81.3, 48.5, 28.1; **MS (ESI$^+$)** 312 (100%, M+H$^+$); **HRMS (ESI-TOF)** m/z: [M + H]$^+$ Calcd for $C_{16}H_{20}NO_4$ 312.1206; Found 312.1214.

**(E)-*N*-(5-benzylidene-3-isobutyl-2-oxopiperidin-3-yl)benzamide, 4b**

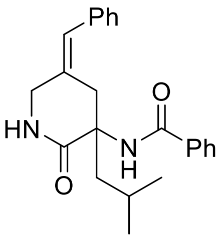


*Tert*-butyl 5-methylene-2-oxo-6-phenyl-1,3-oxazinane-3-carboxylate (**2a**) (62 mg, 0.2 mmol), ligand **L1** (5 mg, 0.015 mmol), {$\eta^3$-$C_3H_5PdCl$}$_2$ (2 mg, 0.005 mmol), $^t$BuOH (0.09 ml, 1.0 mmol) and DIPEA (0.03 ml, 0.4 mmol) were dissolved in dry 20% toluene in dioxane (2 mL) at 0 °C for 20 mins. Then methyl 2-(4-benzyl-5-oxo-4,5-dihydrooxazol-2-yl)benzoate (**1b**) (130 mg, 0.6 mmol) was added. The reaction mixture was stirred at 0 °C overnight. The solvent was removed under reduced pressure give the crude product which was dissolved in dry DCM (2 mL). TFA (1.2 mL, 15 mmol) was added and the reaction stirred at room temperature. After 1 hour, EtOAc was added and the reaction was quenched with sat. aq. $NaHCO_3$. The layers were separated, and the aqueous layer was further extracted with 50% EtOAc. The solvent was removed under reduced pressure and residue purified by flash column chromatography eluting with EtOAc to give (E)-*N*-(5-benzylidene-3-isobutyl-2-oxopiperidin-3-yl)benzamide (**4b**) (60 mg, 83%, >99:1 E/Z) as a white solid.

**FTIR** $\nu_{max}$ (thin film/cm$^{-1}$) 3265, 2956, 1643, 1602; **$^1$H NMR (400 MHz, $CDCl_3$)** δ $^1$H NMR (500 MHz, $CDCl_3$) δ 7.76 – 7.71 (m, 2H, $CH_{Ar}$), 7.56 (s, 1H, NH), 7.47 – 7.40 (m, 1H, $CH_{Ar}$), 7.40 – 7.33 (m, 2H, $CH_{Ar}$), 7.34 – 7.27 (m, 2H, $CH_{Ar}$), 7.26 – 7.17 (m, 3H, $CH_{Ar}$), 6.73 (s, 1H, $CH=CH_2$), 6.47 (s, 1H, NH), 4.19 (d, J = 15.5 Hz, 1H, $CH_2$), 4.12 (d, J = 15.5 Hz, 1H, $CH_2$), 4.05 (d, J = 15.5 Hz, 1H, $CH_2$), 2.74 (d, J = 15.5 Hz, 1H, $CH_2$), 2.25 – 2.15 (m, 1H, CH), 1.64 – 1.47 (m, 2H, $CH_2$), 0.79 (d, J = 6.0 Hz, 3H, $CH_3$), 0.75 (d, J = 6.0 Hz, 3H, $CH_3$); **$^{13}$C NMR (101 MHz, $CDCl_3$)** δ 174.0, 166.4, 136.0, 134.9, 131.5, 129.7, 128.8, 128.6, 128.5, 127.5, 127.2, 127.0, 59.2, 48.0, 42.1, 35.3, 24.4, 24.0, 23.8; **MS (ESI$^+$)** 363 (100%, M+H$^+$); **HRMS (ESI-TOF)** m/z: [M + H]$^+$ Calcd for $C_{23}H_{28}N_2O_2$ 363.2073; Found 363.2077.

**Synthesis of Alkyne Tagged Lactam**

***tert*-butyl (3-isobutyl-5-methylene-2-oxopiperidin-3-yl)carbamate**

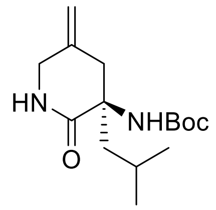


To a solution of 3-amino-3-isobutyl-5-methylenepiperidin-2-one (**16**) (183 mg, 1.0 mmol) dissolved in THF/$H_2O$ (1:2 v/v, 30 mL) was added $Boc_2O$ (426 mg, 2.0 mmol) and TEA (0.42 mL, 3.0 mmol). The reaction mixture was stirred at 0 °C for 2 hours then stirred at room temperature for 4 hours. The solvent was removed under reduced pressure and residue purified by flash column chromatography eluting with 30% EtOAc in petrol to give *tert*-butyl (3-isobutyl-5-methylene-2-oxopiperidin-3-yl)carbamate (259mg, 92%) as a colourless oil.

**FTIR** $\nu_{max}$ (thin film/cm$^{-1}$) 3229, 2957, 1717, 1672; **$^1$H NMR (400 MHz, $CDCl_3$)** δ 6.80 (s, 1H, NH), 5.80 (s, 1H, NH), 5.01 (s, 1H, C=$CH_2$), 4.97 (s, 1H, C=$CH_2$), 4.06 (d, J=15.0, 1H, $CH_2$), 3.92 (d, J = 15.0 Hz, 1H, $CH_2$), 3.24 (d, J = 14.0 Hz, 1H, $CH_2$), 2.76 (d, J = 14.0 Hz, 1H, $CH_2$), 2.03-1.98 (m, 1H, $CH_2$), 1.80-1.72 (m, 1H, CH), 1.50-1.42 (m, 10H, $CH_2$, $C(CH_3)_3$), 0.92 (d, J = 6.5 Hz, 3H), 0.86 (d, J = 6.5 Hz, 3H); **$^{13}$C NMR (101 MHz, $CDCl_3$)** δ 174.0, 154.5, 137.2, 112.8, 79.1, 57.7, 46.7, 42.8, 40.3, 28.4, 24.2, 24.0, 23.7; **MS (ESI$^+$)** 283 (100%, M+H$^+$); **HRMS (ESI-TOF)** m/z: [M + H]$^+$ Calcd for $C_{15}H_{27}N_2O_3$ 283.2022; Found 283.2027.

***tert*-butyl 2-(3-((*tert*-butoxycarbonyl)amino)-3-isobutyl-5-methylene-2-oxopiperidin-1-yl)acetate, 22**

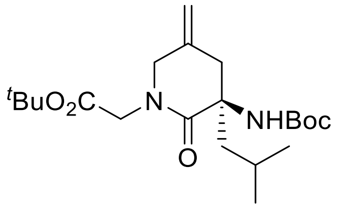


A mixture of (3-isobutyl-5-methylene-2-oxopiperidin-3-yl)carbamate (200 mg, 0.7 mmol) and lithium bis(trimethylsilyl)amide (1.0 M in hexanes) (1.4 mL, 1.4 mmol) was dissolved in dry THF at -78 ℃for 30 mins. Then *tert*-butyl bromoacetate (273 mg, 1.4 mmol) was added. The reaction mixture was stirred at room temperature 2 hours. The solvent was removed under

reduced pressure and residue purified by flash column chromatography eluting with 20% $Et_2O$ in petrol to give *tert*-butyl 2-(3-((*tert*-butoxycarbonyl)amino)-3-isobutyl-5-methylene-2-oxopiperidin-1-yl)acetate (**22**) (231 mg, 83%) as a colorless oil.

**FTIR** $\nu_{max}$ (thin film/cm$^{-1}$) 3408, 2978 1741, 1715, 1650; **$^1$H NMR (400 MHz, $CDCl_3$)** δ 5.91 (s, 1H, NH), 5.02 (s, 1H, C=$CH_2$), 4.98 (s, 1H, C=$CH_2$), 4.14 – 3.96 (m, 3H, $CH_2$), 3.88 (d, J = 17.0 Hz, 1H, $CH_2$), 3.36 (d, J = 14.0 Hz, 1H, $CH_2$), 2.76 (d, J = 14.0 Hz, 1H, $CH_2$), 2.13 – 1.95 (m, 1H, $CH_2$), 1.79 – 1.65 (m, 1H, CH), 1.55 – 1.48 (m, 1H, $CH_2$), 1.46 (s, 9H, $C(CH_3)_3$), 1.42 (s, 9H, $C(CH_3)_3$), 0.92 (d, *J* = 6.5 Hz, 3H, $CH_3$), 0.82 (d, *J* = 6.5 Hz, 3H, $CH_3$); **$^{13}$C NMR (101 MHz, $CDCl_3$)** δ 172.0, 167.6, 154.4, 136.7, 112.6, 82.0, 79.0, 57.9, 53.5, 49.5, 42.8, 40.4, 28.4, 28.1, 24.1, 24.0, 23.7; **MS (ESI$^+$)** 397 (100%, M+H$^+$); **HRMS (ESI-TOF)** m/z: [M + H]$^+$ Calcd for $C_{21}H_{37}N_2O_5$ 397.2702; Found 397.2704.

### ***tert*-butyl 2-(3-((tert-butoxycarbonyl)amino)-3-isobutyl-2,5-dioxopiperidin-1-yl)acetate, 23**

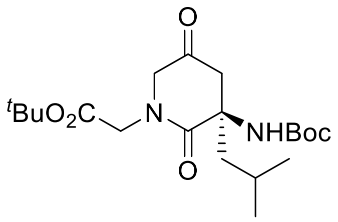


To a solution of *tert*-butyl 2-(3-((*tert*-butoxycarbonyl)amino)-3-isobutyl-5-methylene-2-oxopiperidin-1-yl)acetate (**22**) (198 mg, 0.5 mmol) dissolved in MeCN/DCM/$H_2O$ (1:1:2 v/v/v, 10 mL) was added $RuCl_3$ (16 mg, 0.15 mmol) and the reaction mixture was stirred at 0 °C for 30 mins. Then $NaIO_4$ (852 mg, 4 mmol) was added and the reaction mixture was stirred at room temperate. After 4 hours, DCM was added. The layers were separated, and the aqueous layer was further extracted with DCM. The solvent was removed under reduced pressure and residue purified by flash column chromatography eluting with 40% $Et_2O$ in petrol to give *tert*-butyl 2-(3-((*tert*-butoxycarbonyl)amino)-3-isobutyl-2,5-dioxopiperidin-1-yl)acetate (**23**) (150 mg, 75%) as a colorless oil.

**FTIR** $\nu_{max}$ (thin film/cm$^{-1}$) 3404, 2978, 1738, 1721, 1667; **$^1$H NMR (400 MHz, $CDCl_3$)** δ 5.78 (s, 1H, NH), 4.32 – 4.05 (m, 2H, $CH_2$), 3.83 (d, J = 19.0 Hz, 2H, $CH_2$), 3.49 (d, J = 16.0 Hz, 1H, $CH_2$), 3.12 (d, J = 16.0 Hz, 1H, $CH_2$), 2.24 – 2.06 (m, 1H, $CH_2$), 1.82 – 1.66 (m, 1H, CH), 1.45 (s, 10H, $CH_2$, $C(CH_3)_3$), 1.42 (s, 9H, $C(CH_3)_3$), 0.93 (d, J = 6.5 Hz, 3H, $CH_3$), 0.86 (d, J = 6.5 Hz, 3H, $CH_3$); **$^{13}$C NMR (101 MHz, $CDCl_3$)** δ 201.5, 170.9, 167.2, 154.2, 82.6, 79.7, 57.5, 49.1, 47.5, 42.7, 42.1,

28.3, 28.0, 24.0, 23.9, 23.8; **MS (ESI$^+$)** 421 (100%, M+Na$^+$); **HRMS (ESI-TOF)** m/z: [M + Na]$^+$ Calcd for $C_{20}H_{34}N_2O_6Na$ 421.2315; Found 421.2316.

***tert*-butyl 2-(3-((*tert*-butoxycarbonyl)amino)-3-isobutyl-2-oxo-5-(prop-2-yn-1-yloxy) pi peridin-1-yl)acetate, 24**

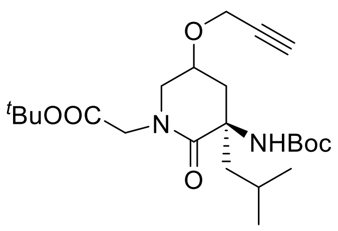


To a solution of *tert*-butyl 2-(3-((tert-butoxycarbonyl)amino)-3-isobutyl-2,5-dioxopiperidin-1-yl)acetate (**23**) (100 mg, 0.25 mmol) dissolved in dry THF (2.5 mL) was added K-selectride (1.0 M in THF) (0.5 mL, 0.5 mmol) at -78 °C. Then the reaction mixture was stirred at room temperature. After 30 mins, DCM was added and the reaction was quenched with brine. The layers were separated, and the aqueous layer was further extracted with DCM. The solvent was removed under reduced pressure to give the crude product. The crude product and propargyl bromide (150 mg, 1.25 mmol) were dissolved in toluene (2.5 mL). Tetrabutylammonium bisulfate (8.5 mg, 0.025 mmol) was dissolved in 50% NaOH solution (0.8 mL). The two solutions were combined and stirred at room temperature overnight. The layers were separated, and the aqueous layer was further extracted with DCM. The solvent was removed under reduced pressure and residue purified by flash column chromatography eluting with 20% EtOAc in petrol to give *tert*-butyl 2-(3-((*tert*-butoxycarbonyl)amino)-3-isobutyl-2-oxo-5-(prop-2-yn-1-yloxy) piperidin-1-yl)acetate (**24**) (100 mg, 92%, 5:1 dr) as a colorless oil.

Major diastereomer: **FTIR** $\nu_{max}$ (thin film/cm$^{-1}$) 3407, 3301, 2977, 1739, 1713, 1652; **$^1$H NMR (400 MHz, $CDCl_3$)** δ 5.50 (s, 1H, NH), 4.31 – 4.16 (m, 3H, $CH_2$, CH), 4.05 (d, $J$ = 17.0 Hz, 1H, $CH_2$), 3.96 (d, $J$ = 17.0 Hz, 1H, $CH_2$), 3.61 (dd, $J$ = 12.0, 5.5 Hz, 1H, $CH_2$), 3.51 (dd, $J$ = 12.0, 7.0 Hz, 1H, $CH_2$), 2.74 – 2.57 (m, 1H, $CH_2$), 2.51 – 2.34 (m, 2H, $CH_2$, CH), 2.14 – 1.98 (m, 1H, $CH_2$), 1.88 – 1.70 (m, 2H, $CH_2$, CH), 1.48 (s, 9H, $C(CH_3)_3$), 1.44 (s, 9H, $C(CH_3)_3$), 0.96 – 0.87 (m, 6H, $2CH_3$); **$^{13}$C NMR (101 MHz, $CDCl_3$)** δ 172.0, 167.8, 154.5, 82.0, 79.5, 79.2, 74.6, 69.4, 58.1, 56.0, 52.3, 50.3, 45.4, 36.4, 28.4, 28.0, 24.3, 24.2, 23.9; **MS (ESI$^+$)** 429 (100%, M+H$^+$); **HRMS (ESI-TOF)** m/z: [M + H]$^+$ Calcd for $C_{23}H_{39}N_2O_6$ 439.2808; Found 439.2809.

***tert*-butyl 2-((7R)-7-((tert-butoxycarbonyl)amino)-7-isobutyl-6-oxo-1-oxa-5-azaspiro[2.5]-octan-5-yl)acetate, 25**

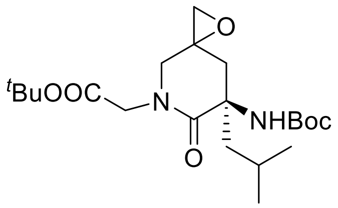


Oxone (1.07 g, 3.5 mmol) and $NaHCO_3$ (1.57 g, 18.7 mmol) were dissolved in water (3 mL) and stirred for 10 mins. To this solution, *tert*-butyl 2-(3-((*tert*-butoxycarbonyl)amino)-3-isobutyl-5-methylene-2-oxopiperidin-1-yl)acetate (**22**) (85 mg, 0.22 mmol) in acetone (3 mL) was added and the reaction was left to stir at room temperature 3 days. The mixture was diluted with water and extracted with EtOAc. Purification by flash column chromatography eluting with a gradient of 40% $Et_2O$ in petrol to give *tert*-butyl 2-((7R)-7-((tert-butoxycarbonyl)amino)-7-isobutyl-6-oxo-1-oxa-5-azaspiro[2.5]-octan-5-yl)acetate (**25**) the major diastereomer (73 mg, 61%) as a colourless oil and the minor diastereomer (37 mg, 31%) as a colourless oil.

Major diastereomer: **FTIR** $\nu_{max}$ (thin film/cm$^{-1}$) 3408, 2977, 1740, 1716, 1656; **$^1$H NMR (400 MHz, $CDCl_3$)** δ 5.94 (s, 1H,NH), 4.22 (d, *J* = 17.0 Hz, 1H, $CH_2$), 3.76 (d, *J* = 17.0 Hz, 1H, $CH_2$), 3.59 – 3.42 (m, 2H, $CH_2$), 2.88 (s, 2H, $CH_2$), 2.63 (d, *J* = 13.5 Hz, 1H, $CH_2$), 2.52 (d, *J* = 13.5 Hz, 1H, $CH_2$), 2.29 – 2.19 (m, 1H, $CH_2$), 1.82 – 1.67 (m, 2H, $CH_2$), 1.47 (s, 9H, $C(CH_3)_3$), 1.42 (s, 9H, $C(CH_3)_3$), 0.95 (d, *J* = 6.5 Hz, 3H, $CH_3$), 0.87 (d, *J* = 6.5 Hz, 3H, $CH_3$); **$^{13}$C NMR (101 MHz, $CDCl_3$)** δ 171.5, 167.5, 154.3, 82.3, 65.8, 58.0, 55.9, 53.3, 52.8, 49.7, 43.1, 38.8, 28.4, 28.0, 24.1, 24.1, 23.9; **MS (ESI$^+$)** 413 (100%, M+H$^+$); **HRMS (ESI-TOF)** m/z: [M + H]$^+$ Calcd for $C_{21}H_{37}N_2O_6$ 413.2652; Found 413.2659.

***tert*-butyl 2-((3R)-3-((tert-butoxycarbonyl)amino)-3-isobutyl-5-methyl-2-oxopiperidin-1-yl)-acetate, 26**

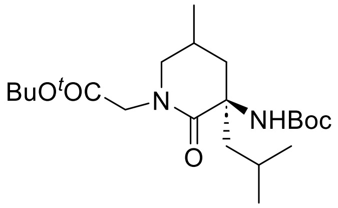

To a solution of *tert*-butyl 2-(3-((tert-butoxycarbonyl)amino)-3-isobutyl-5-methylene-2-oxopiperidin-1-yl)acetate (22) (80 mg, 0.2 mmol) dissolved in degas MeOH/EtOAc (3:1, 2 mL) was added Pd/C (10 wt%, 25 mg, 2 mmol) and the reaction stirred at room temperature overnight. The solvent was removed under reduced pressure and residue purified by flash column chromatography eluting with 20% $Et_2O$ in petrol to give *tert*-butyl 2-((3R)-3-((tert-butoxycarbonyl)amino)-3-isobutyl-5-methyl-2-oxopiperidin-1-yl)-acetate (**26**) the major diastereomer (59 mg, 74%) as a colourless oil and the minor diastereomer (20 mg, 25%) as a colourless oil.

Major diastereomer: **FTIR** $\nu_{max}$ (thin film/cm$^{-1}$) 2961, 2253, 1708, 1646; **$^1$H NMR (400 MHz, $CDCl_3$)** δ $^1$H NMR (400 MHz, $CDCl_3$) δ 5.47 (br, 1H, NH), 4.14 (d, J = 17.0 Hz, 1H, $CH_2$), 3.71 (d, J = 17.0 Hz, 1H, $CH_2$), 3.36 – 3.20 (m, 1H, $CH_2$), 3.17 – 2.95 (m, 1H, $CH_2$), 2.57 – 2.37 (m, 1H, $CH_2$), 2.32 – 2.19 (m, 1H, $CH_2$), 2.09 – 1.97(m, 2H, CH, $CH_2$), 1.91 – 1.78 (m, 1H, CH), 1.62 (dd, J = 14.5, 6.0 Hz, 1H, $CH_2$), 1.46 (s, 9H, $C(CH_3)_3$), 1.42 (s, 9H, $C(CH_3)_3$), 0.99 (d, J = 6.5 Hz, 3H, $CH_3$), 0.95 (d, J = 6.5 Hz, 3H, $CH_3$), 0.90 (d, J = 6.5 Hz, 3H, $CH_3$); **$^{13}$C NMR (101 MHz, $CDCl_3$)** δ 172.6, 168.1, 154.8, 81.7, 79.1, 58.3, 56.2, 49.9, 45.4, 39.6, 28.4, 28.1, 25.3, 24.5, 24.3, 23.7, 19.0; **MS (ESI$^+$)** 399 (100%, M+H$^+$); **HRMS (ESI-TOF)** m/z: $[M + H]^+$ Calcd for $C_{21}H_{39}N_2O_5$ 399.2859; Found 399.2857.

**Synthesis of MIF-1 analogue**

***tert*-butyl (*S*)-2-(((R)-3-isobutyl-5-methylene-2-oxopiperidin-3-yl)carbamoyl)pyrrolidine-1-carboxylate**

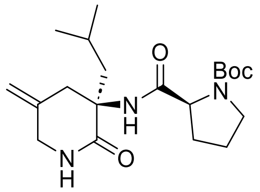


L-Proline-Boc (431 mg, 2.0 mmol), HOAT (272 mg, 2.0 mmol), DIPEA (0.57 mL, 3.3 mmol), EDC.HCl (383 mg, 2.0 mmol) were dissolved in dry DCM (20 mL) at room temperature for 20 mins. Then (*R*)-3-amino-3-isobutyl-5-methylenepiperidin-2-one (**16**) (300 mg, 1.64 mmol) was added and the reaction mixture was stirred at room temperature overnight. The solvent was removed under reduced pressure and residue purified by flash column chromatography eluting with 50% EtOAc in petrol to give *tert*-butyl (*S*)-2-(((*R*)-3-isobutyl-5-methylene-2-oxopiperidin-3-yl)carbamoyl)-pyrrolidine-1-carboxylate (592 mg, 95%, 90:10 dr) as a colourless oil and a mixture of rotamers (1:1).

**FTIR** $\nu_{max}$ (thin film/cm$^{-1}$) 3285, 2956, 1661; **$^1$H NMR (400 MHz, $CDCl_3$)** δ 7.75 (br, 0.5H, NH), 7.28 (br, 0.5H, NH), 6.79 (br, 0.5H, NH), 6.46 (br, 0.5H, NH), 5.03 (br, 1H, C=$CH_2$), 5.00 (s, 1H, C=$CH_2$), 4.38 – 4.12 (m, 1H, $CH_2$), 4.07 (d, J = 15.5 Hz, 1H, $CH_2$), 3.94 (br, 1H, CH), 3.56 – 3.21 (m, 3H, $CH_2$), 2.81 – 2.54 (m, 1H, $CH_2$), 2.32 – 2.00 (m, 3H, $CH_2$), 1.93 – 1.82 (m, 2H, $CH_2$, CH), 1.72 (br, 1H, $CH_2$) 1.56 – 1.37 (m, 10H, $CH_2$, $C(CH_3)_3$), 0.89 (d, J = 6.5 Hz, 3H, $CH_3$), 0.84 (d, J = 6.5 Hz, 3H, $CH_3$); **$^{13}$C NMR (101 MHz, $CDCl_3$)** δ 173.5, 171.6, 171.3, 137.2, 112.9, 80.1, 61.5, 60.4, 58.3, 46.9, 46.6, 46.3, 42.8, 42.5, 39.6, 28.4, 24.3, 23.9, 23.7, 23.4; **MS (ESI$^+$)** 380 (100%, M+H$^+$); **HRMS (ESI-TOF)** m/z: [M + H]$^+$ Calcd for $C_{20}H_{34}N_3O_4$ 380.2549; Found 380.2555.

***tert*-butyl (S)-2-(((R)-1-(2-amino-2-oxoethyl)-3-isobutyl-5-methylene-2-oxopiperidin-3-yl)carbamoyl)pyrrolidine-1-carboxylate, 27**

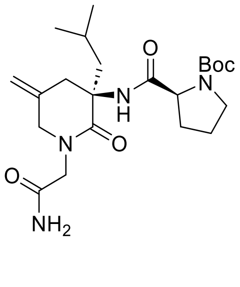

A mixture of (*S*)-2-(((*R*)-3-isobutyl-5-methylene-2-oxopiperidin-3-yl)carbamoyl)-pyrrolidine-1-carboxylate (543 mg, 1.43 mmol) and lithium bis(trimethylsilyl)amide (1.0 M in hexanes) (2 mL, 2.0 mmol) was dissolved in dry THF at -78 ℃ for 30 mins. Then $\alpha$-iodoacetamide (370 mg, 2.0 mmol) was added. The reaction mixture was stirred at room temperature overnight. The solvent was removed under reduced pressure and residue purified by flash column chromatography eluting with 90% EtOAc in petrol to give *tert*-butyl (S)-2-(((R)-1-(2-amino-2-oxoethyl)-3-isobutyl-5-methylene-2-oxopiperidin-3-yl)carbamoyl)pyrrolidine-1-carboxylate (**27**) (592 mg, 95%, 90:10 dr) as a white solid and a mixture of rotamers (1:1).

Crystallization Method: A sample of product (20 mg) was dissolved in acetone (1 mL) and the sample filterered to remove residual solid. Hexane (9 mL) was added and the sample transferred to a glass tube. Slow evaporation of the solvent gave crystals of **27** for X-ray analysis.

**m.p.:** 206 – 209 ℃ (After recrystallisation from acetone and hexane). **FTIR** $\nu_{max}$ (thin film/cm$^{-1}$) 3375, 2957, 1643; **$^{1}$H NMR (400 MHz, $CDCl_3$)** δ 7.72 (s, 0.5H, NH), 7.44 (s, 1H, NH), 6.78 (s, 0.5H, NH), 5.74 (s, 1H, NH), 5.28 (s, 1H, CH), 5.04 (s, 1H, C=$CH_2$), 4.97 (s, 1H, C=$CH_2$), 4.83 (d, J = 16.5 Hz, 1H, $CH_2$), 4.25-4.14 (m, 2H, CH, $CH_2$), 3.74 (d, J = 13.5 Hz, 1H, $CH_2$), 3.37 (br, 2H, $CH_2$), 3.25 (d, J = 16.5 Hz, 1H, $CH_2$), 3.11 (d, J = 12.6 Hz, 1H, $CH_2$), 2.57 (br, 1H, $CH_2$), 1.96 – 1.75 (m, 4H, CH, $CH_2$), 1.71 – 1.52 (m, 2H, $CH_2$), 1.45 (s, 9H, $C(CH_3)_3$), 0.95-0.91 (m, 6H, $2CH_3$); **$^{13}$C NMR (101 MHz, $CDCl_3$)** δ 171.2, 171.0, 170.7, 136.4, 113.8, 80.6, 59.6, 59.0, 54.6, 54.5, 53.5, 51.5, 47.1, 44.8, 39.2, 28.3, 24.6, 24.2, 24.1, 23.7; **MS (ESI$^+$)** 437 (100%, M+H$^+$); **HRMS (ESI-TOF)** m/z: $[M + H]^+$ Calcd for $C_{22}H_{37}N_4O_5$ 437.2764; Found 437.2765.

**(*R*)-2-(((*R*)-1-(2-Amino-2-oxoethyl)-3-isobutyl-5-methylene-2-oxopiperidin-3-yl)carbamoyl)-pyrrolidin-1-ium, 28**

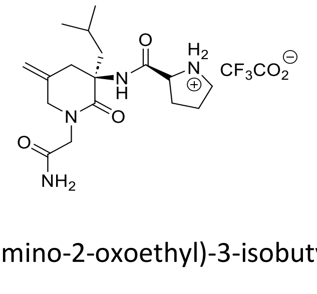


The *tert*-butyl (S)-2-(((R)-1-(2-amino-2-oxoethyl)-3-isobutyl-5-methylene-2-oxopiperidin-3-yl)carbamoyl)pyrrolidine-1-carboxylate (**27**) was dissolved in TFA (8 mL, 105 mmol) and the reaction mixture was stirred at room temperature for 1 hour. TFA was removed under

reduced pressure to furnish (*R*)-2-(((*R*)-1-(2-amino-2-oxoethyl)-3-isobutyl-5-methylene-2-oxopiperidin-3-yl)carbamoyl) pyrrolidin-1-ium (**28**) (610 mg, 100%, 90:10 dr) as a yellow oil.

**FTIR** $\nu_{max}$ (thin film/cm$^{-1}$) 3285, 2960, 1666, 1637; **$^{1}$H NMR (400 MHz, CDCl$_3$)** 9.71 (br, 1H,NH), 7.87 (br, 1H, NH), 7.52 (br, 1H, NH), 7.44 (br, 1H, NH), 7.11 (br, 1H, NH), 5.13 (s, 1H, C=CH$_2$), 5.07 (s, 1H, C=CH$_2$), 4.79 (d, J = 16.5 Hz, 1H, CH$_2$), 4.63 (br, 1H, CH$_2$), 4.25 (d, J = 14.0 Hz, 1H, CH$_2$), 3.84 (d, J = 14.0 Hz, 1H, CH$_2$), 3.44 – 3.34 (m, 3H, CH, CH$_2$), 3.18 (d, J = 13.5 Hz, 1H, CH$_2$), 2.55 (d, J = 13.5 Hz, 1H, CH$_2$), 2.45 (br, 1H, CH$_2$), 2.25 – 1.92 (m, 3H, CH, CH$_2$), 1.90 – 1.55 (m, 3H, CH$_2$), 0.96 (d, J = 6.5 Hz, 3H, CH$_3$), 0.88 (d, J = 6.5 Hz, 3H, CH$_2$); **$^{13}$C NMR (101 MHz, CDCl$_3$)** 172.4, 170.1, 168.7, 161.17 (q, J = 47.0 Hz), 135.3, 115.8 (q, J = 290.0 Hz), 114.7, 59.8, 59.8, 54.8, 51.1, 46.7, 45.1, 40.0, 29.8, 24.1, 24.0, 23.8, 23.5; **$^{19}$F NMR (377 MHz, CDCl$_3$)** δ -76.9; **MS (ESI$^+$)** 337 (100%, M+H$^+$); **HRMS (ESI-TOF)** m/z: [M + H]$^+$ Calcd for $C_{17}H_{29}N_4O_3$ 337.2225; Found 337.2222.

**Gram scale synthesis**

**Methyl (*R*)-2-((3-isobutyl-5-methylene-2-oxopiperidin-3-yl)carbamoyl)benzoate**

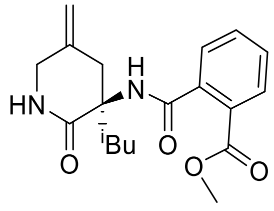


*N*-Boc-5-Methylenecyclohexacarbamate (**2**) (2.1 g, 10 mmol), (*R*,*R*)-ANDEN-phenyl Trost ligand (600 mg, 0.75 mmol), {$\eta^3$-$C_3H_5PdCl$}$_2$ (100 mg, 0.25 mmol), $^t$BuOH (4.5 ml, 50 mmol) and DIPEA (1.5 ml, 20 mmol) were dissolved in dry 20% toluene in dioxane (100 mL) at 0 °C for 20 mins. Then methyl 2-(4-isobutyl-5-oxo-4,5-dihydrooxazol-2-yl)benzoate (**1l**) (8.2 g, 30 mmol) was added. The reaction mixture was stirred at 0 °C overnight. The solvent was removed under reduced pressure give the crude product which was dissolved in dry DCM (50 mL). TFA (75 mL, 3.8 mol) was added and the reaction stirred at room temperature. After 1 hour, EtOAc was added and the reaction was quenched with sat. aq. NaHCO$_3$. The layers were separated, and the aqueous layer was further extracted with EtOAc. The solvent was removed under reduced pressure and residue purified by flash column chromatography eluting with EtOAc to give methyl (*R*)-2-((3-isobutyl-5-methylene-2-oxopiperidin-3-yl)carbamoyl)benzoate (3.1 g, 90%) as a colorless oil.

## NMR Spectra

## 1f

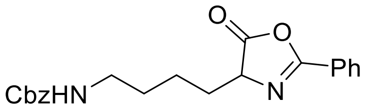

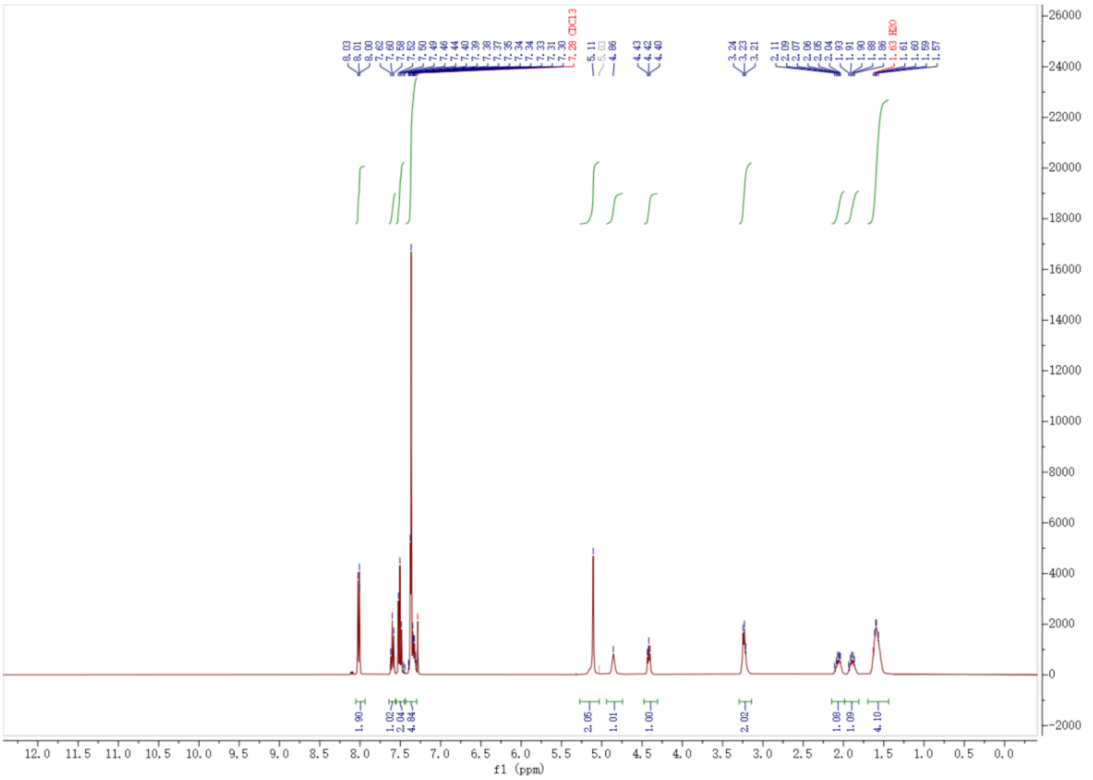

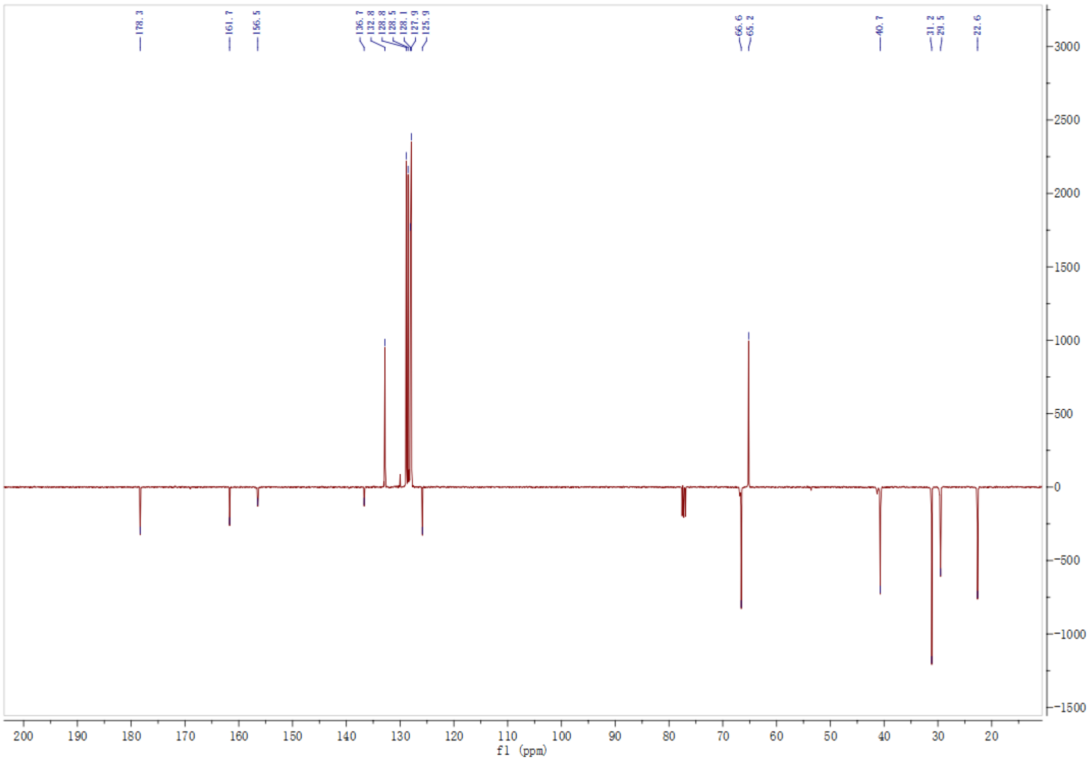

**1m**

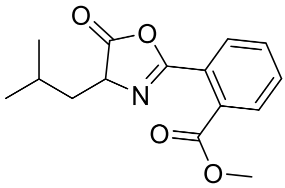
O
O
N
O
O

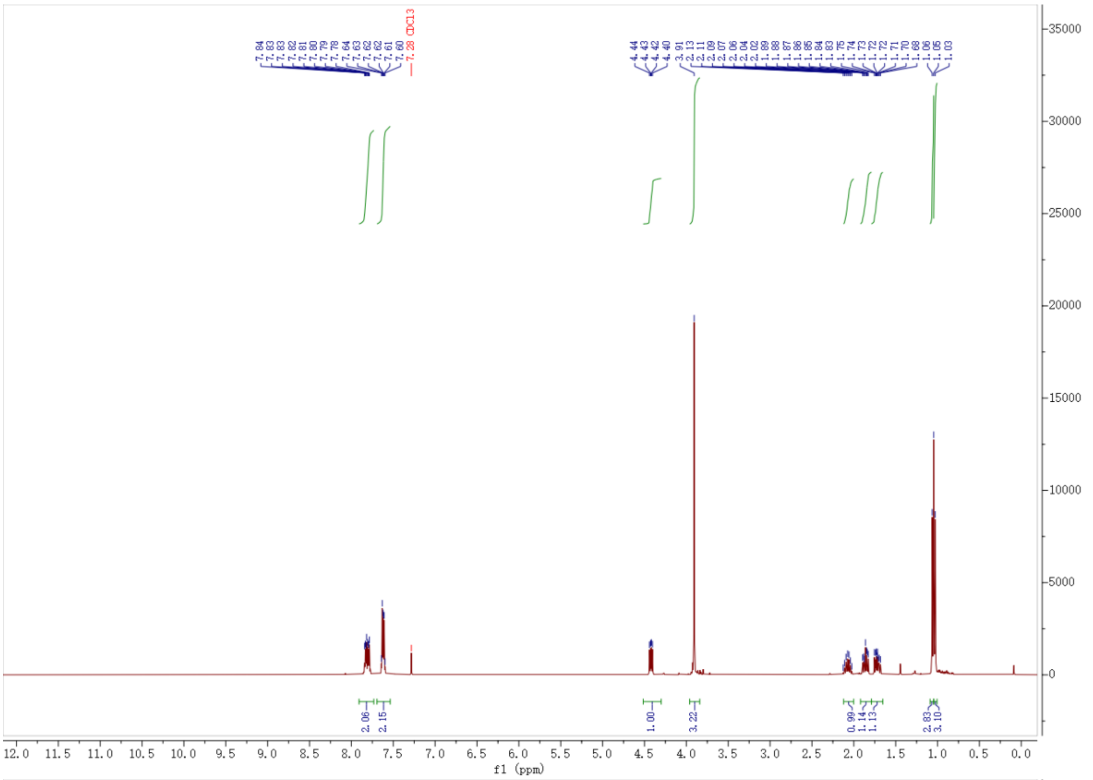
7.84
7.83
7.83
7.82
7.81
7.80
7.79
7.78
7.64
7.63
7.62
7.62
7.61
7.60
7.28 CDCl3
4.44
4.43
4.42
4.40
3.91
2.13
2.11
2.09
2.07
2.06
2.04
2.02
1.89
1.88
1.87
1.86
1.85
1.84
1.83
1.76
1.74
1.73
1.72
1.71
1.70
1.68
1.06
1.05
1.03
2.06
2.15
1.00
3.22
0.99
1.14
1.13
2.83
3.10
f1 (ppm)

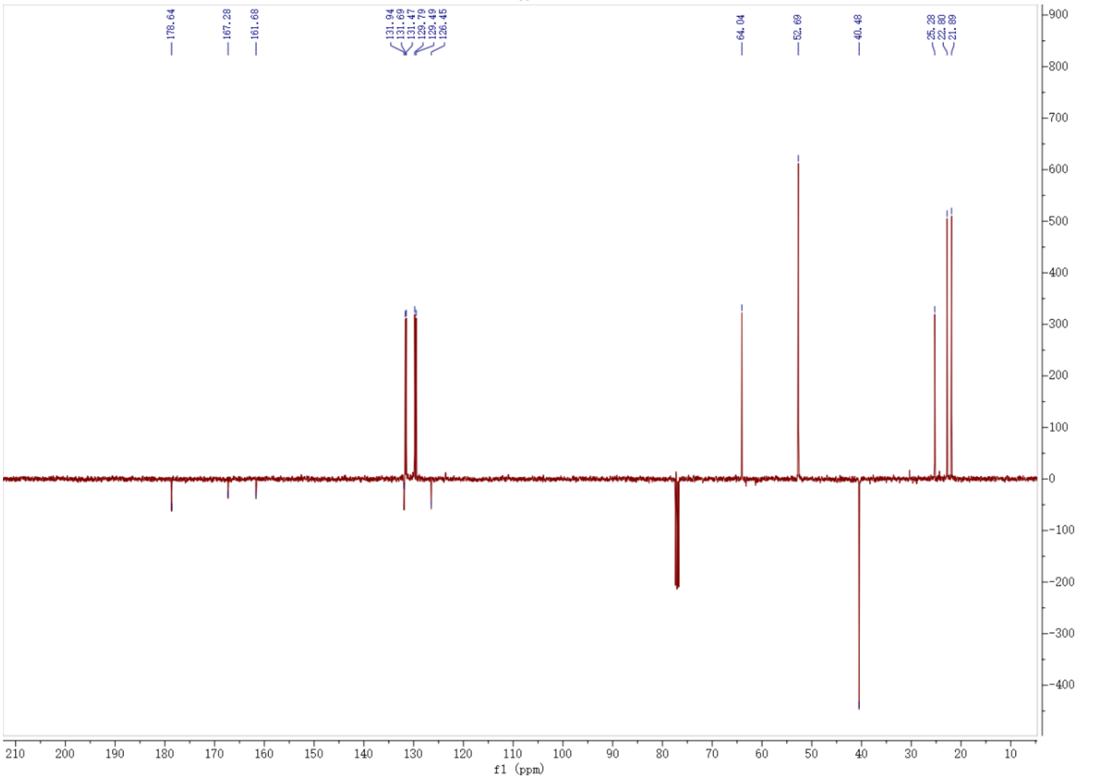
178.64
167.28
161.68
131.94
131.69
131.47
129.79
129.49
126.45
64.04
52.69
40.48
25.28
22.80
21.89
f1 (ppm)

**1n**

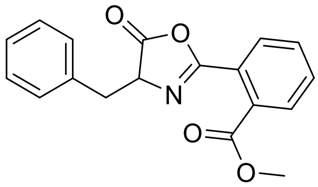
O
O
N
O
O

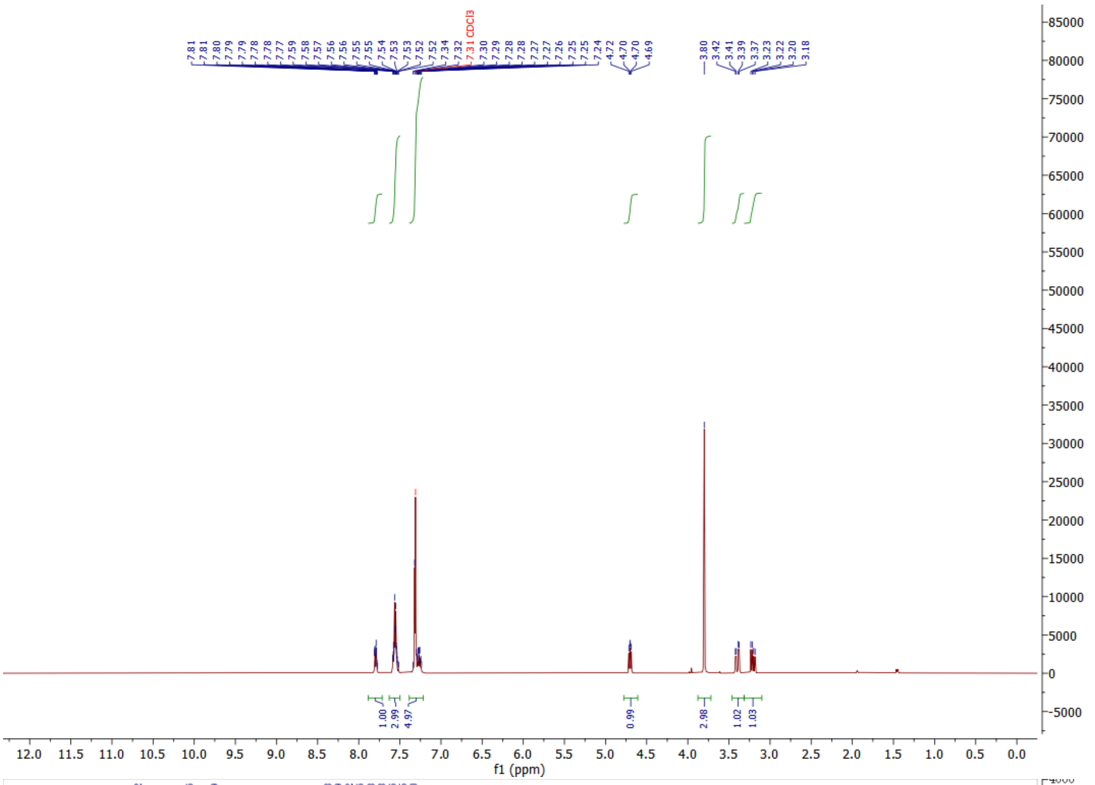
7.31 CDCl3
3.80
1.00
2.99
4.97
0.99
2.98
1.02
1.03
f1 (ppm)

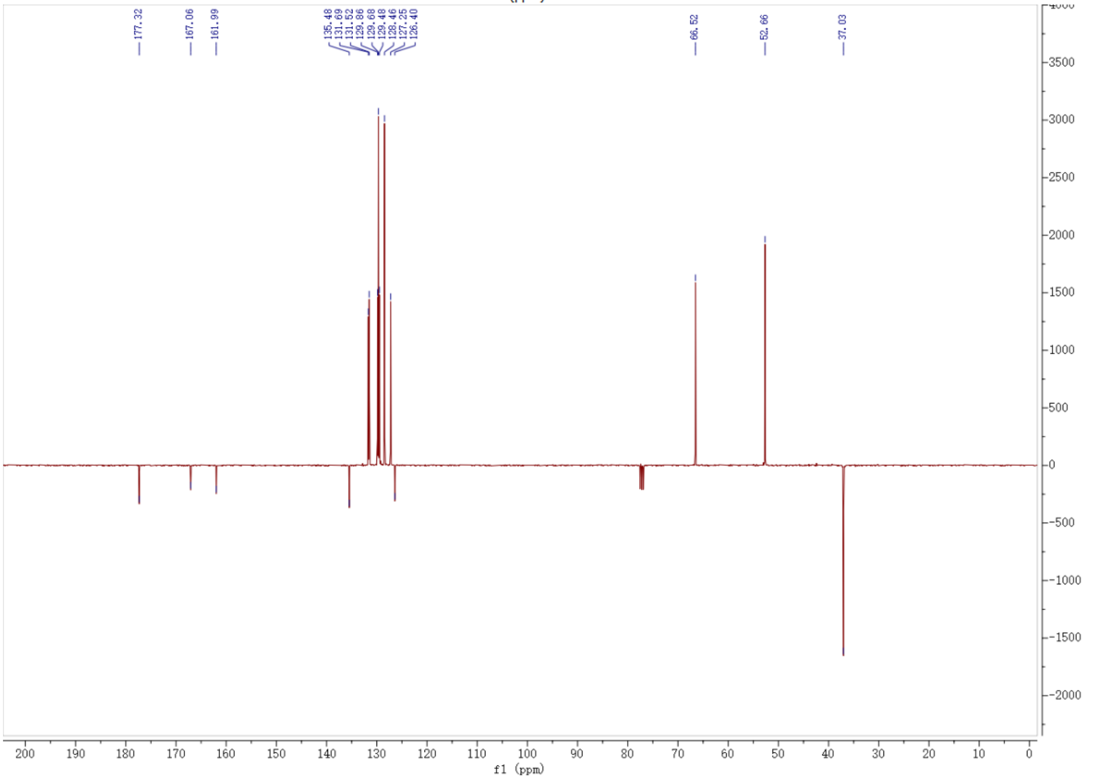
177.32
167.06
161.99
135.48
131.69
131.52
129.86
129.68
129.48
128.46
127.25
126.40
66.52
52.66
37.03
f1 (ppm)

**1o**

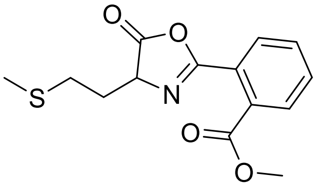


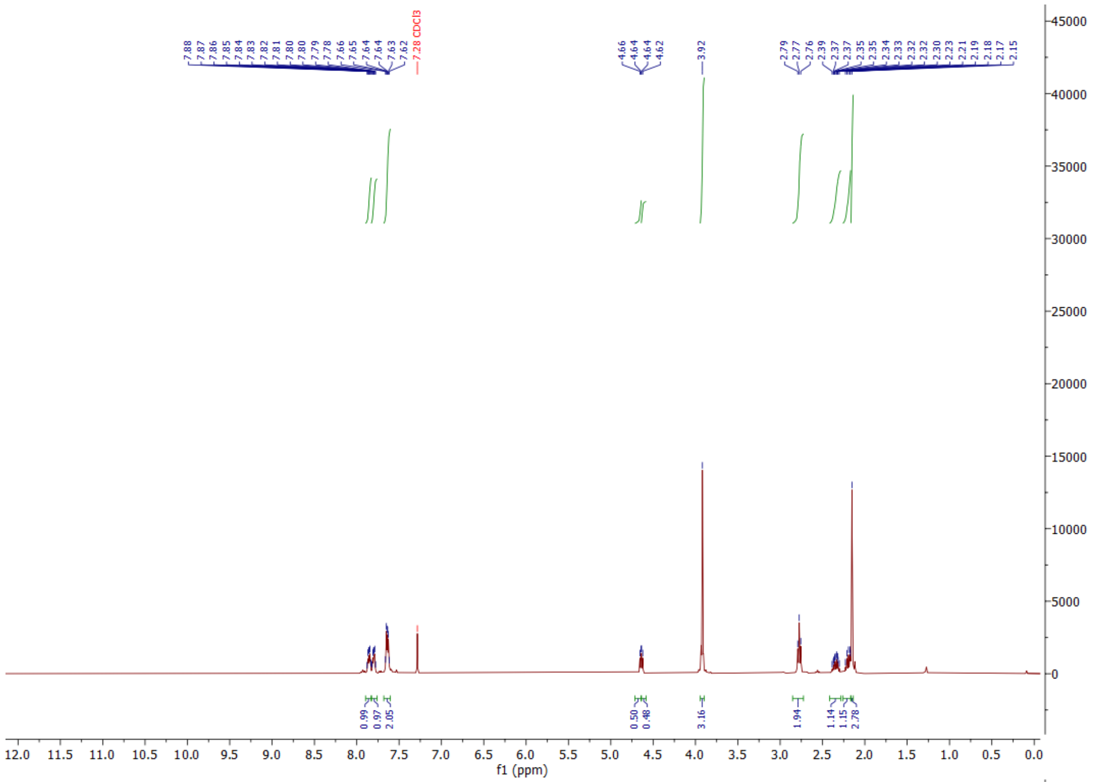


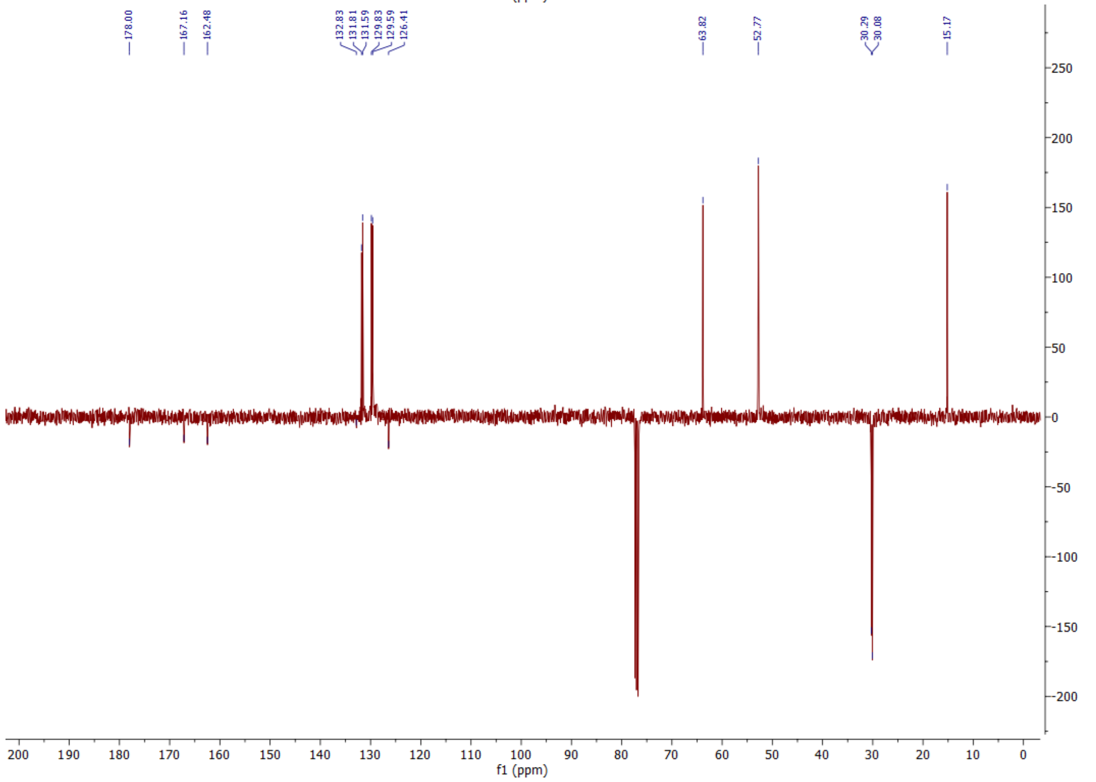

**1p**

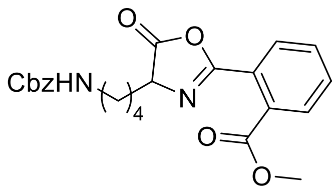


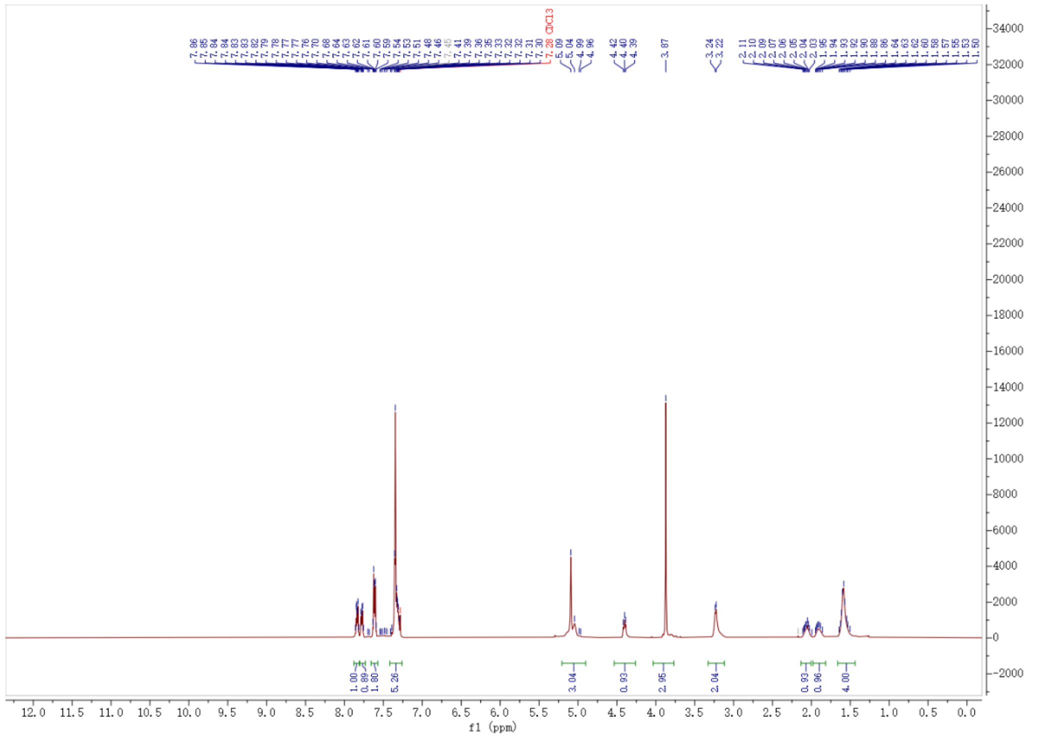

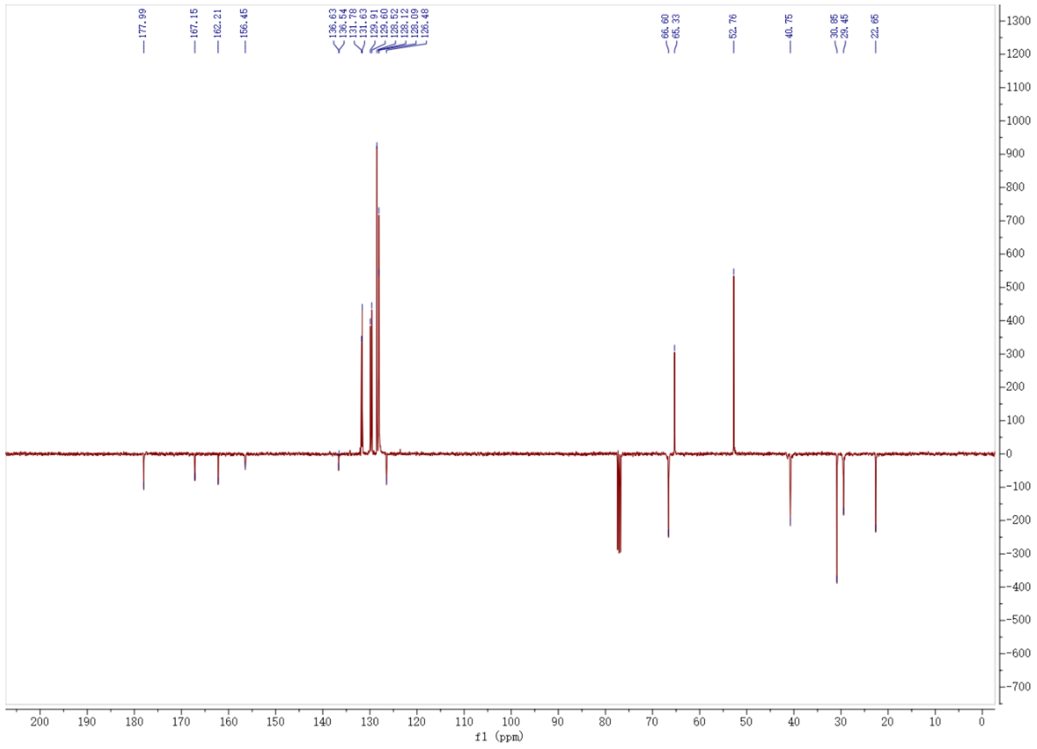

**1q**

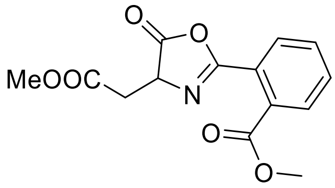

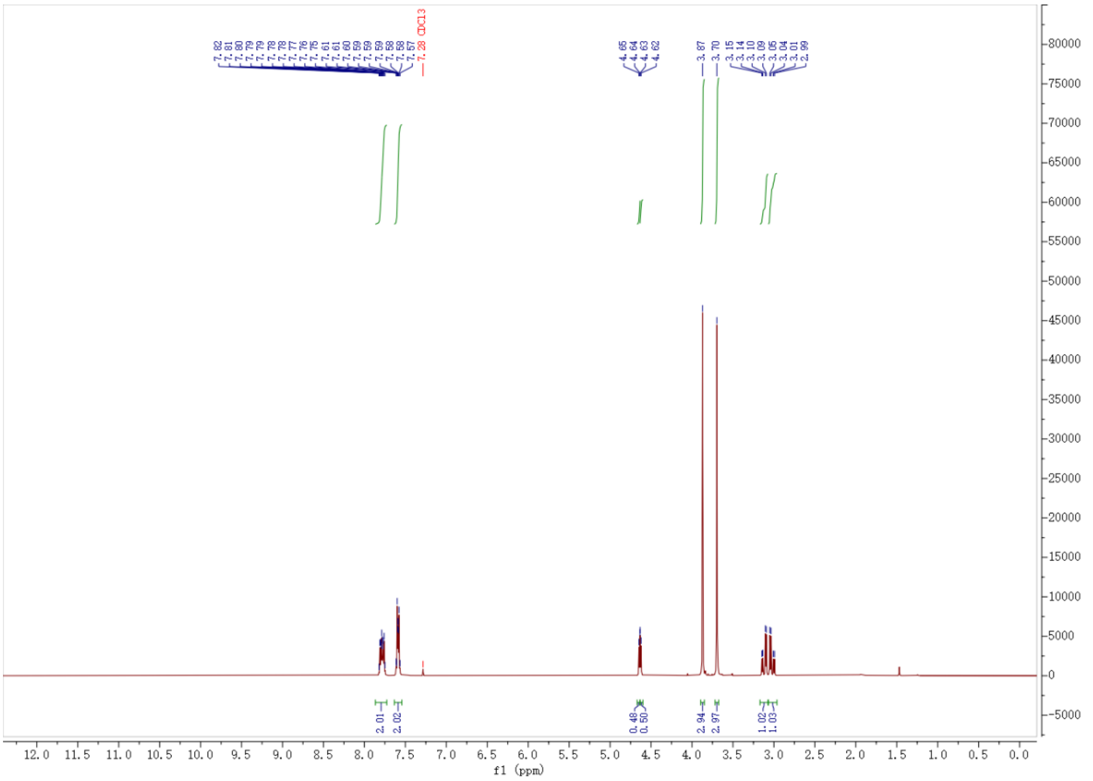

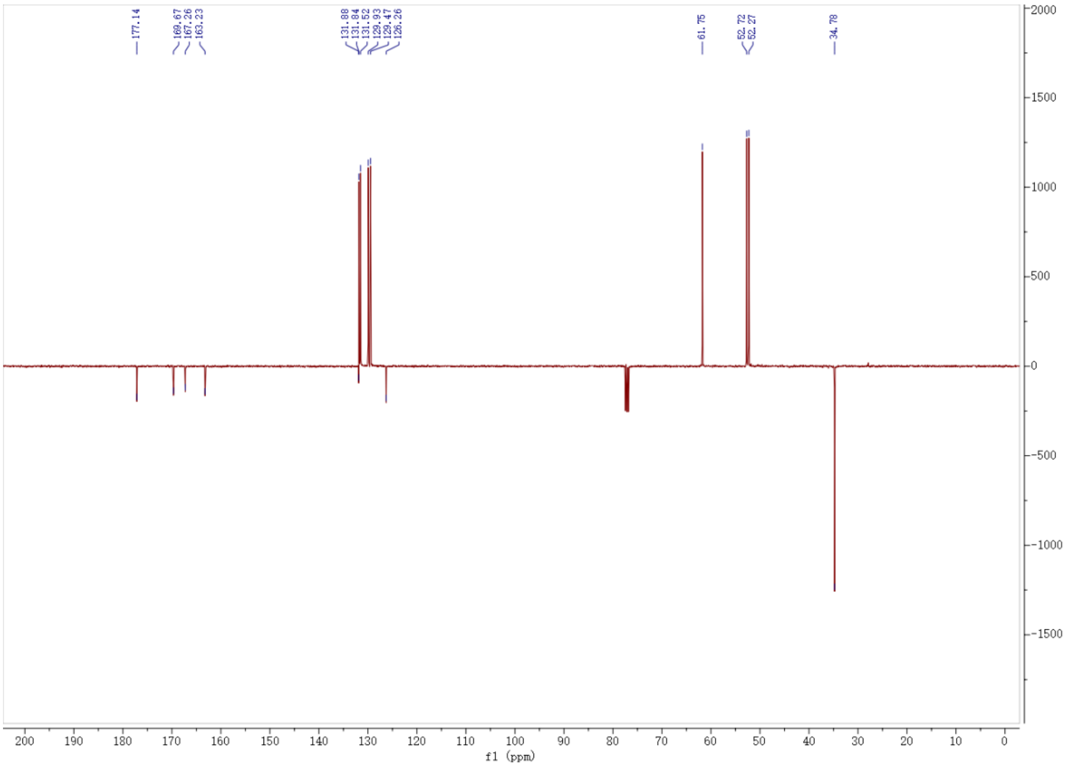

**1r**

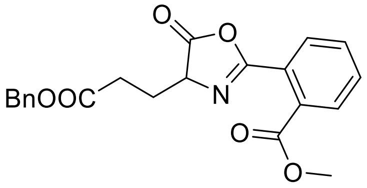


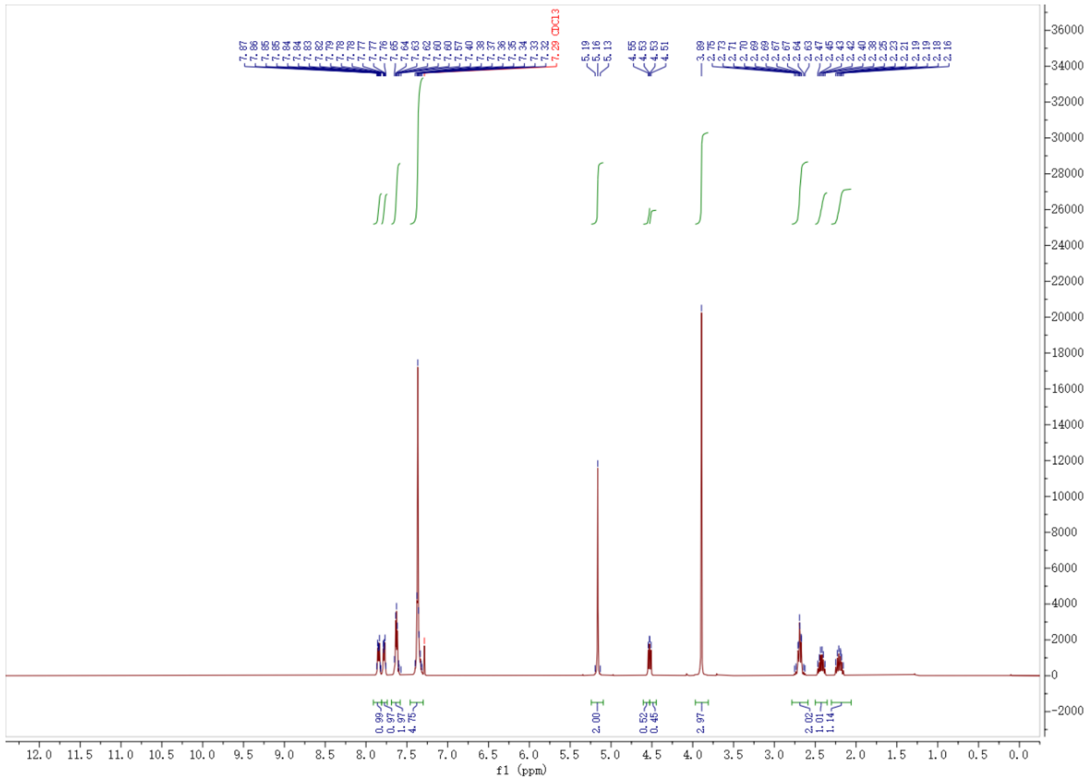

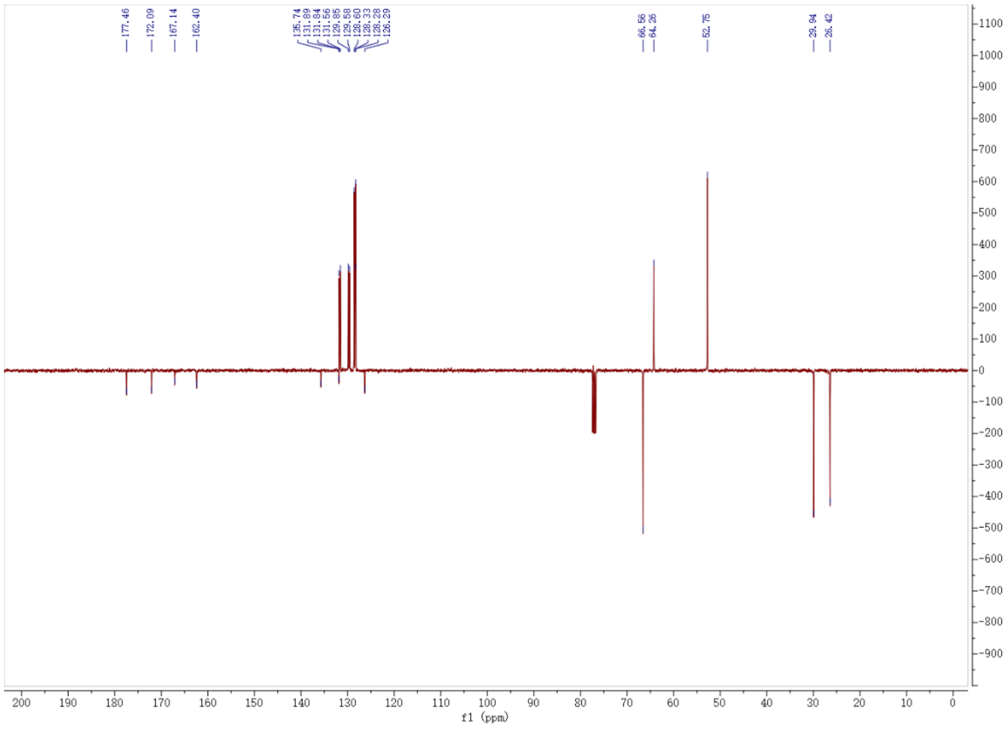

**2a**

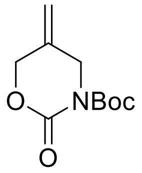
O
NBoc
O

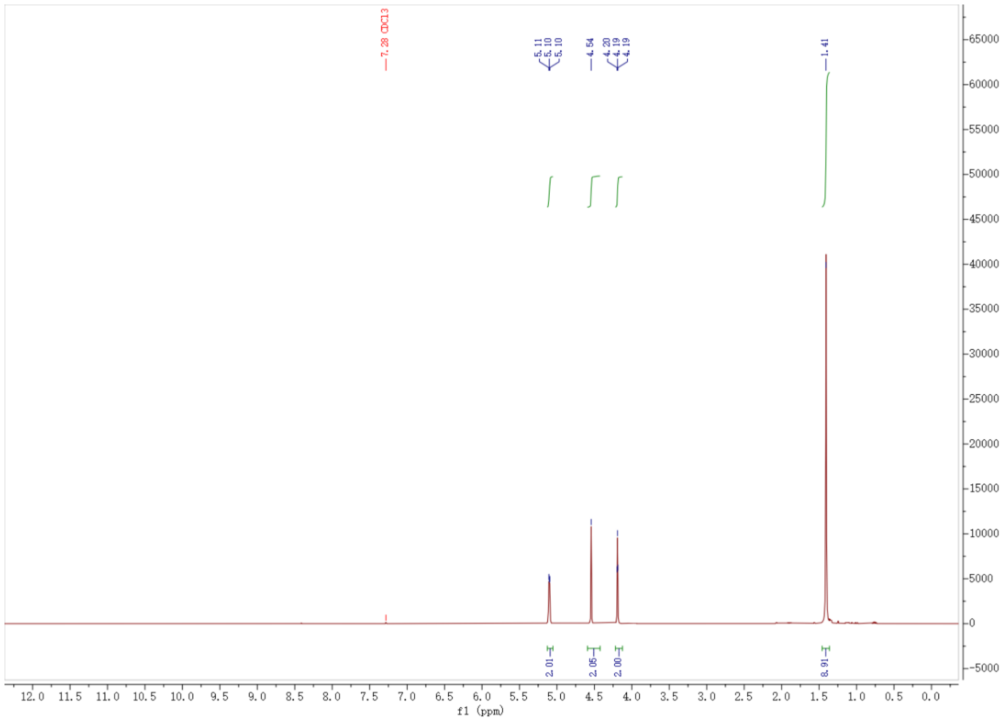
7.28 CDCl3
5.11
5.10
5.10
4.54
4.20
4.19
4.19
1.41
2.01
2.05
2.00
8.91
f1 (ppm)

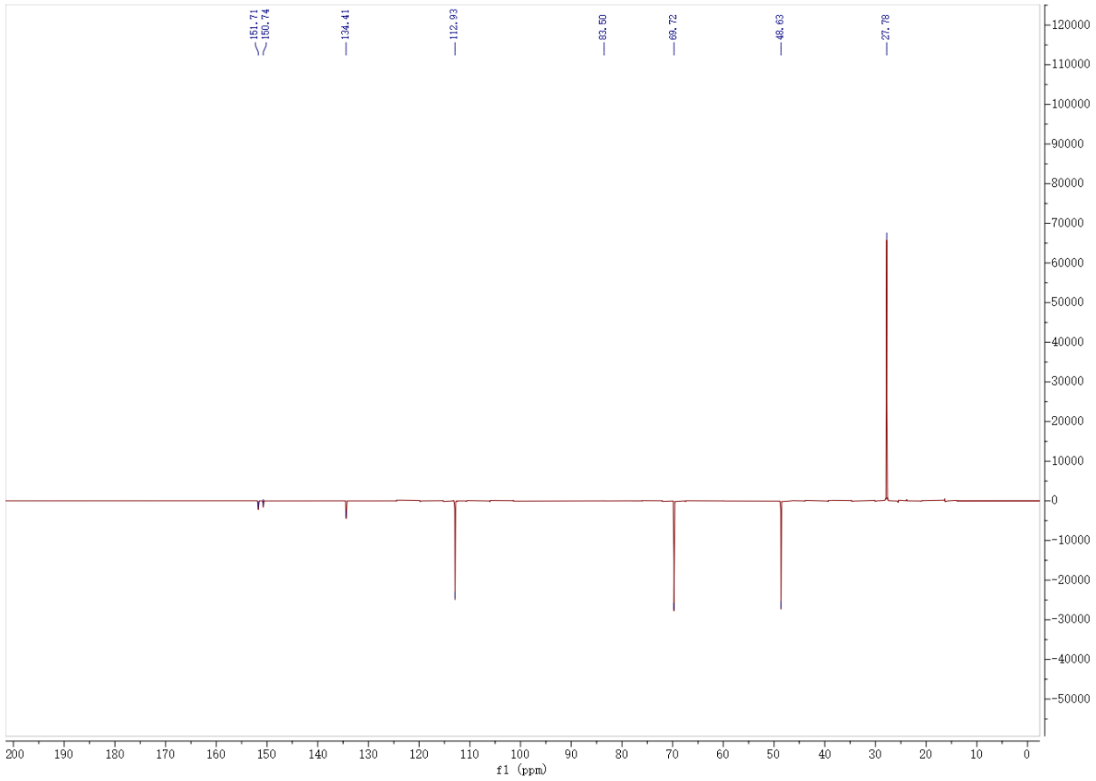
151.71
150.74
134.41
112.93
83.50
69.72
48.63
27.78
f1 (ppm)

**_N,N_-Diisoproyldibenzo[d,f][1,3,2]dioxaphosphenpin-6-amine**[1]

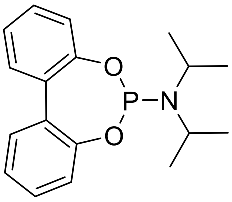

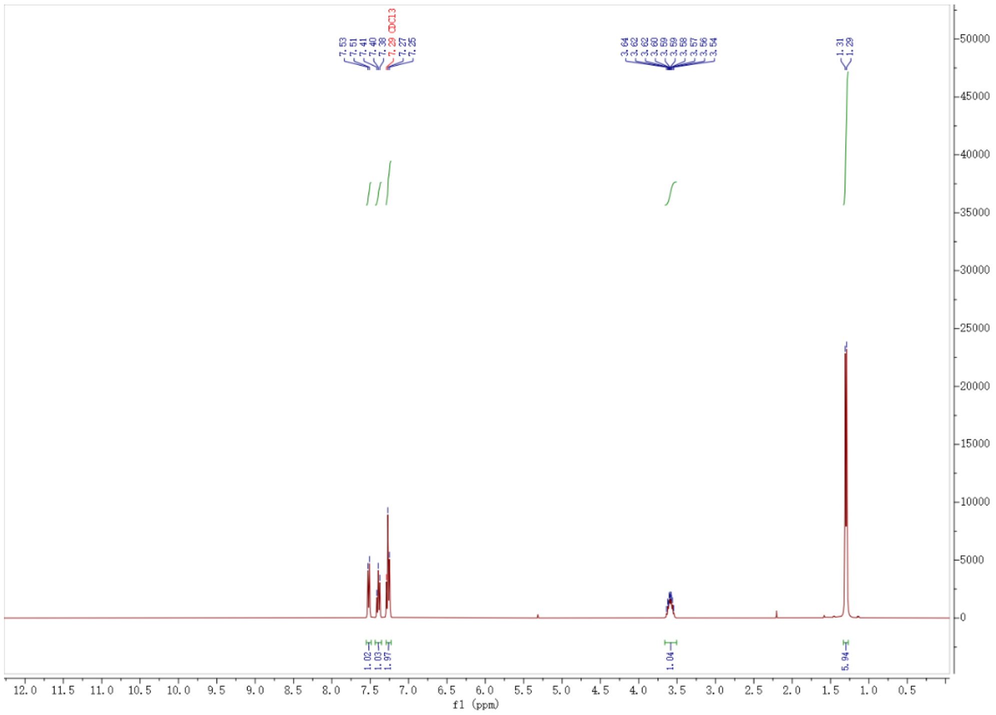


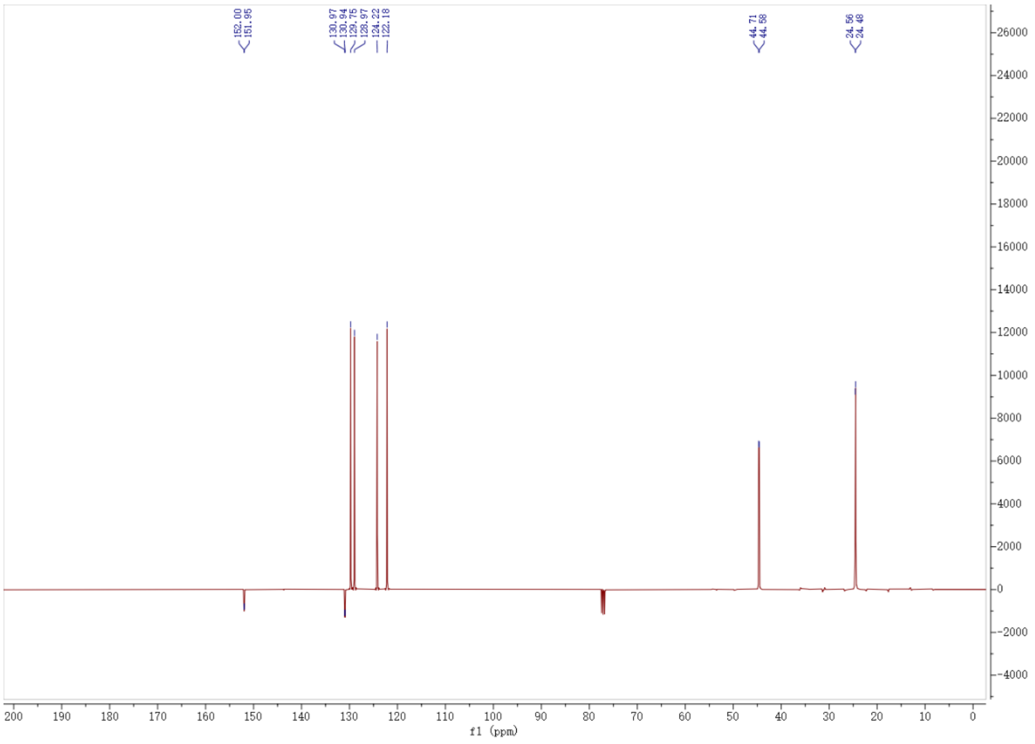

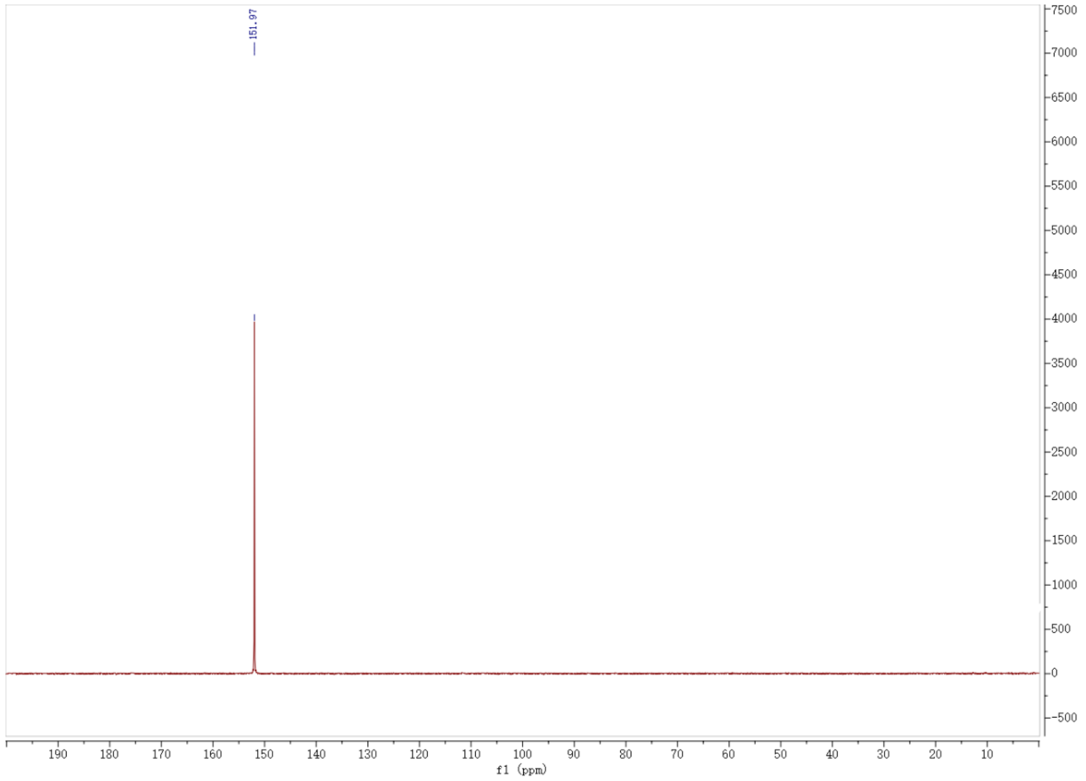

151.97
7500
7000
6500
6000
5500
5000
4500
4000
3500
3000
2500
2000
1500
1000
500
0
−500
190
180
170
160
150
140
130
120
110
100
90
80
70
60
50
40
30
20
10
f1 (ppm)

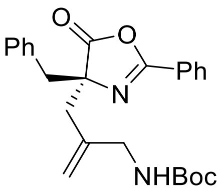

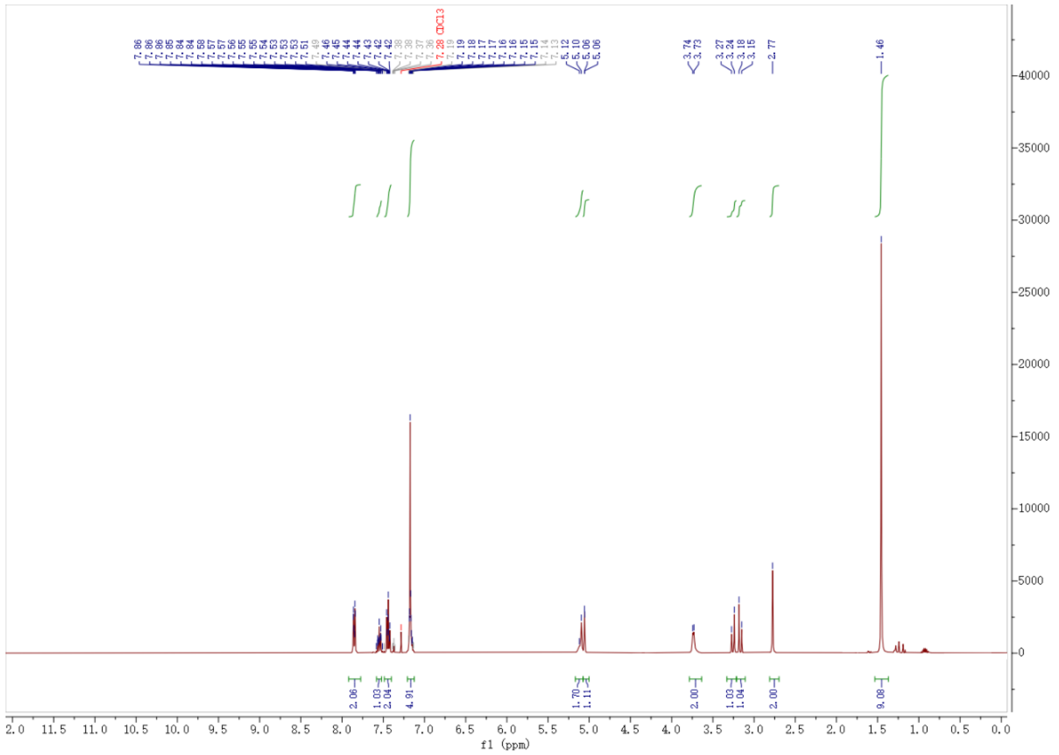

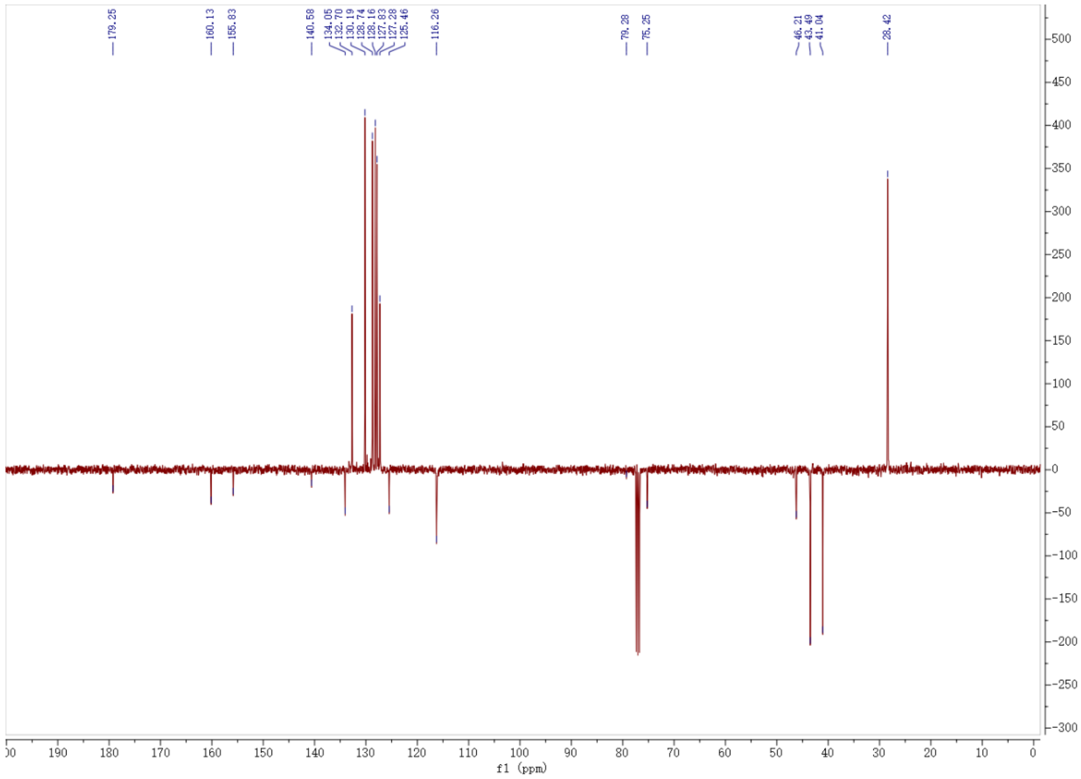

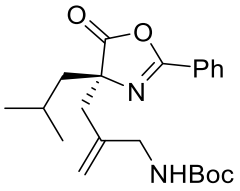
O
O
Ph
N
NHBoc

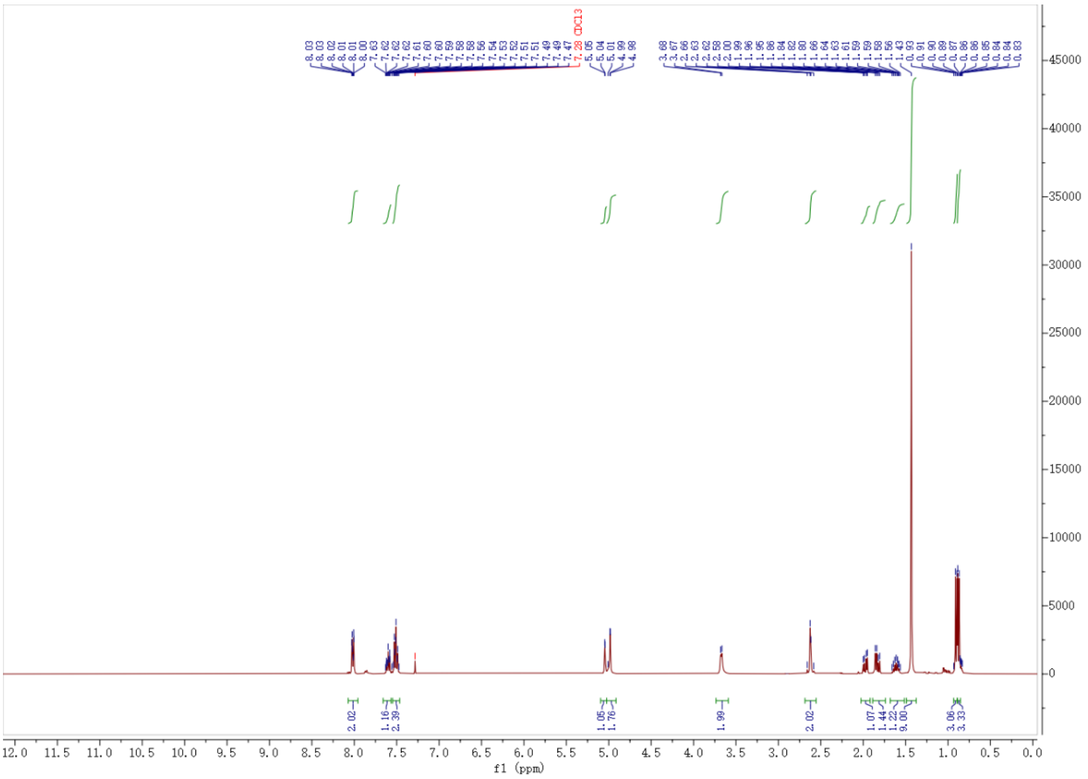
CDCl3
f1 (ppm)

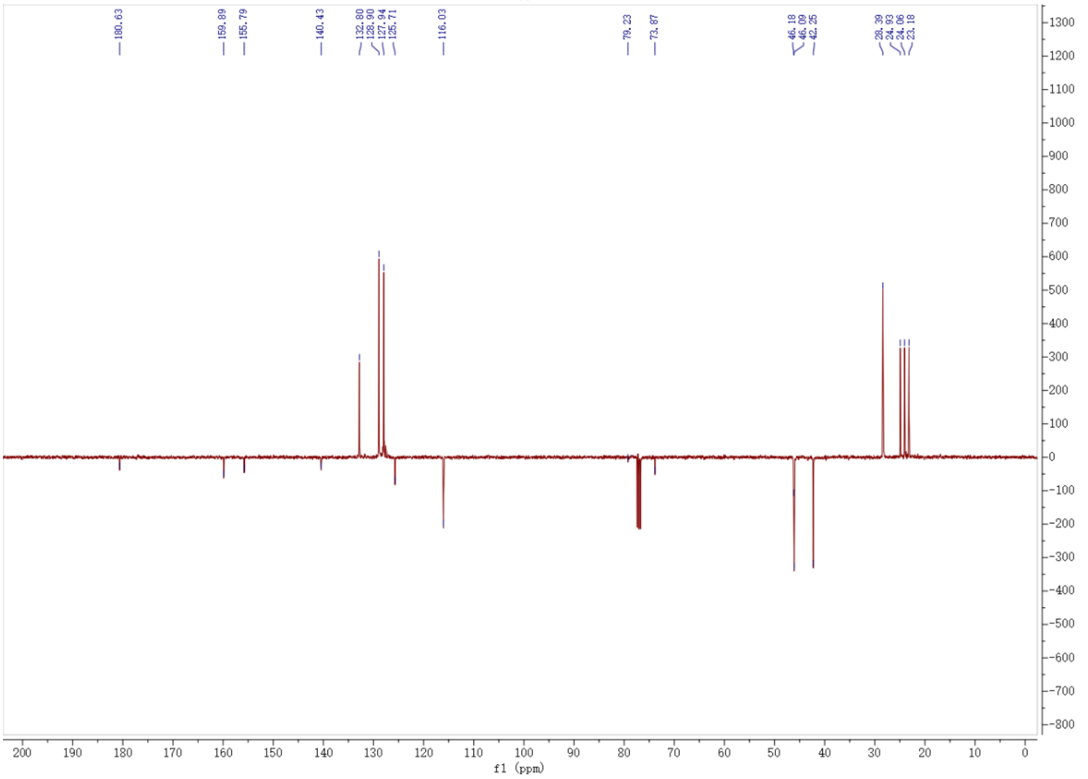
180.63
159.89
155.79
140.43
132.80
128.90
127.94
125.71
116.03
79.23
73.87
46.18
46.09
42.25
28.39
24.93
24.06
23.18
f1 (ppm)

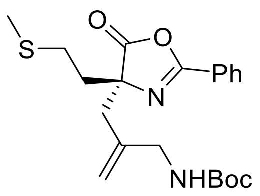
O
O
S
Ph
N
NHBoc

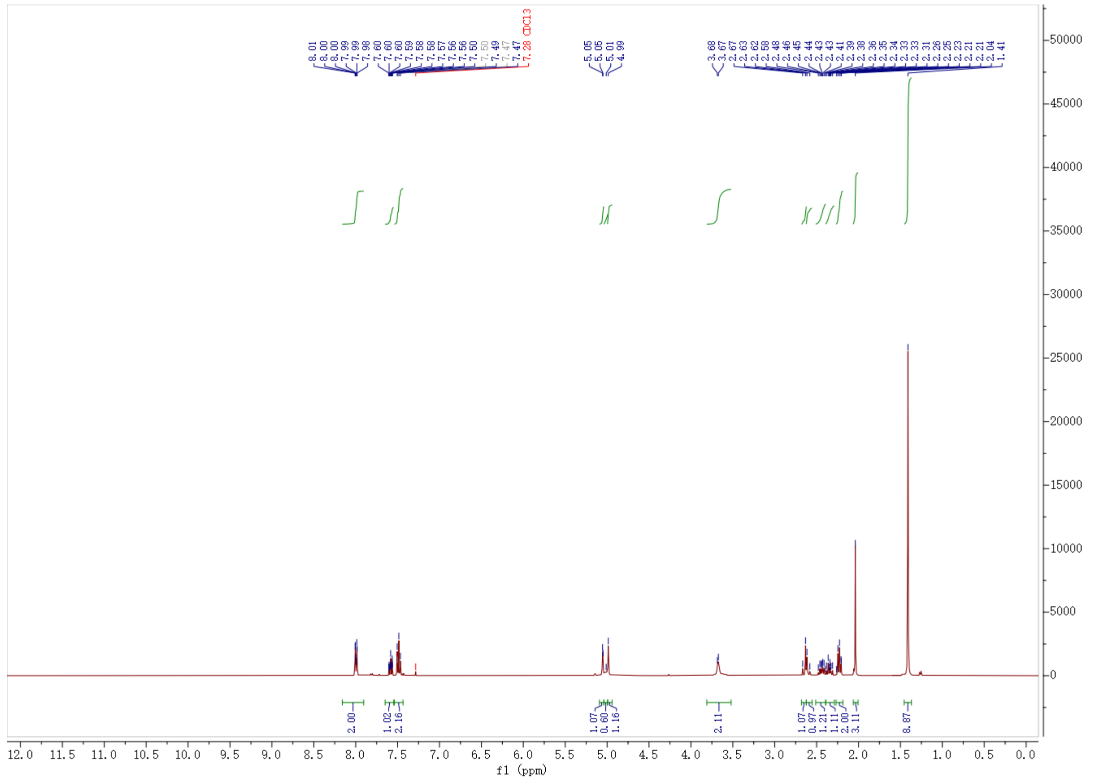

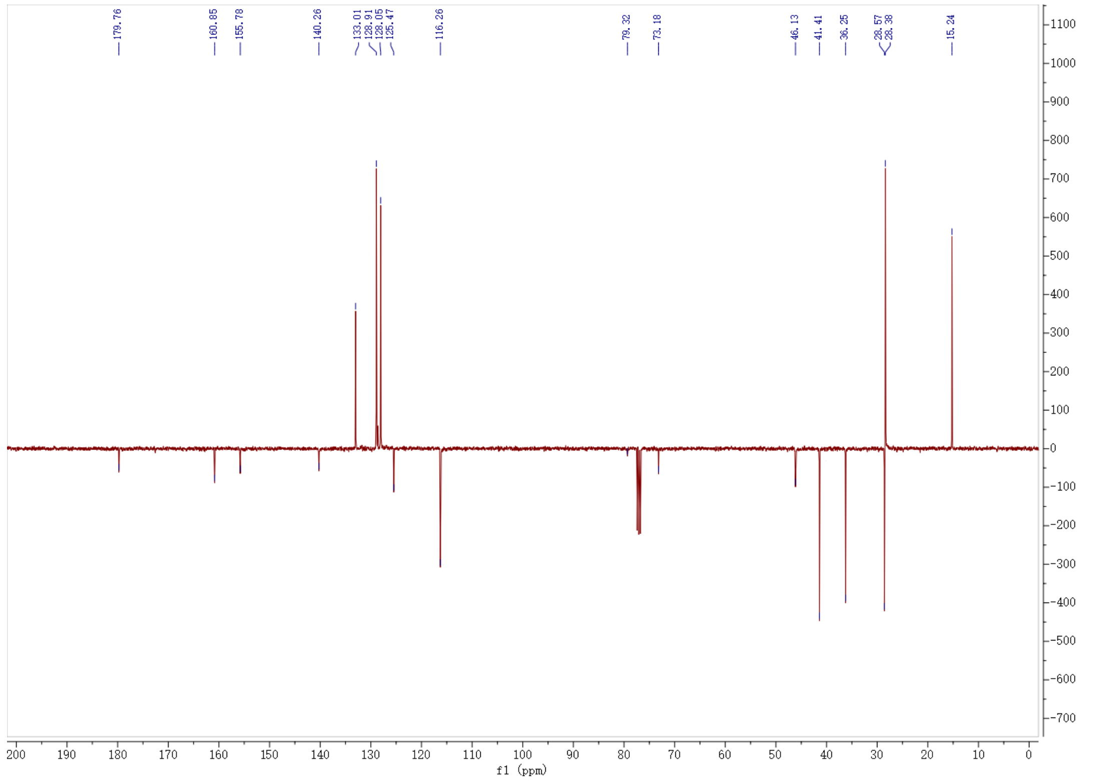

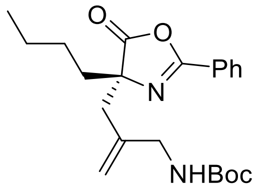

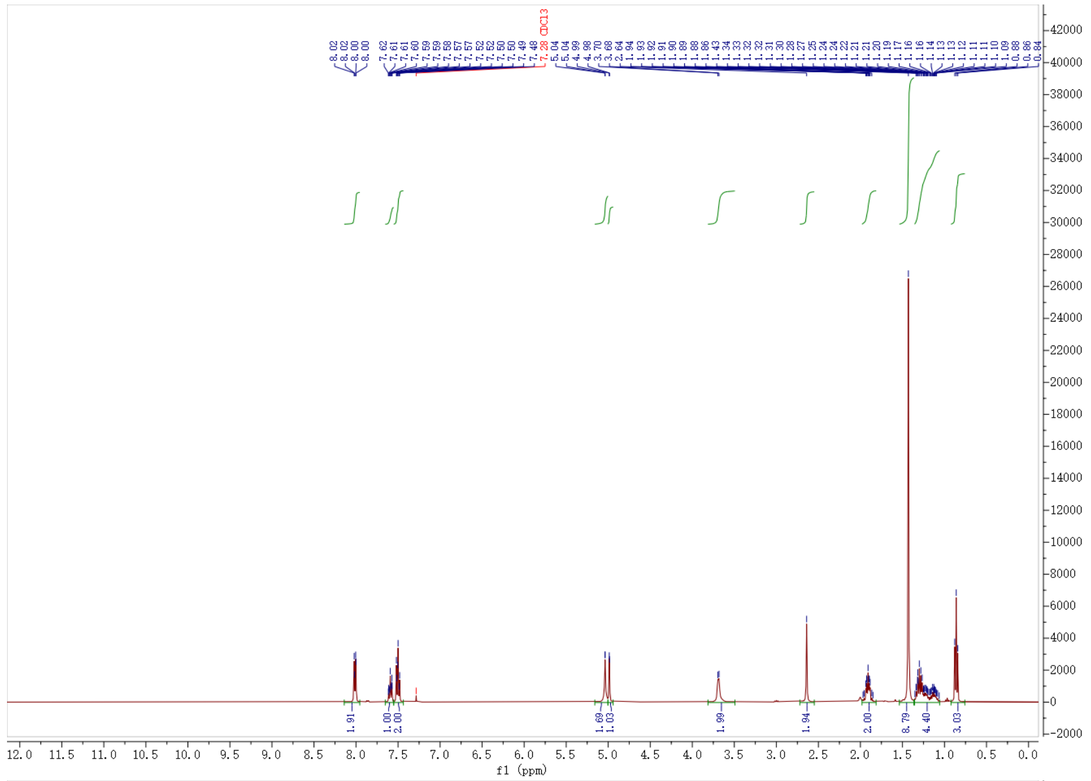

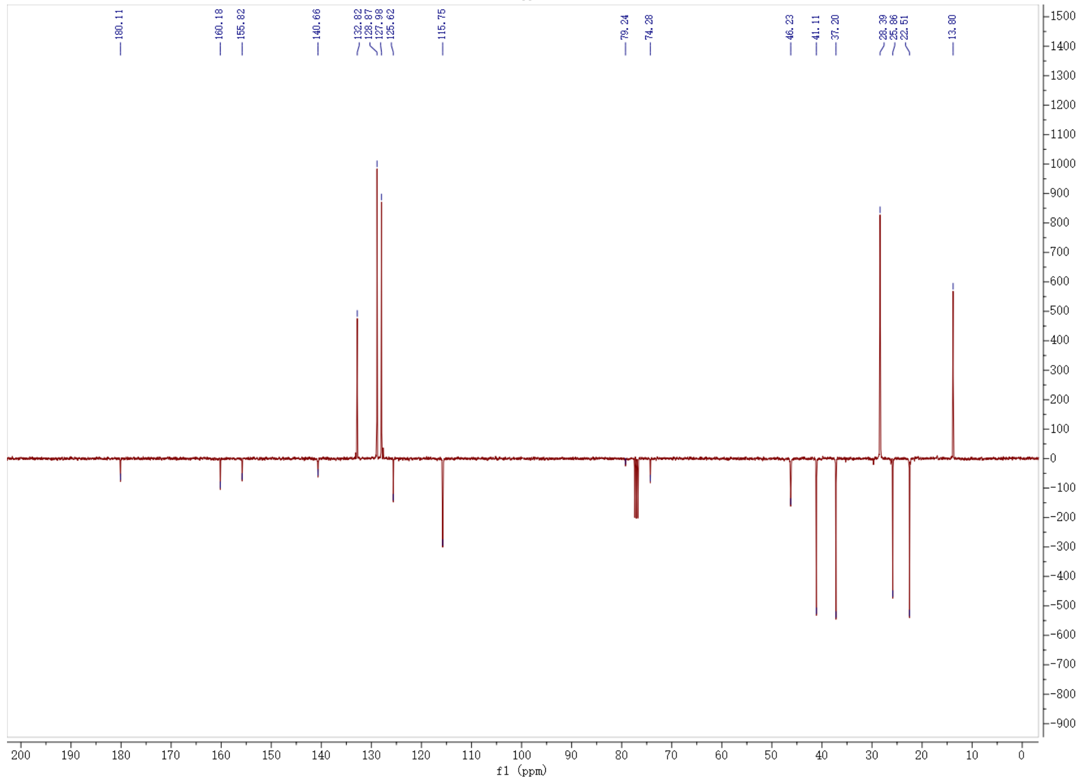

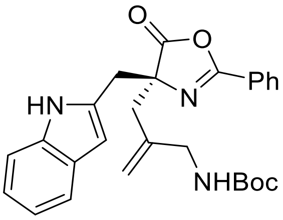
O
O
Ph
N
HN
NHBoc

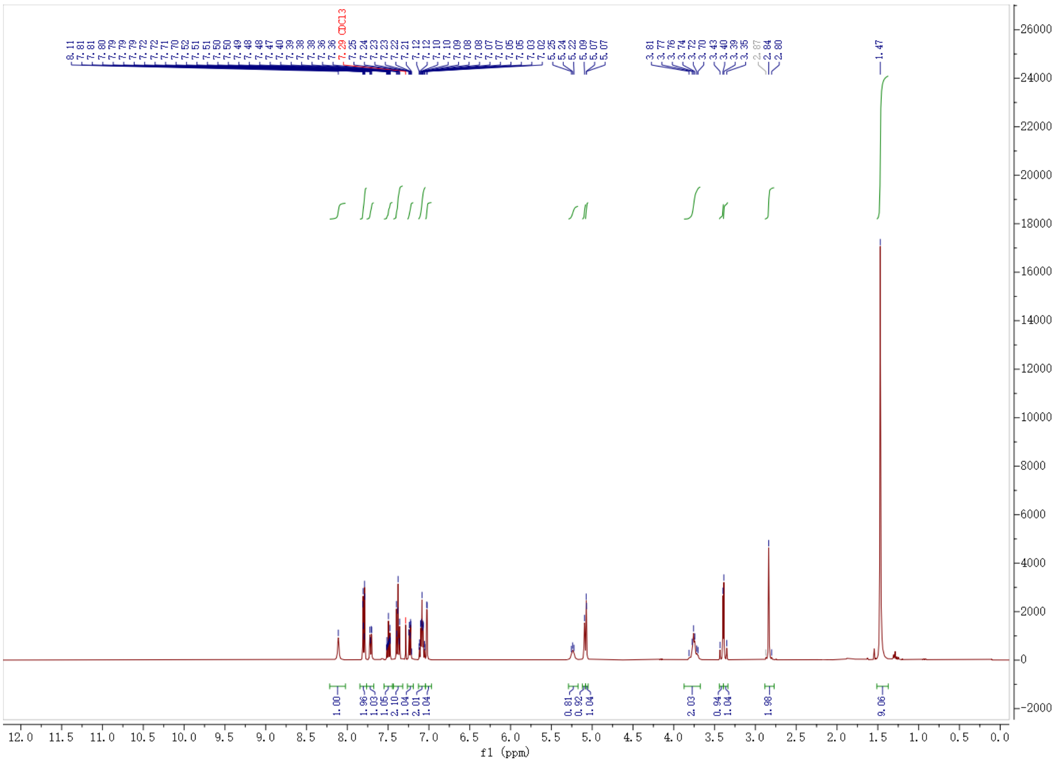
CDCl3
1.47
f1 (ppm)

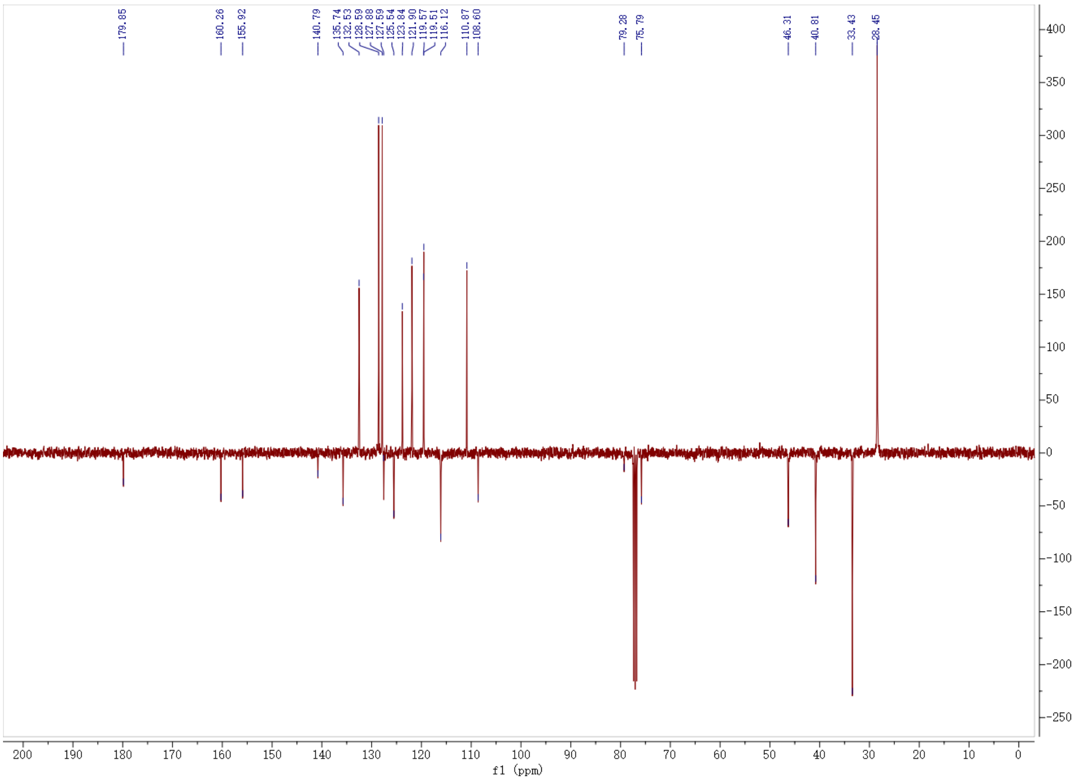
179.85
160.26
155.92
140.79
79.28
75.79
46.31
40.81
33.43
28.45
f1 (ppm)

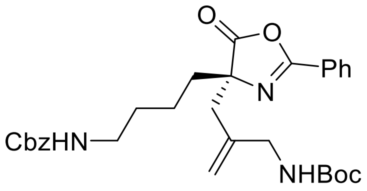

O
O
Ph
N
CbzHN
NHBoc


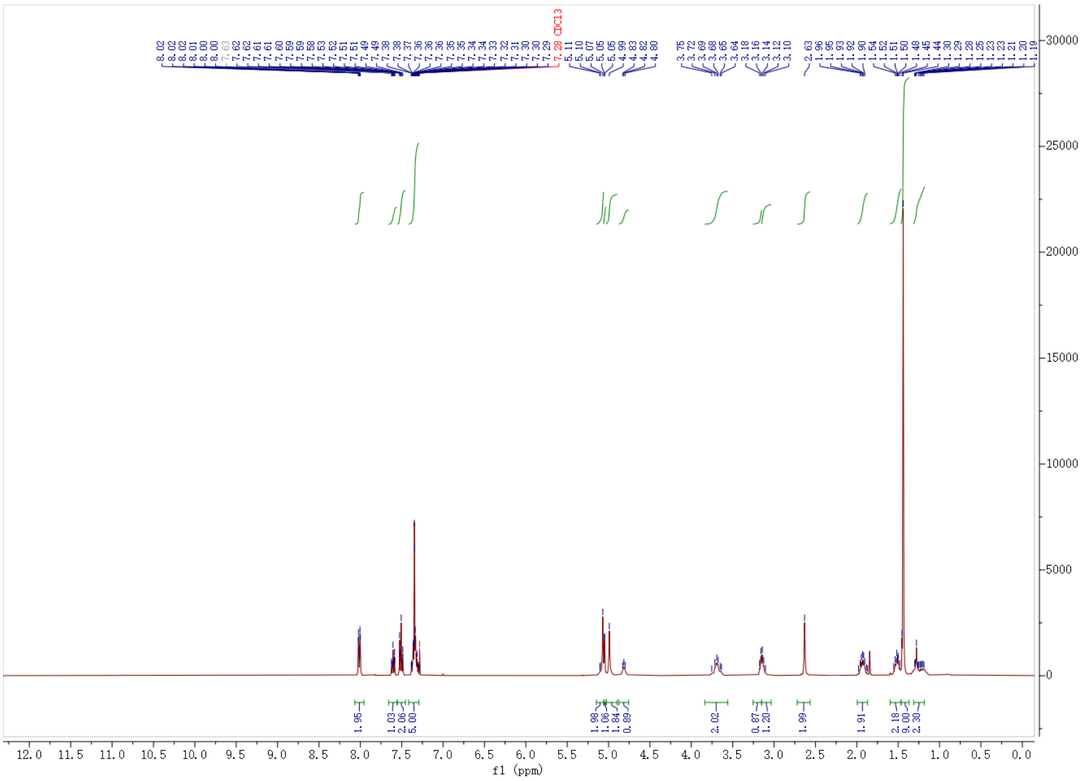

CDCl3
1.95
1.03
2.06
5.00
1.98
1.06
1.84
0.89
2.02
0.87
1.20
1.99
1.91
2.18
9.00
2.30
f1 (ppm)


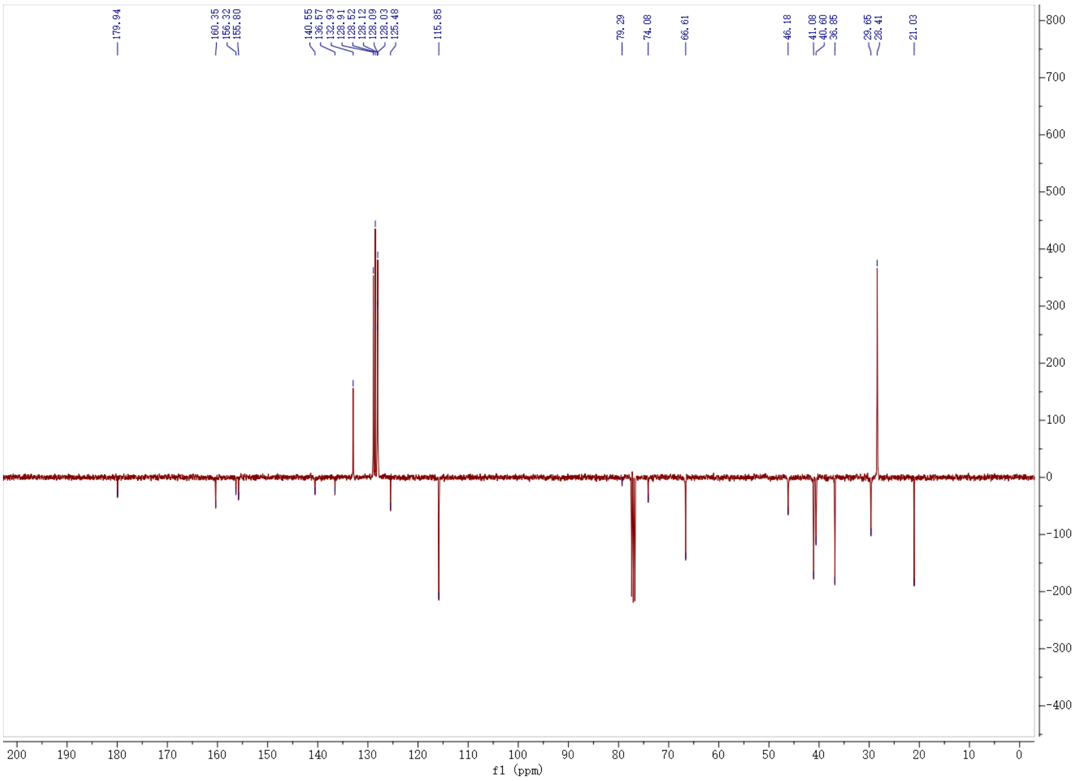

179.94
160.35
156.32
155.80
140.55
136.57
132.93
128.91
128.52
128.12
128.09
128.03
125.48
115.85
79.29
74.08
66.61
46.18
41.08
40.60
36.85
29.65
28.41
21.03
f1 (ppm)

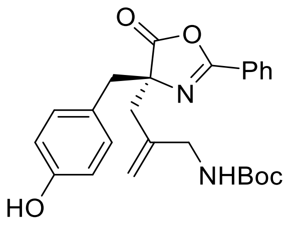
O
O
N
Ph
NHBoc
HO

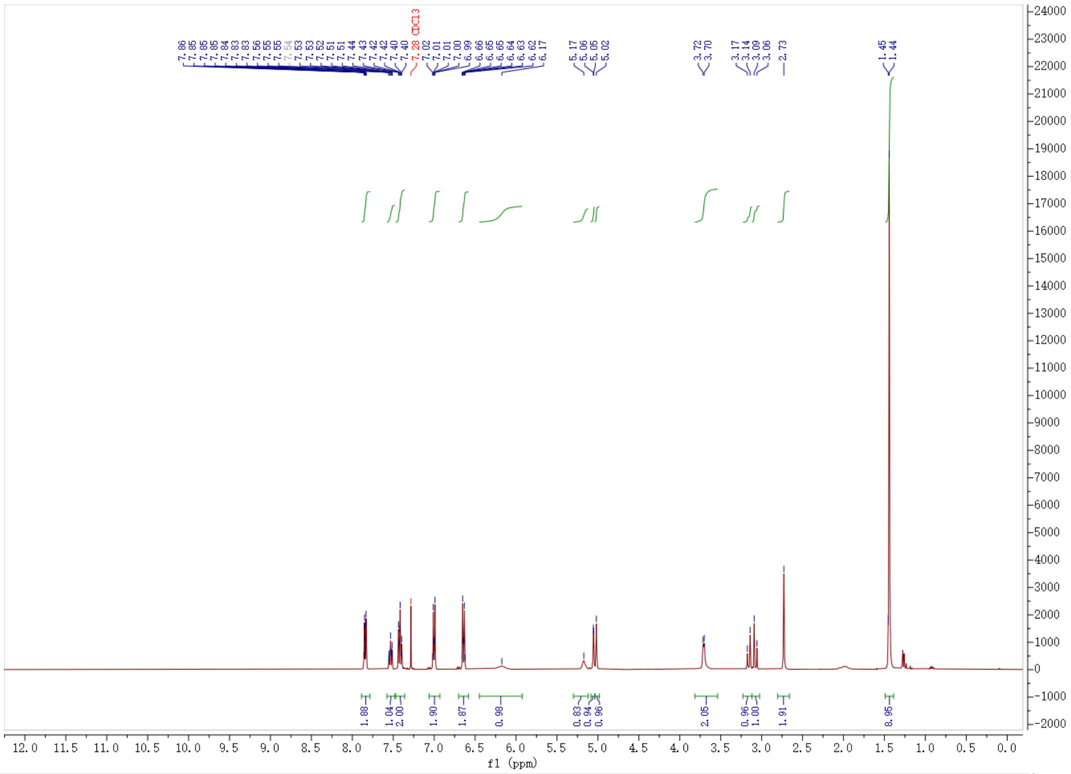

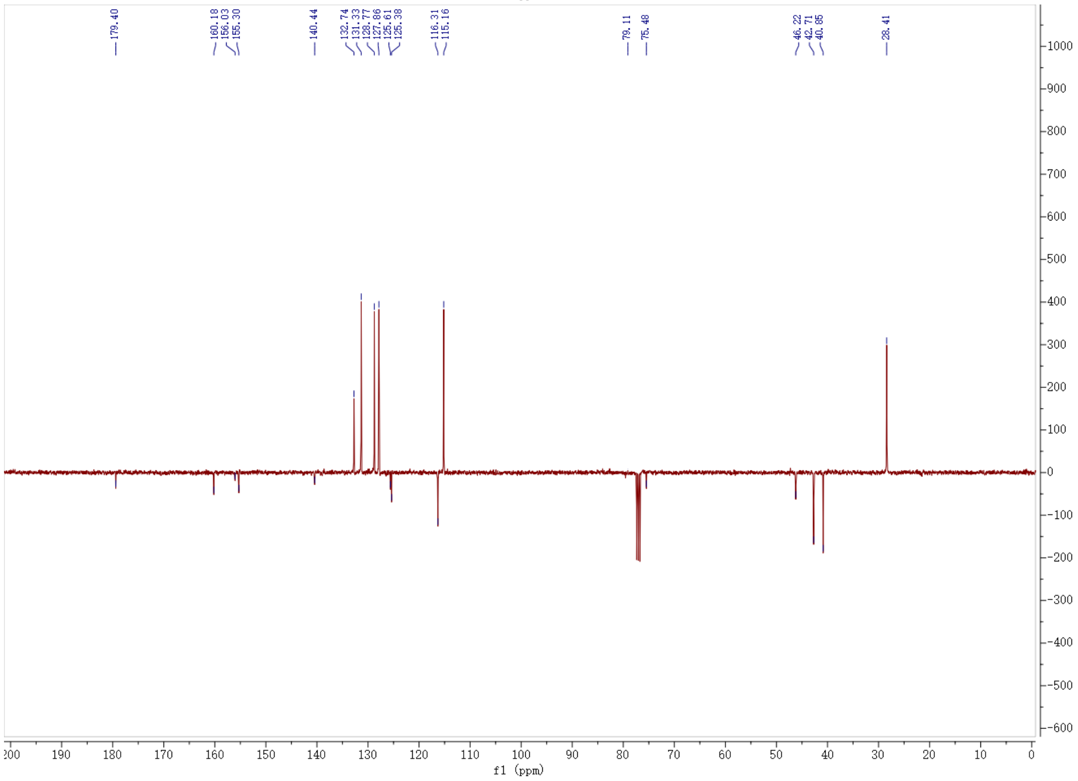

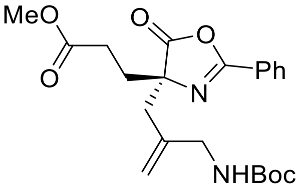
MeO
O
O
O
N
Ph
NHBoc

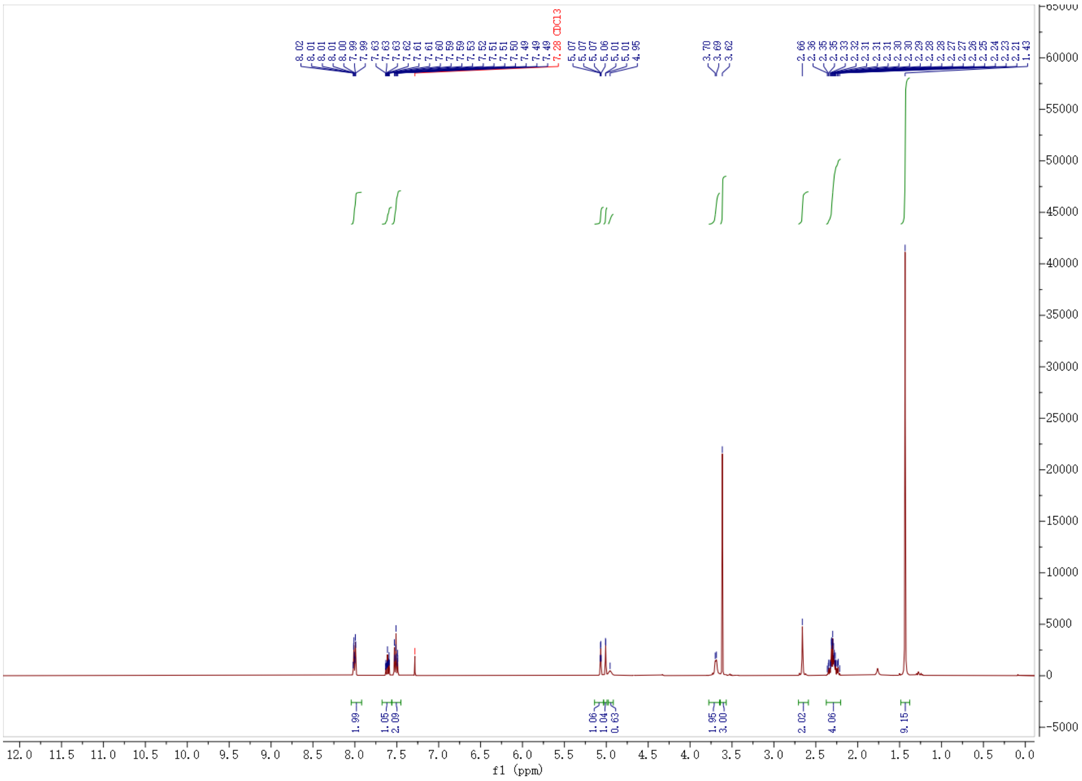
CDCl3
f1 (ppm)

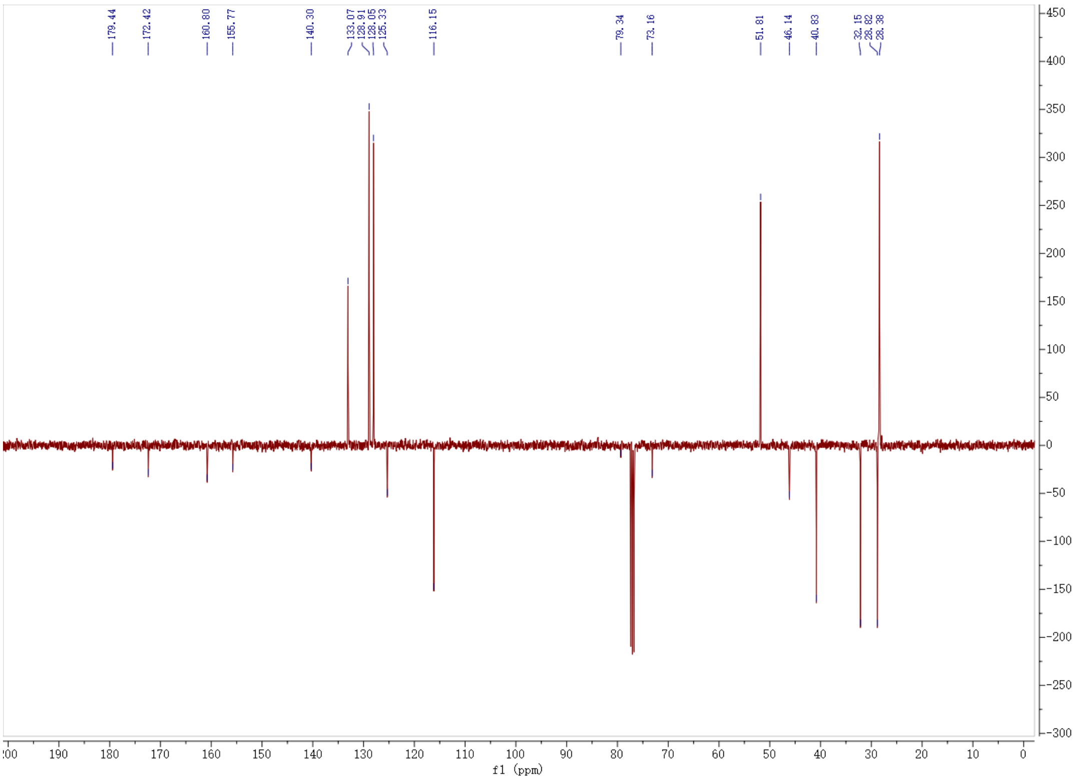
179.44
172.42
160.80
155.77
140.30
133.07
128.91
128.05
125.33
116.15
79.34
73.16
51.81
46.14
40.83
32.15
28.82
28.38
f1 (ppm)

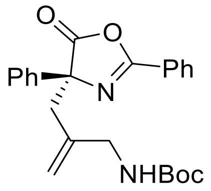
O
O
Ph
Ph
N
NHBoc

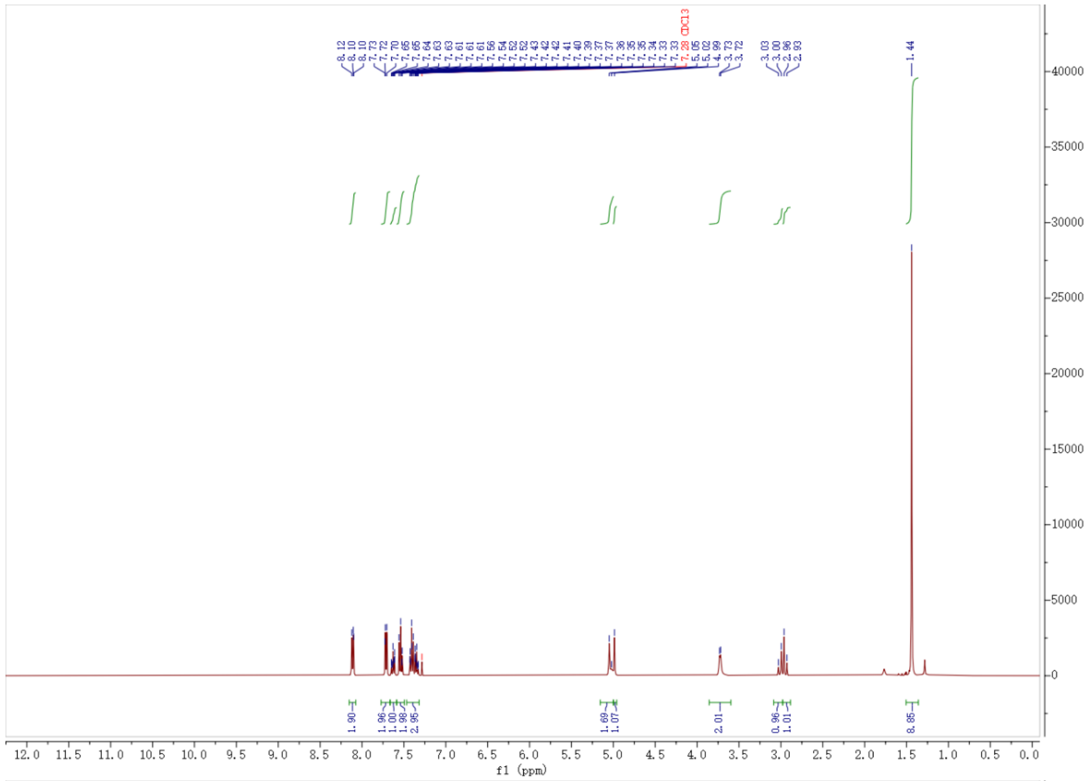
CDCl3
f1 (ppm)

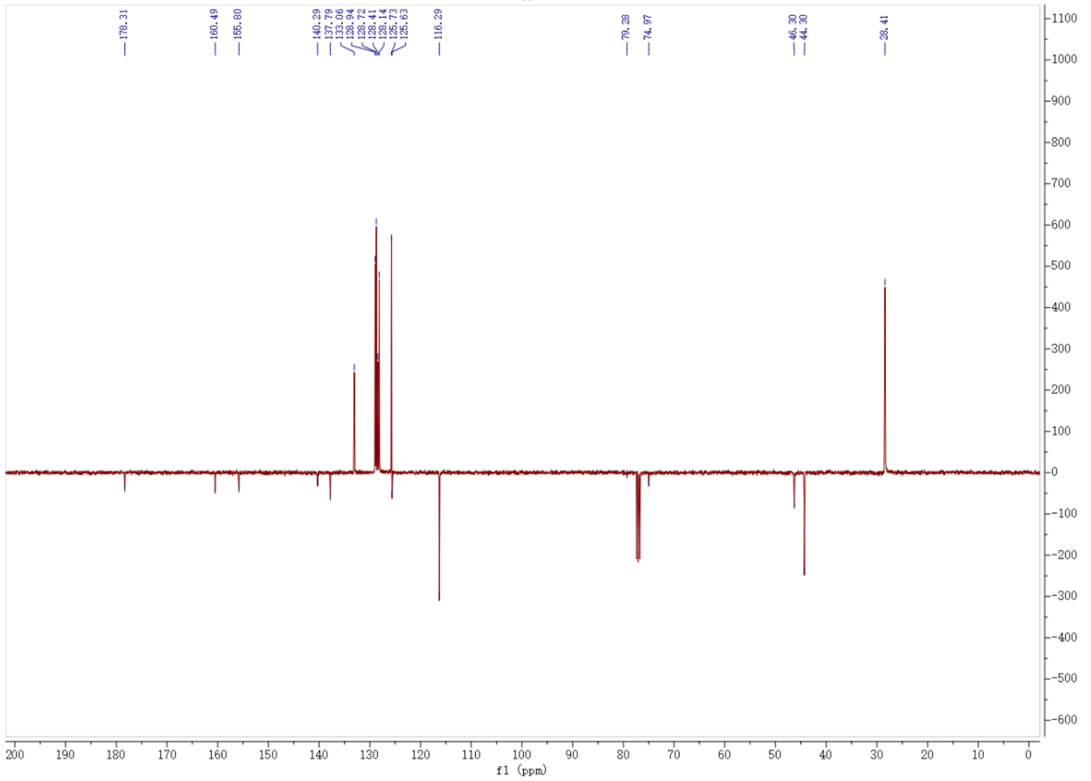
178.31
160.49
155.80
140.29
137.79
133.06
128.94
128.72
128.41
128.14
125.73
125.63
116.29
79.28
74.97
46.30
44.30
28.41
f1 (ppm)

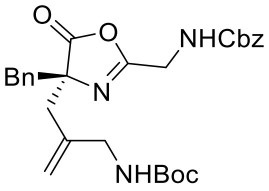

O
O
NHCbz
Bn
N
NHBoc


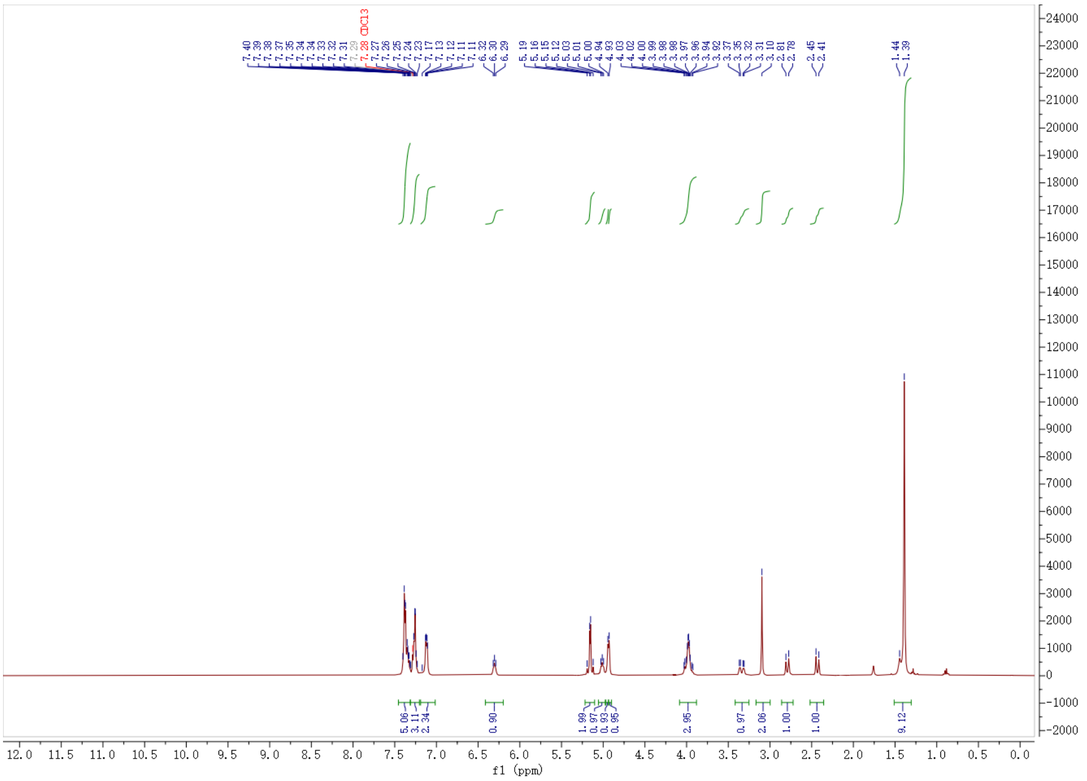

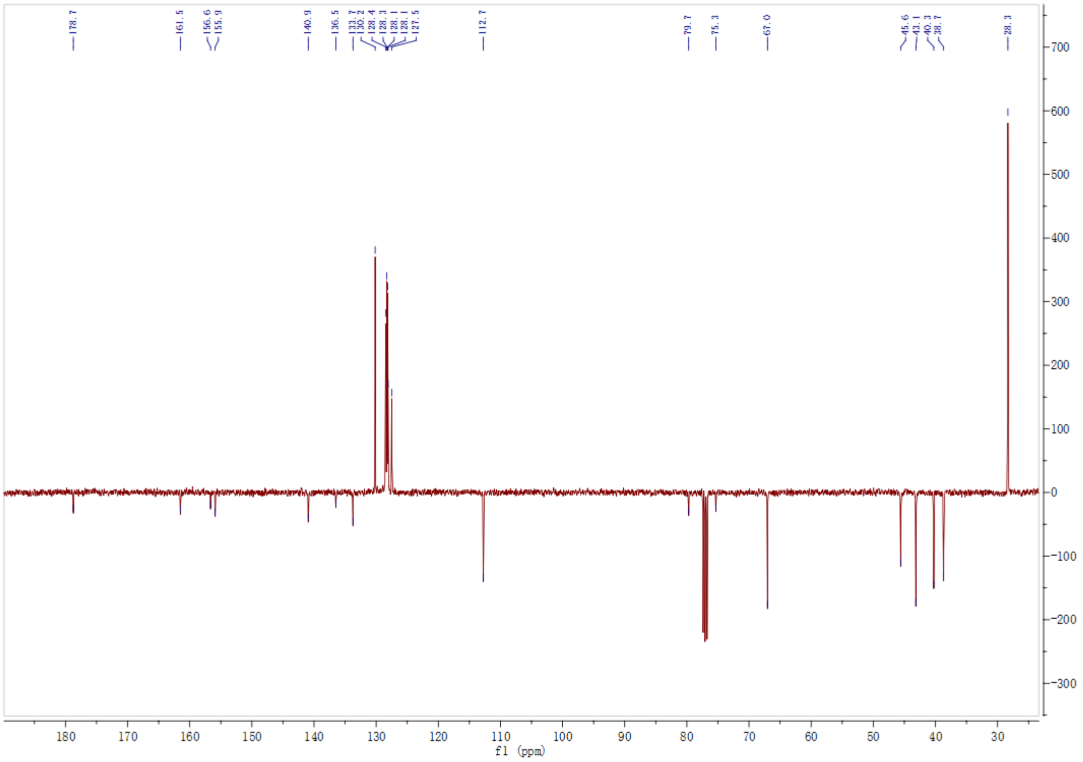

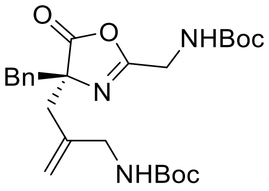
O
O
NHBoc
Bn
N
NHBoc

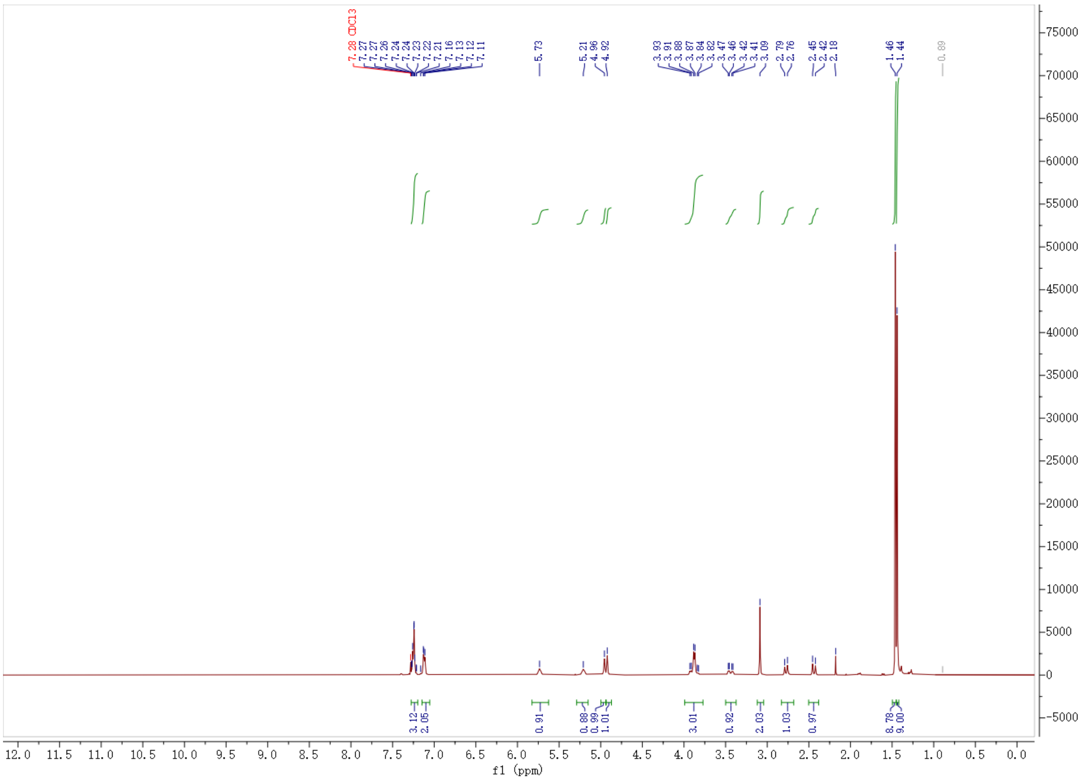
7.28 CDCl3
5.73
1.46
1.44
0.89
f1 (ppm)

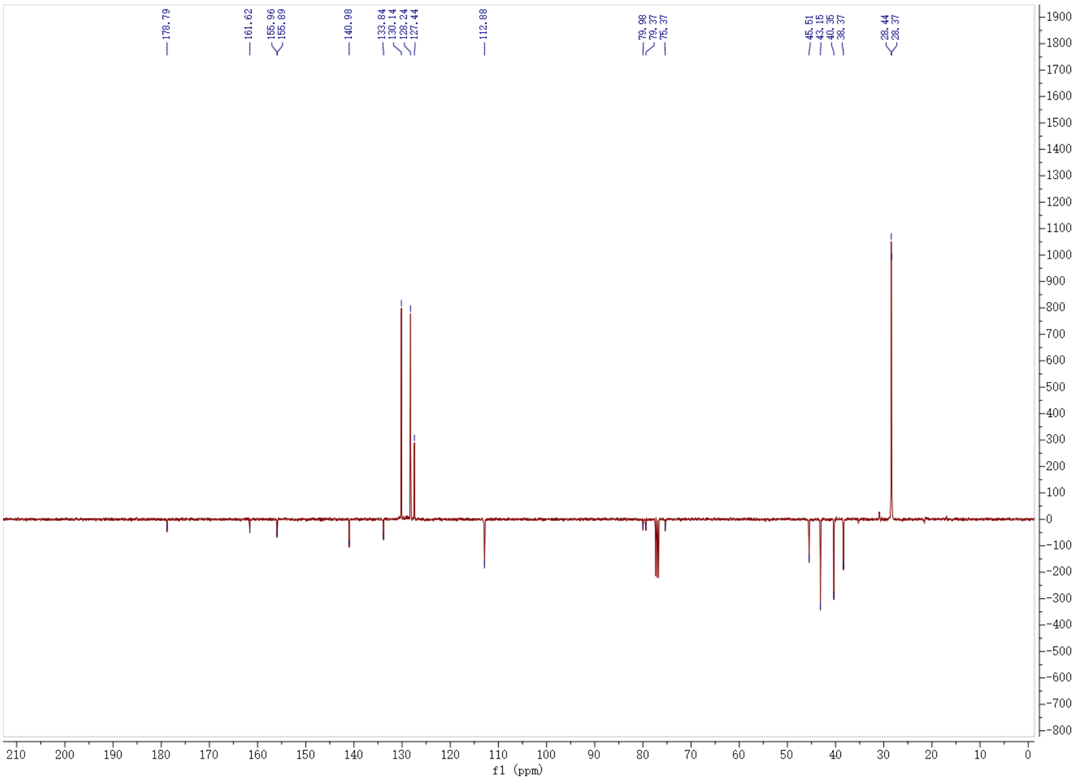
178.79
161.62
155.96
155.89
140.98
133.84
130.14
128.24
127.44
112.88
79.98
79.37
75.37
45.51
43.15
40.35
38.37
28.44
28.37
f1 (ppm)

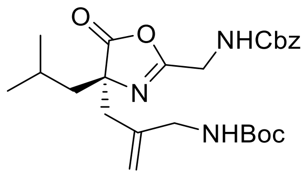
O
O
N
NHCbz
NHBoc

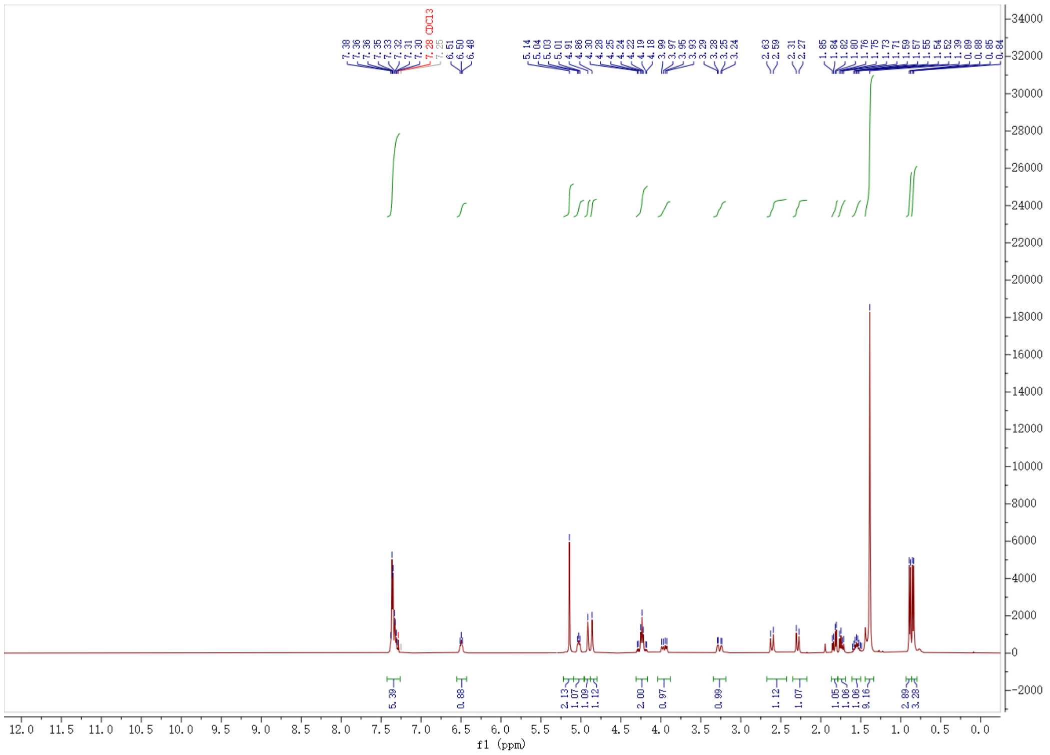
CDCl3
f1 (ppm)

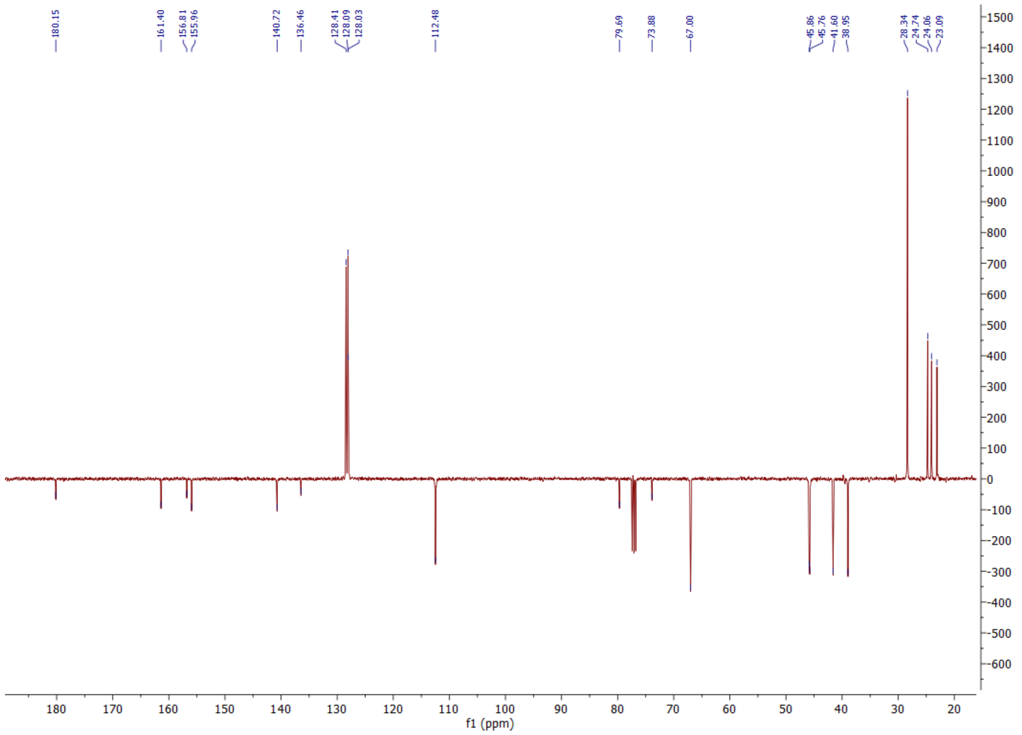
180.15
161.40
156.81
155.96
140.72
136.46
128.41
128.09
128.03
112.48
79.69
73.88
67.00
45.86
45.76
41.60
38.95
28.34
24.74
24.06
23.09
f1 (ppm)

**3**

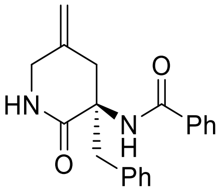


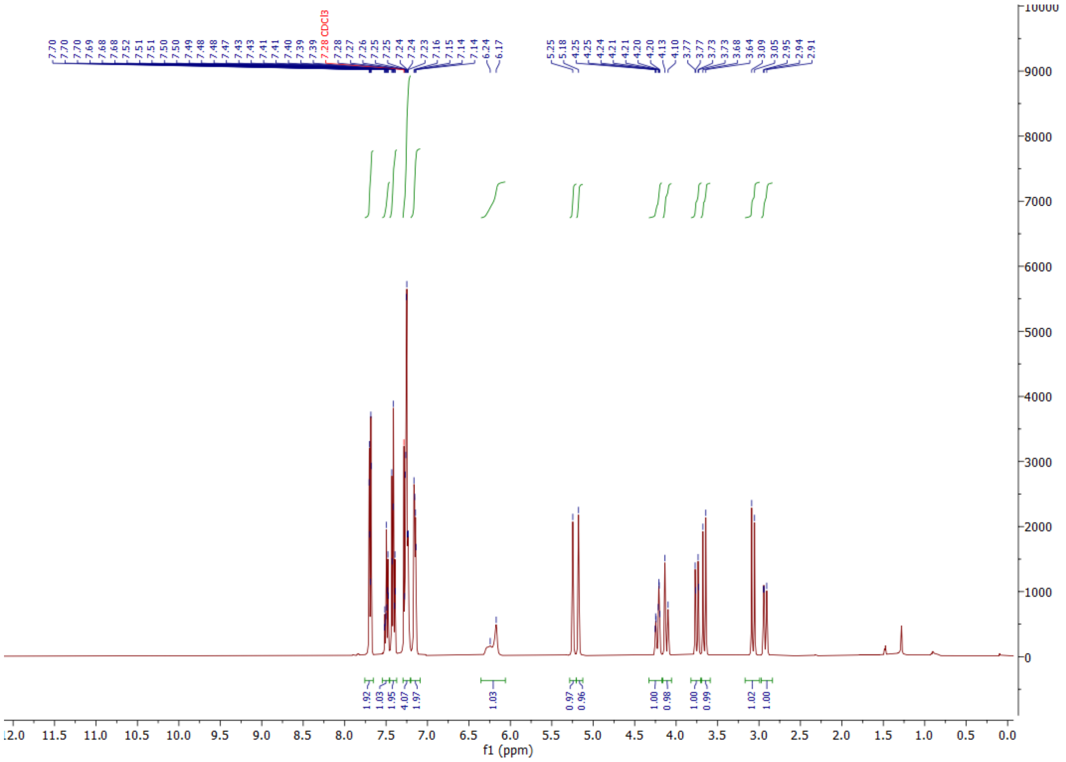

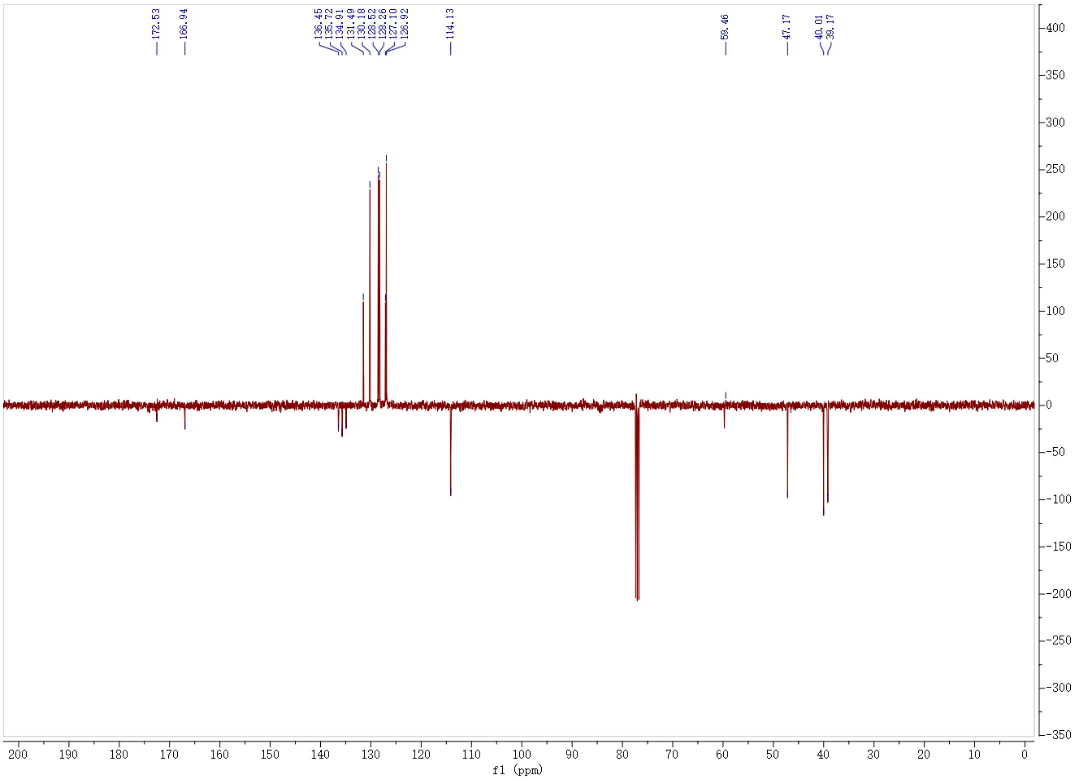

**5**

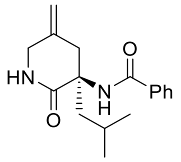


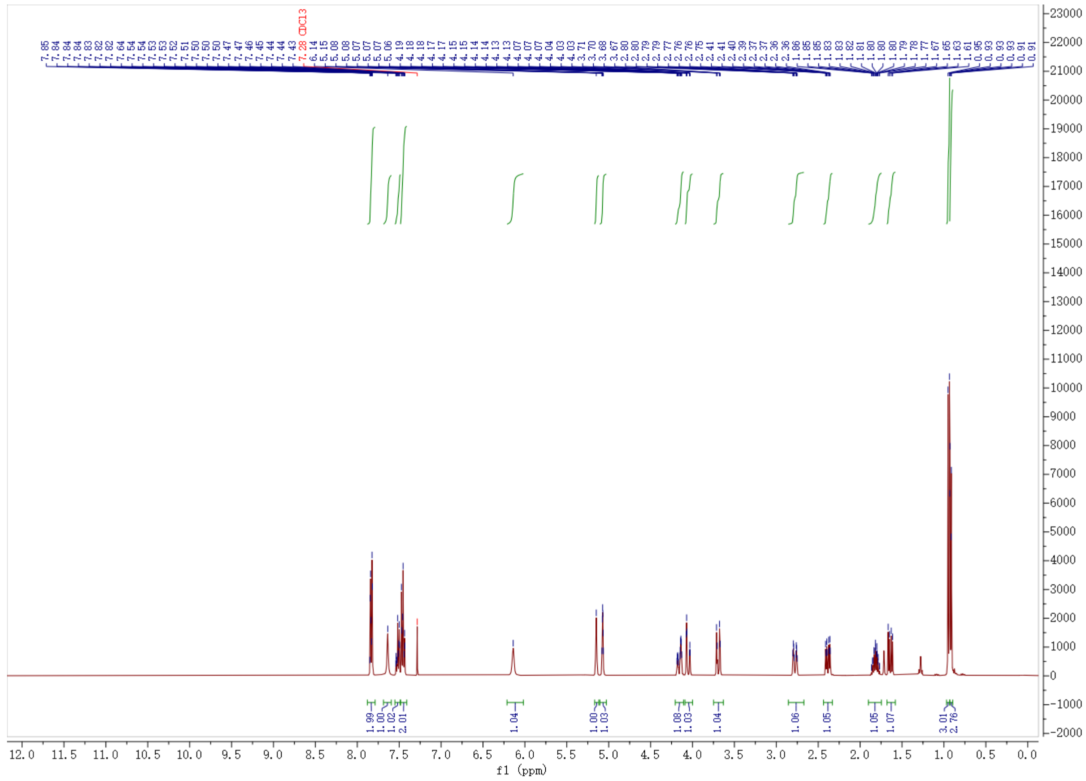

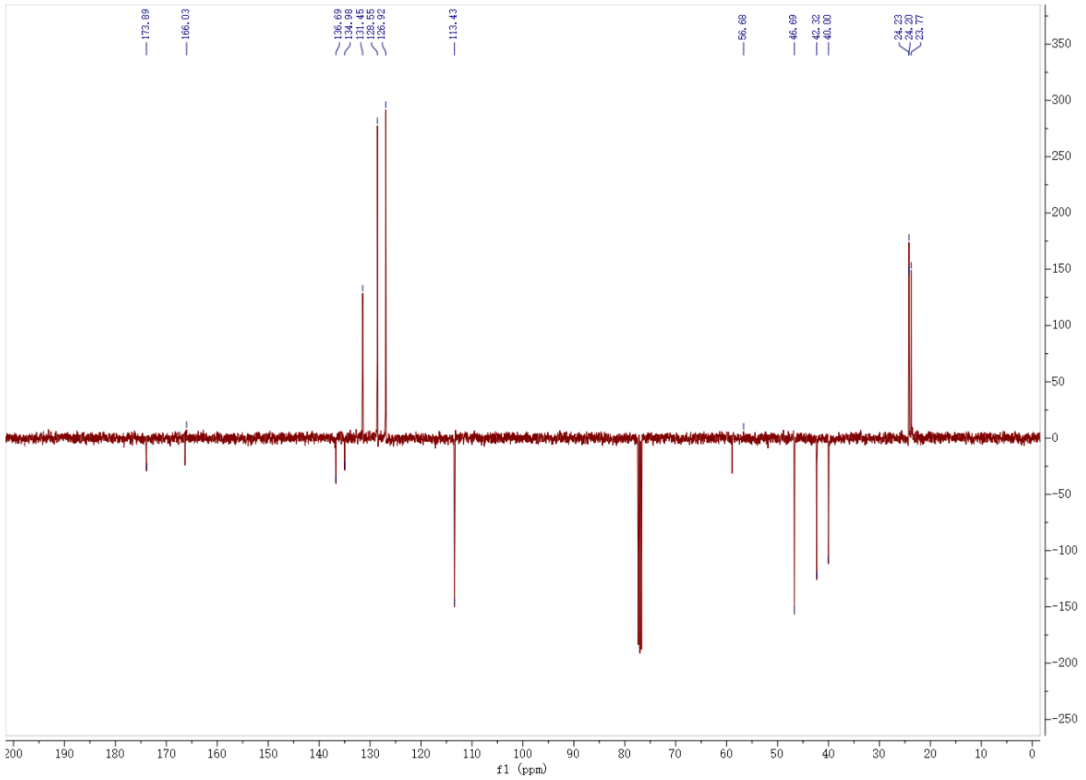

**6**

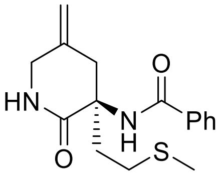

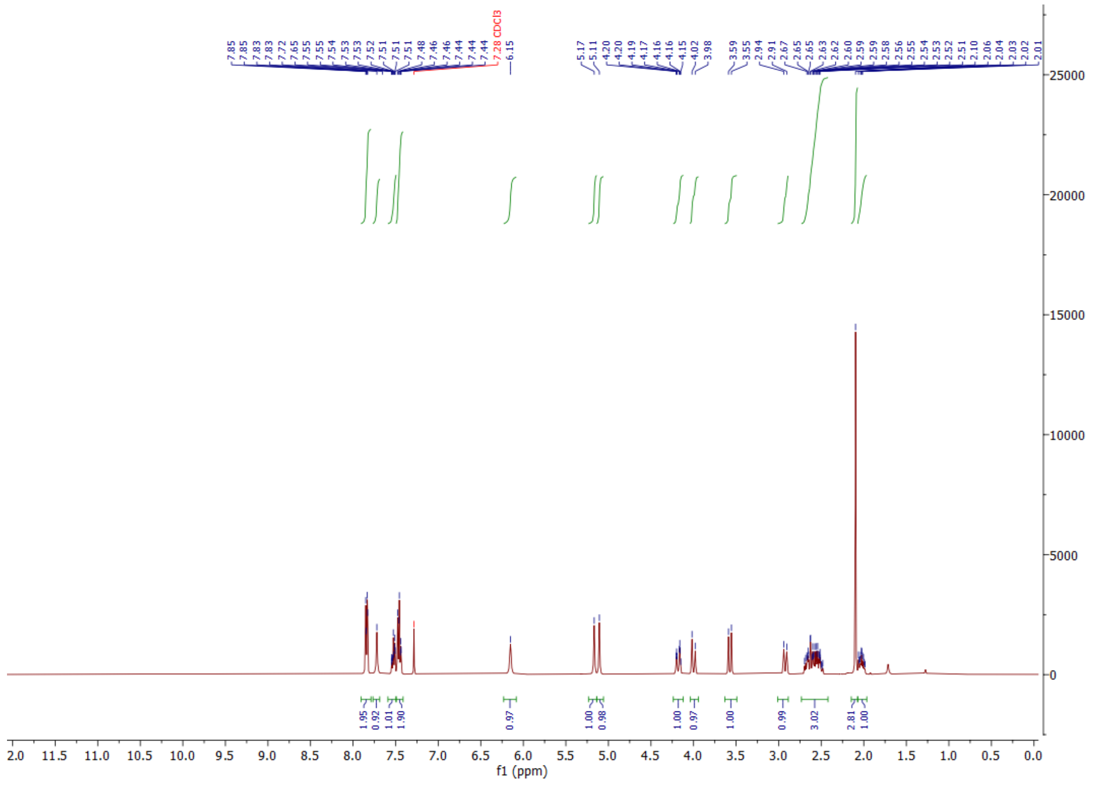

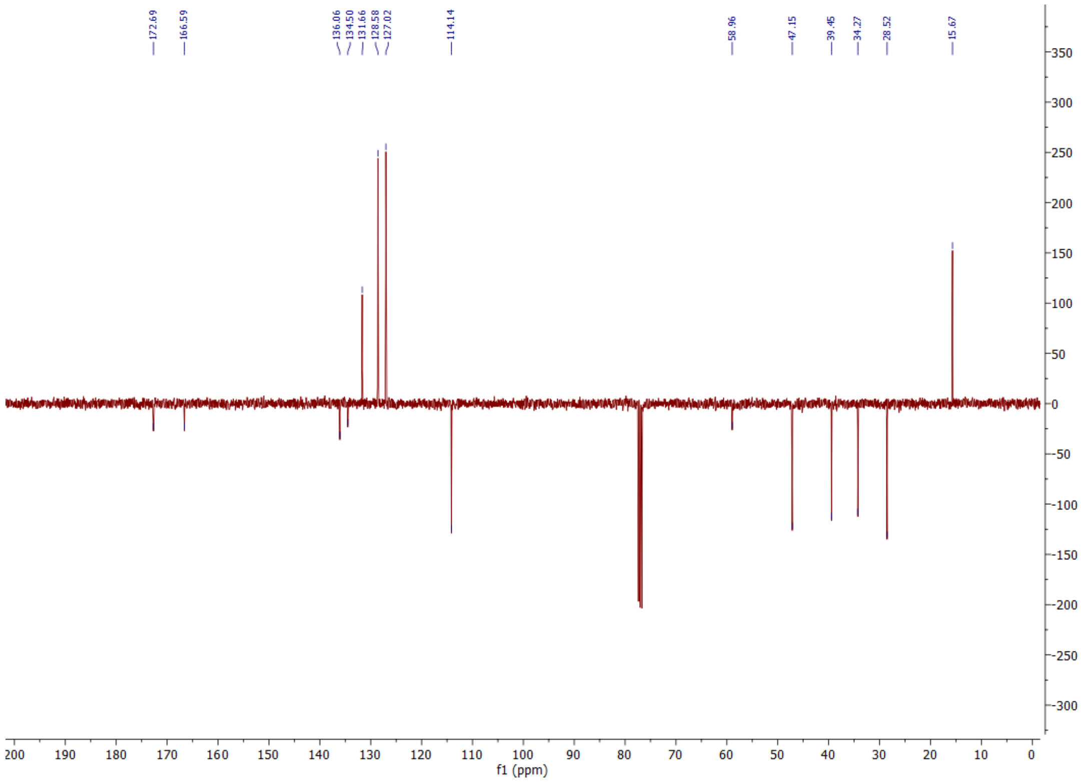

**7**

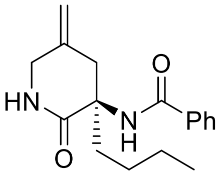


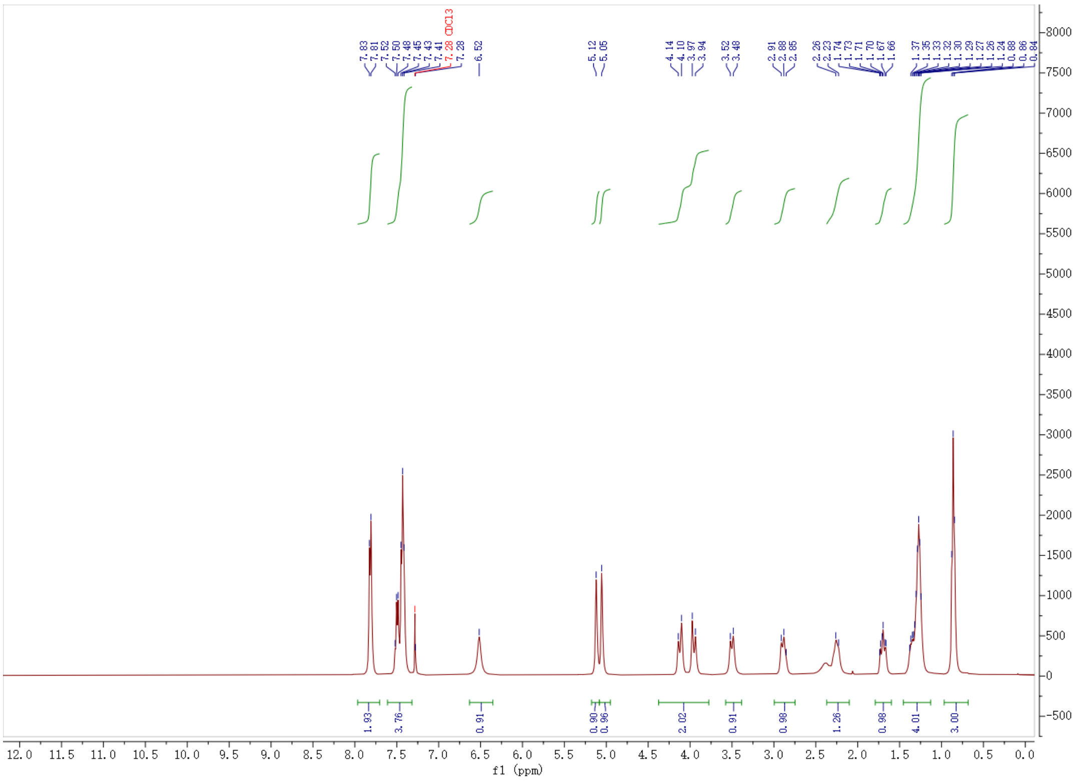

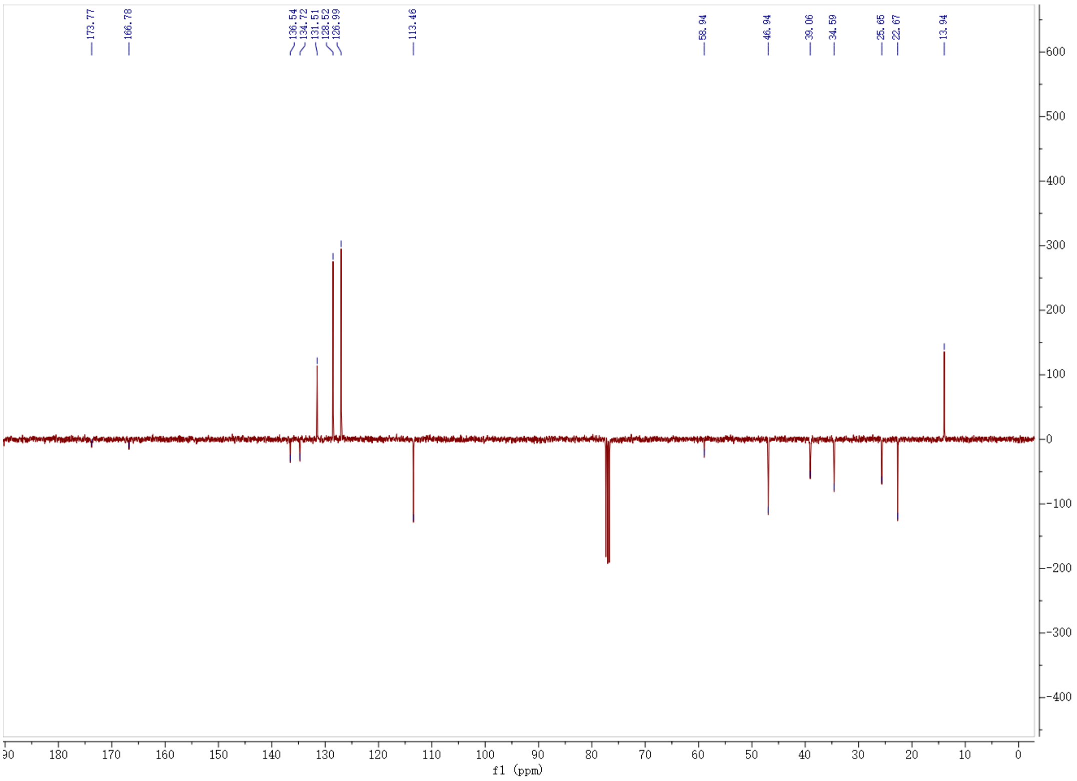

**8**

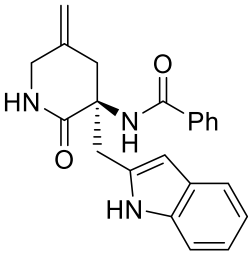


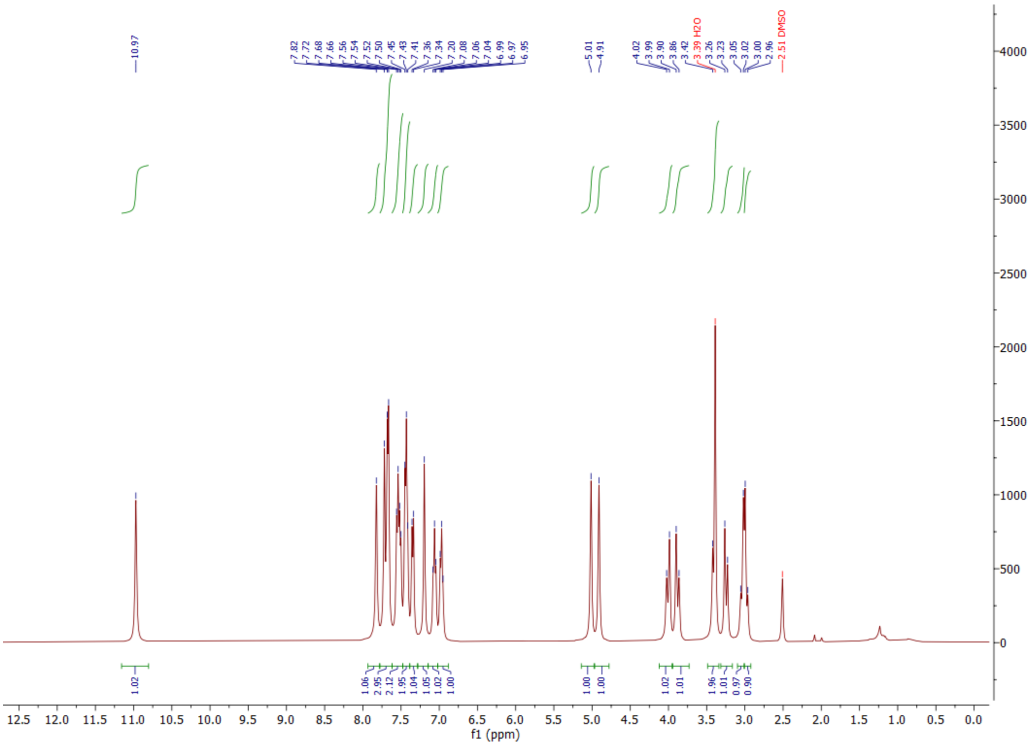


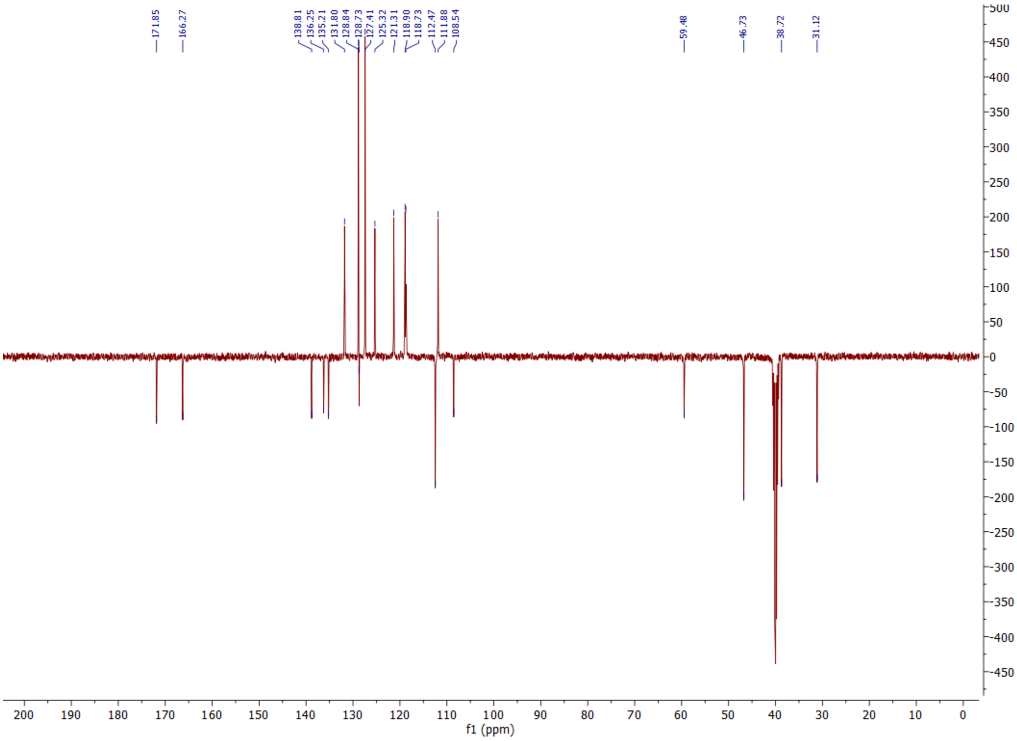

**9**

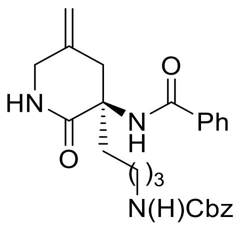


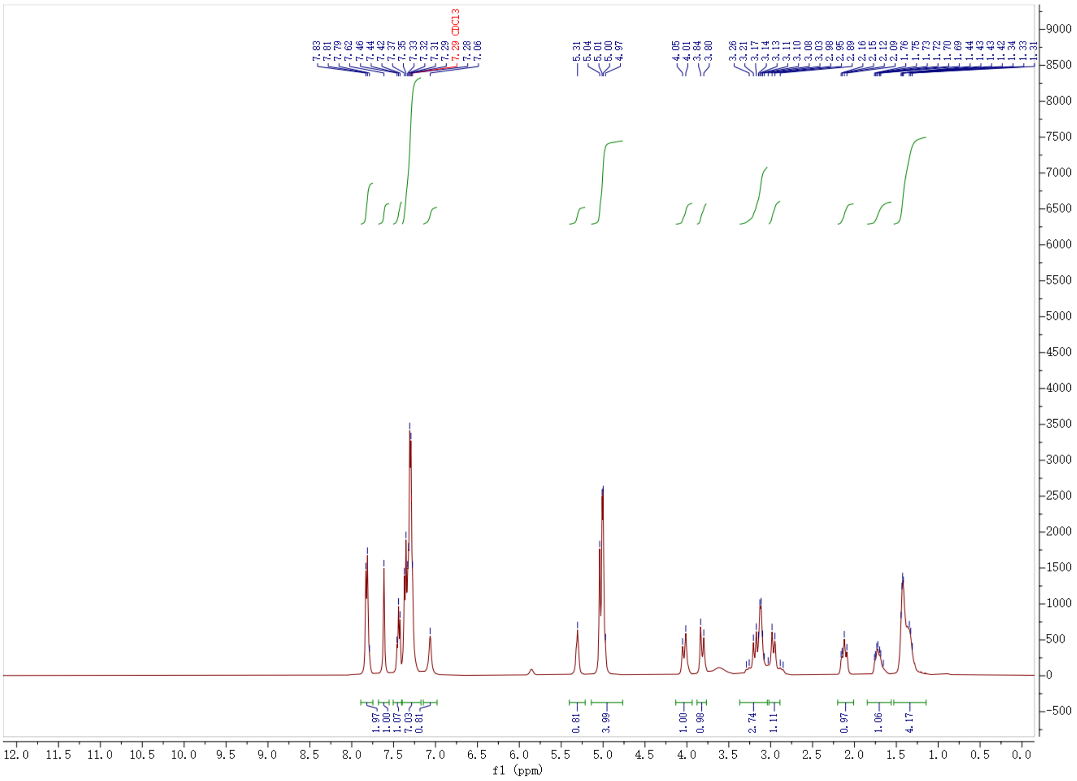

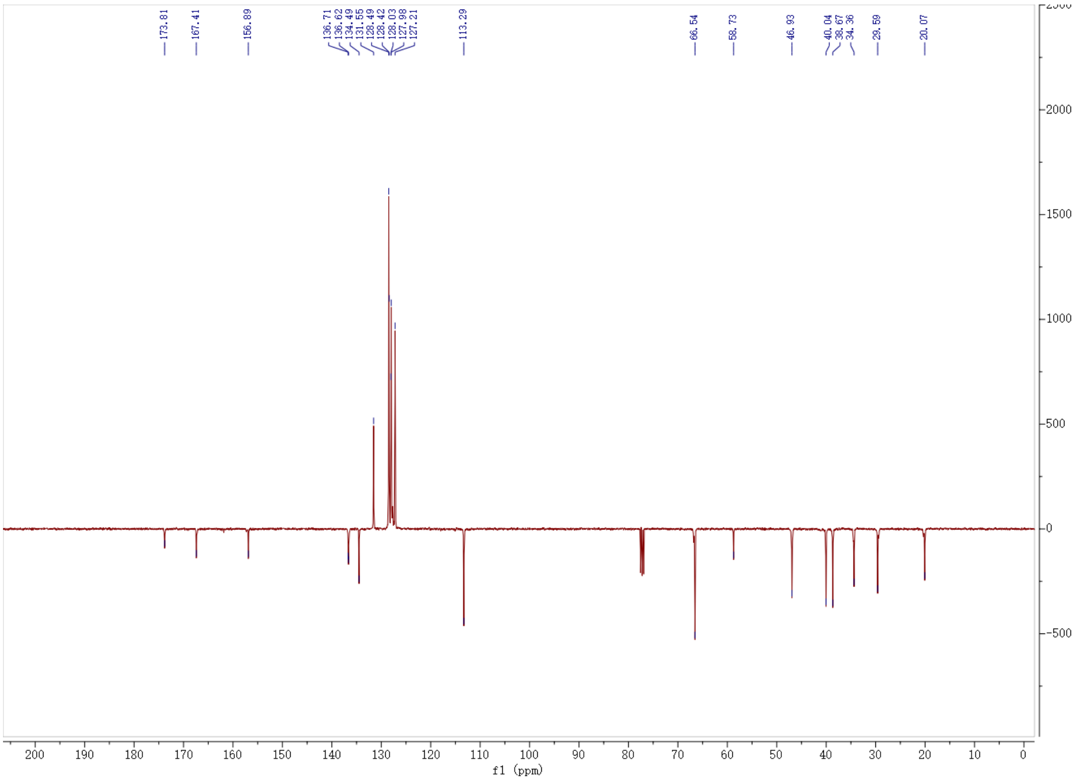

**10**

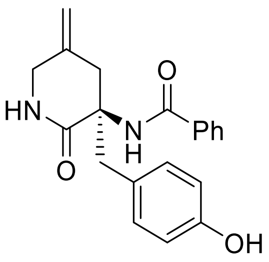
HN
O
N
H
O
Ph
OH

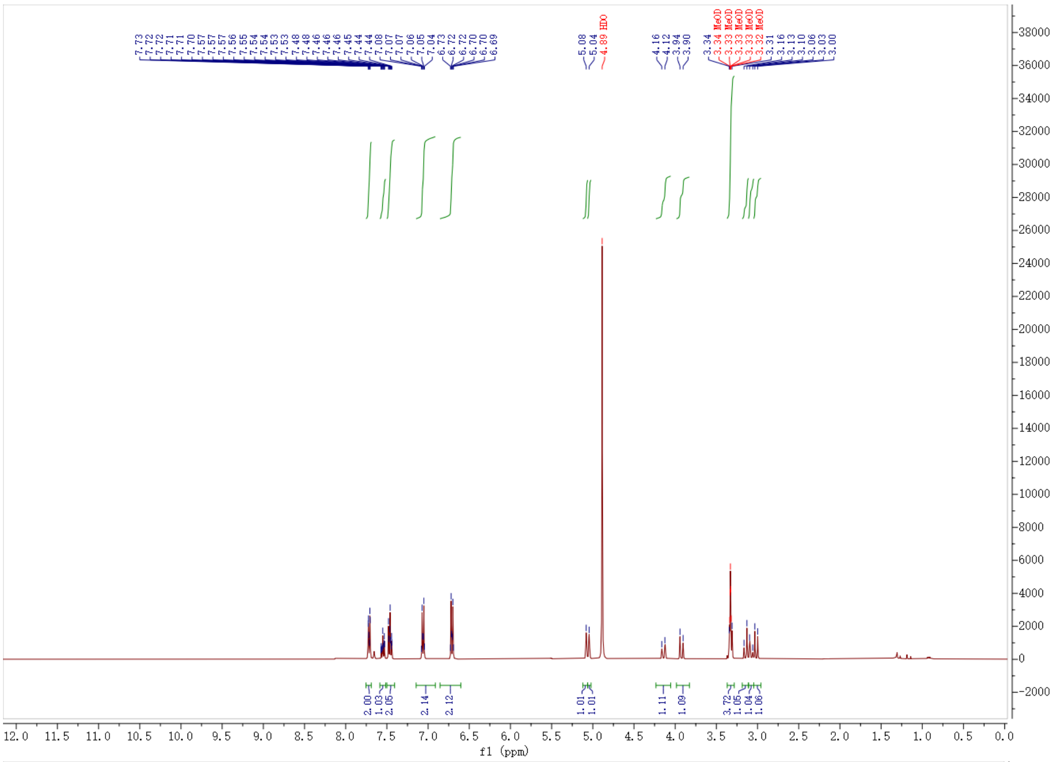
7.73
7.72
7.72
7.71
7.71
7.70
7.57
7.57
7.56
7.55
7.54
7.54
7.53
7.53
7.48
7.48
7.46
7.46
7.45
7.44
7.44
7.08
7.07
7.07
7.06
7.05
7.04
6.73
6.72
6.72
6.70
6.70
6.69
5.08
5.04
4.89 HDO
4.16
4.12
3.94
3.90
3.34
3.34 MeOD
3.33 MeOD
3.33 MeOD
3.33 MeOD
3.32 MeOD
3.32 MeOD
3.31
3.16
3.13
3.10
3.06
3.03
3.00
2.00
1.03
2.05
2.14
2.12
1.01
1.01
1.11
1.09
3.72
1.05
1.04
1.06
f1 (ppm)

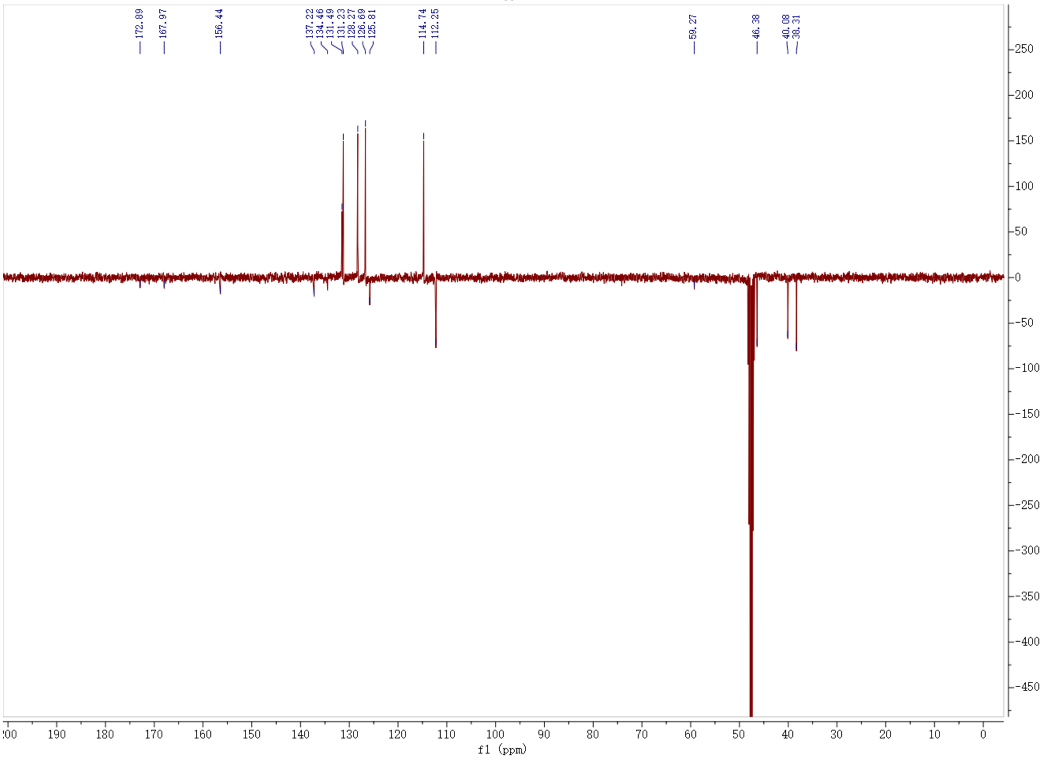
172.89
167.97
156.44
137.22
134.46
131.49
131.23
128.27
126.69
125.81
114.74
112.25
59.27
46.38
40.08
38.31
f1 (ppm)

**11**

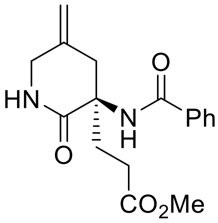
HN
O
N
H
O
Ph
CO2Me

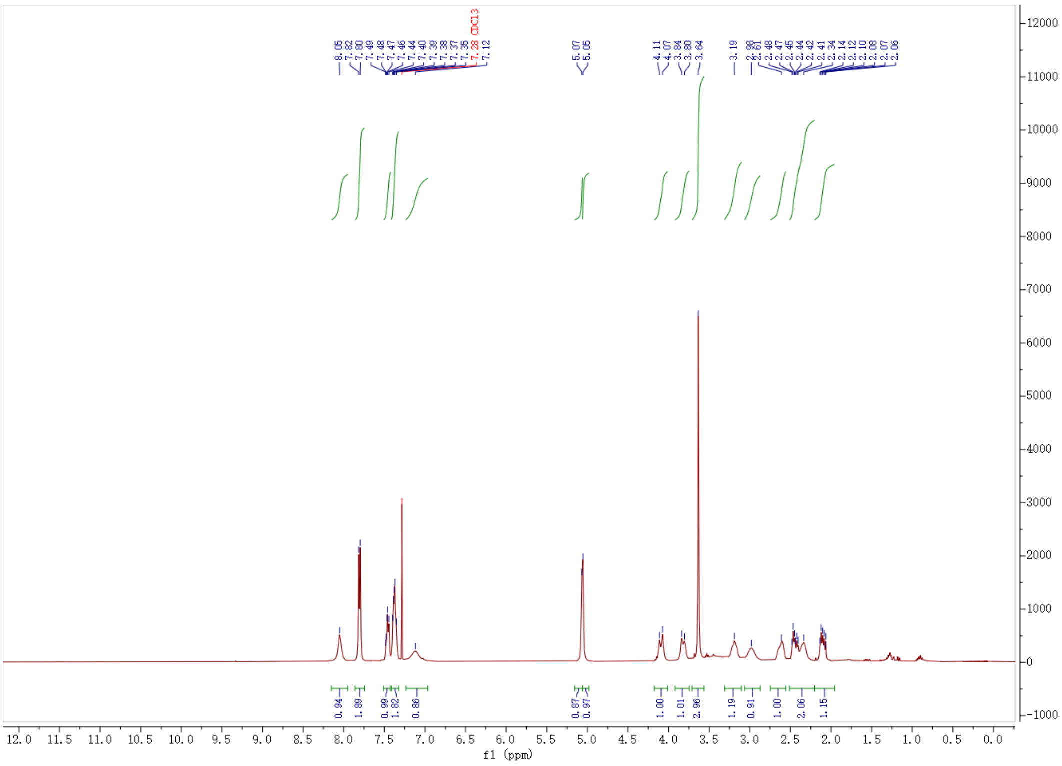
8.05
7.82
7.80
7.49
7.48
7.47
7.46
7.44
7.40
7.39
7.38
7.37
7.35
7.28 CDCl3
7.12
5.07
5.05
4.11
4.07
3.84
3.80
3.64
3.19
2.98
2.61
2.48
2.47
2.45
2.44
2.42
2.41
2.34
2.14
2.12
2.10
2.08
2.07
2.06
0.94
1.89
0.99
1.82
0.86
0.87
0.97
1.00
1.01
2.96
1.19
0.91
1.00
2.06
1.15
f1 (ppm)

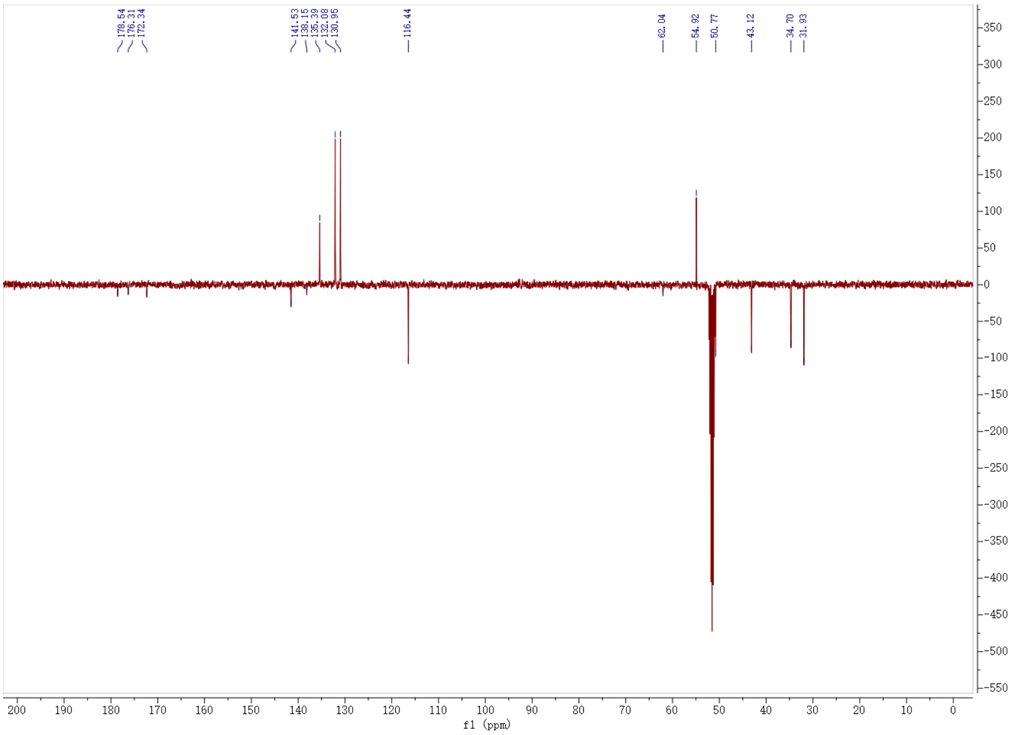
178.54
176.31
172.34
141.53
138.15
135.39
132.08
130.95
116.44
62.04
54.92
50.77
43.12
34.70
31.93
f1 (ppm)

**12**

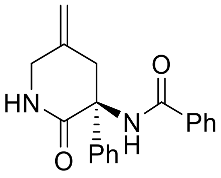
HN
O
Ph
N
H
O
Ph

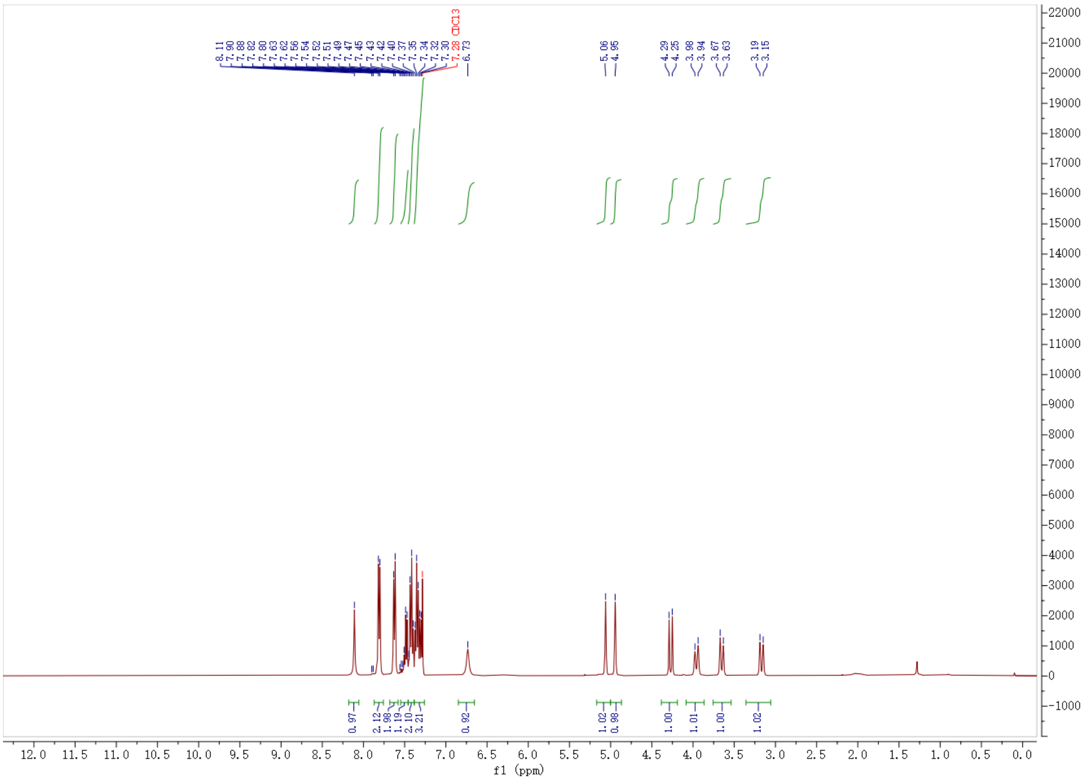
8.11
7.90
7.88
7.82
7.80
7.63
7.62
7.56
7.54
7.52
7.51
7.49
7.47
7.45
7.43
7.42
7.40
7.37
7.35
7.34
7.32
7.30
7.28 CDCl3
6.73
5.06
4.95
4.29
4.25
3.98
3.94
3.67
3.63
3.19
3.15
0.97
2.12
1.98
1.19
2.10
3.21
0.92
1.02
0.98
1.00
1.01
1.00
1.02
12.0
11.5
11.0
10.5
10.0
9.5
9.0
8.5
8.0
7.5
7.0
6.5
6.0
5.5
5.0
4.5
4.0
3.5
3.0
2.5
2.0
1.5
1.0
0.5
0.0
f1 (ppm)
22000
21000
20000
19000
18000
17000
16000
15000
14000
13000
12000
11000
10000
9000
8000
7000
6000
5000
4000
3000
2000
1000
0
-1000

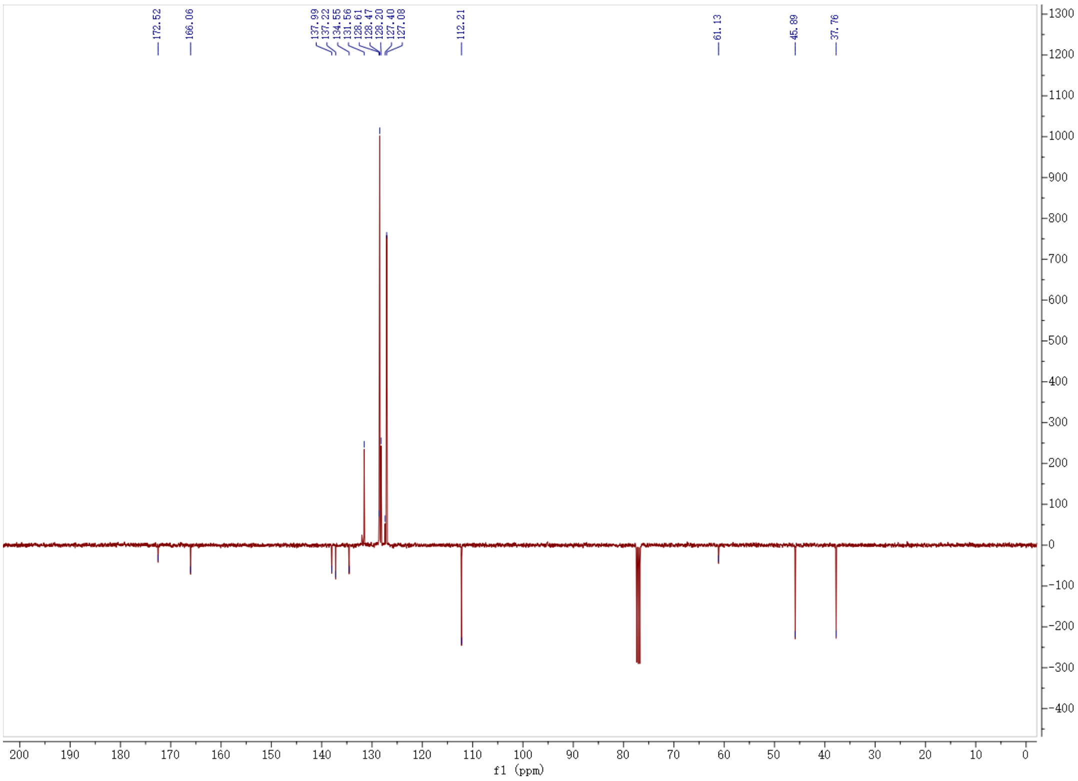
172.52
166.06
137.99
137.22
134.55
131.56
128.61
128.47
128.20
127.40
127.08
112.21
61.13
45.89
37.76
200
190
180
170
160
150
140
130
120
110
100
90
80
70
60
50
40
30
20
10
0
f1 (ppm)
1300
1200
1100
1000
900
800
700
600
500
400
300
200
100
0
-100
-200
-300
-400

**13**

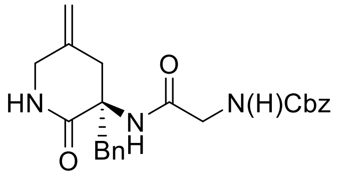

O
HN
N
N(H)Cbz
Bn
H
O


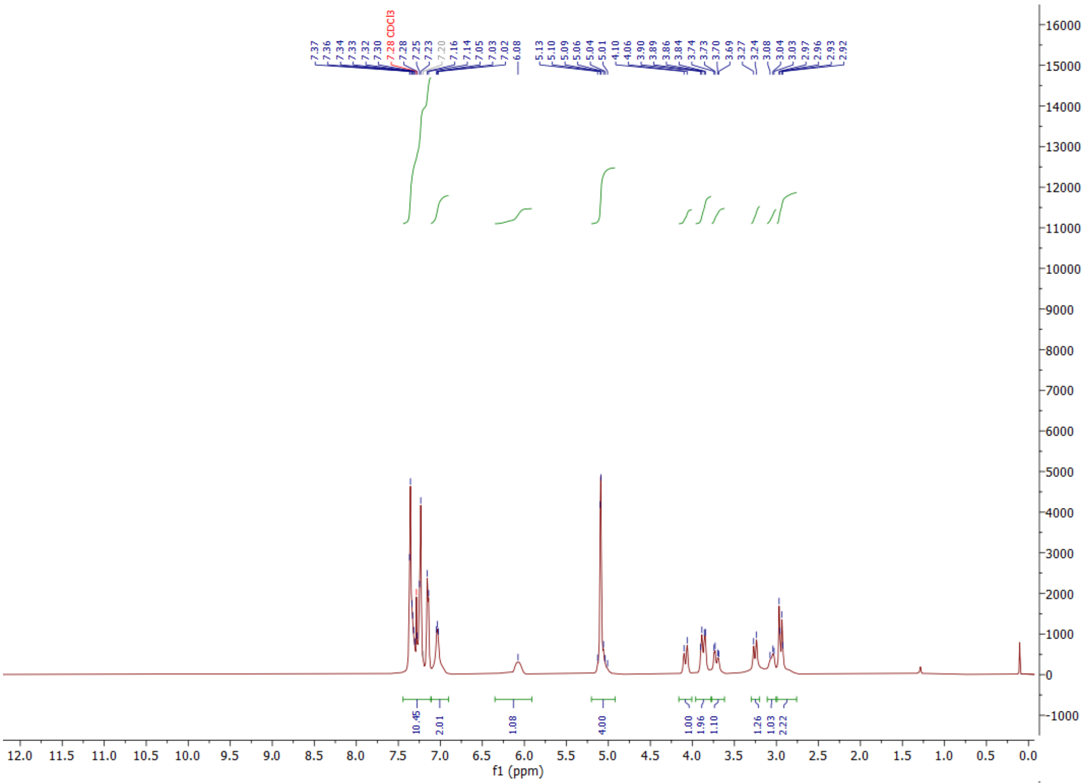

7.37
7.36
7.34
7.33
7.32
7.30
7.28 CDCl3
7.28
7.25
7.23
7.20
7.16
7.14
7.05
7.03
7.02
6.08
5.13
5.10
5.09
5.06
5.04
5.01
4.10
4.06
3.90
3.89
3.86
3.84
3.74
3.73
3.70
3.69
3.27
3.24
3.08
3.04
3.03
2.97
2.96
2.93
2.92
10.45
2.01
1.08
4.00
1.00
1.96
1.10
1.26
1.03
2.22
f1 (ppm)


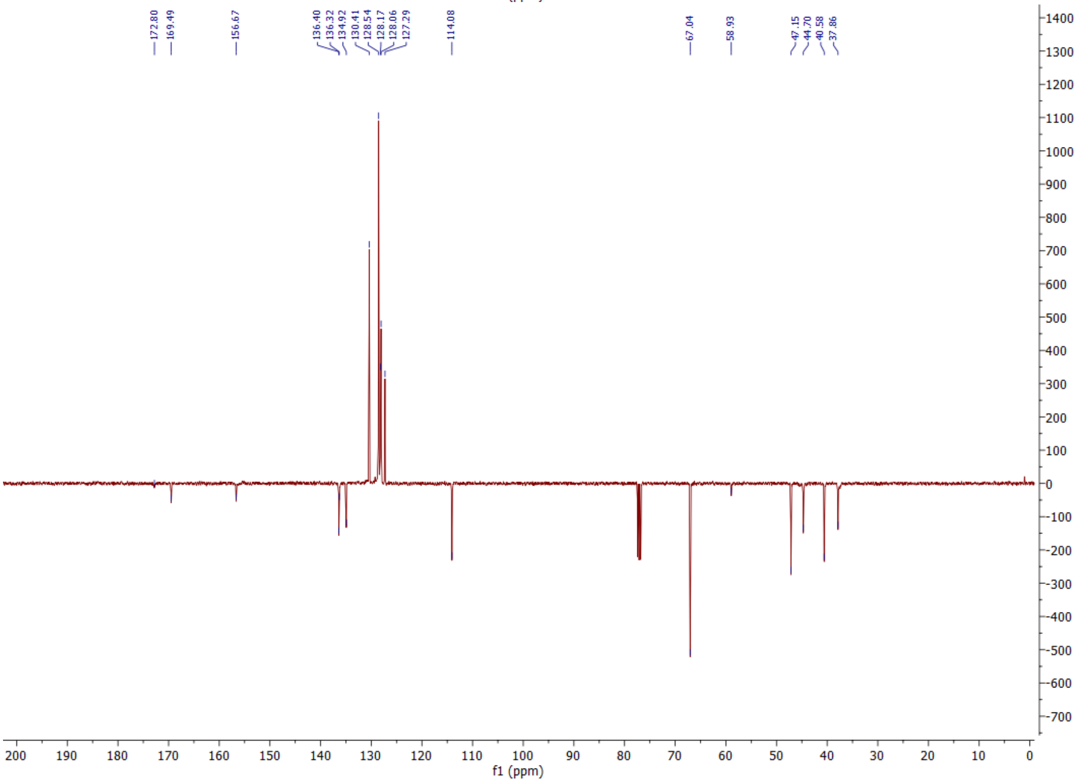

172.80
169.49
156.67
136.40
136.32
134.92
130.41
128.54
128.17
128.06
127.29
114.08
67.04
58.93
47.15
44.70
40.58
37.86
f1 (ppm)

**14**

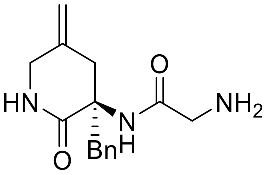
HN
O
NH2
N
H
Bn
O

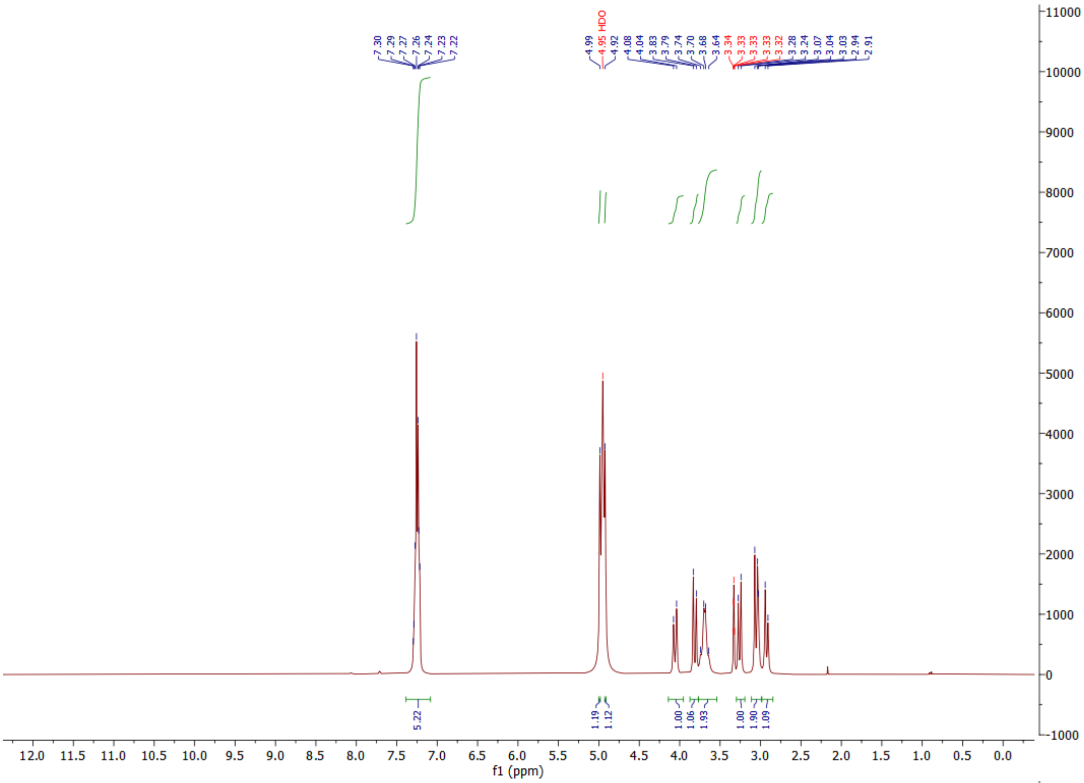
7.30
7.29
7.27
7.26
7.24
7.23
7.22
4.99
4.95 HDO
4.92
4.08
4.04
3.83
3.79
3.74
3.70
3.68
3.64
3.34
3.33
3.33
3.33
3.33
3.32
3.28
3.24
3.07
3.04
3.03
2.94
2.91
5.22
1.19
1.12
1.00
1.06
1.93
1.00
1.90
1.09
f1 (ppm)

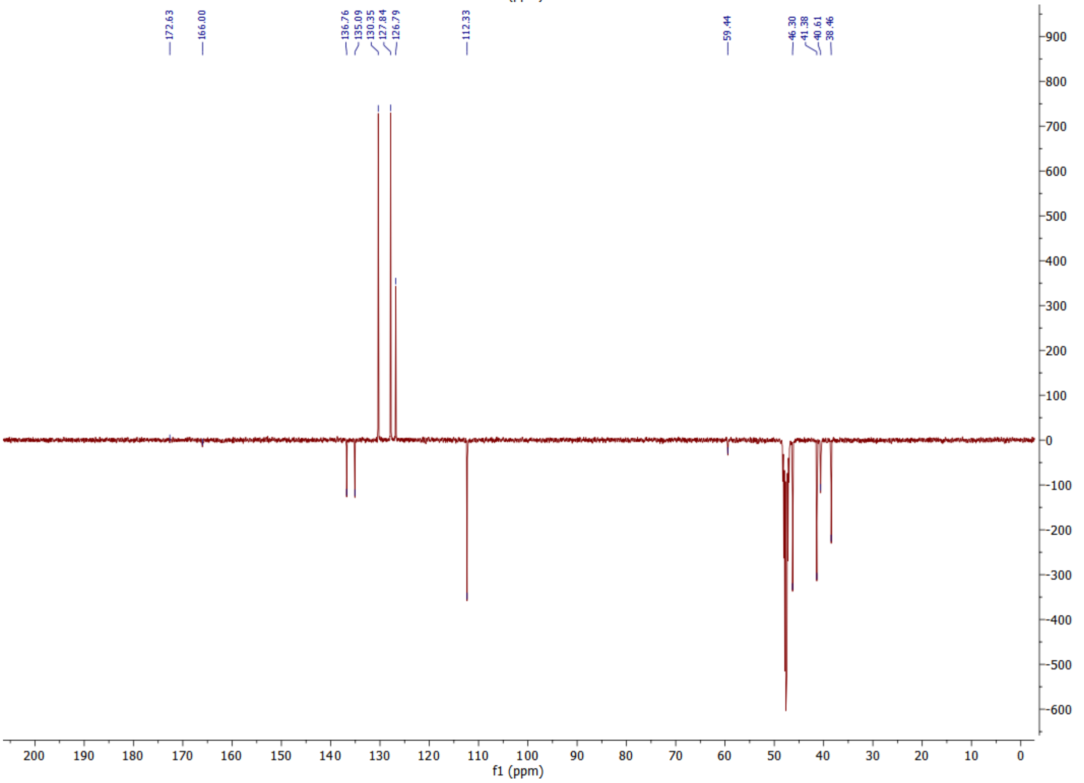
172.63
166.00
136.76
135.09
130.35
127.84
126.79
112.33
59.44
46.30
41.38
40.61
38.46
f1 (ppm)

**15**

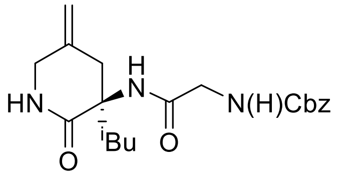


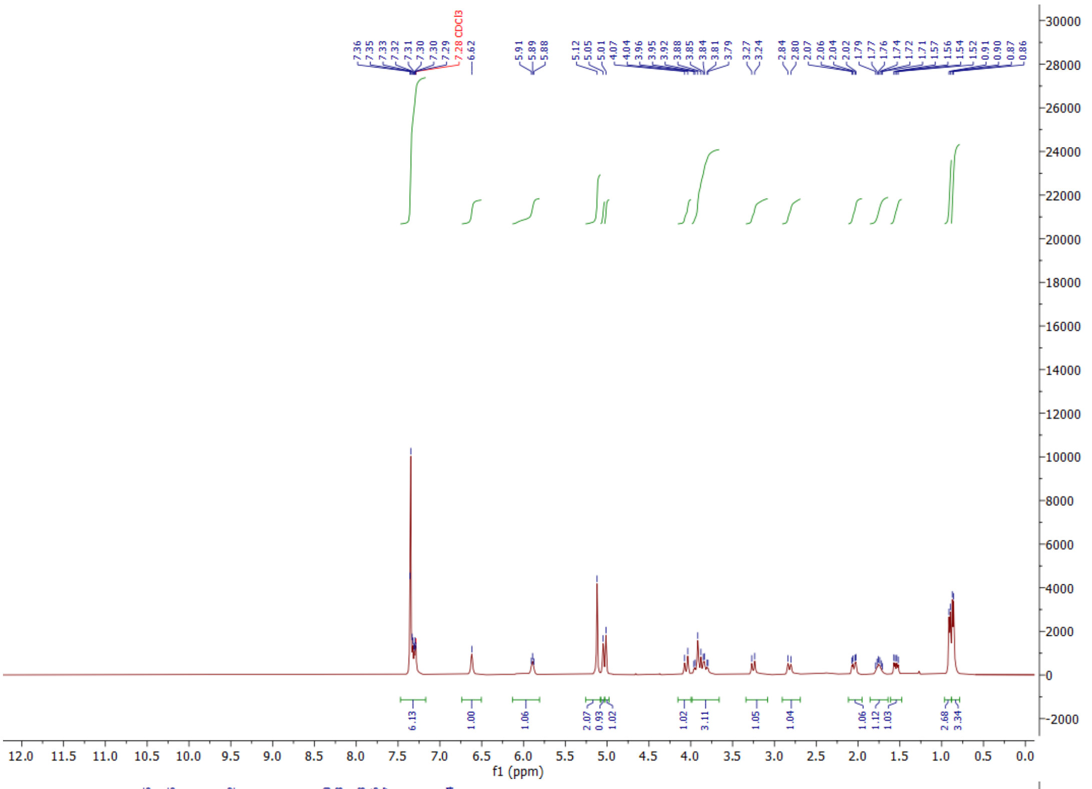


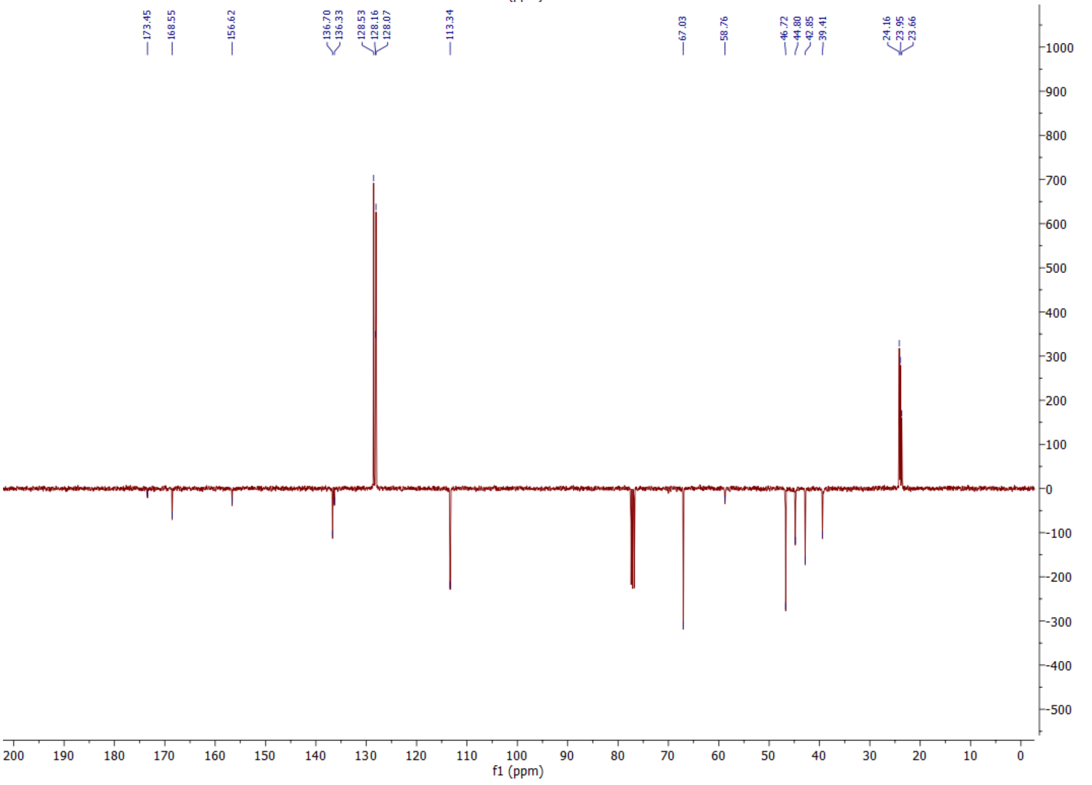

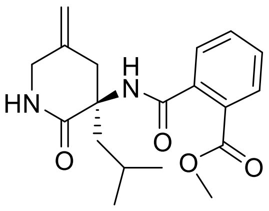
HN
O
H
N
O
O
O

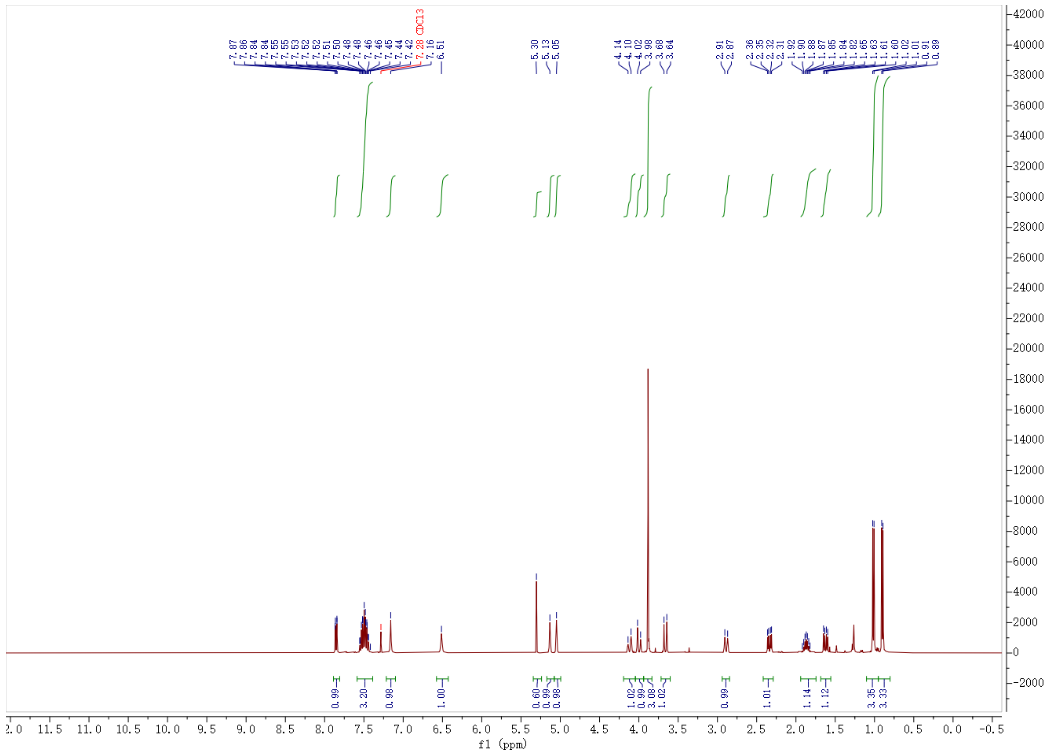
7.87 7.86 7.84 7.84 7.55 7.55 7.53 7.52 7.52 7.51 7.50 7.48 7.48 7.46 7.46 7.45 7.44 7.42 7.28 CDCl3 7.16 6.51
5.30 5.13 5.05
4.14 4.10 4.02 3.98 3.68 3.64
2.91 2.87 2.36 2.35 2.32 2.31 1.92 1.90 1.88 1.87 1.85 1.84 1.82 1.65 1.63 1.61 1.60 1.02 1.01 0.91 0.89
0.99 3.20 0.98 1.00 0.60 0.99 0.98 1.02 0.99 3.08 1.02 0.99 1.01 1.14 1.12 3.35 3.33
f1 (ppm)

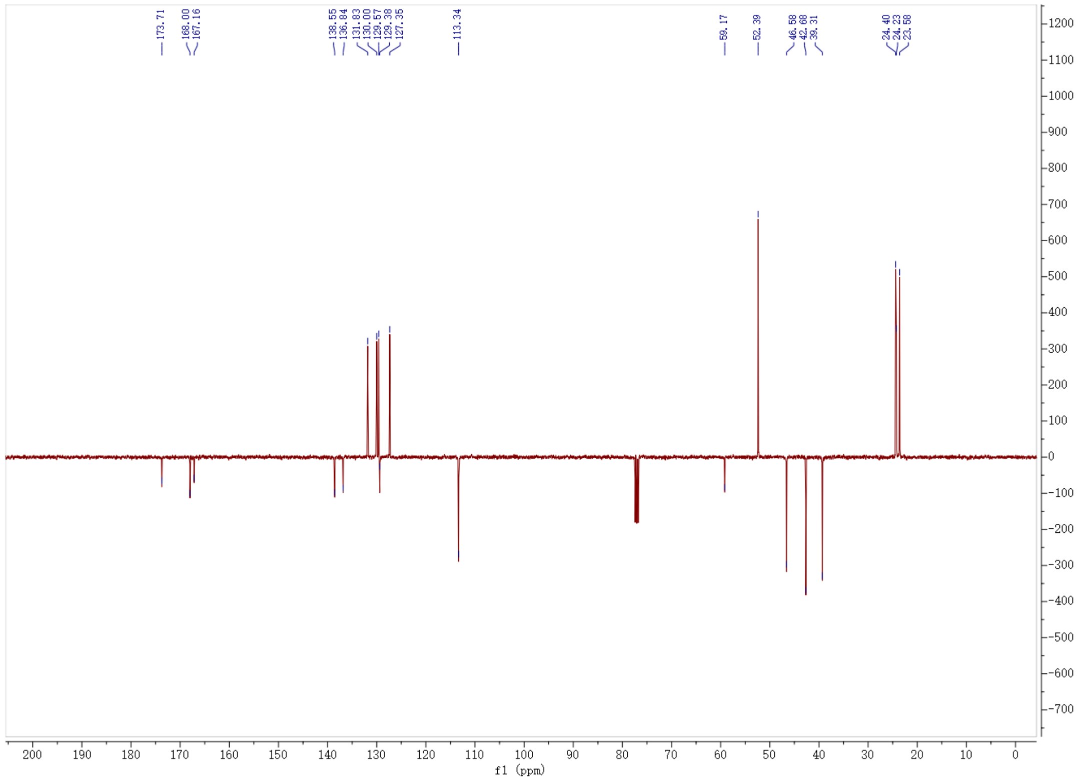
173.71
168.00 167.16
138.55 136.84 131.83 130.00 129.57 129.38 127.35
113.34
59.17
52.39
46.58 42.68 39.31
24.40 24.23 23.58
f1 (ppm)

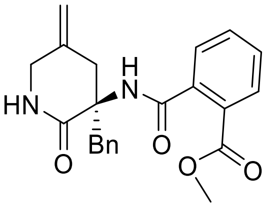

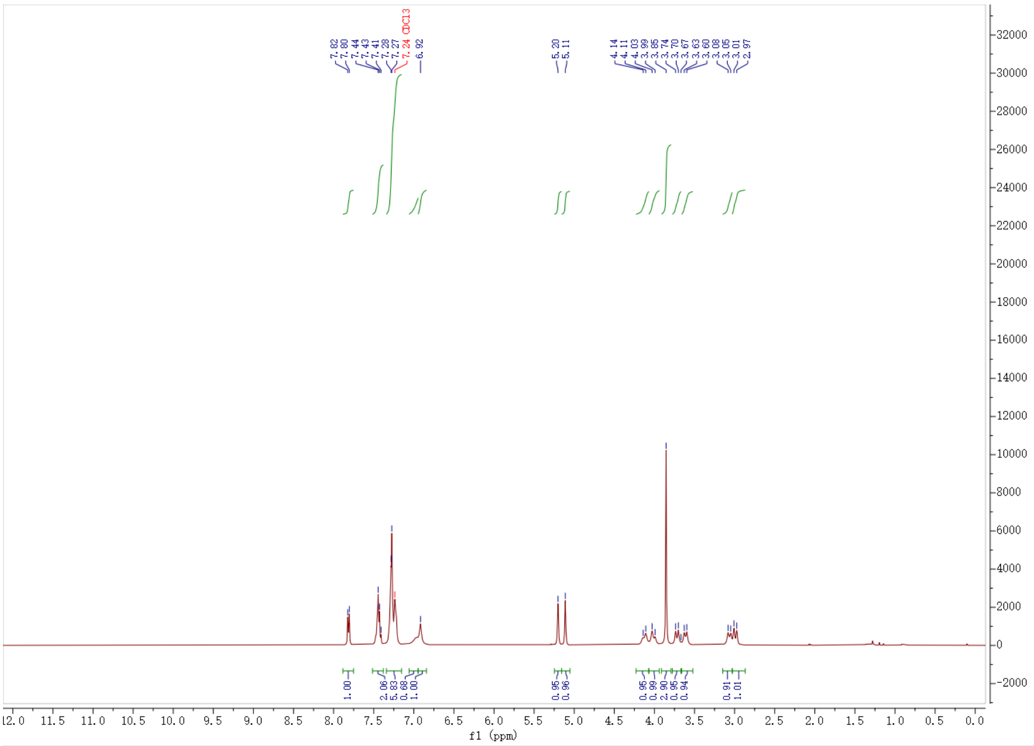

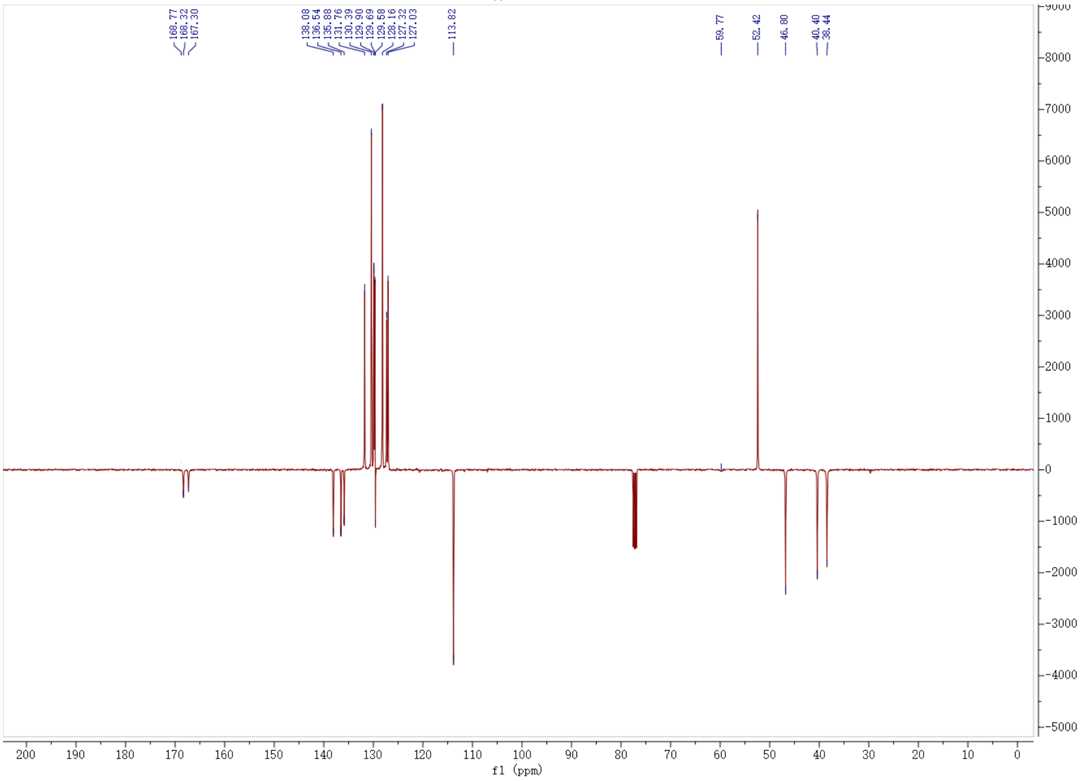

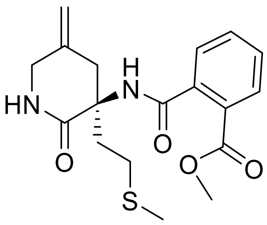
HN
O
H
N
O
O
O
S

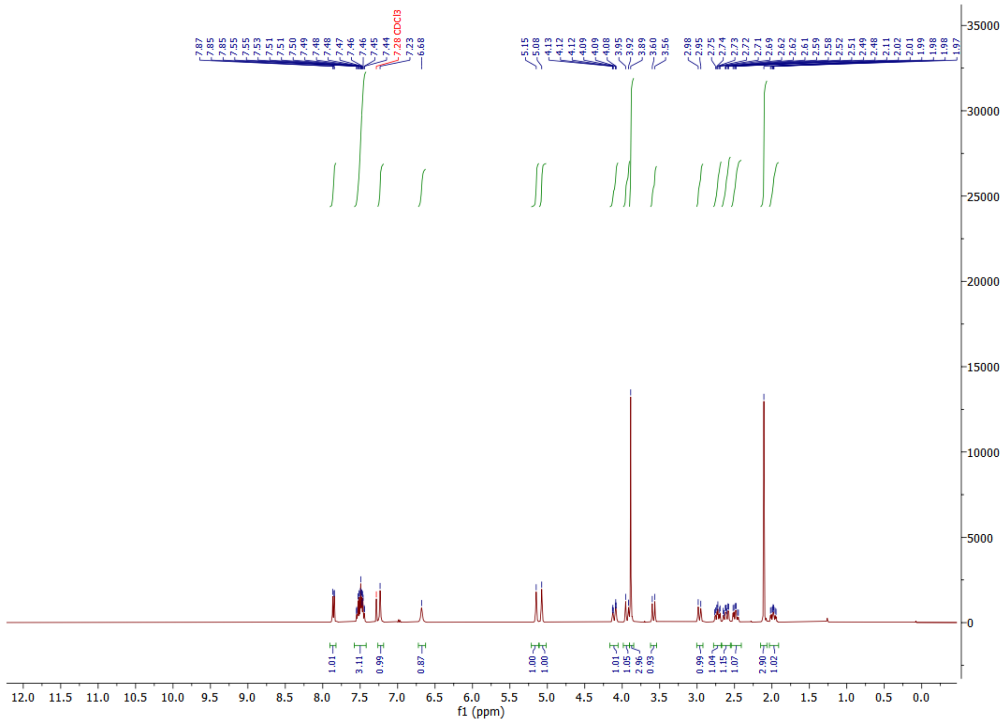
7.87 7.85 7.85 7.55 7.55 7.53 7.51 7.51 7.50 7.49 7.48 7.48 7.47 7.46 7.45 7.45 7.44 7.28 CDCl3 7.23 6.68
5.15 5.08 4.13 4.12 4.12 4.09 4.09 4.08 3.95 3.92 3.89 3.60 3.56
2.98 2.95 2.75 2.74 2.73 2.72 2.71 2.69 2.62 2.62 2.61 2.59 2.58 2.52 2.51 2.49 2.48 2.11 2.02 2.01 1.99 1.98 1.98 1.97
1.01 3.11 0.99 0.87 1.00 1.00 1.01 1.05 2.96 0.93 0.99 1.04 1.15 1.07 2.90 1.02
f1 (ppm)

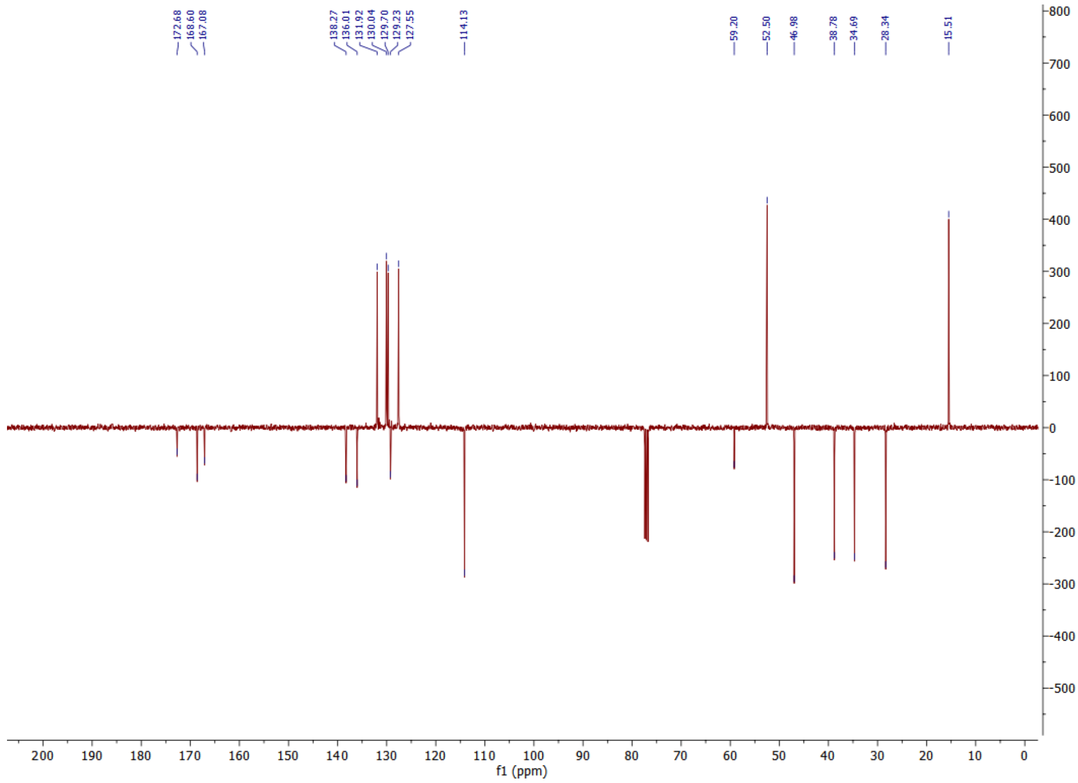
172.68 168.60 167.08
138.27 136.01 131.92 130.04 129.70 129.23 127.55
114.13
59.20 52.50 46.98 38.78 34.69 28.34 15.51
f1 (ppm)

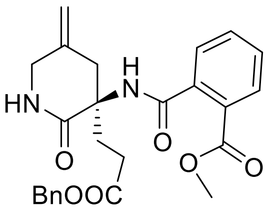

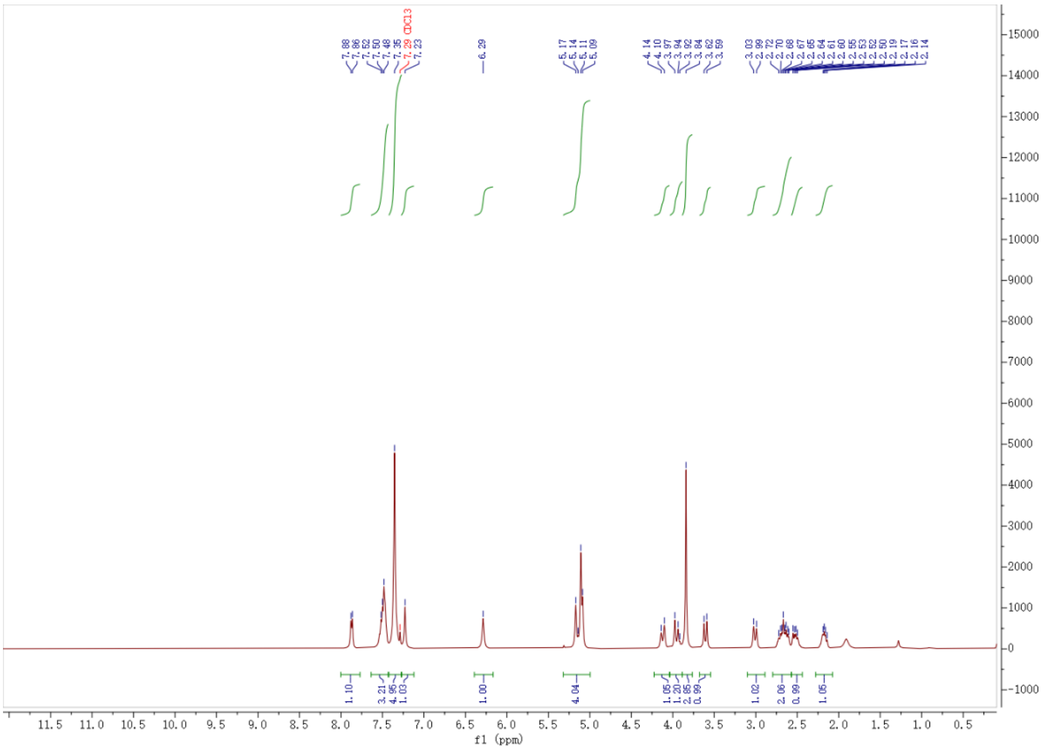

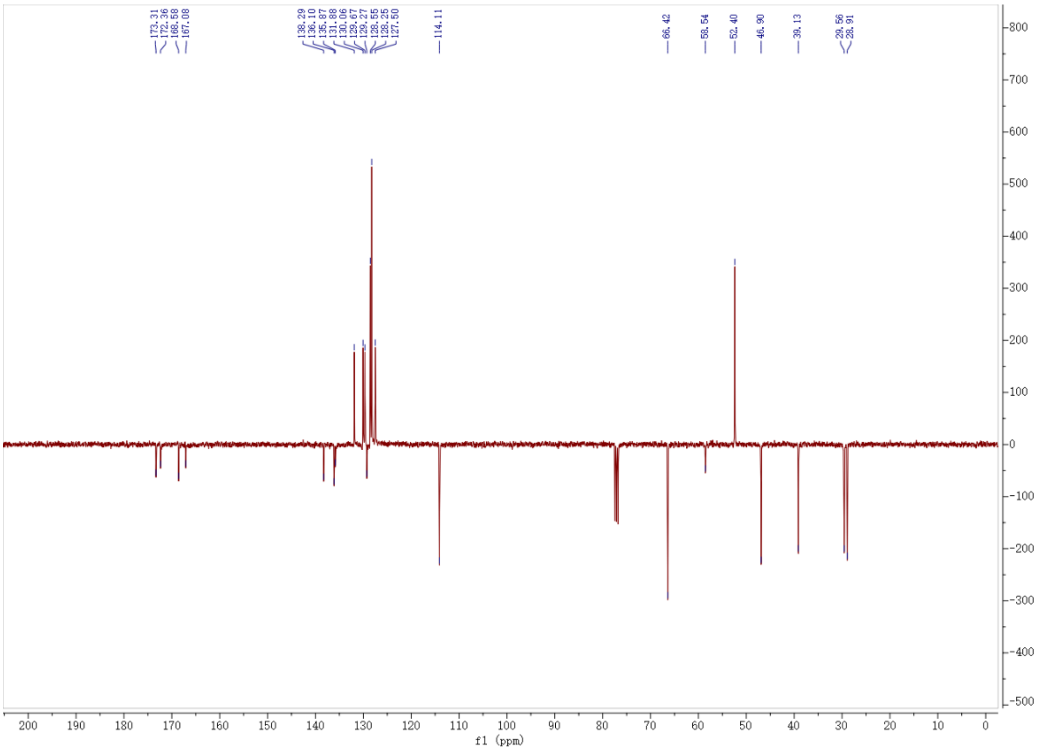

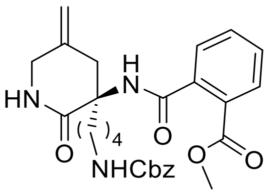
HN
H
N
O
O
O
O
4
NHCbz

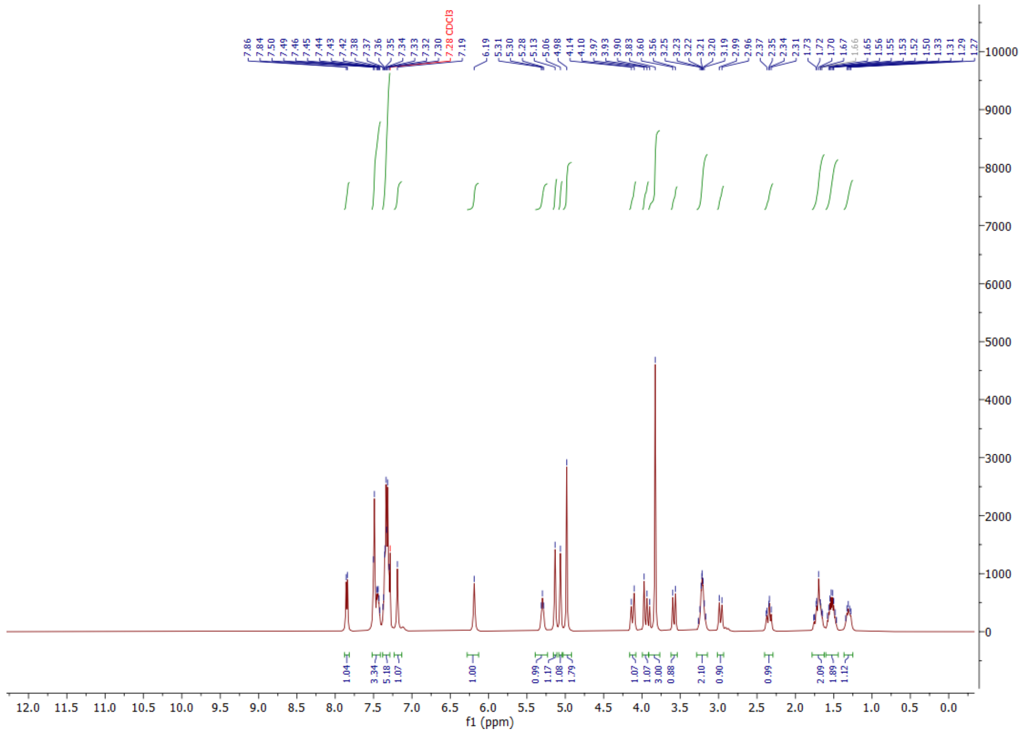
7.86 7.84 7.50 7.49 7.46 7.45 7.44 7.43 7.42 7.38 7.37 7.36 7.35 7.34 7.33 7.32 7.30 7.28 CDCl3 7.19
6.19 5.31 5.30 5.28 5.13 5.06 4.98 4.14 4.10 3.97 3.93 3.90 3.83 3.60 3.56 3.25 3.23 3.22 3.21 3.20 3.19 2.99 2.96 2.37 2.35 2.34 2.31 1.73 1.72 1.70 1.67 1.66 1.65 1.56 1.55 1.53 1.52 1.50 1.33 1.31 1.29 1.27
1.04 3.34 5.18 1.07 1.00 0.99 1.17 1.08 1.79 1.07 1.07 3.00 0.88 2.10 0.90 0.99 2.09 1.89 1.12
f1 (ppm)

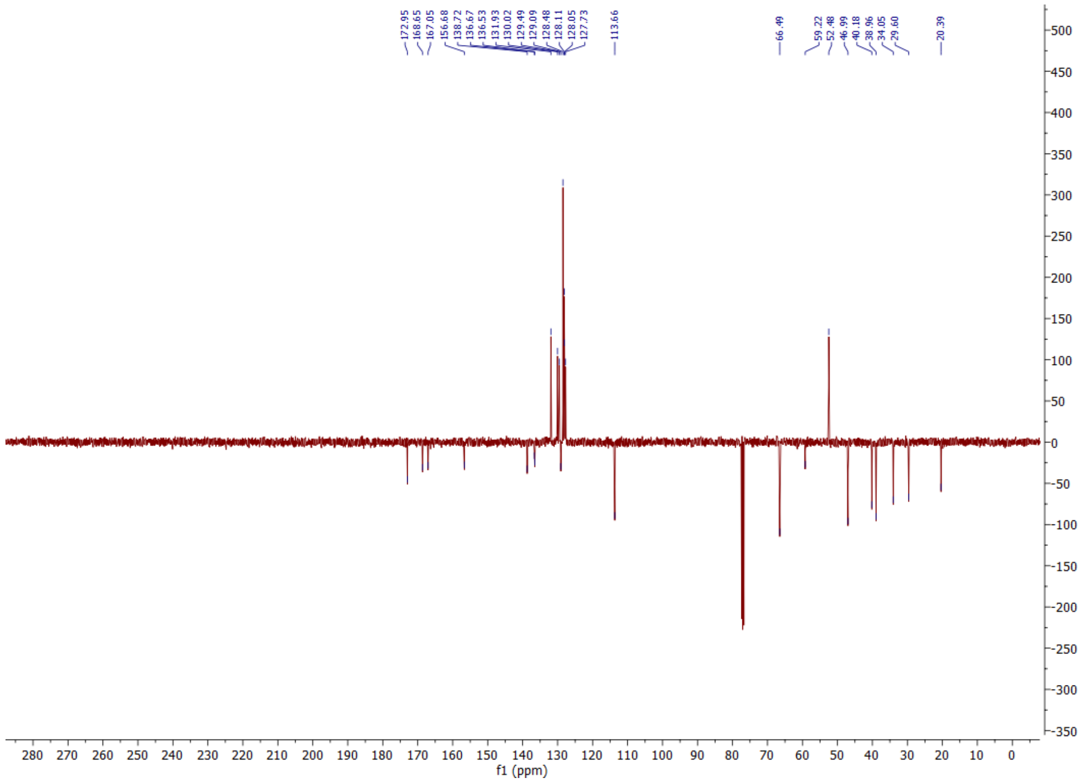
172.95 168.65 167.05 156.68 138.72 136.67 136.53 131.93 130.02 129.49 129.09 128.48 128.11 128.05 127.73 113.66
66.49 59.22 52.48 46.99 40.18 38.96 34.05 29.60 20.39
f1 (ppm)

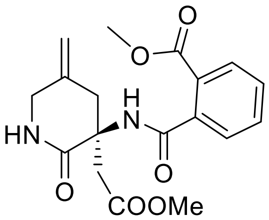
HN
O
H
N
O
O
O
COOMe

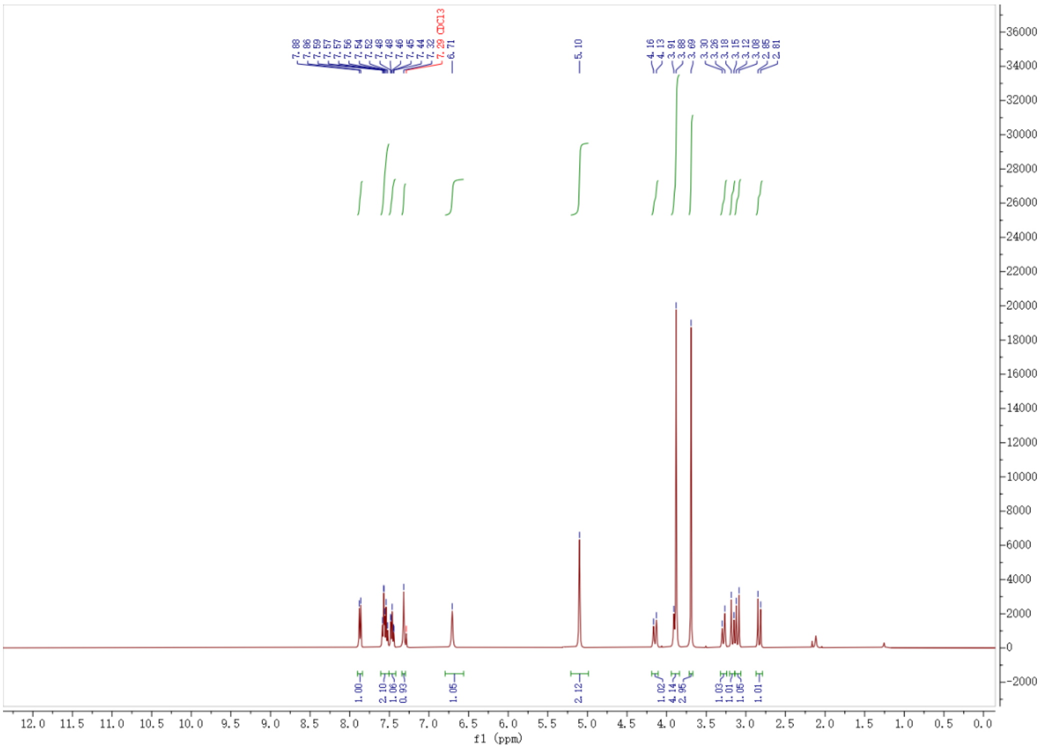
7.88 7.86 7.59 7.57 7.57 7.56 7.54 7.52 7.48 7.48 7.46 7.45 7.44 7.32 7.29 CDCl3 6.71
5.10
4.16 4.13 3.91 3.88 3.69 3.30 3.26 3.18 3.15 3.12 3.08 2.85 2.81
1.00 2.10 1.06 0.93 1.05 2.12 1.02 4.14 2.95 1.03 1.01 1.05 1.01
f1 (ppm)

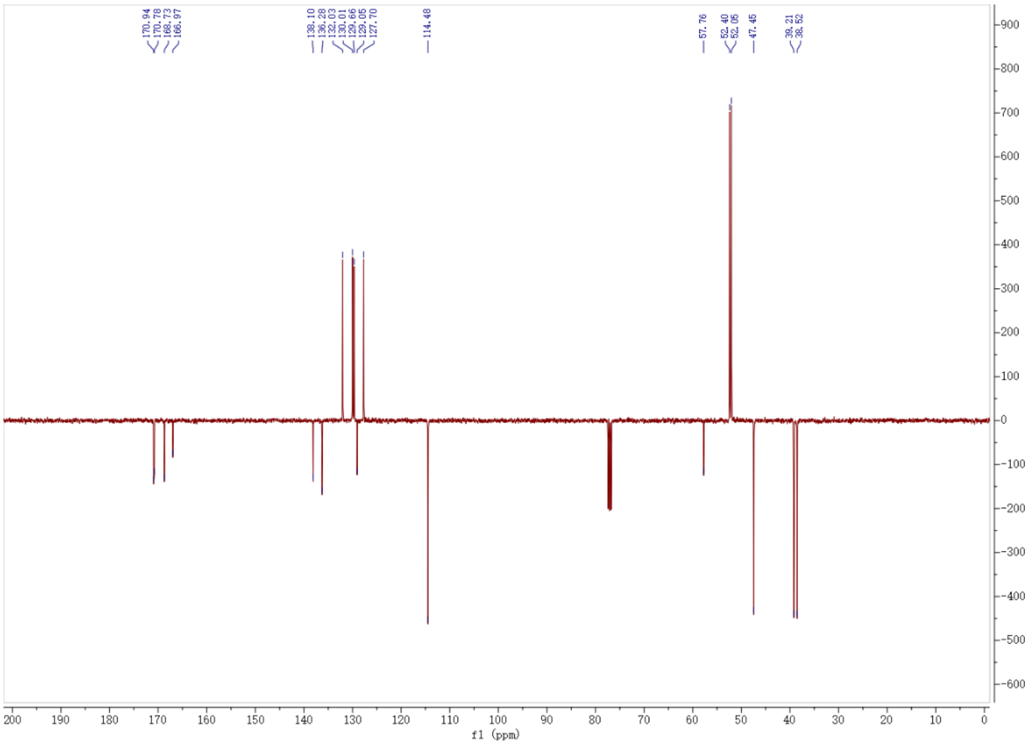
170.94 170.78 168.73 166.97
138.10 136.28 132.03 130.01 129.66 129.05 127.70
114.48
57.76 52.40 52.05 47.45 39.21 38.52
f1 (ppm)

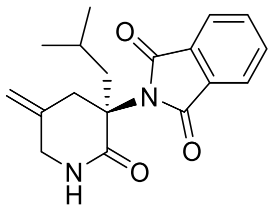
O
N
O
N
H
O

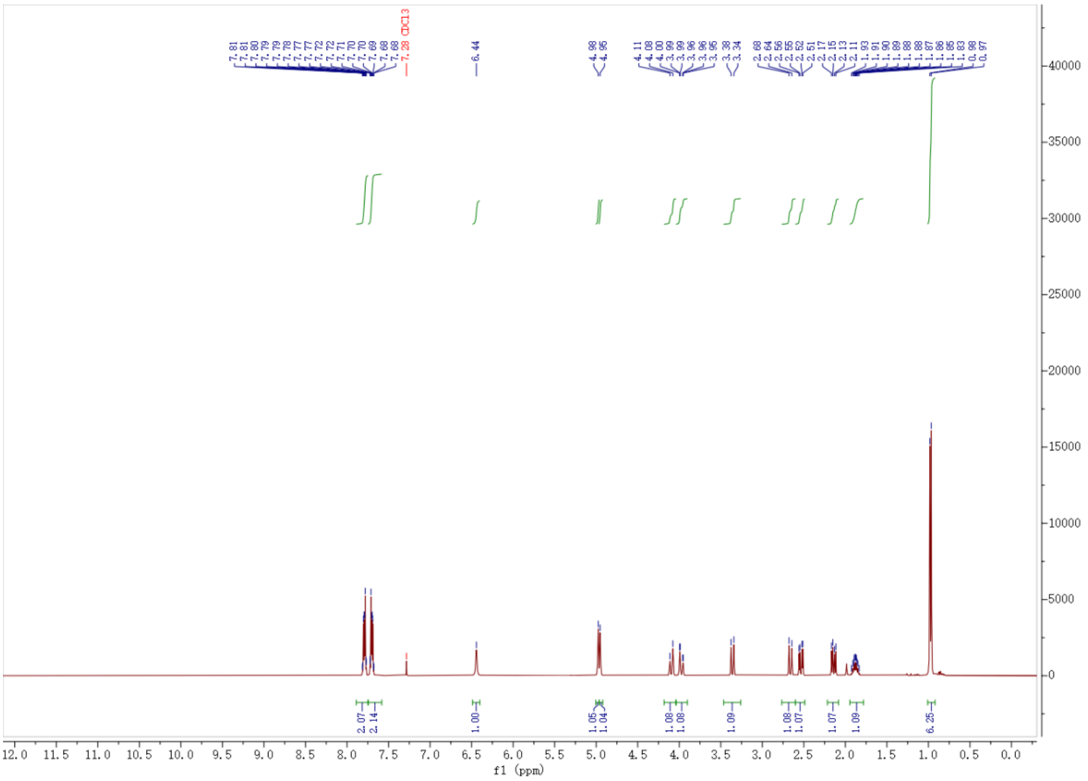
CDCl3
6.44
2.07
2.14
1.00
1.05
1.04
1.08
1.08
1.09
1.08
1.07
1.07
1.09
6.25
f1 (ppm)

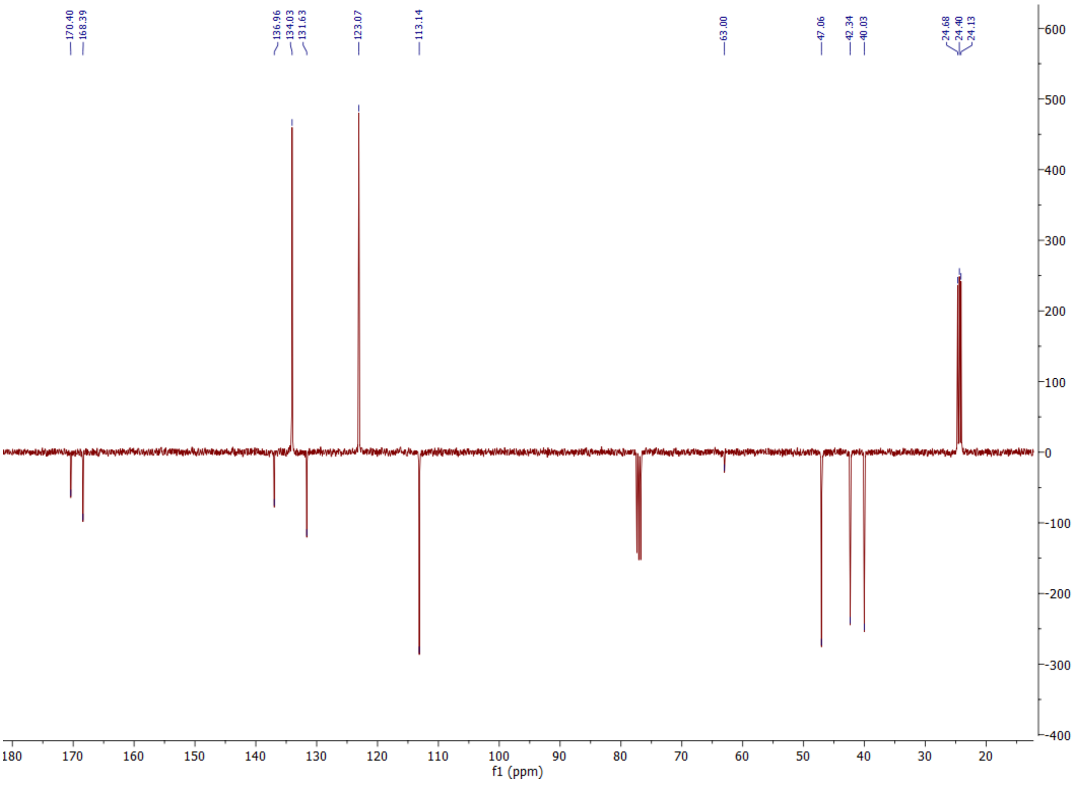
170.40
168.39
136.96
134.03
131.63
123.07
113.14
63.00
47.06
42.34
40.03
24.68
24.40
24.13
f1 (ppm)

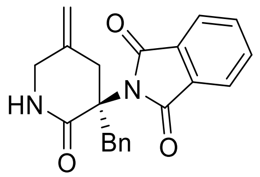
HN
N
O
O
O
Bn

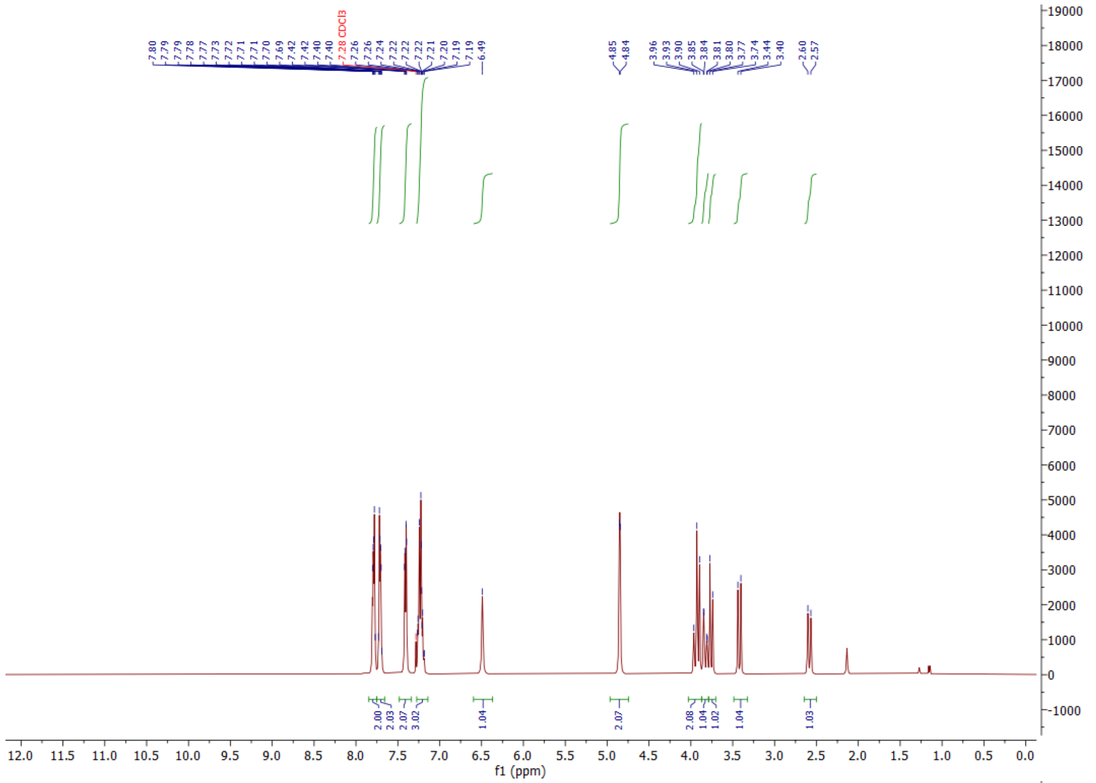
CDCl3
f1 (ppm)

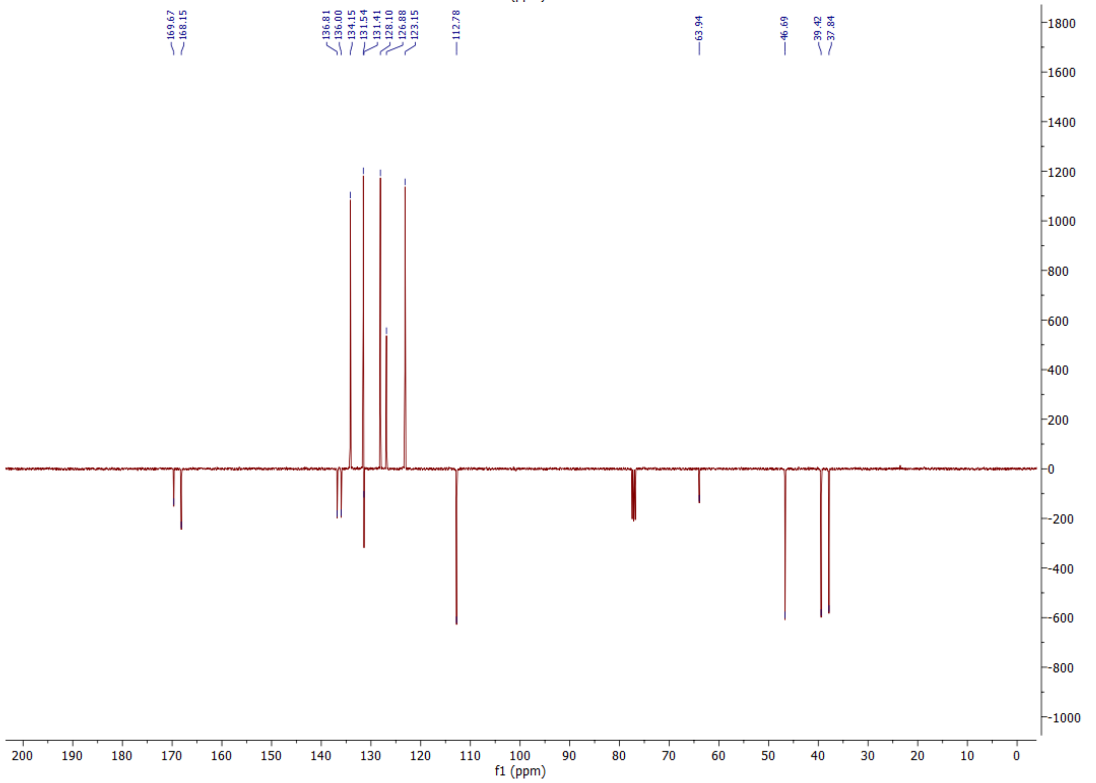
169.67
168.15
136.81
136.00
134.15
131.54
131.41
128.10
126.88
123.15
112.78
63.94
46.69
39.42
37.84
f1 (ppm)

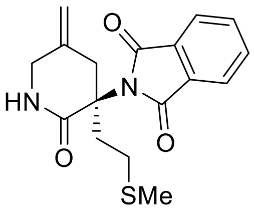
O
HN
N
O
O
SMe

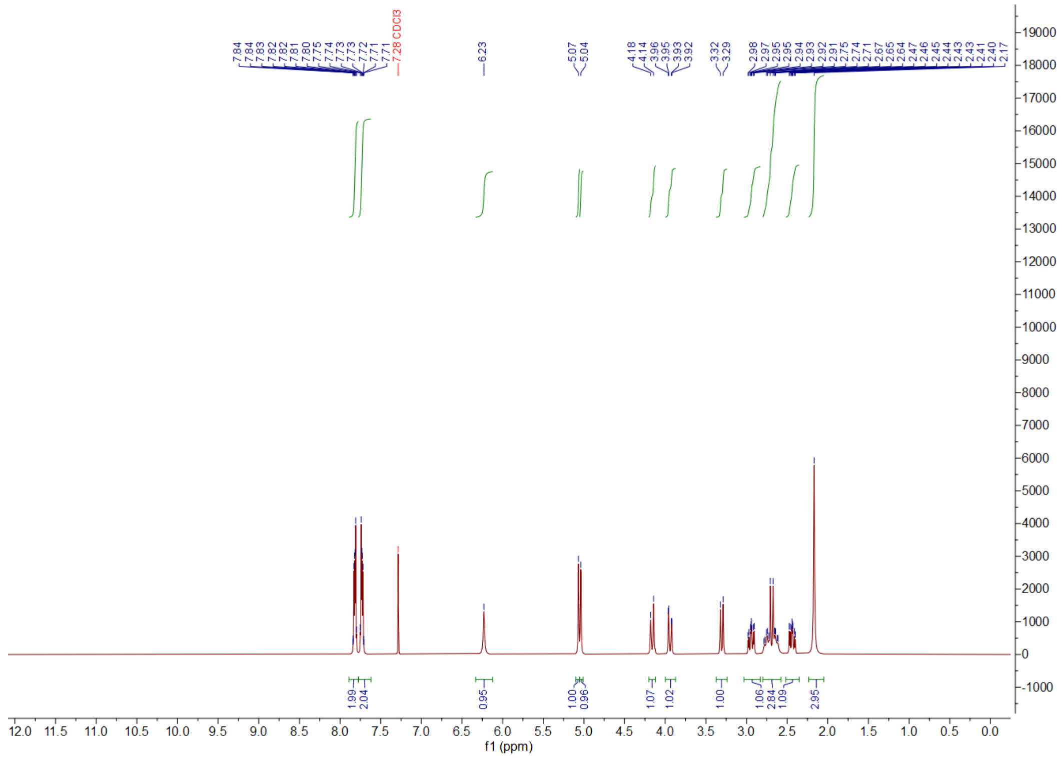
7.84
7.84
7.83
7.82
7.82
7.81
7.80
7.75
7.74
7.73
7.73
7.72
7.71
7.28 CDCl3
6.23
5.07
5.04
4.18
4.14
3.96
3.95
3.93
3.92
3.32
3.29
2.98
2.97
2.96
2.95
2.94
2.93
2.92
2.91
2.75
2.74
2.71
2.67
2.65
2.64
2.47
2.46
2.45
2.44
2.43
2.43
2.41
2.40
2.17
1.99
2.04
0.95
1.00
0.96
1.07
1.02
1.00
1.06
2.84
1.09
2.95
f1 (ppm)

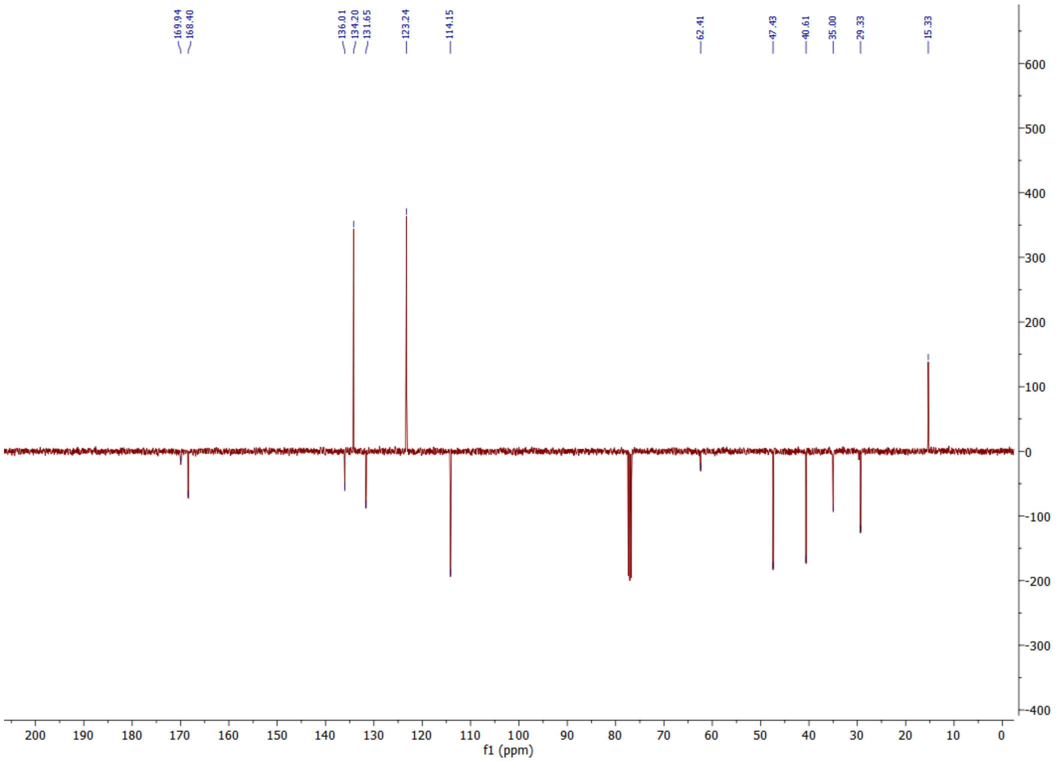
169.94
168.40
136.01
134.20
131.65
123.24
114.15
62.41
47.43
40.61
35.00
29.33
15.33
f1 (ppm)

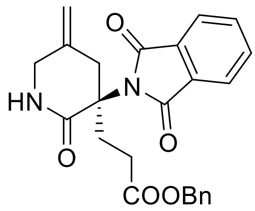
HN
N
O
O
O
COOBn

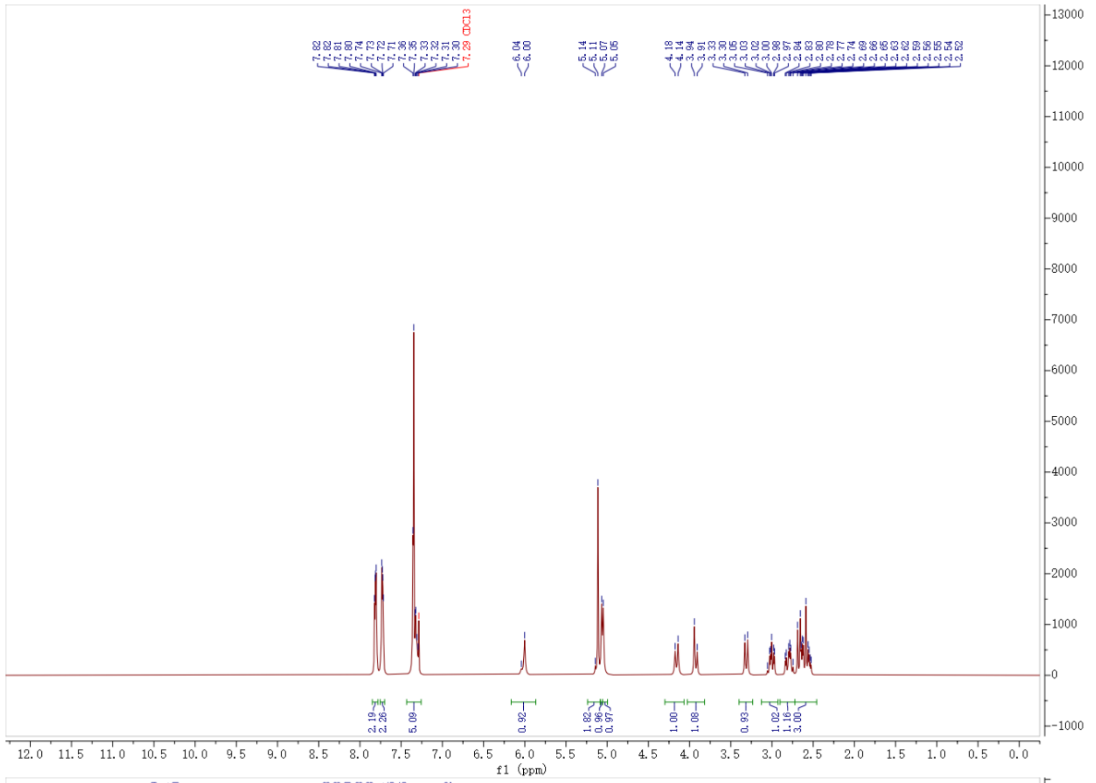

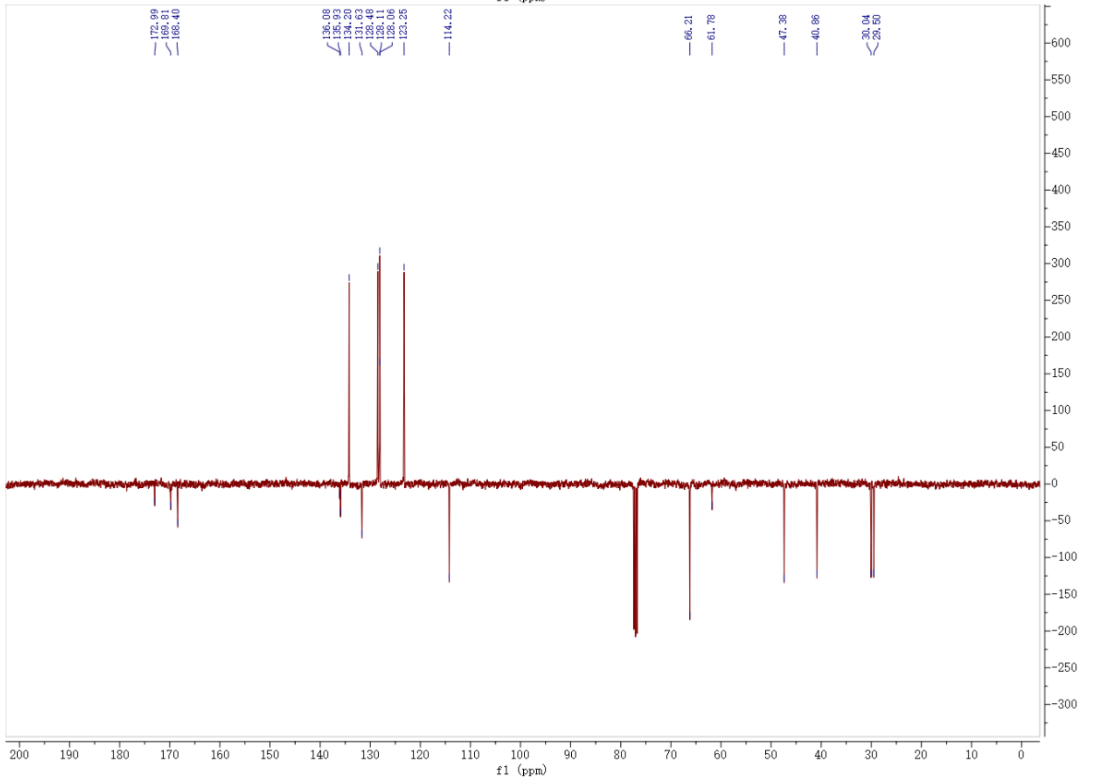

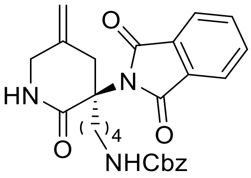
HN
O
N
O
O
4
NHCbz

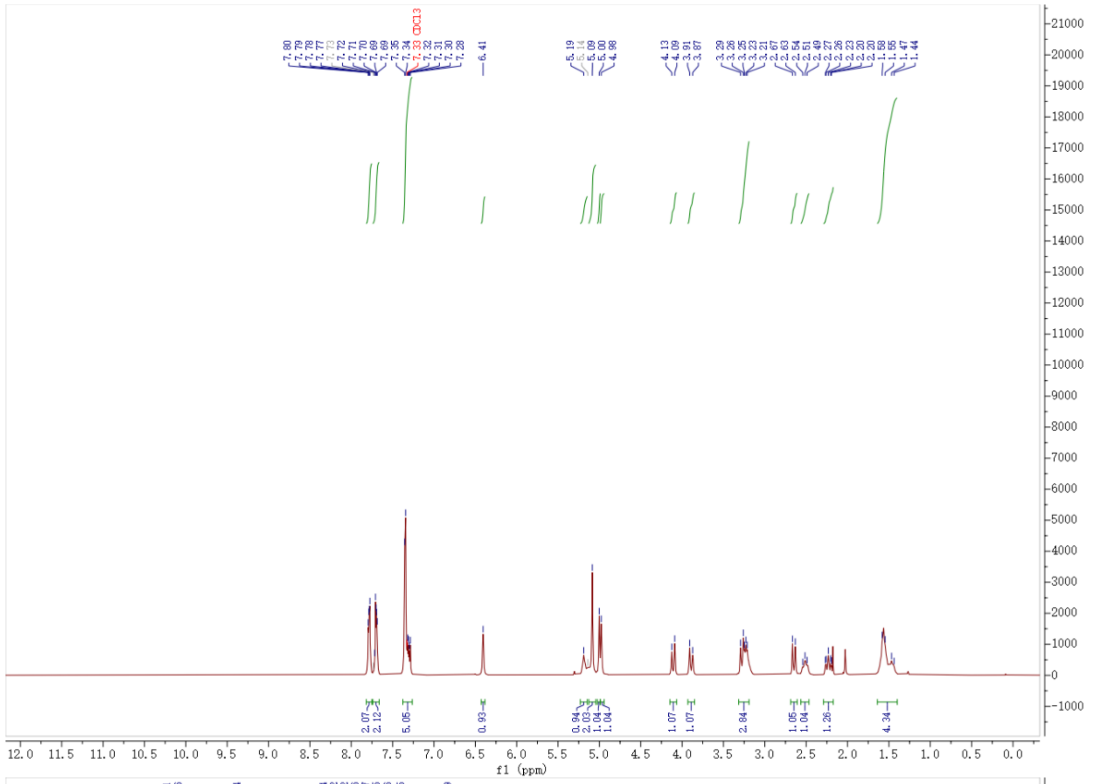
f1 (ppm)

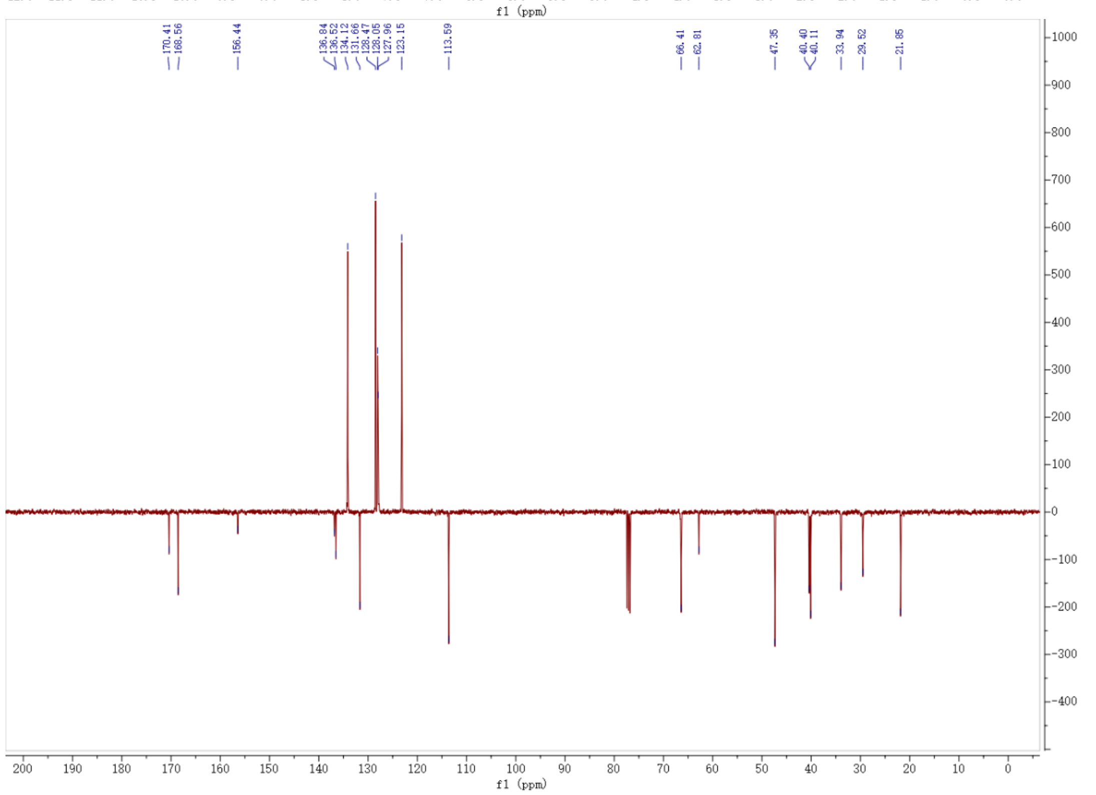
f1 (ppm)

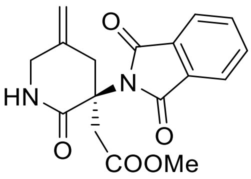
O
HN
N
O
O
COOMe

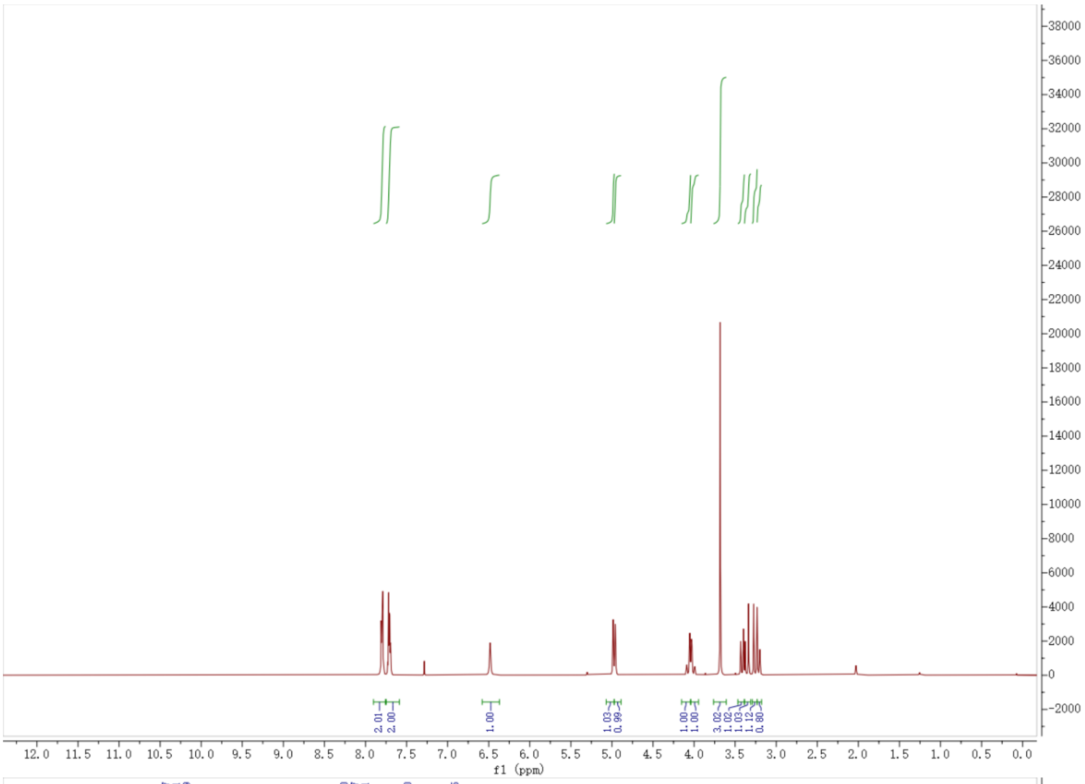
2.01
2.00
1.00
1.03
0.99
1.00
1.00
3.02
1.02
1.03
1.12
0.80
f1 (ppm)

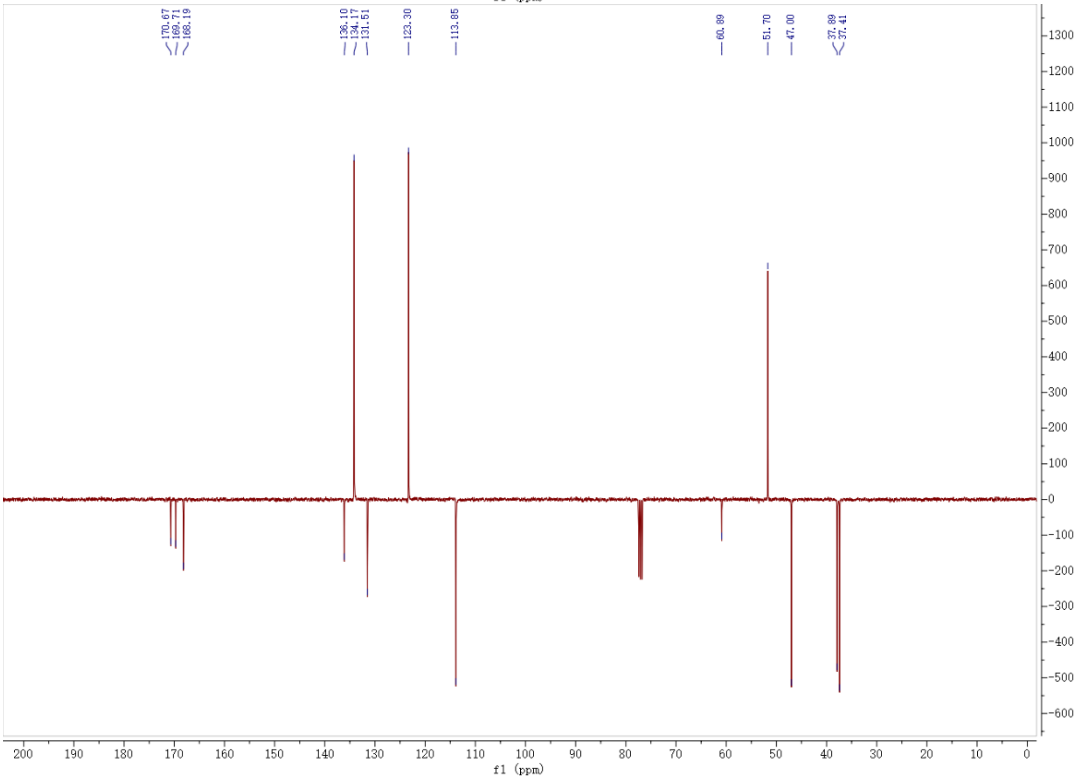
170.67
169.71
168.19
136.10
134.17
131.51
123.30
113.85
60.89
51.70
47.00
37.89
37.41
f1 (ppm)

**16**

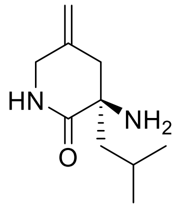
HN
NH2
O

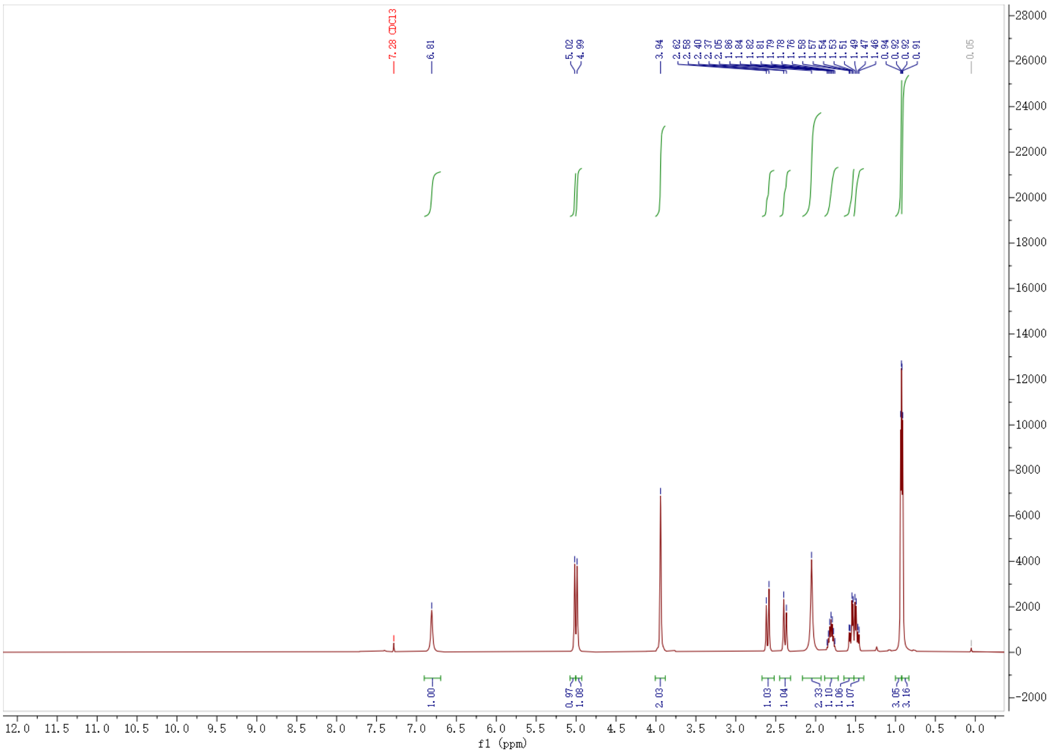
7.28 CDCl3
6.81
5.02
4.99
3.94
2.62
2.58
2.40
2.37
2.05
1.86
1.84
1.82
1.81
1.79
1.78
1.76
1.58
1.57
1.54
1.53
1.51
1.49
1.47
1.46
0.94
0.92
0.92
0.91
0.05
1.00
0.97
1.08
2.03
1.03
1.04
2.33
1.10
1.06
1.07
3.05
3.16
f1 (ppm)

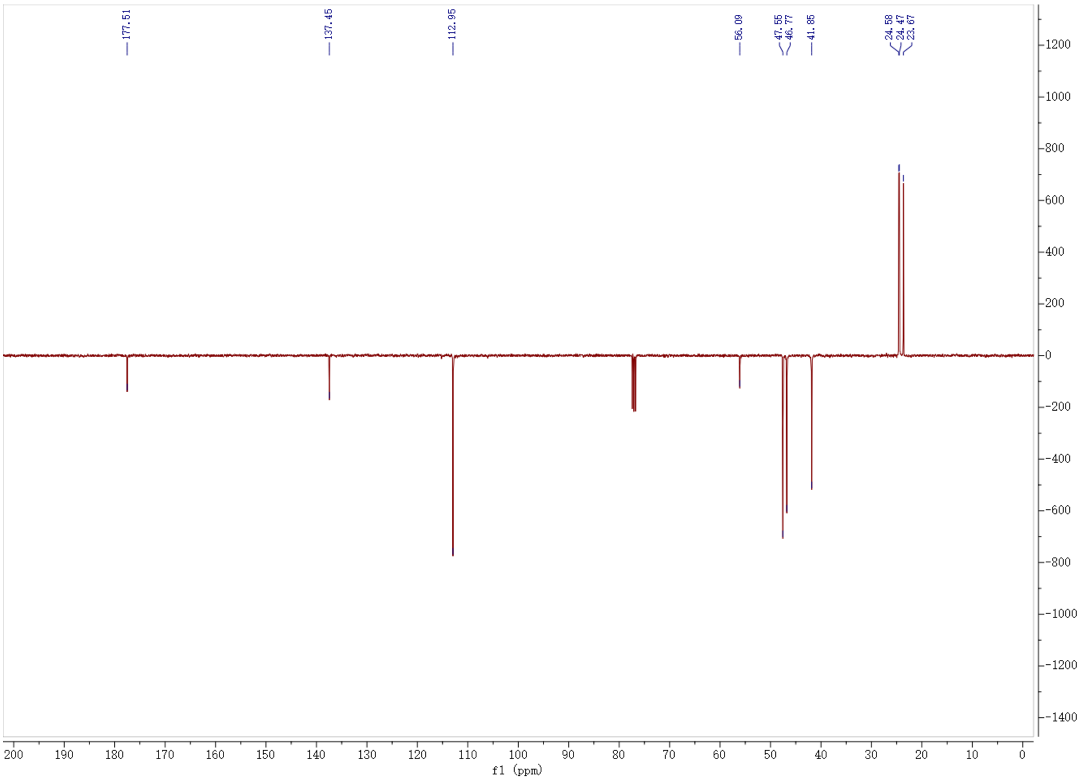
177.51
137.45
112.95
56.09
47.55
46.77
41.85
24.58
24.47
23.67
f1 (ppm)

**17**

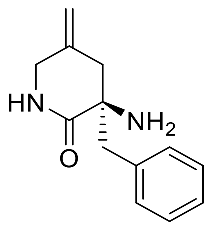
HN
NH2
O

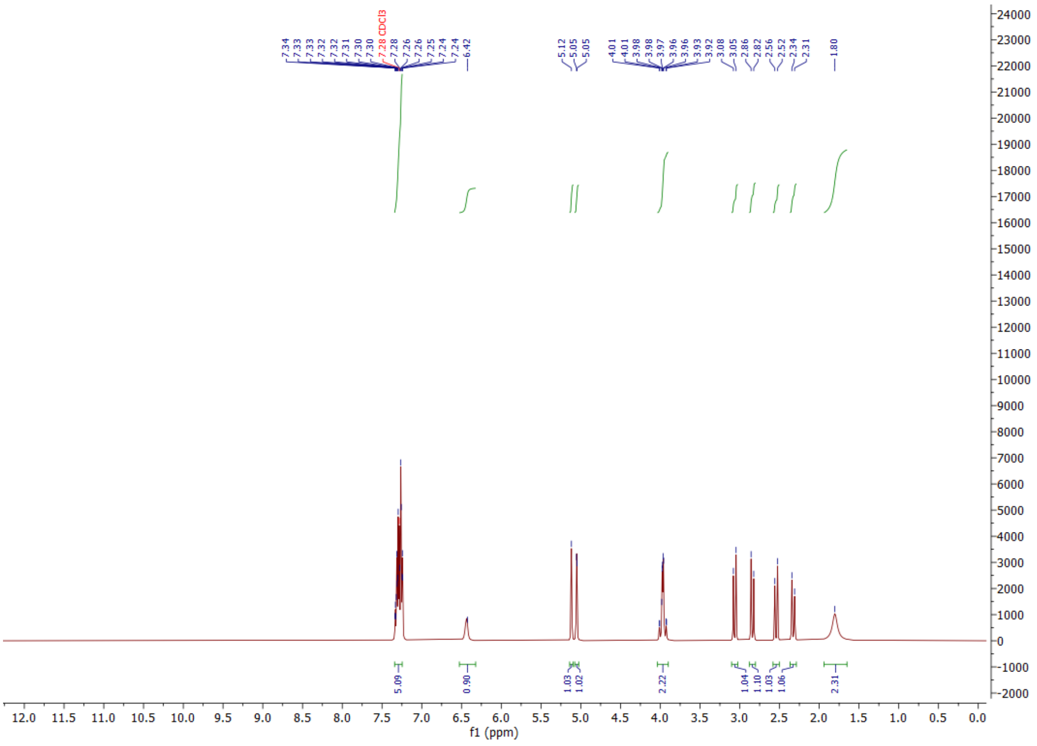
7.34
7.33
7.33
7.32
7.32
7.31
7.30
7.30
7.28 CDCl3
7.28
7.26
7.26
7.25
7.24
7.24
6.42
5.12
5.05
5.05
4.01
4.01
3.98
3.98
3.97
3.96
3.96
3.93
3.92
3.08
3.05
2.86
2.82
2.56
2.52
2.34
2.31
1.80
5.09
0.90
1.03
1.02
2.22
1.04
1.10
1.03
1.06
2.31
f1 (ppm)

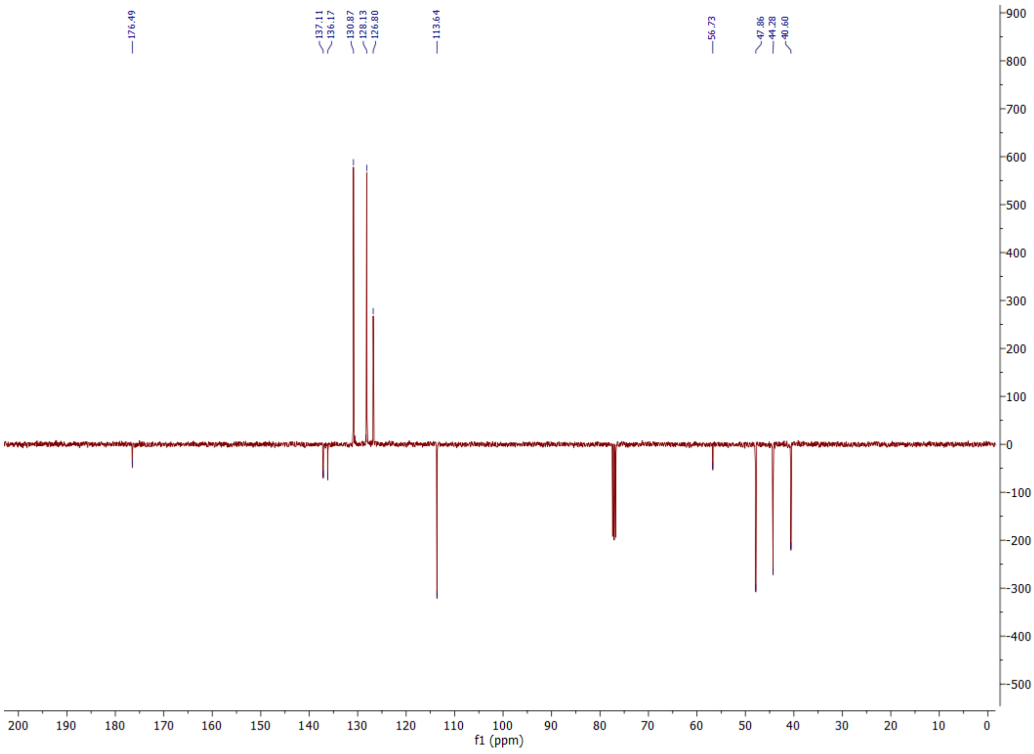
176.49
137.11
136.17
130.87
128.13
126.80
113.64
56.73
47.86
44.28
40.60
f1 (ppm)

**18**

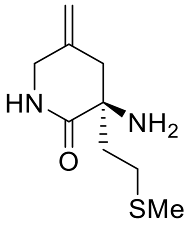
HN
NH2
O
SMe

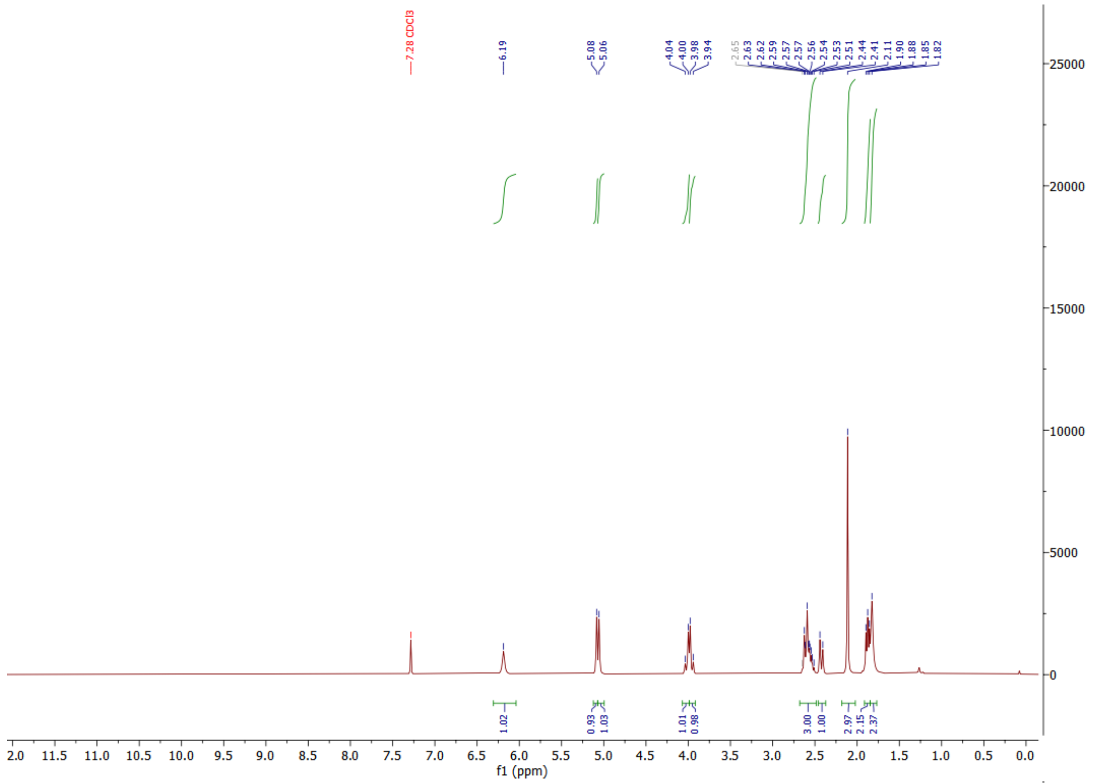
7.28 CDCl3
f1 (ppm)

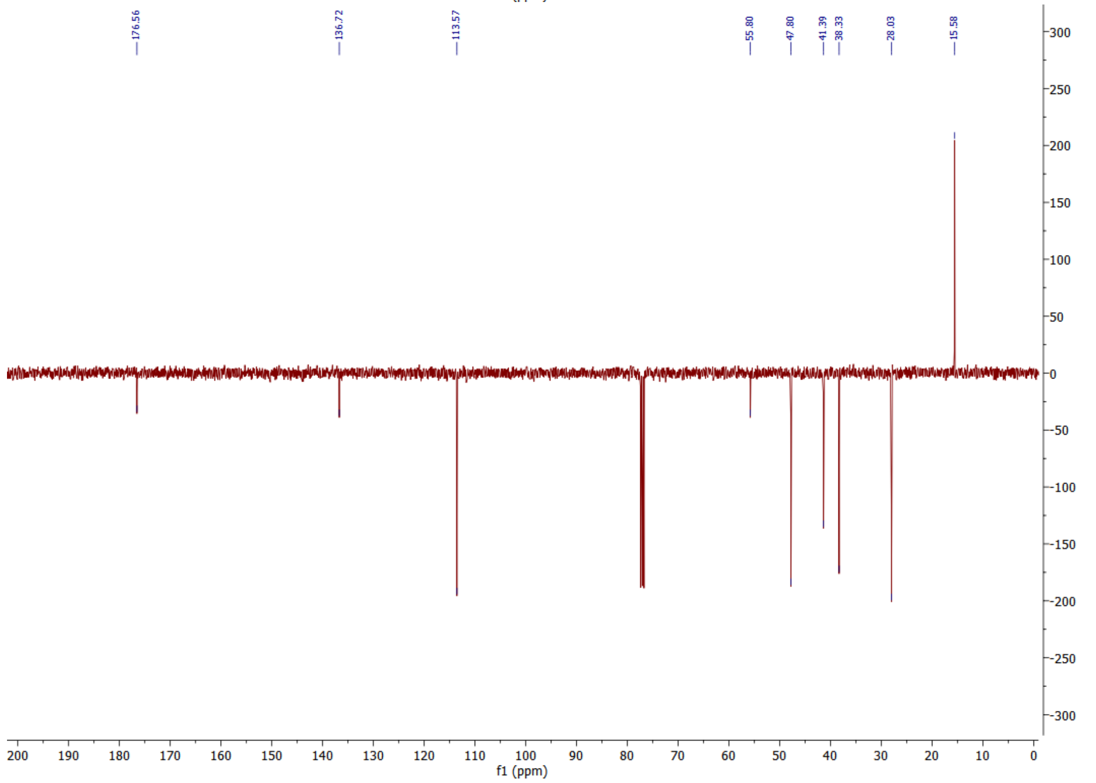
176.56
136.72
113.57
55.80
47.80
41.39
38.33
28.03
15.58
f1 (ppm)

**19**

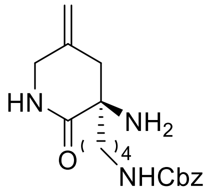


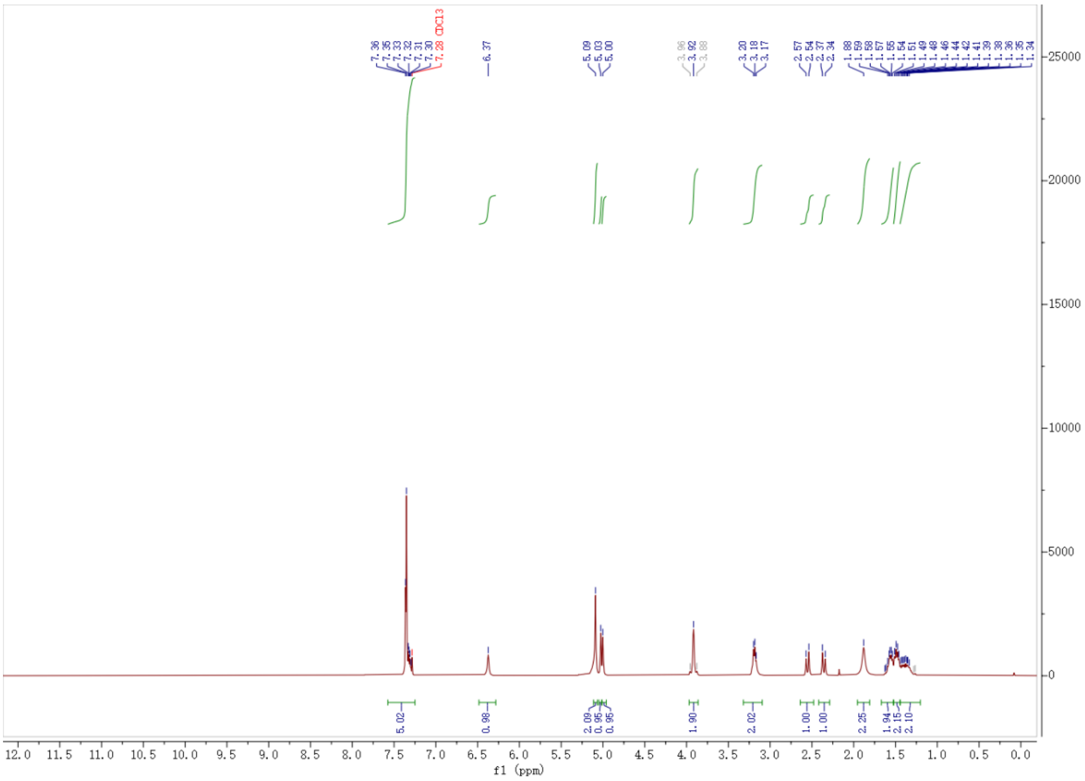

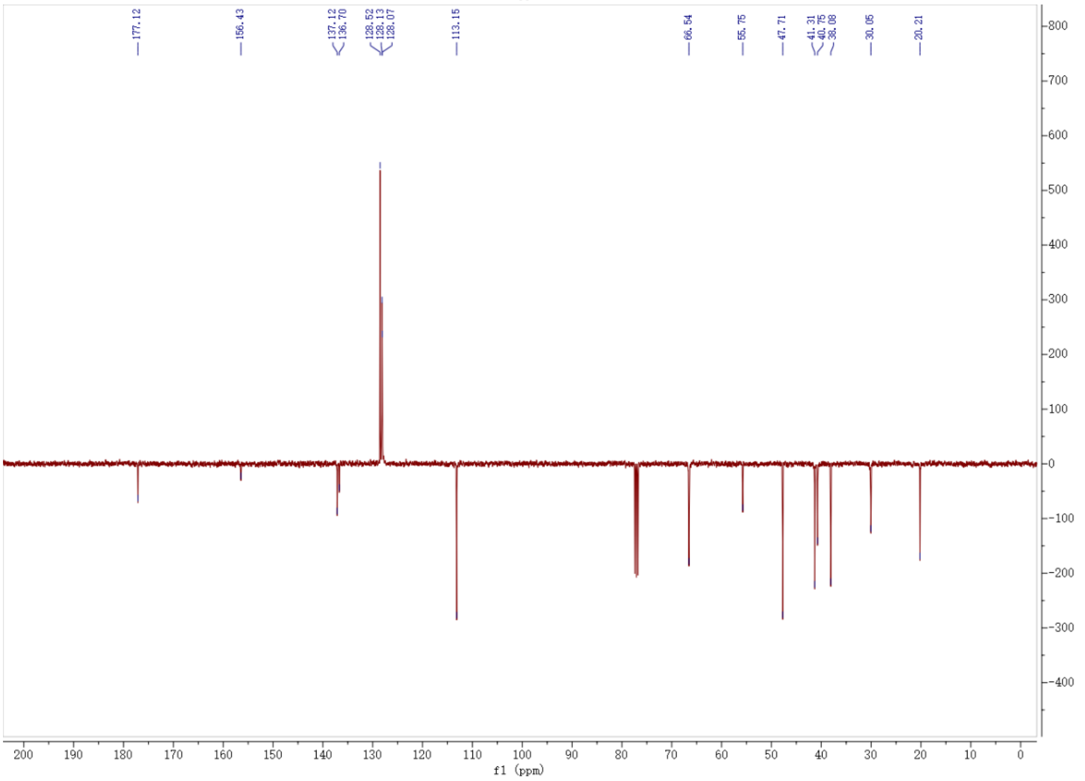

**20**

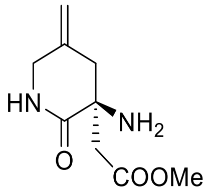
HN
$NH_2$
O
COOMe

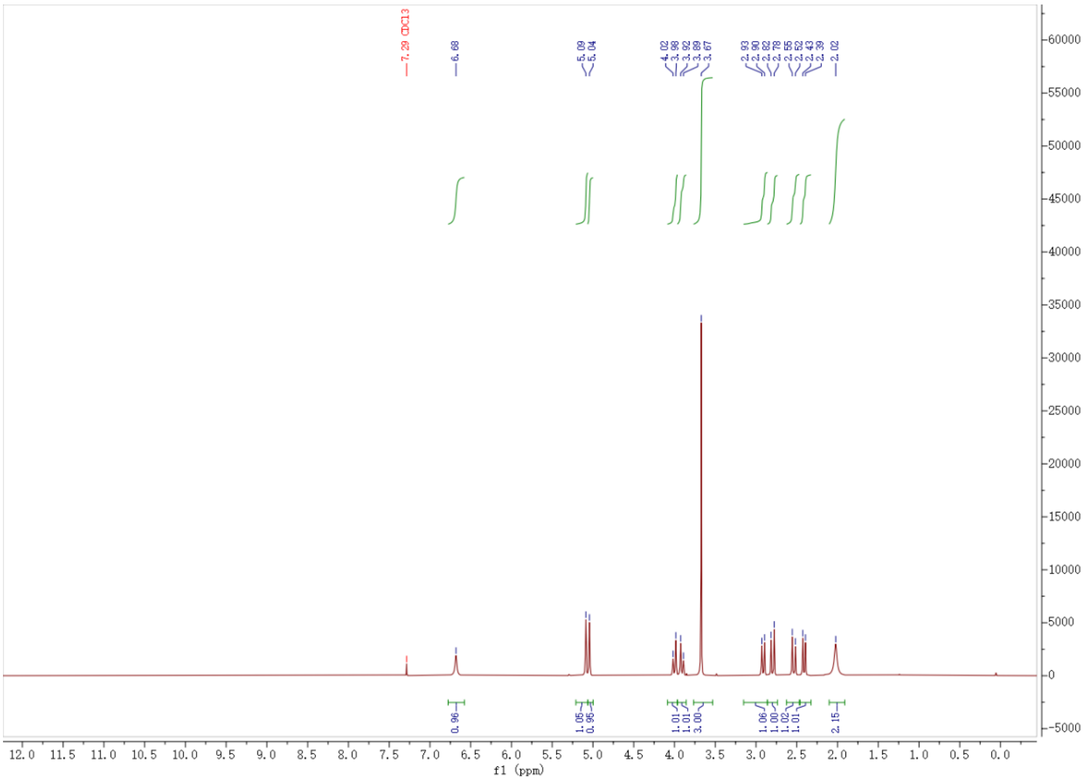
7.29 CDCl3
6.68
5.09
5.04
4.02
3.98
3.92
3.89
3.67
2.93
2.90
2.82
2.78
2.55
2.52
2.43
2.39
2.02
0.96
1.05
0.95
1.01
1.01
3.00
1.06
1.00
1.02
1.01
2.15
f1 (ppm)

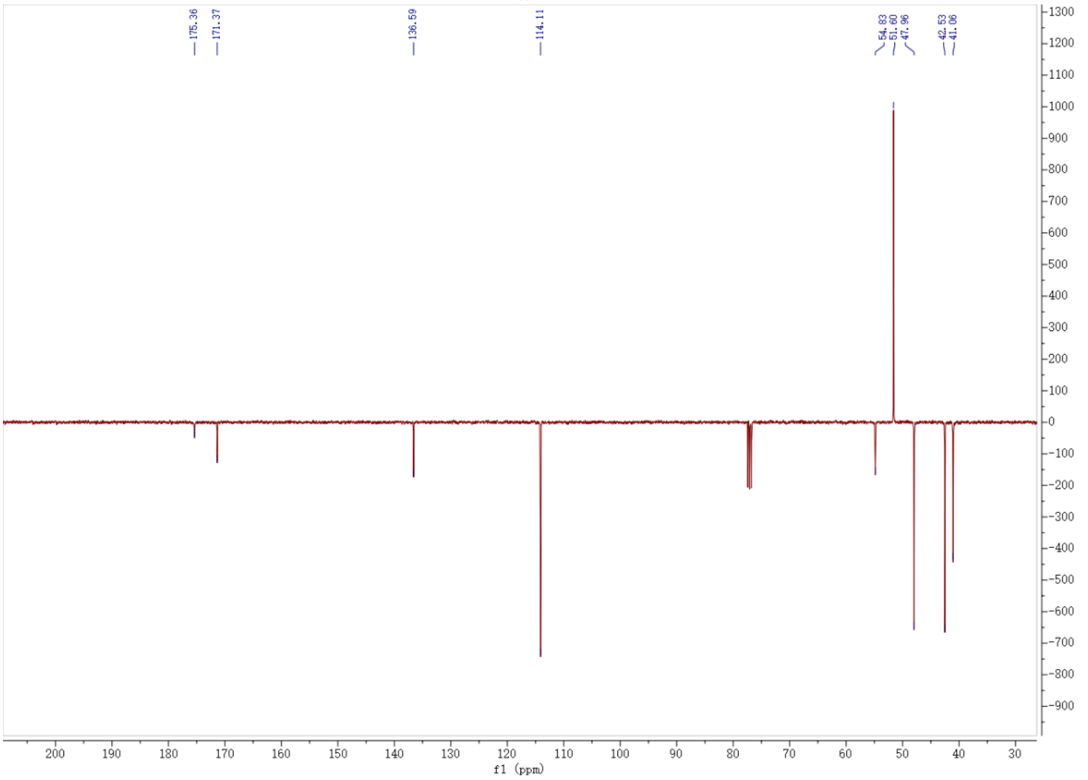
175.36
171.37
136.59
114.11
54.83
51.60
47.96
42.53
41.06
f1 (ppm)

**21**

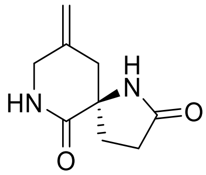

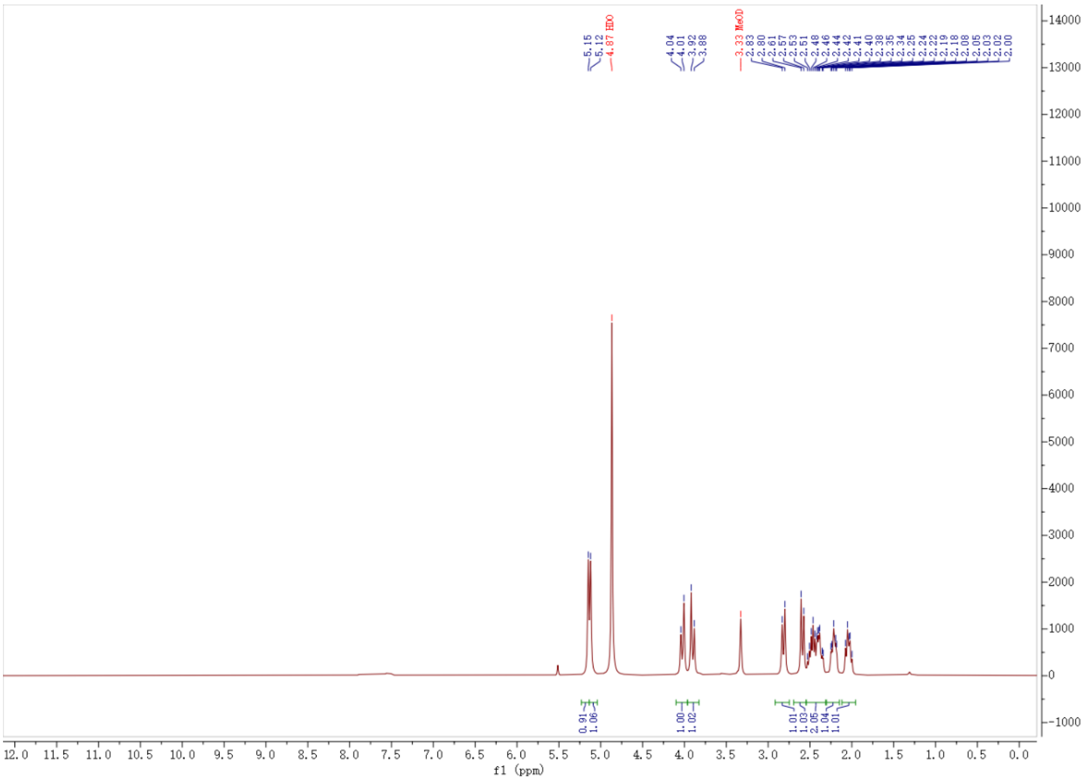

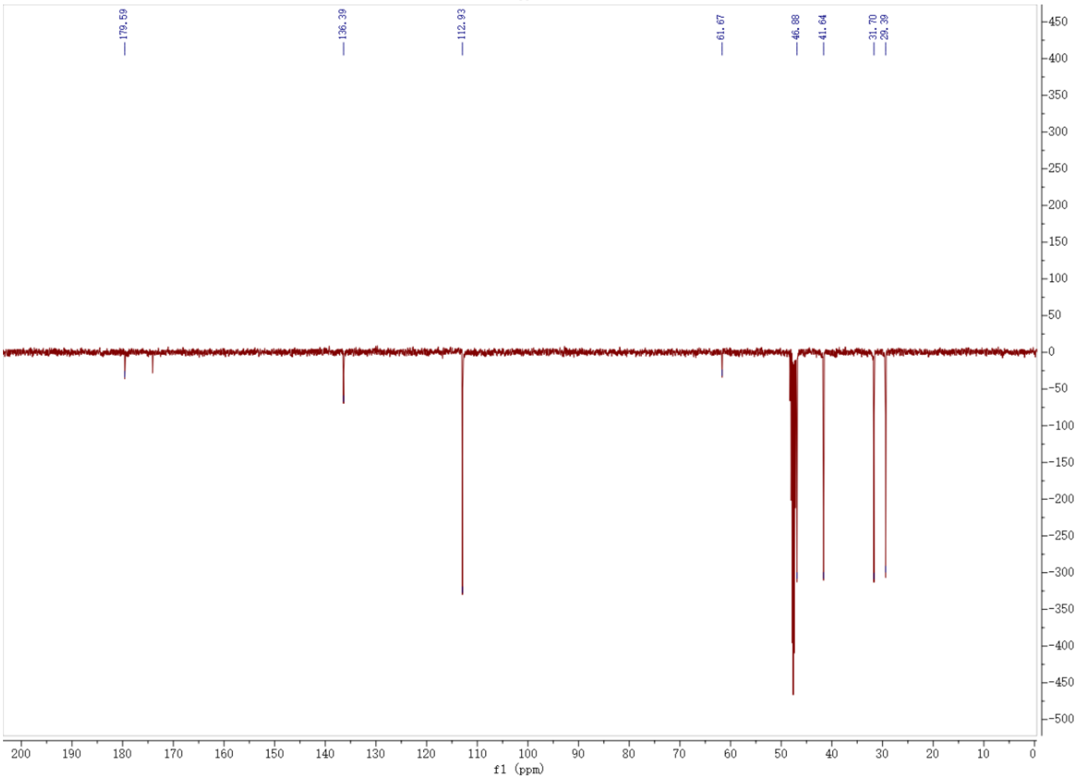

**4a**

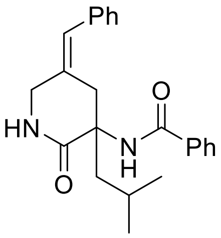


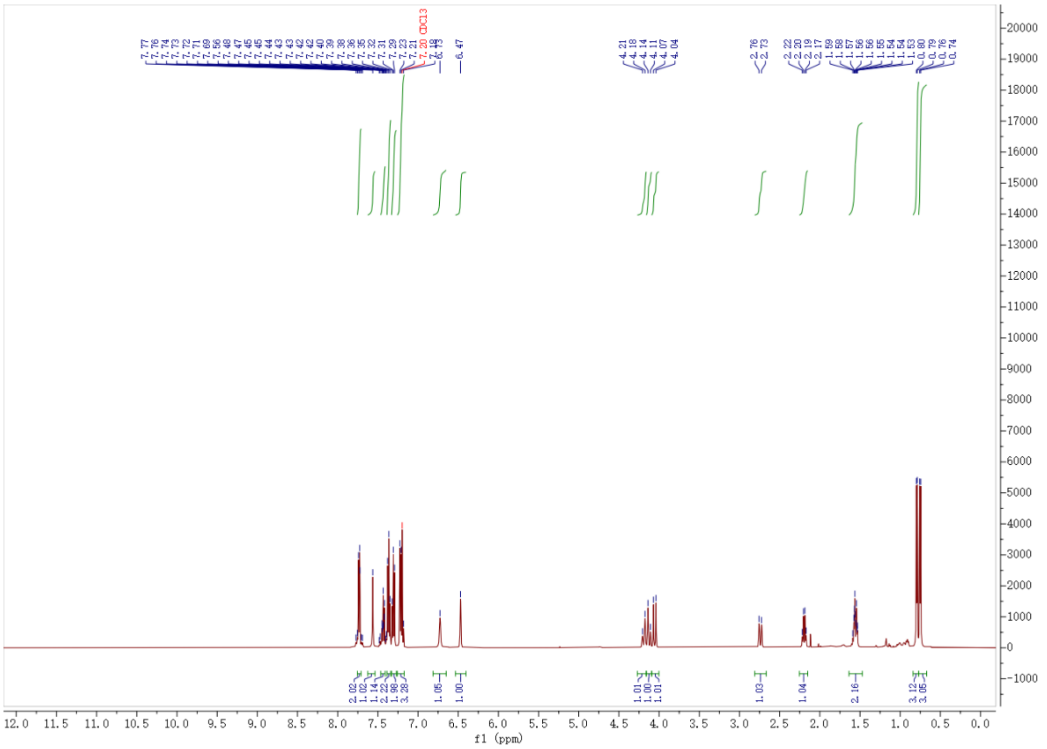

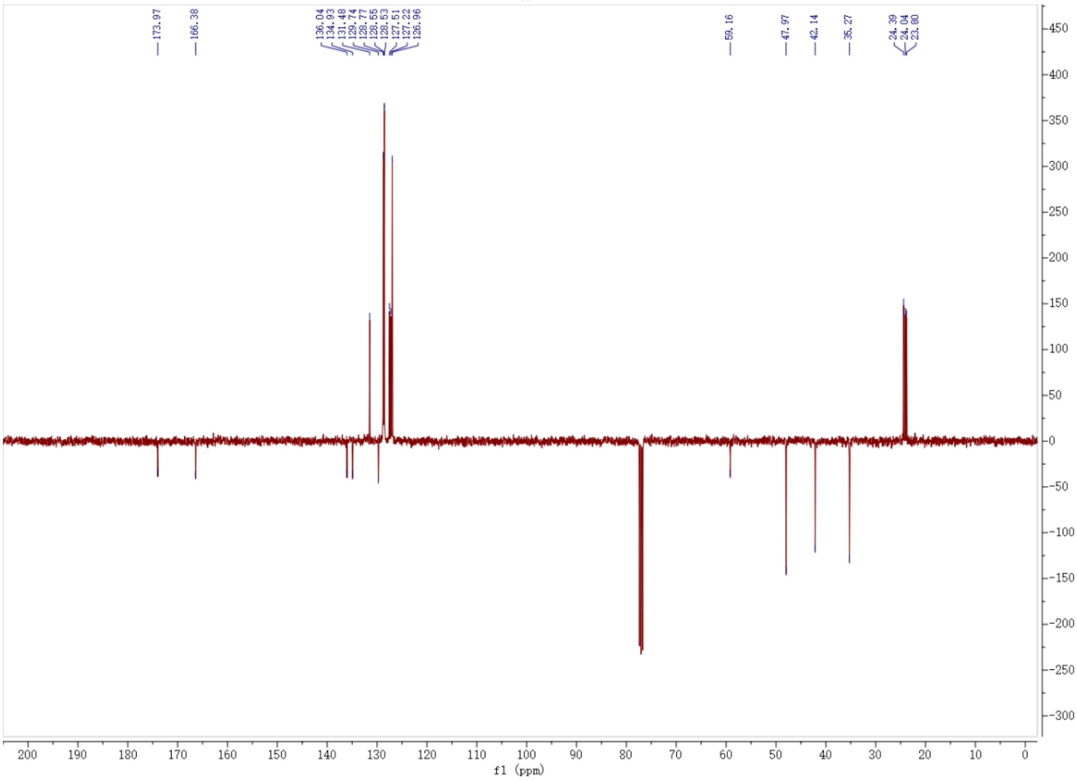

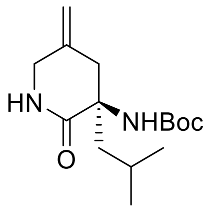
HN
NHBoc
O

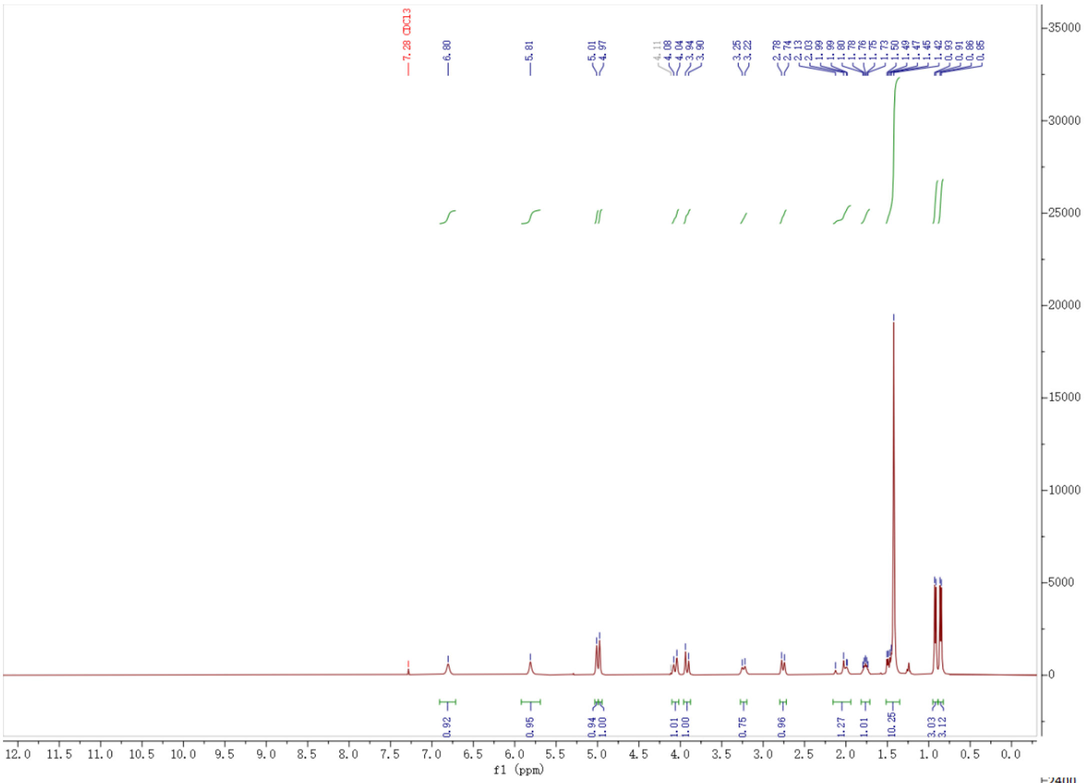
7.28 CDCl3
6.80
5.81
5.01
4.97
4.11
4.08
4.04
3.94
3.90
3.25
3.22
2.78
2.74
2.13
2.03
1.99
1.80
1.78
1.76
1.75
1.73
1.50
1.49
1.47
1.45
1.42
0.93
0.91
0.86
0.85
0.92
0.95
0.94
1.00
1.01
1.00
0.75
0.96
1.27
1.01
10.25
3.03
3.12
f1 (ppm)

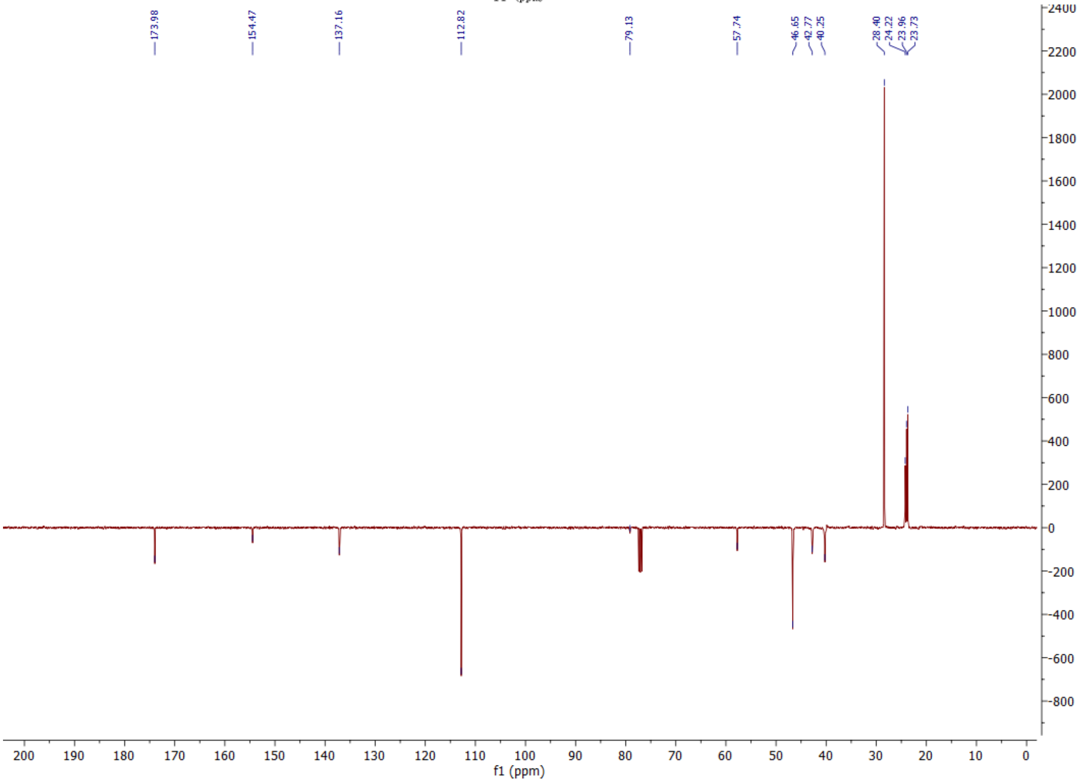
173.98
154.47
137.16
112.82
79.13
57.74
46.65
42.77
40.25
28.40
24.22
23.96
23.73
f1 (ppm)

**22**

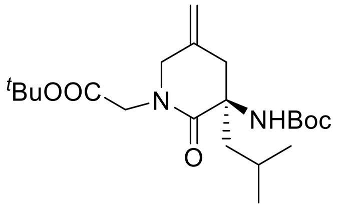

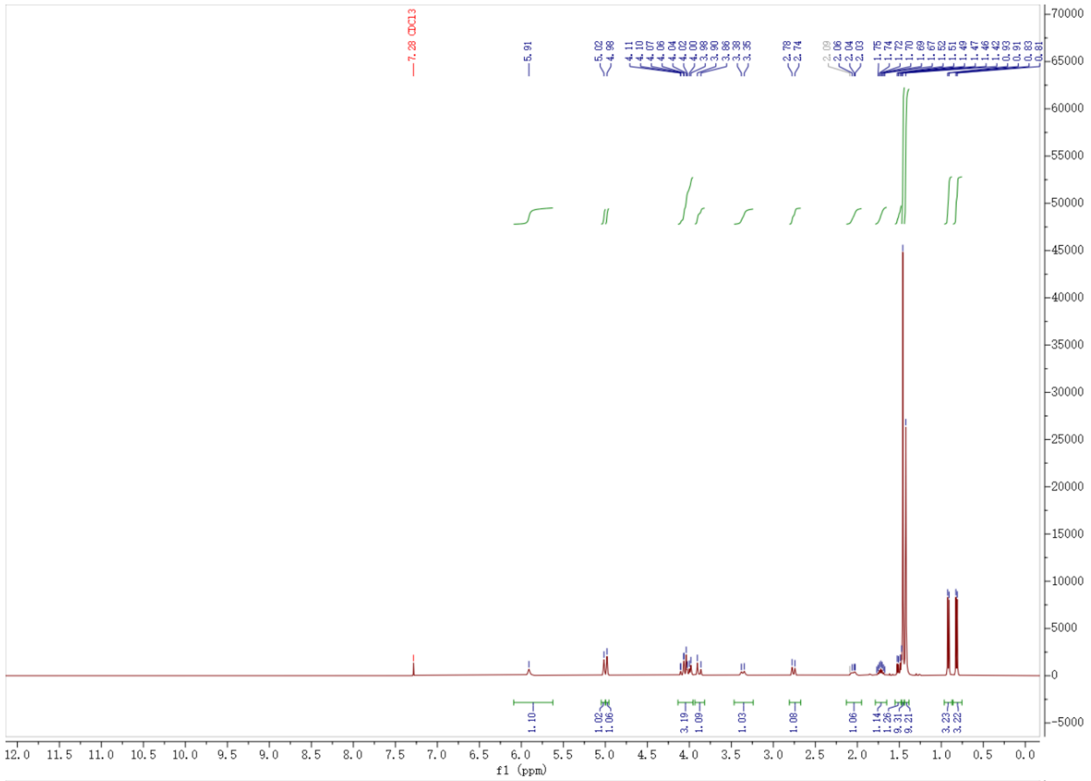

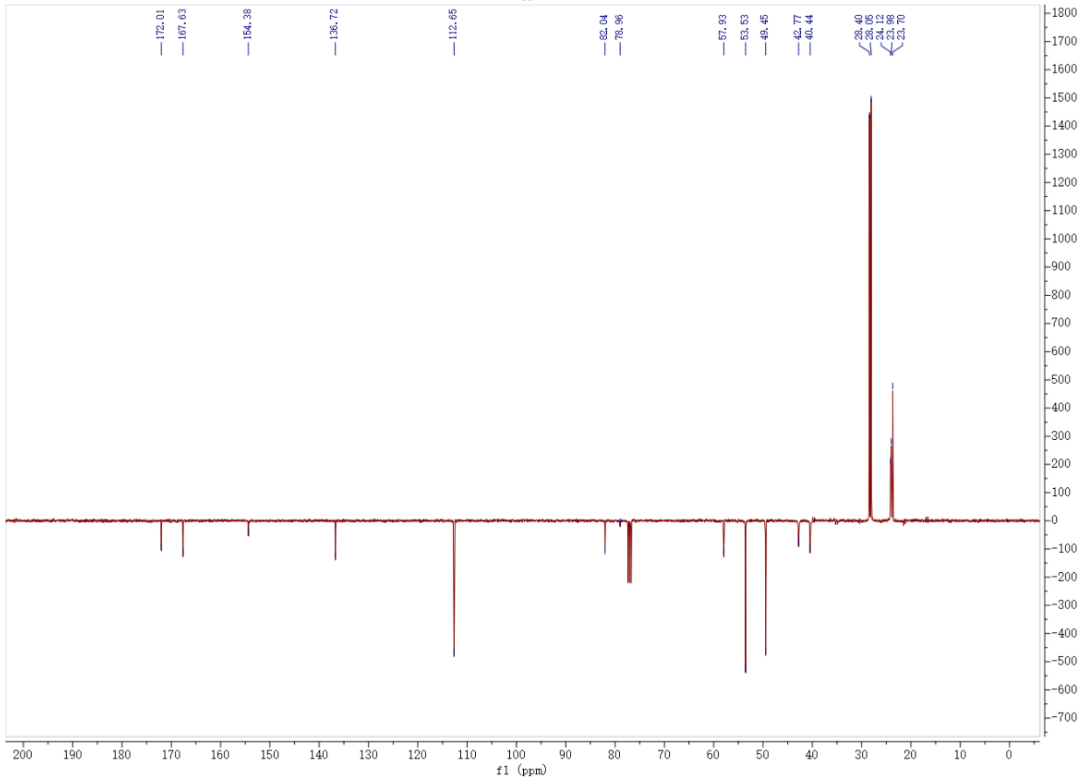

23

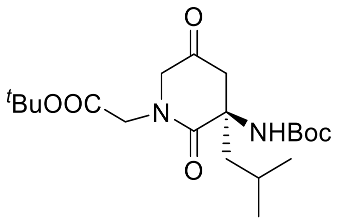

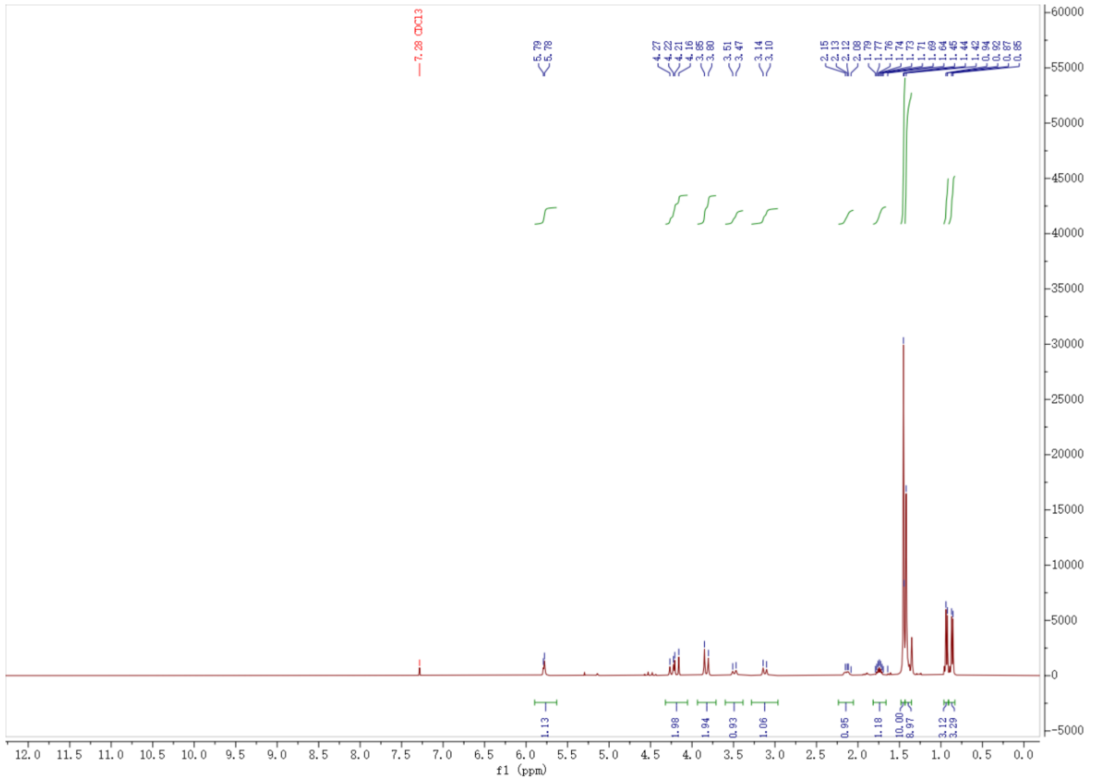

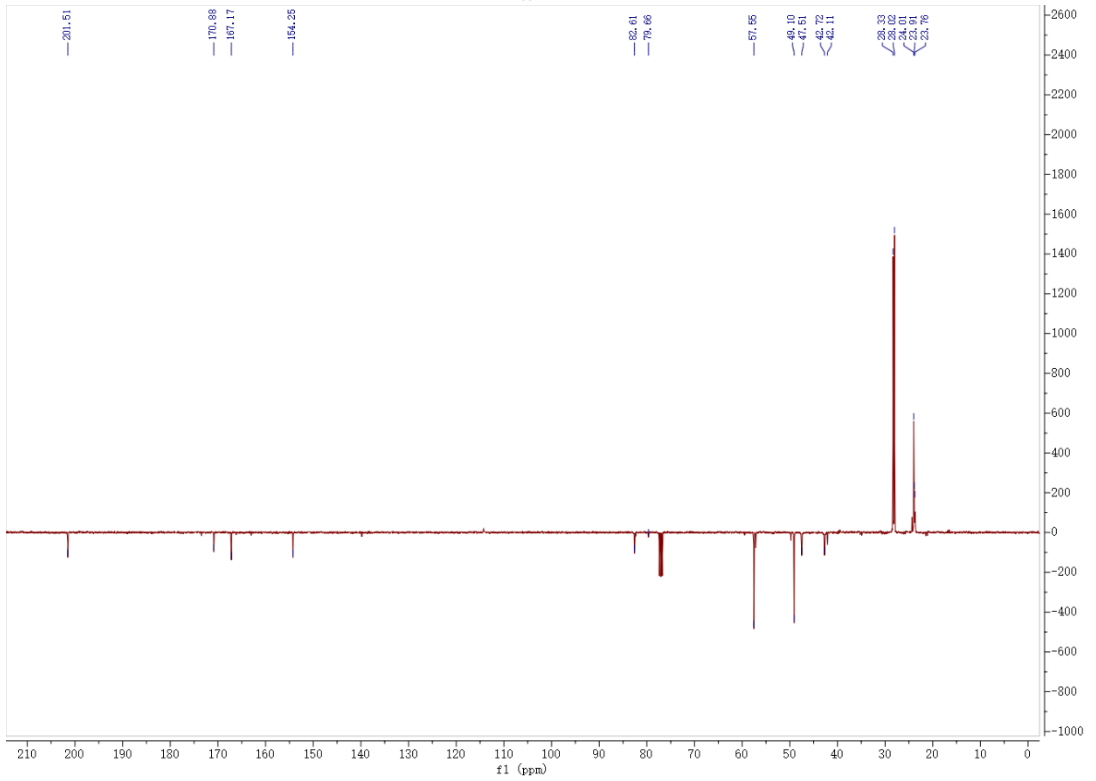

**24**

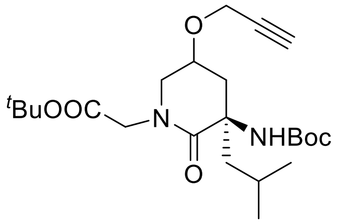


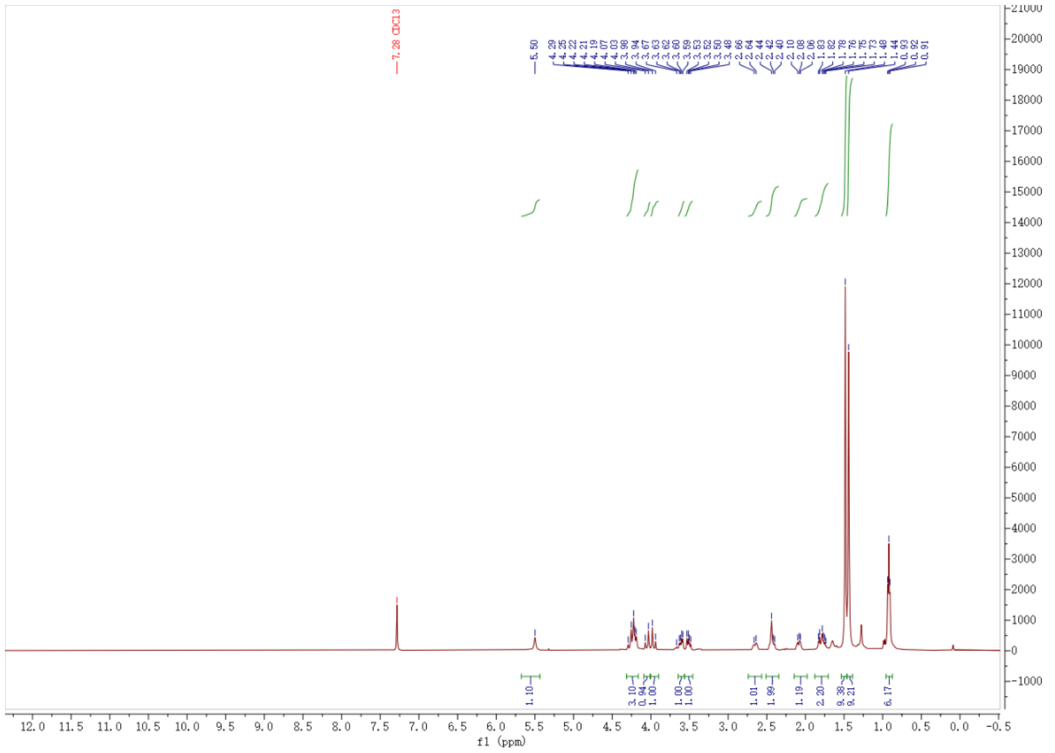


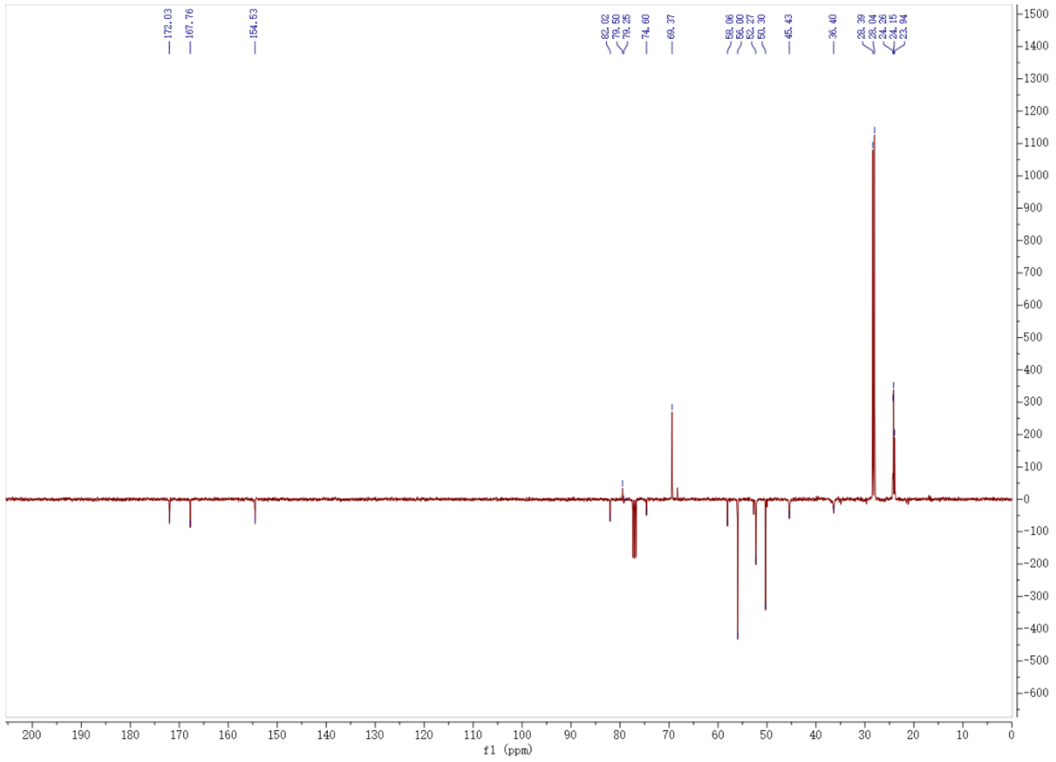

**25**

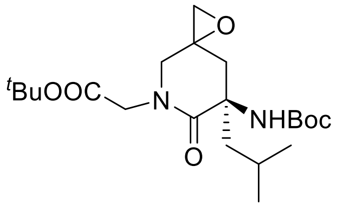

O
$^t$BuOOC
N
NHBoc
O


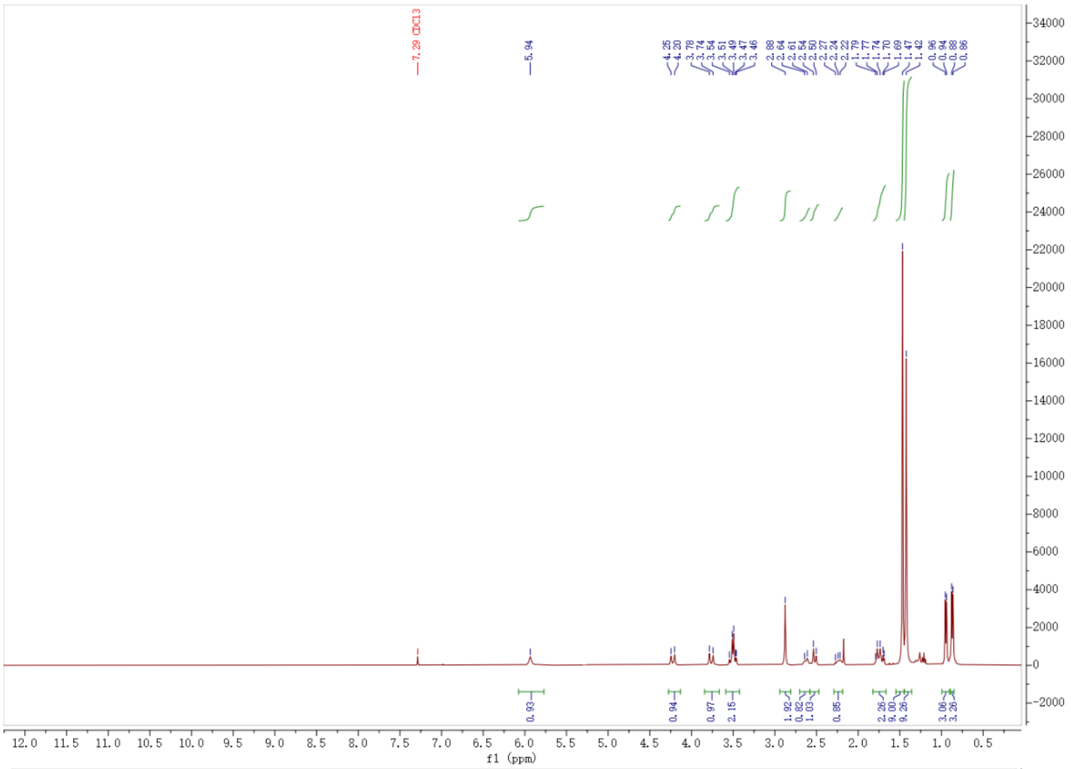

7.29 CDCl3
5.94
4.25
4.20
3.78
3.74
3.54
3.51
3.49
3.47
3.46
2.88
2.64
2.61
2.54
2.50
2.27
2.24
2.22
1.79
1.77
1.74
1.70
1.69
1.47
1.42
0.96
0.94
0.88
0.86
0.93
0.94
0.97
2.15
1.92
0.82
1.03
0.85
2.26
9.00
9.26
3.06
3.26
f1 (ppm)


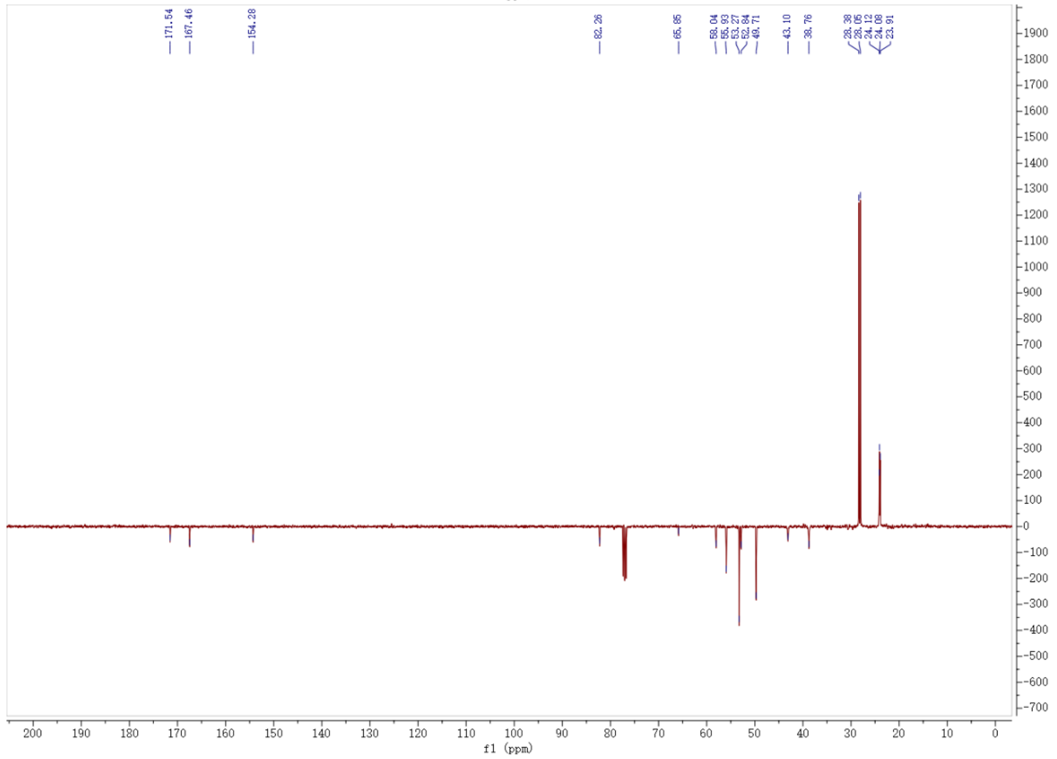

171.54
167.46
154.28
82.26
65.85
58.04
55.93
53.27
52.84
49.71
43.10
38.76
28.38
28.05
24.12
24.08
23.91
f1 (ppm)

**26**

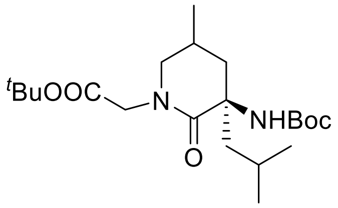


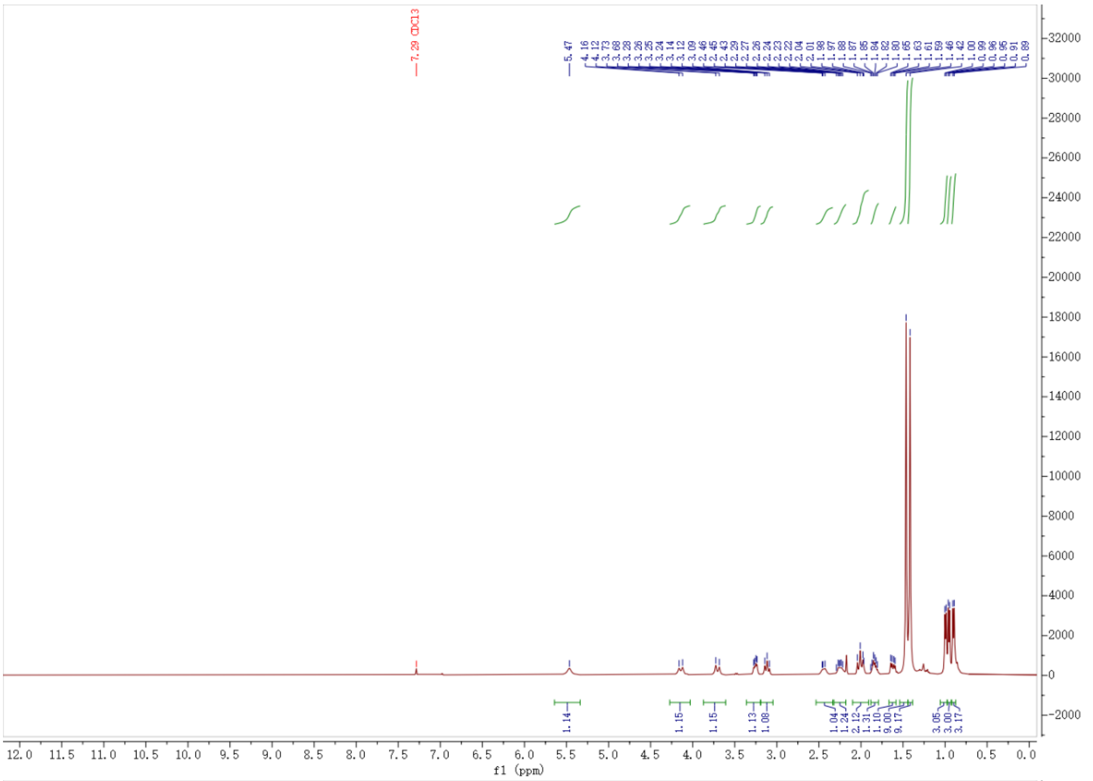

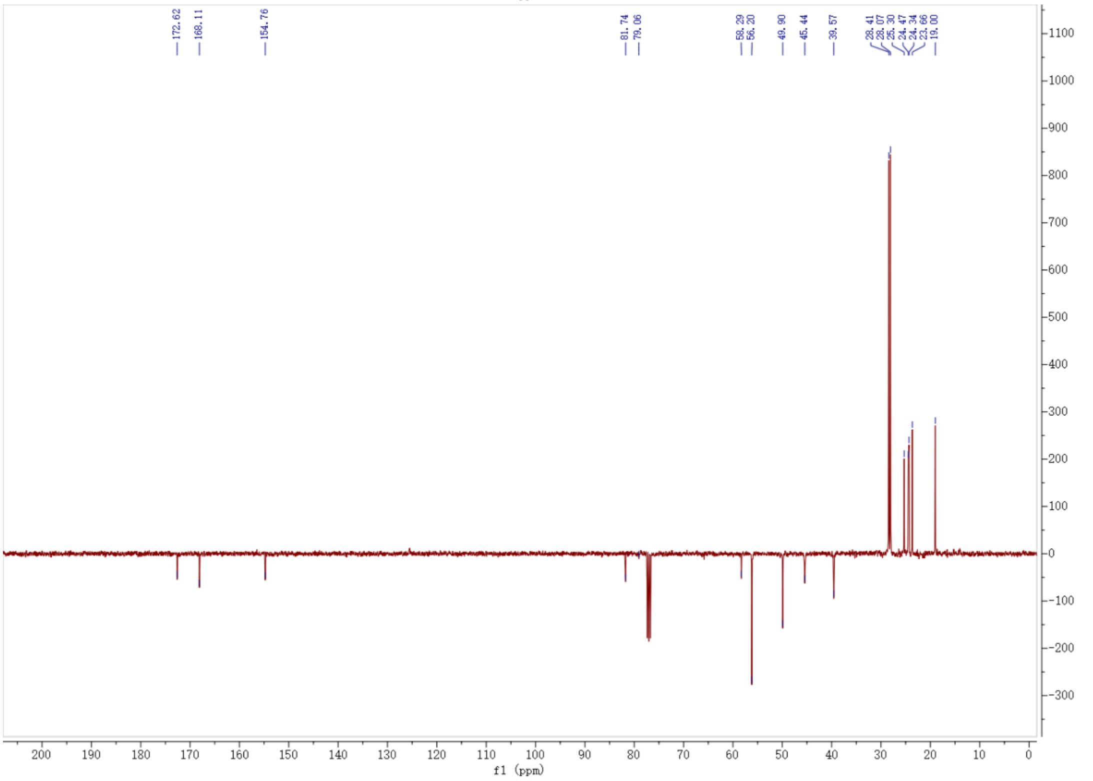

**27**

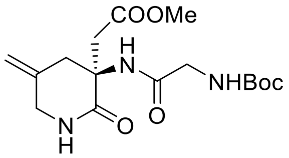


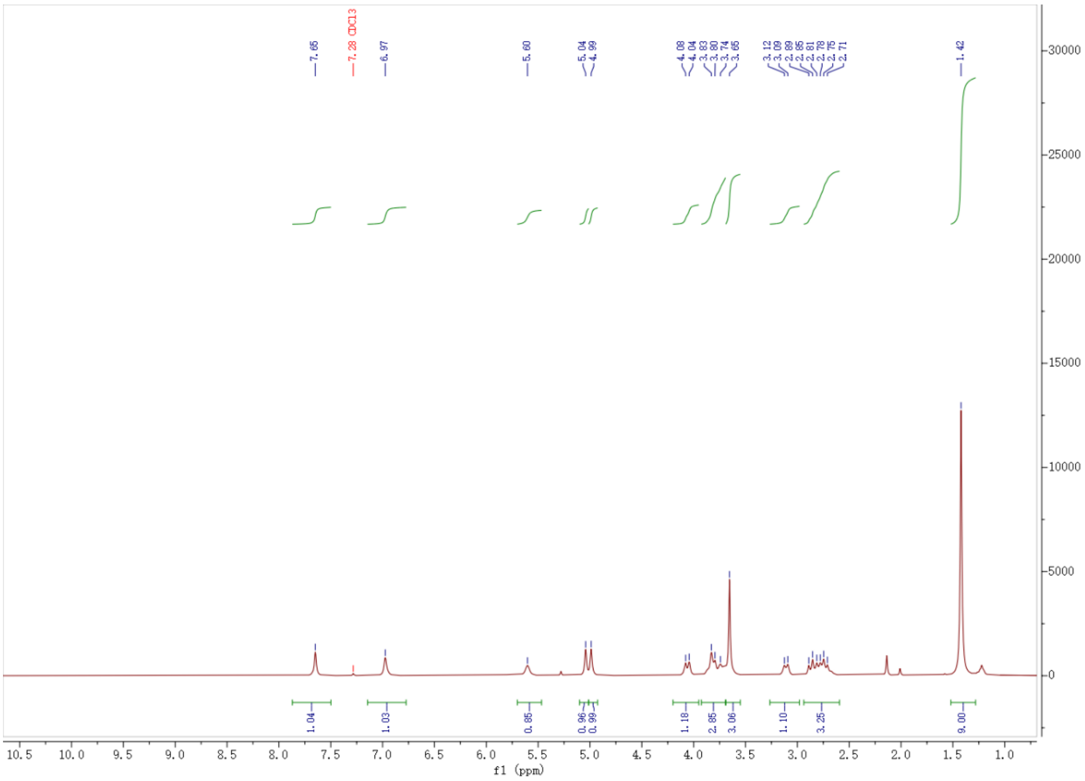

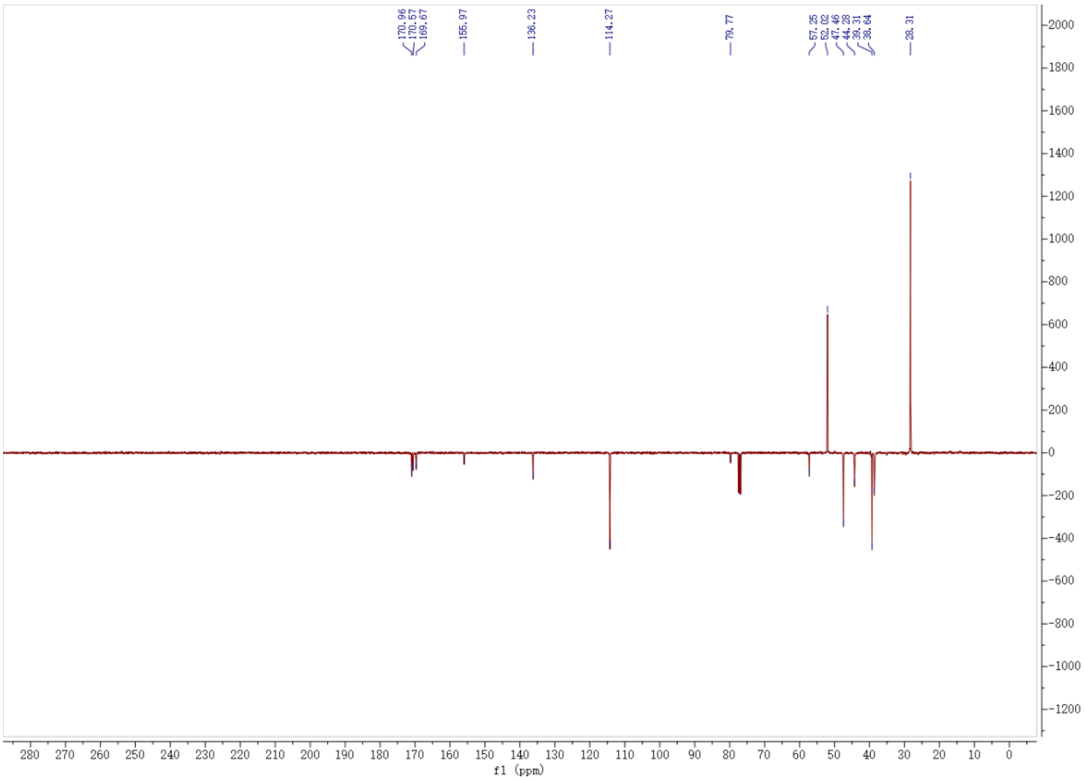

**28**

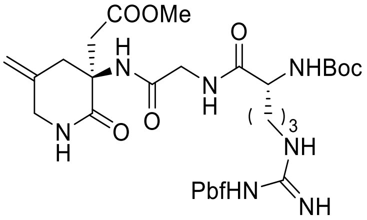


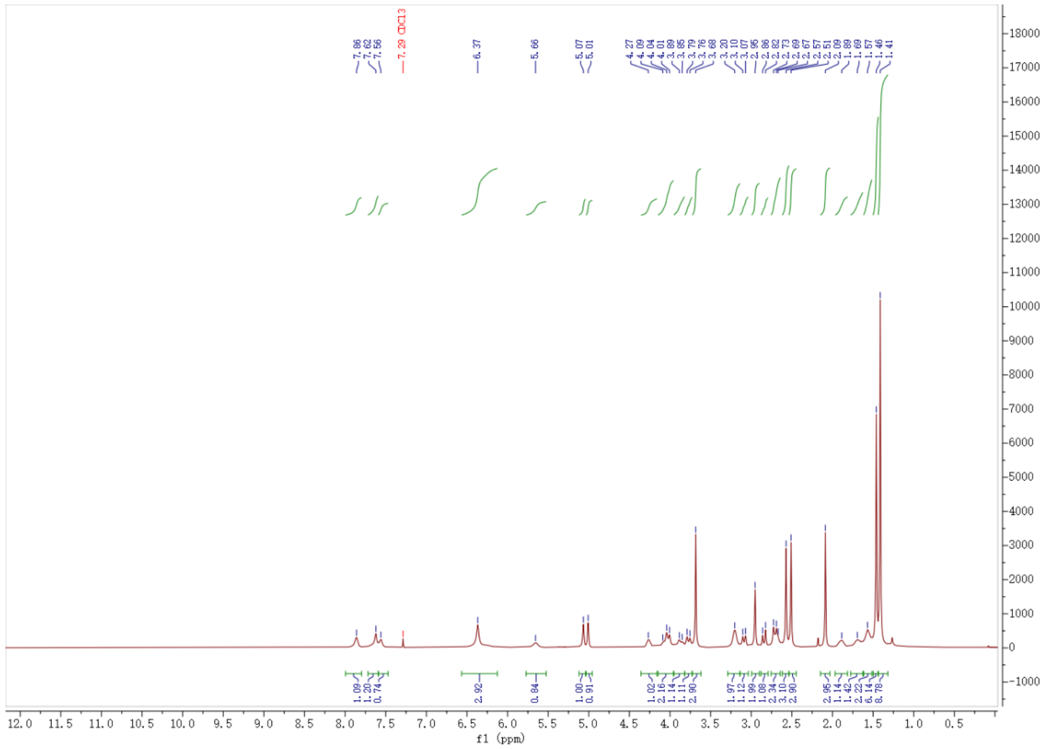

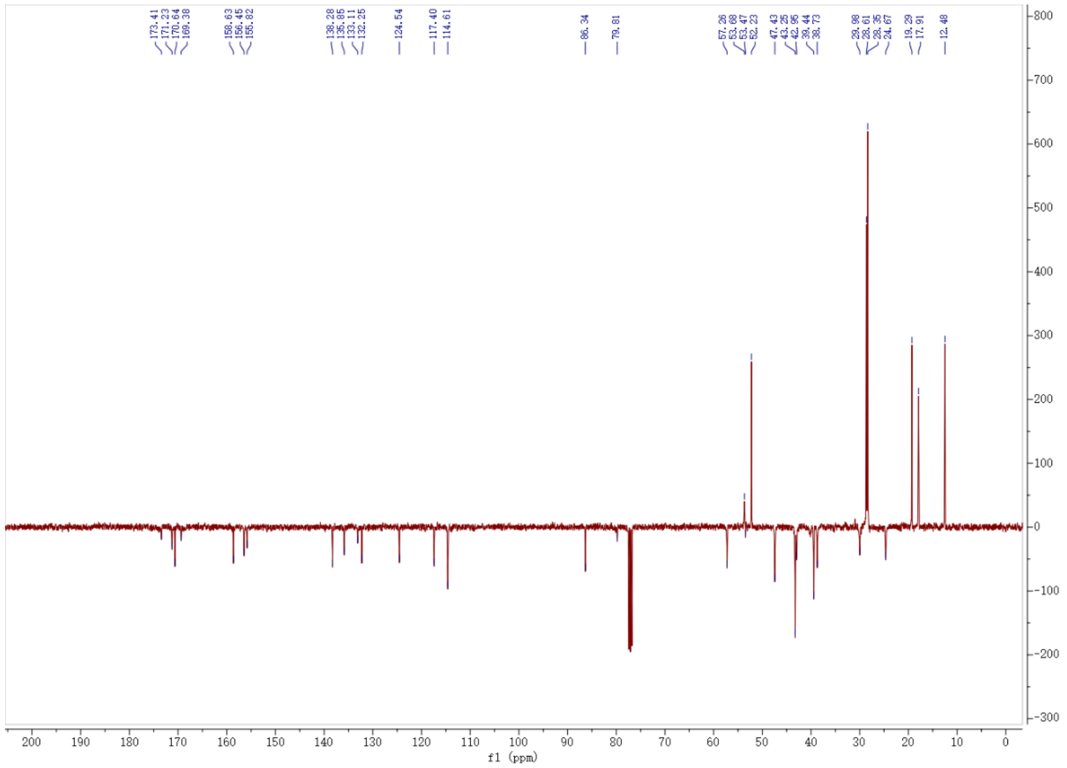

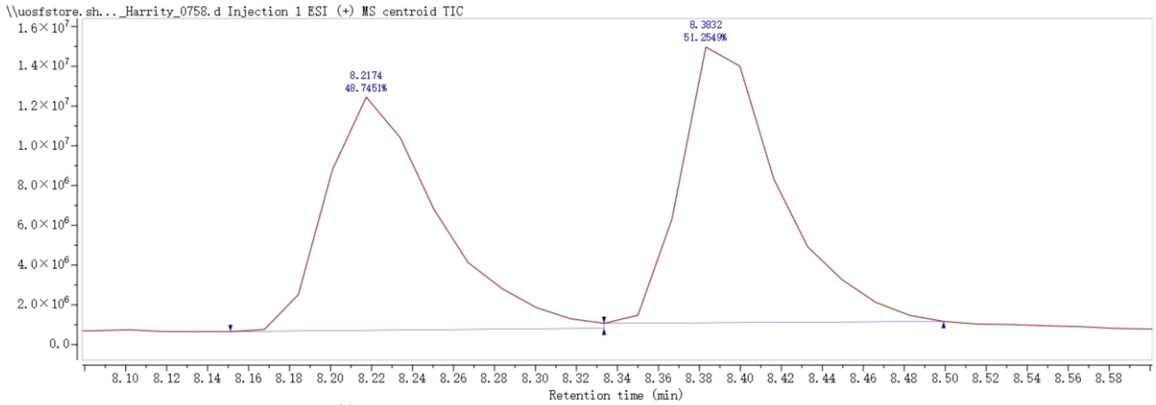
\\uosfstore.sh..._Harrity_0758.d Injection 1 ESI (+) MS centroid TIC
8.2174
48.7451%
8.3832
51.2549%
Retention time (min)

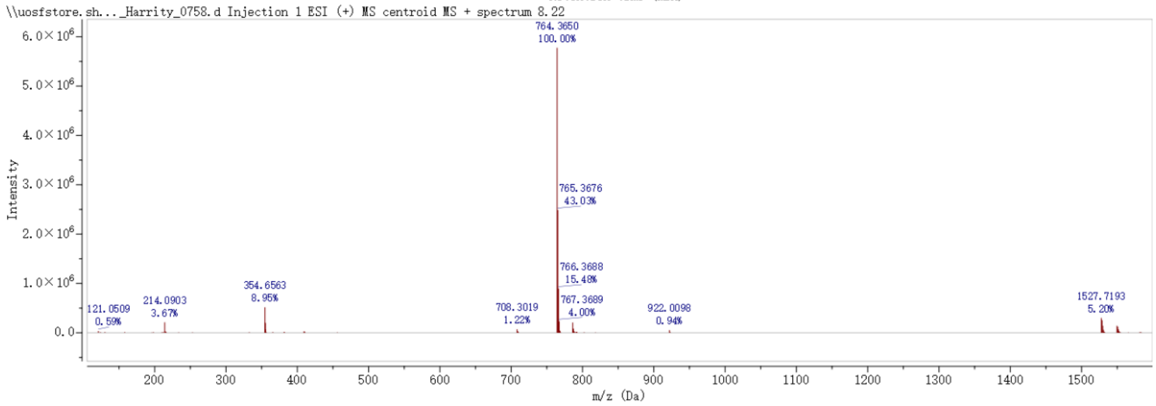
\\uosfstore.sh..._Harrity_0758.d Injection 1 ESI (+) MS centroid MS + spectrum 8.22
Intensity
764.3650
100.00%
765.3676
43.03%
766.3688
15.48%
767.3689
4.00%
121.0509
0.59%
214.0903
3.67%
354.6563
8.95%
708.3019
1.22%
922.0098
0.94%
1527.7193
5.20%
m/z (Da)

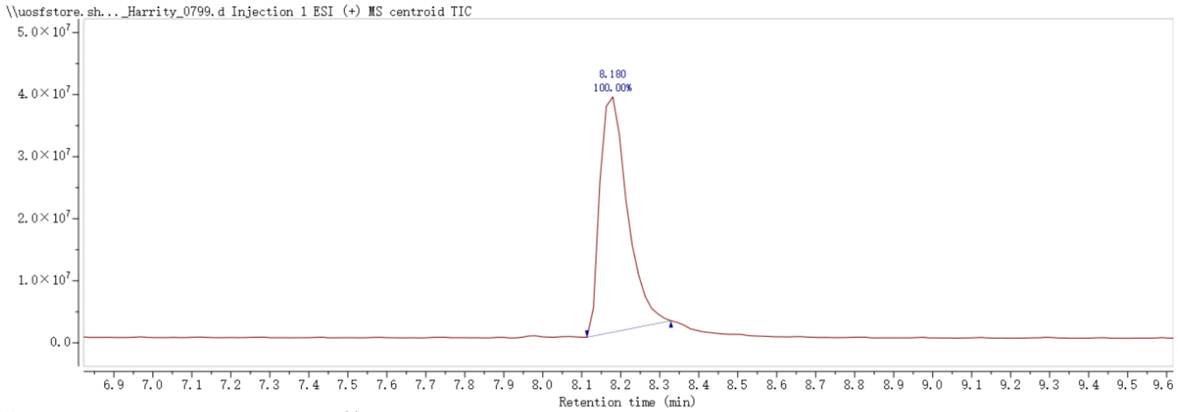
\\uosfstore.sh..._Harrity_0799.d Injection 1 ESI (+) MS centroid TIC
8.180
100.00%
Retention time (min)

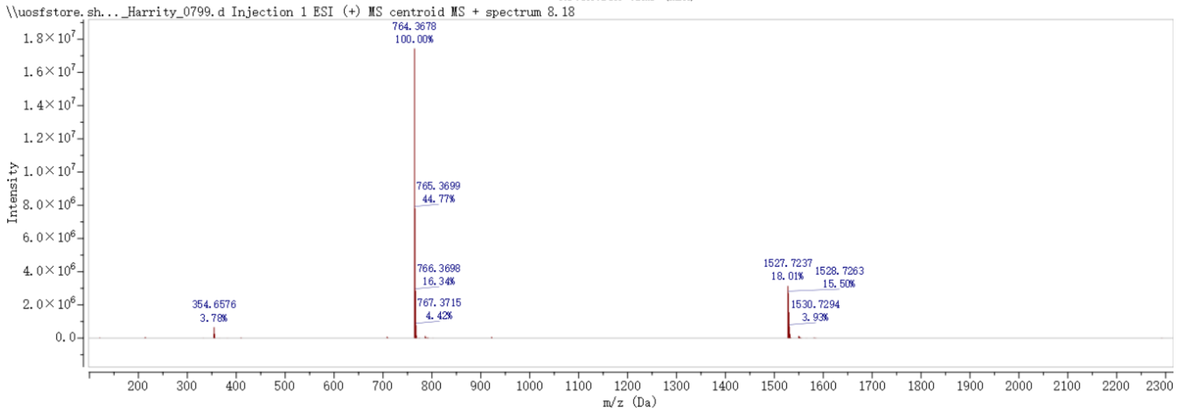
\\uosfstore.sh..._Harrity_0799.d Injection 1 ESI (+) MS centroid MS + spectrum 8.18
Intensity
764.3678
100.00%
765.3699
44.77%
766.3698
16.34%
767.3715
4.42%
354.6576
3.78%
1527.7237
18.01%
1528.7263
15.50%
1530.7294
3.93%
m/z (Da)

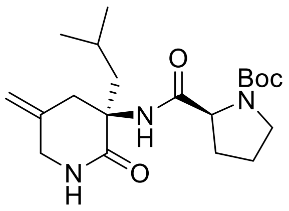

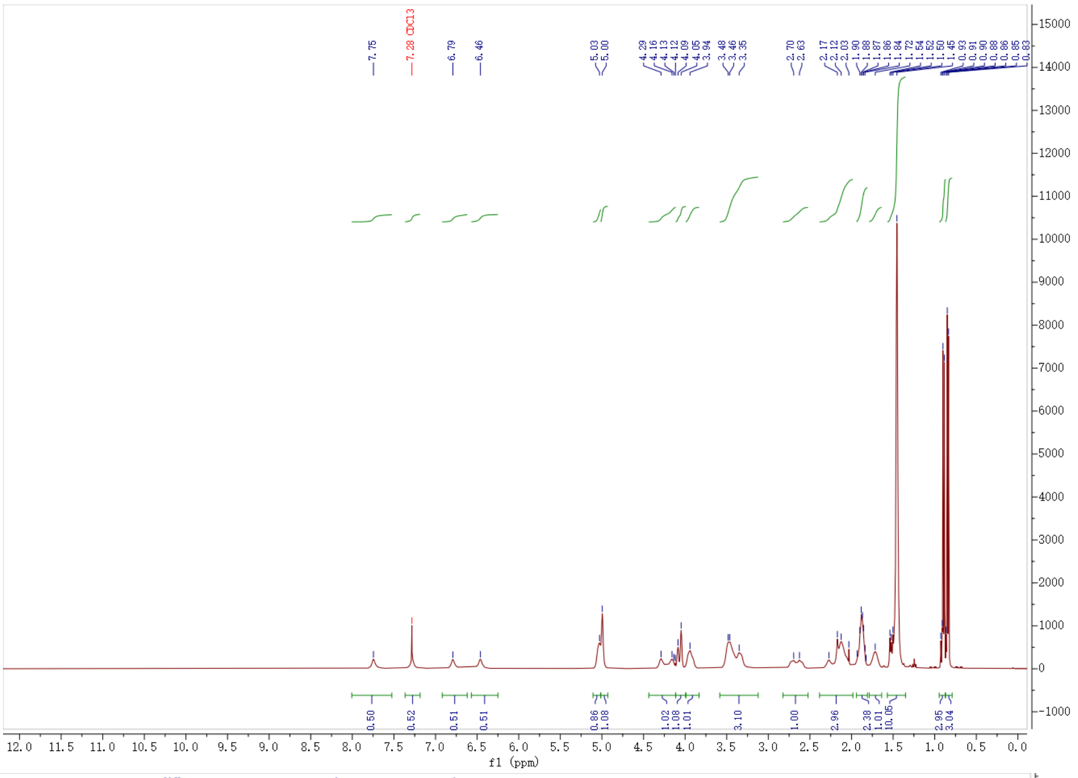

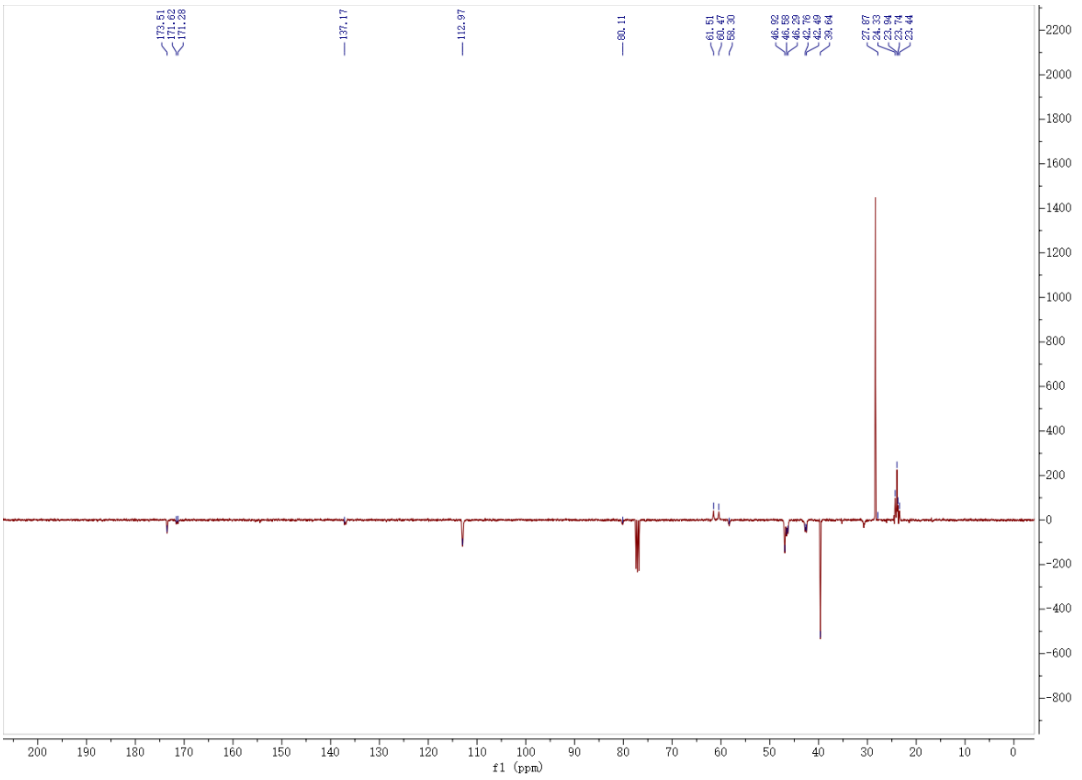

**29**

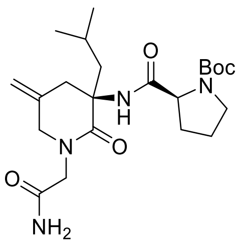


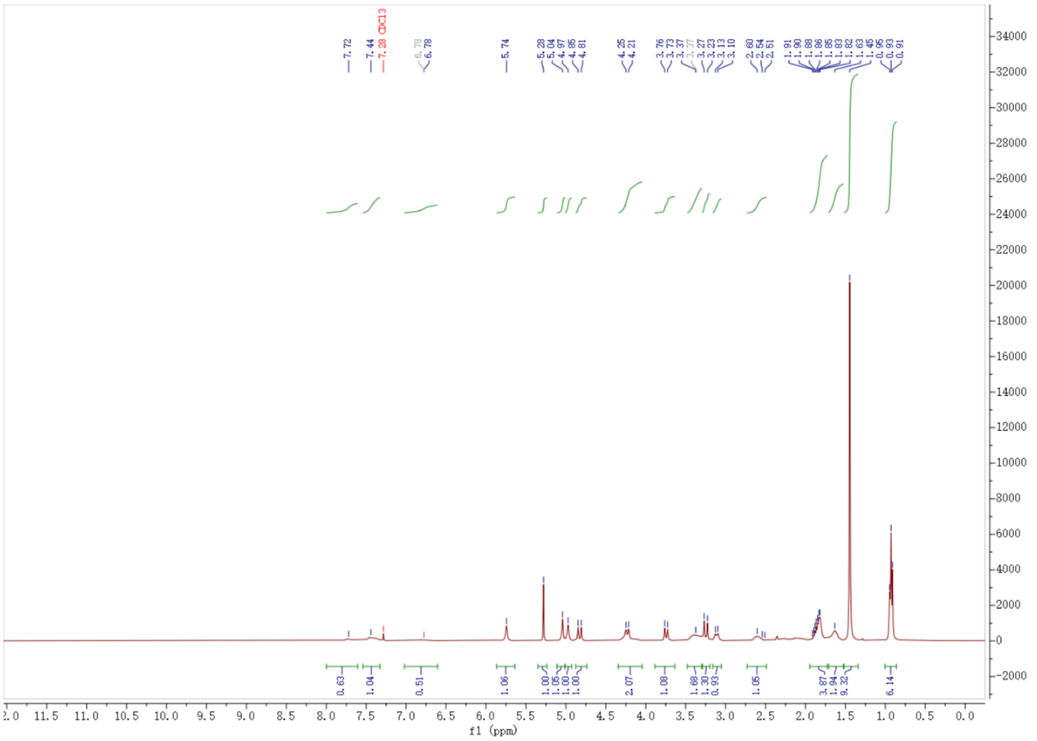

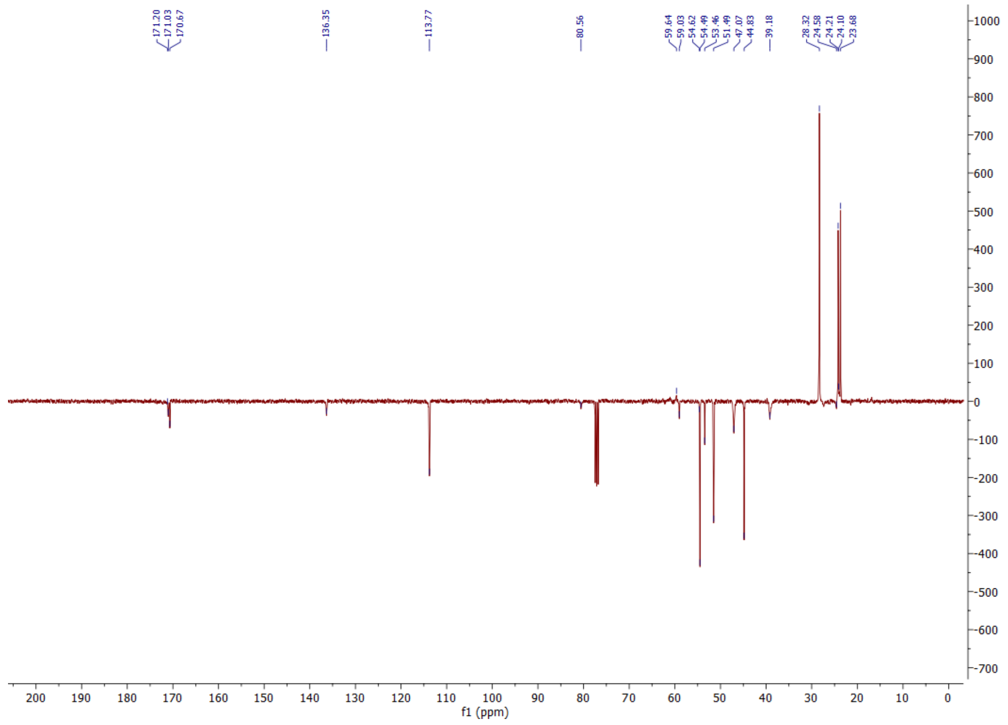

**30**

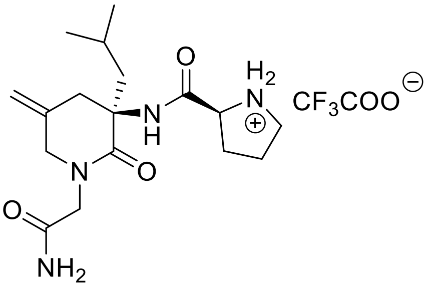

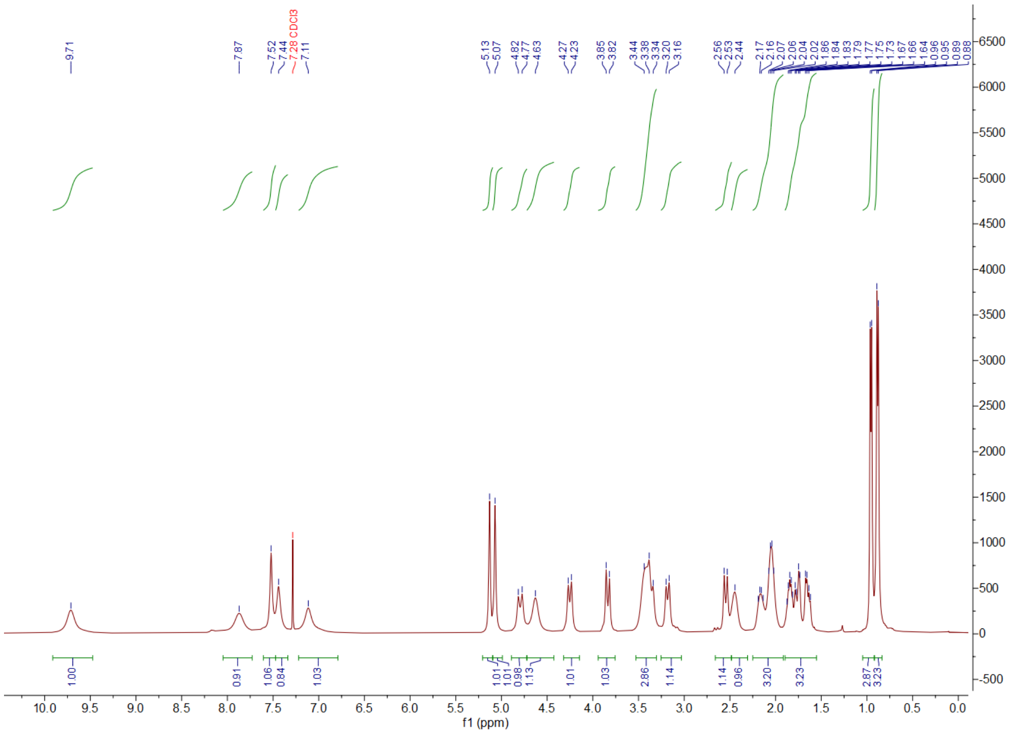


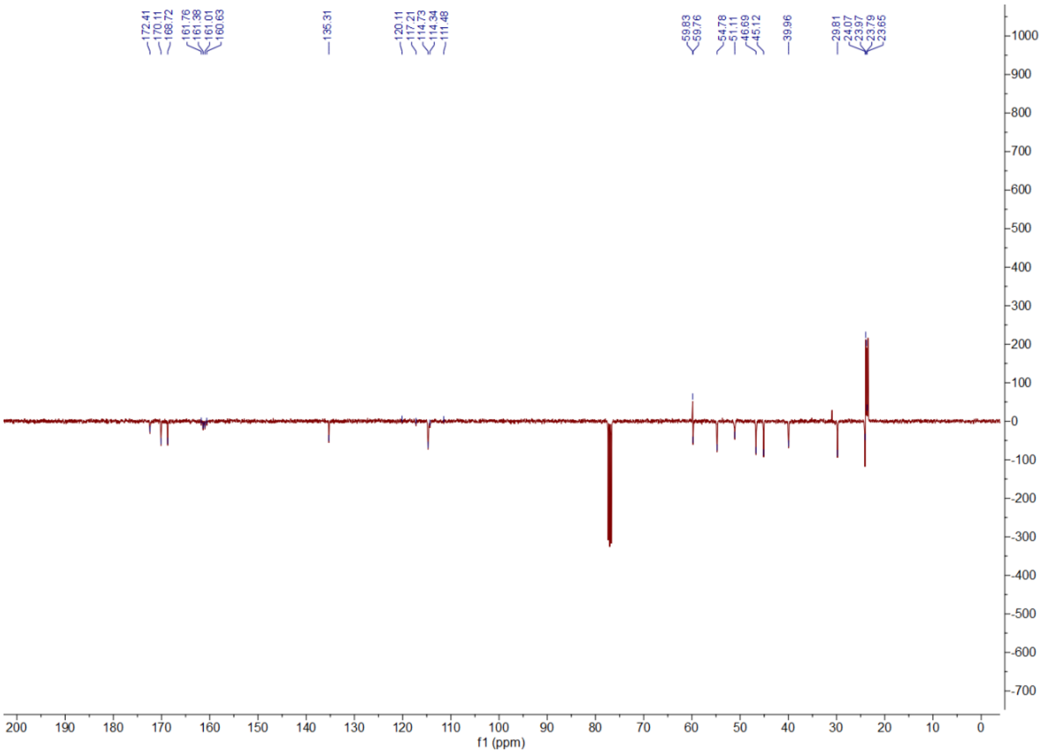

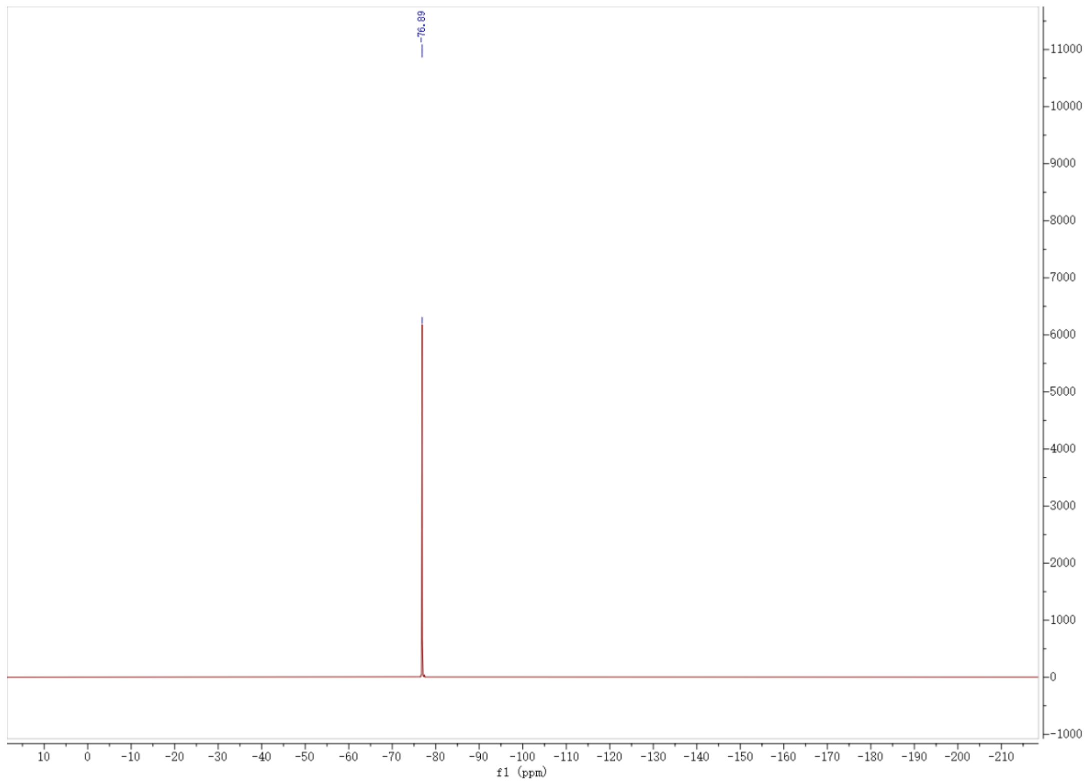
-76.89
f1 (ppm)

## nOe Analysis of compound 9 and 12

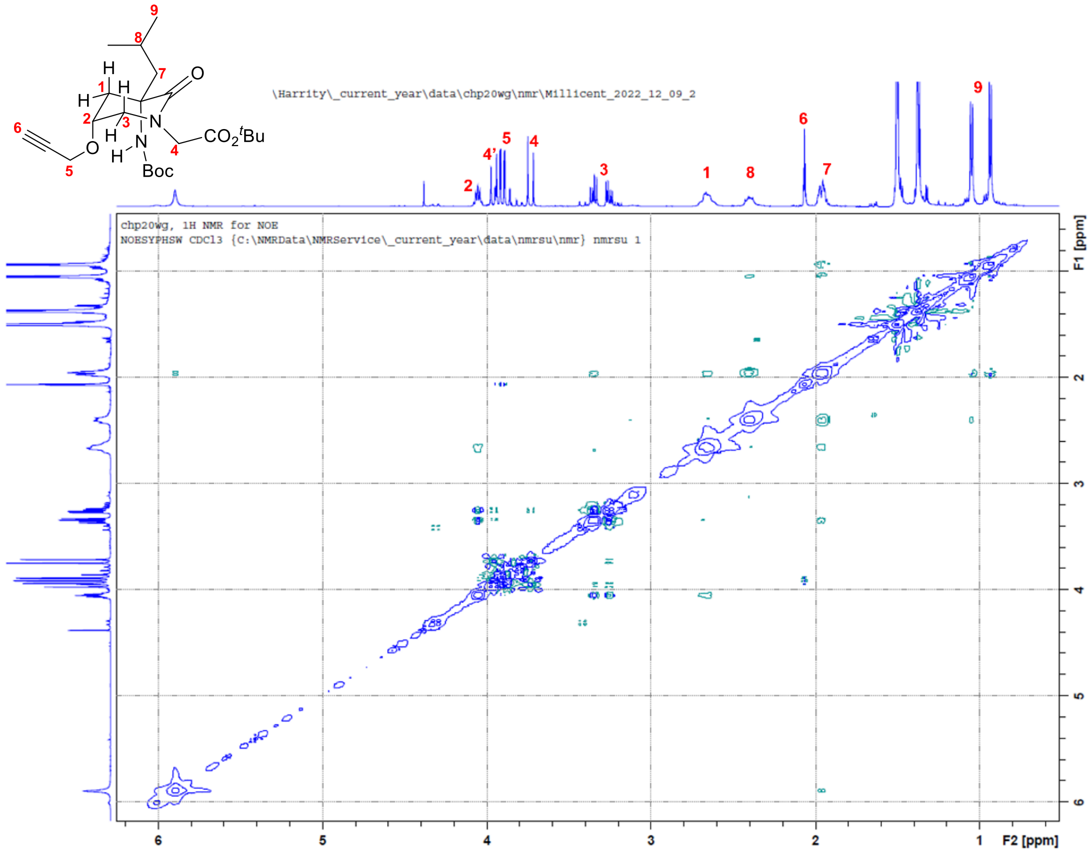


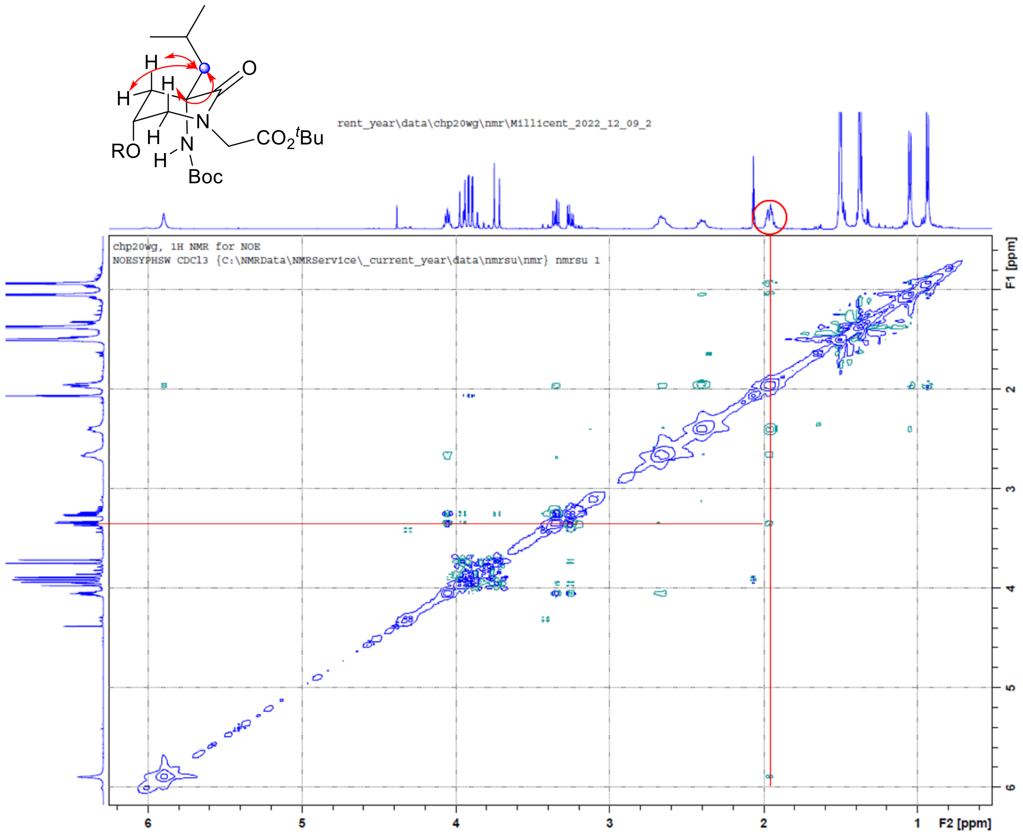

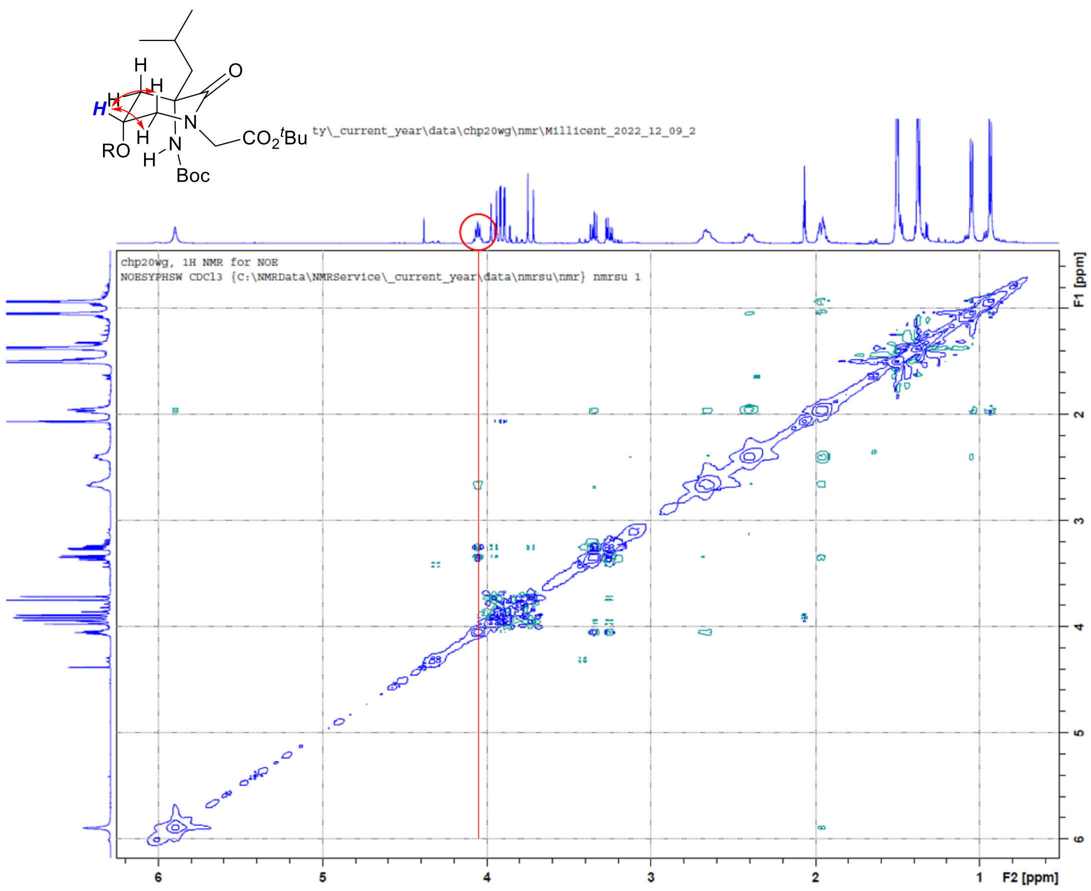

H
H
H
H
N
O
RO
H
N
CO2tBu
Boc
ty_current_year\data\chp20wg\nmr\Millicent_2022_12_09_2
chp20wg, 1H NMR for NOE
NOESYPHSW CDCl3 {C:\NMRData\NMRService_current_year\data\nmrsu\nmr} nmrsu 1
F1 [ppm]
F2 [ppm]
6
5
4
3
2
1

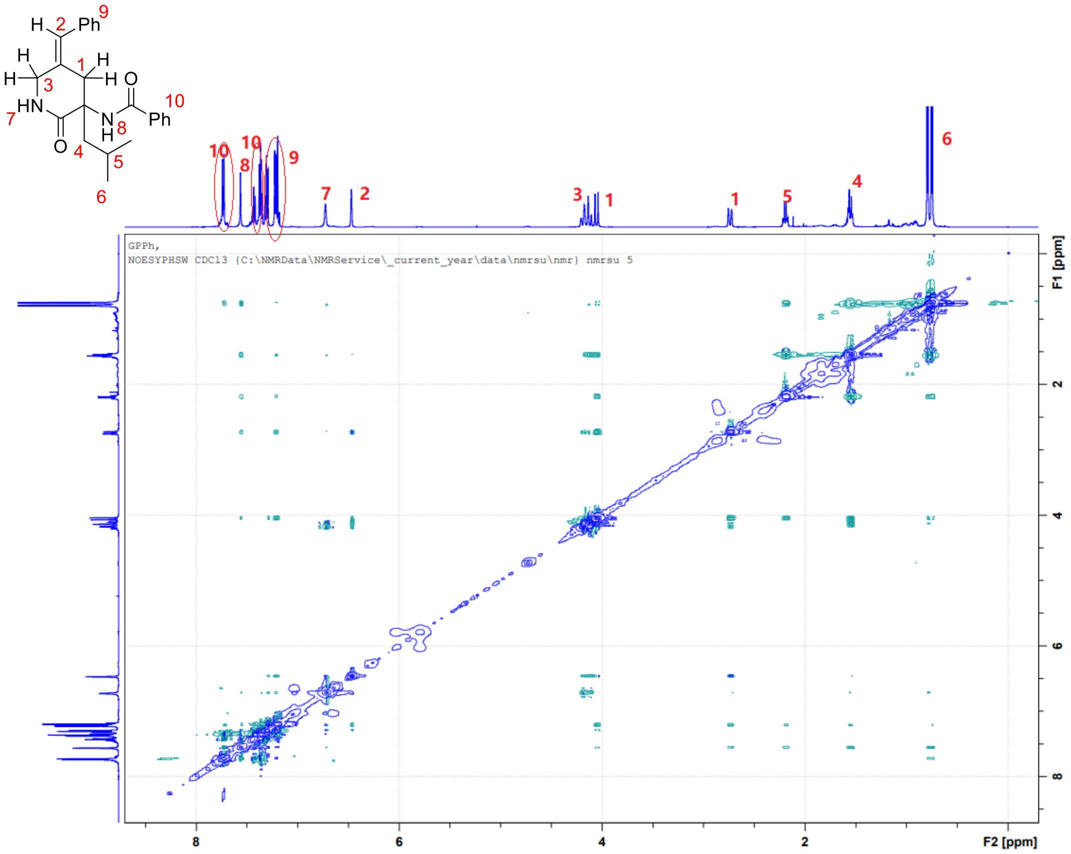

GPPh,
NOESYPHSW CDCl3 {C:\NMRData\NMRService_current_year\data\nmrsu\nmr} nmrsu 5
F1 [ppm]
F2 [ppm]
HN
Ph


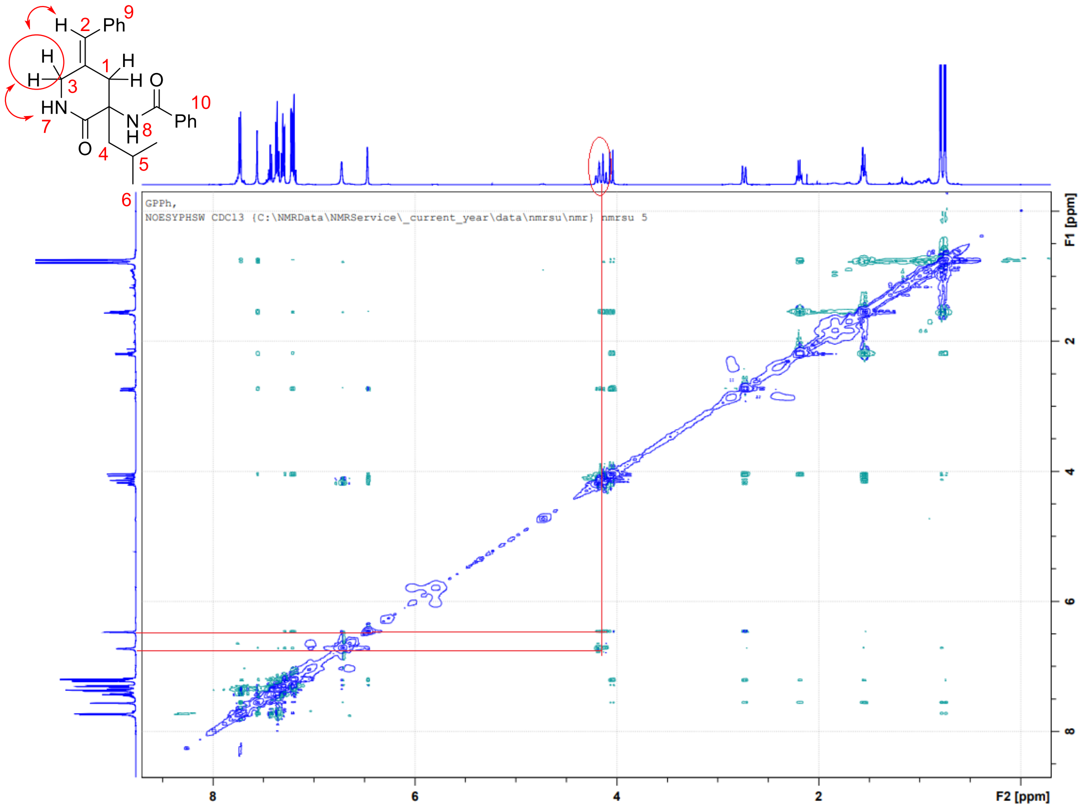

GPPh,
NOESYPHSW CDCl3 {C:\NMRData\NMRService_current_year\data\nmrsu\nmr} nmrsu 5
F1 [ppm]
F2 [ppm]
HN
Ph

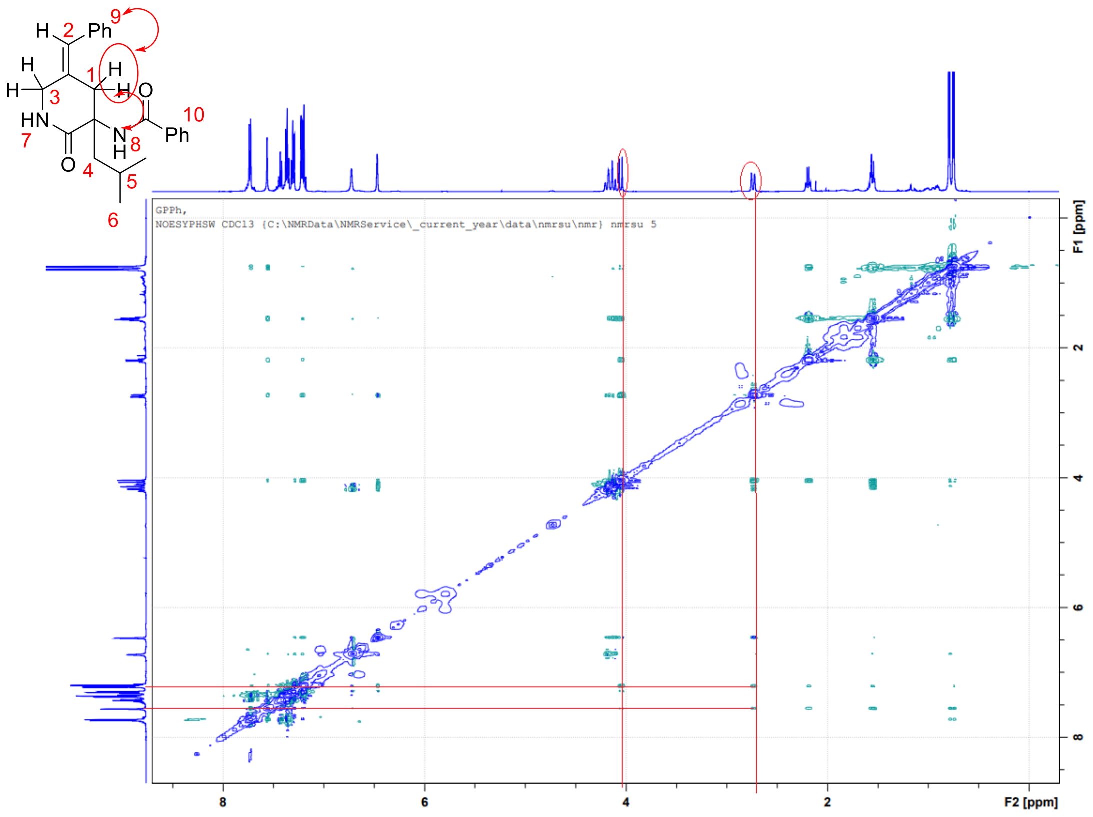

GPPh,
NOESYPHSW CDCl3 {C:\NMRData\NMRService_current_year\data\nmrsu\nmr} nmrsu 5
F1 [ppm]
F2 [ppm]

## HPLC traces of enantioenriched products

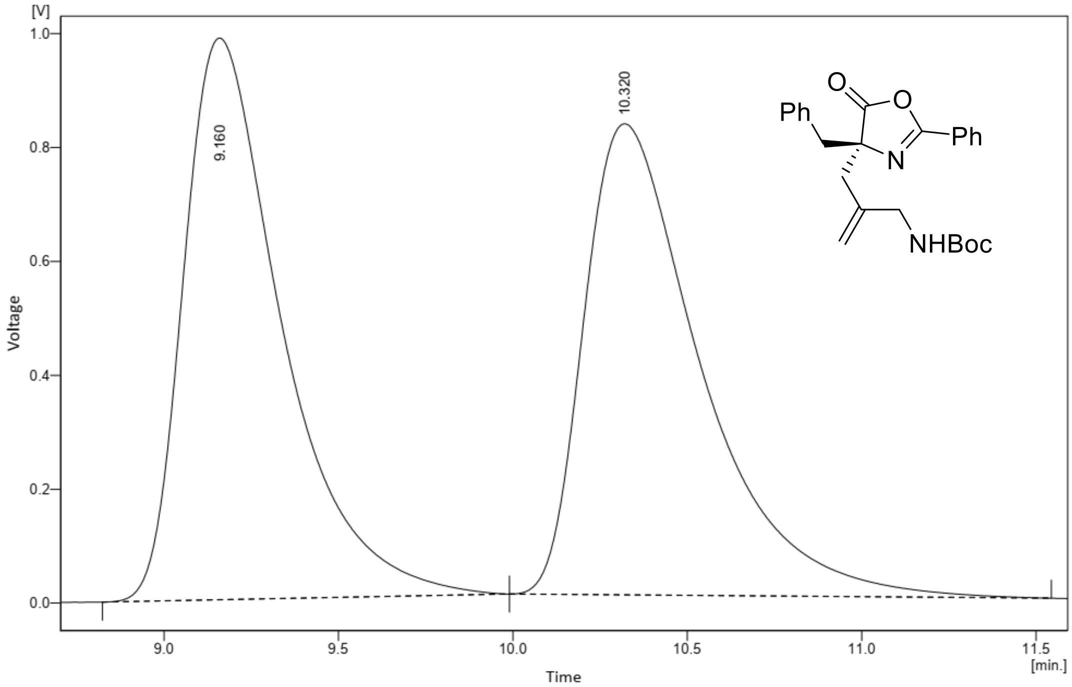


*Result Table - Calculation Method Uncal*

| | Reten. Time [min] | Area [mV.s] | Height [mV] | Area [%] | Height [%] | W05 [min] |
|---|---|---|---|---|---|---|
| 1 | 9.160 | 19336.033 | 986.302 | 50.0 | 54.4 | 0.29 |
| 2 | 10.320 | 19304.501 | 827.300 | 50.0 | 45.6 | 0.34 |
| | Total | 38640.534 | 1813.602 | 100.0 | 100.0 | |

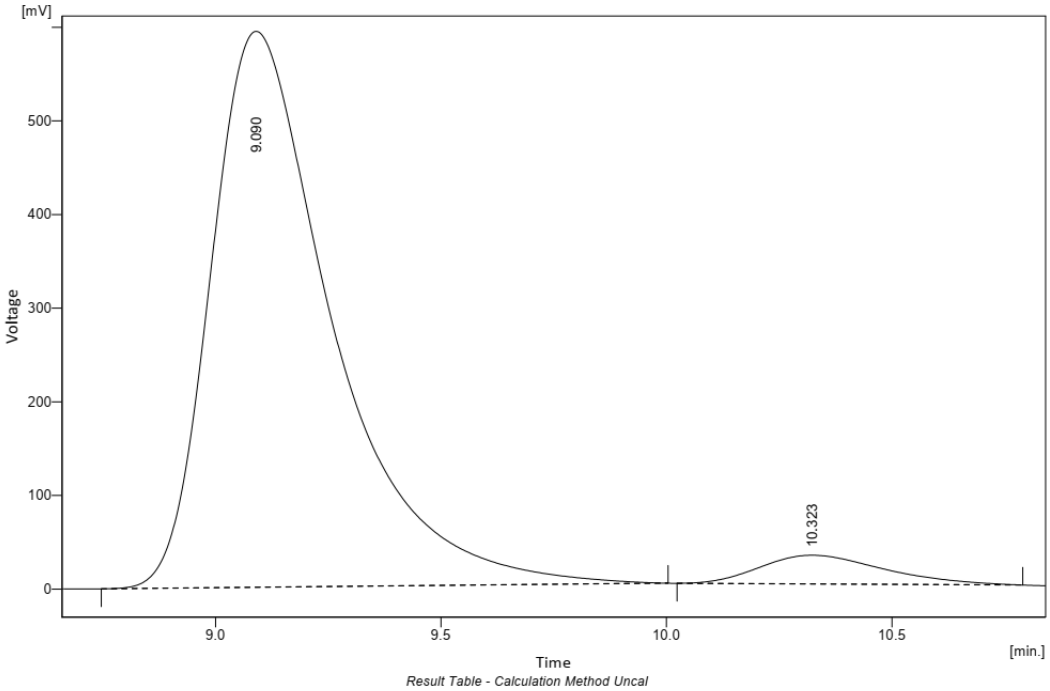


*Result Table - Calculation Method Uncal*

| | Reten. Time [min] | Area [mV.s] | Height [mV] | Area [%] | Height [%] | W05 [min] |
|---|---|---|---|---|---|---|
| 1 | 9.090 | 11138.131 | 593.789 | 95.0 | 95.1 | 0.27 |
| 2 | 10.323 | 586.937 | 30.710 | 5.0 | 4.9 | 0.30 |
| | Total | 11725.069 | 624.500 | 100.0 | 100.0 | |

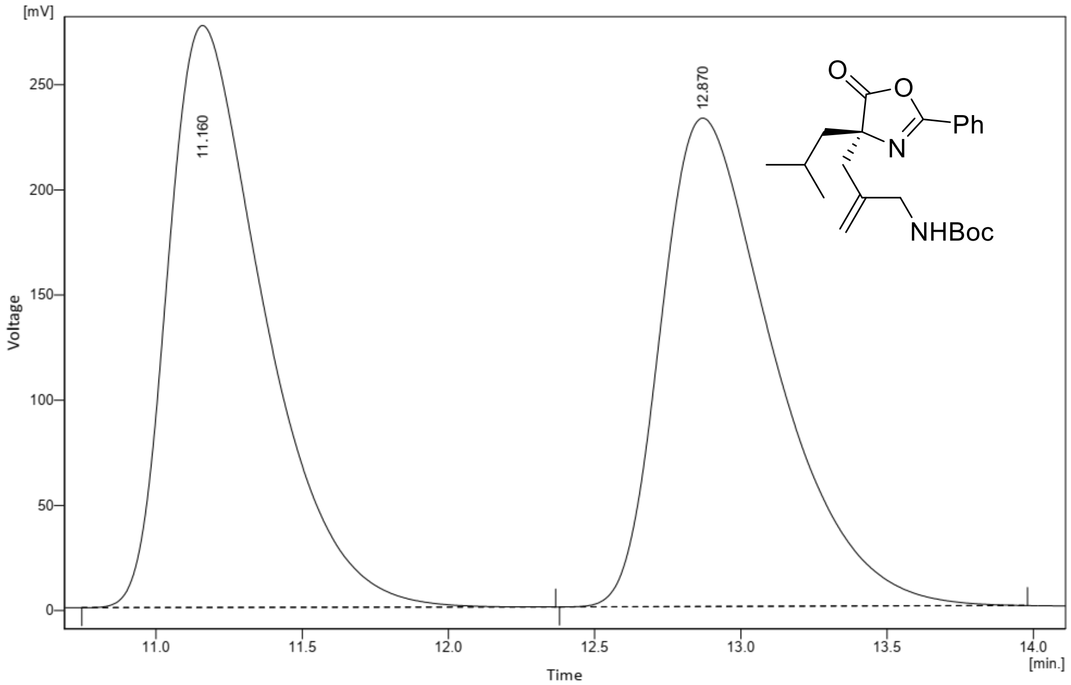


*Result Table - Calculation Method Uncal*

| | Reten. Time [min] | Area [mV.s] | Height [mV] | Area [%] | Height [%] | W05 [min] |
|---|---|---|---|---|---|---|
| 1 | 11.160 | 6356.519 | 276.771 | 50.4 | 54.4 | 0.35 |
| 2 | 12.870 | 6257.937 | 232.235 | 49.6 | 45.6 | 0.41 |
| | Total | 12614.455 | 509.006 | 100.0 | 100.0 | |

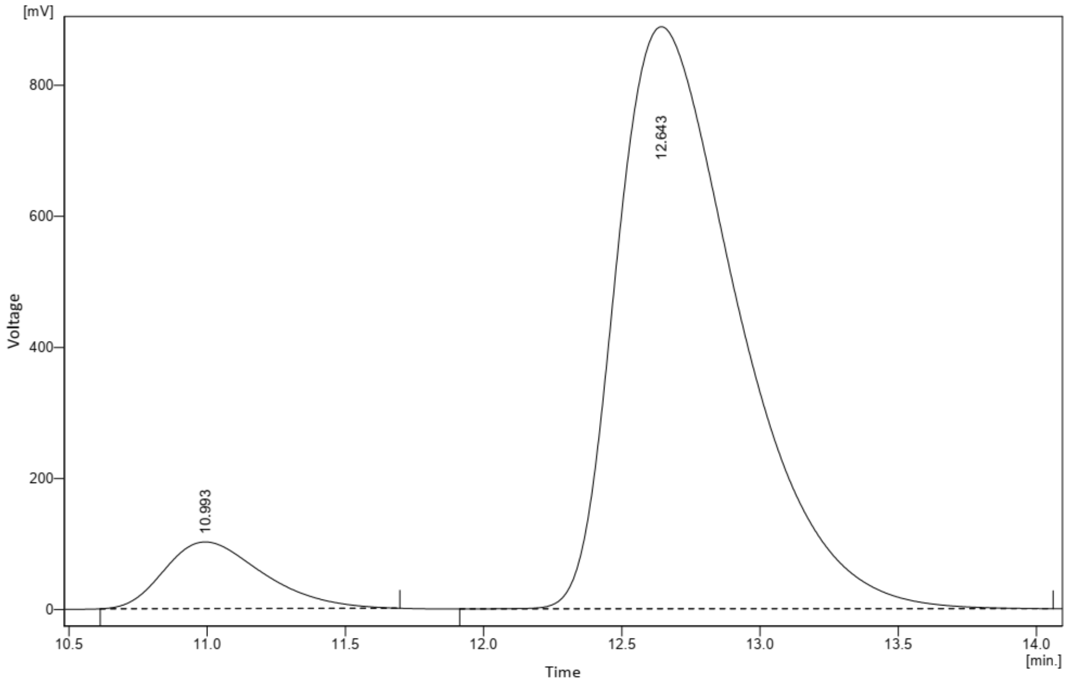


*Result Table - Calculation Method Uncal*

| | Reten. Time [min] | Area [mV.s] | Height [mV] | Area [%] | Height [%] | W05 [min] |
|---|---|---|---|---|---|---|
| 1 | 10.993 | 2532.736 | 101.797 | 8.5 | 10.3 | 0.39 |
| 2 | 12.643 | 27230.439 | 888.472 | 91.5 | 89.7 | 0.47 |
| | Total | 29763.175 | 990.268 | 100.0 | 100.0 | |

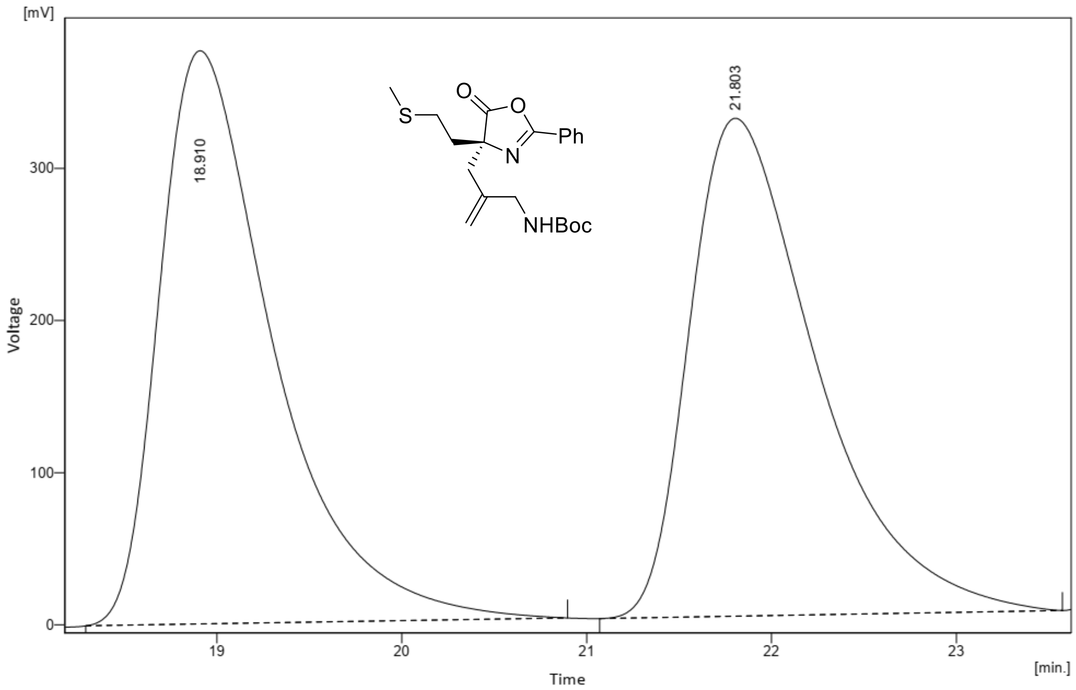


*Result Table - Calculation Method Uncal*

| | Reten. Time [min] | Area [mV.s] | Height [mV] | Area [%] | Height [%] | W05 [min] |
|---|---|---|---|---|---|---|
| 1 | 18.910 | 16185.670 | 376.904 | 50.8 | 53.5 | 0.63 |
| 2 | 21.803 | 15688.113 | 327.365 | 49.2 | 46.5 | 0.73 |
| | Total | 31873.782 | 704.269 | 100.0 | 100.0 | |

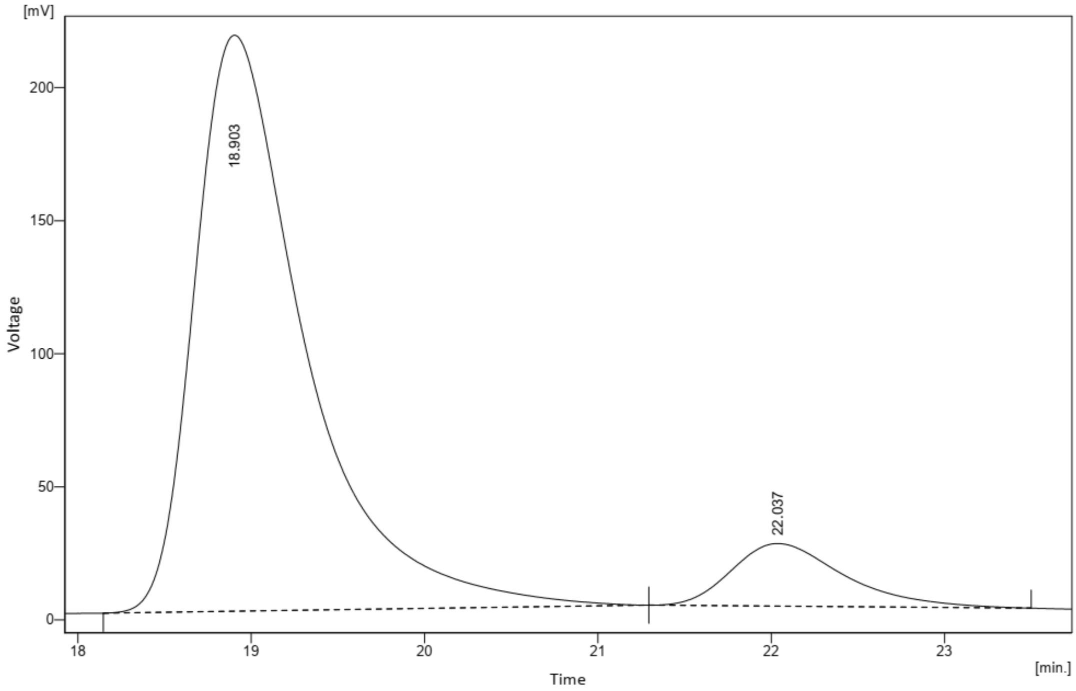


*Result Table - Calculation Method Uncal*

| | Reten. Time [min] | Area [mV.s] | Height [mV] | Area [%] | Height [%] | W05 [min] |
|---|---|---|---|---|---|---|
| 1 | 18.903 | 9629.384 | 216.443 | 90.0 | 90.2 | 0.64 |
| 2 | 22.037 | 1064.248 | 23.557 | 10.0 | 9.8 | 0.68 |
| | Total | 10693.632 | 240.000 | 100.0 | 100.0 | |

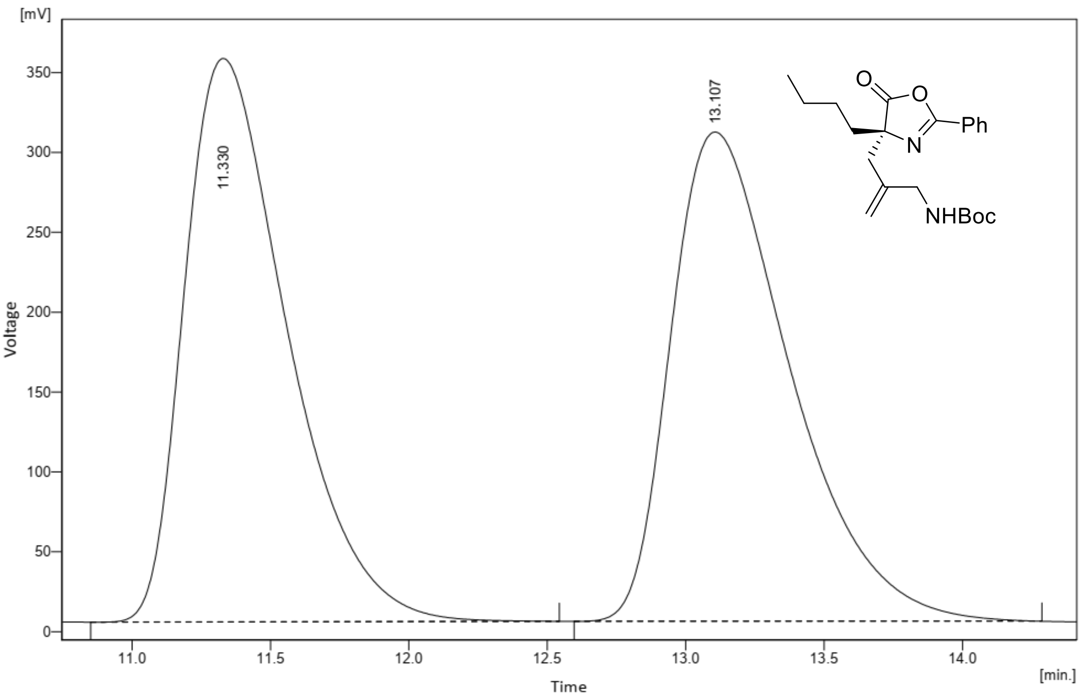


*Result Table - Calculation Method Uncal*

| | Reten. Time [min] | Area [mV.s] | Height [mV] | Area [%] | Height [%] | W05 [min] |
|---|---|---|---|---|---|---|
| 1 | 11.330 | 9103.382 | 352.795 | 49.9 | 53.5 | 0.40 |
| 2 | 13.107 | 9150.940 | 306.364 | 50.1 | 46.5 | 0.46 |
| | Total | 18254.322 | 659.159 | 100.0 | 100.0 | |

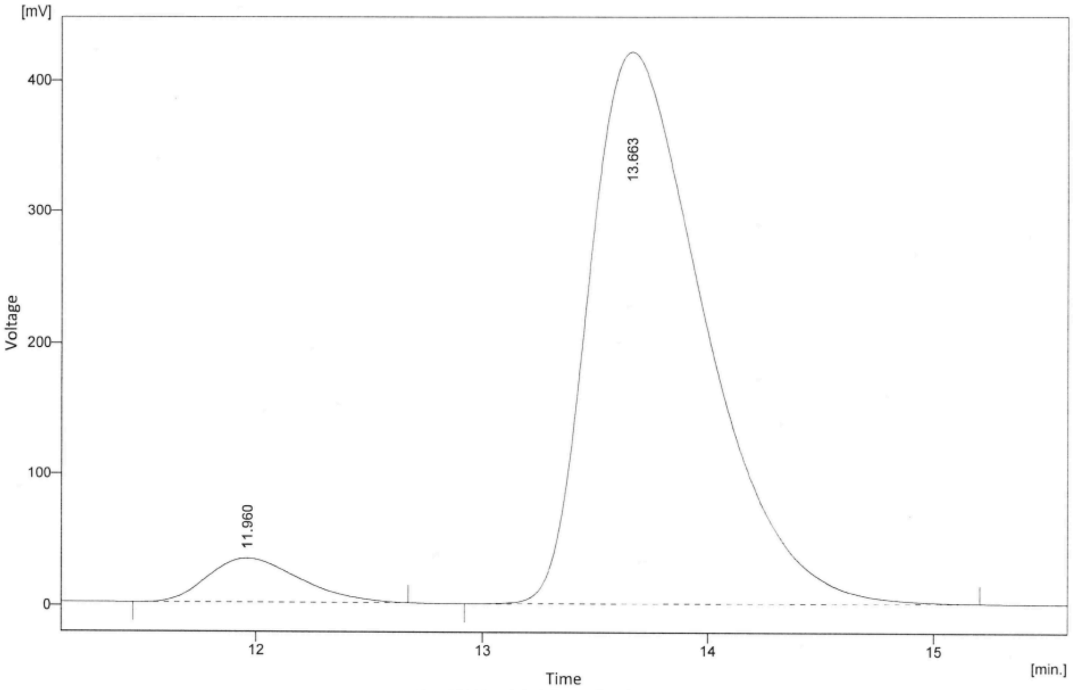


*Result Table - Calculation Method Uncal*

| | Reten. Time [min] | Area [mV.s] | Height [mV] | Area [%] | Height [%] | W05 [min] |
|---|---|---|---|---|---|---|
| 1 | 11.960 | 957.502 | 33.861 | 6.0 | 7.5 | 0.44 |
| 2 | 13.663 | 14887.215 | 419.707 | 94.0 | 92.5 | 0.55 |
| | Total | 15844.717 | 453.568 | 100.0 | 100.0 | |

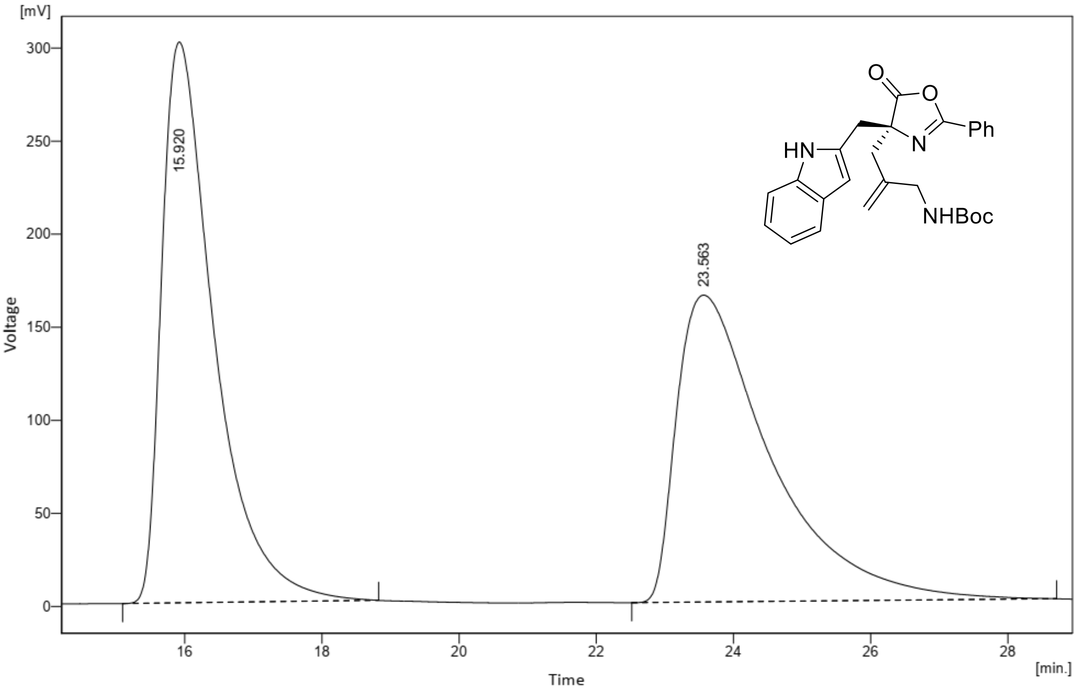


*Result Table - Calculation Method Uncal*

| | Reten. Time [min] | Area [mV.s] | Height [mV] | Area [%] | Height [%] | W05 [min] |
|---|---|---|---|---|---|---|
| 1 | 15.920 | 16036.522 | 301.407 | 50.5 | 64.6 | 0.78 |
| 2 | 23.563 | 15722.725 | 164.951 | 49.5 | 35.4 | 1.37 |
| | Total | 31759.247 | 466.358 | 100.0 | 100.0 | |

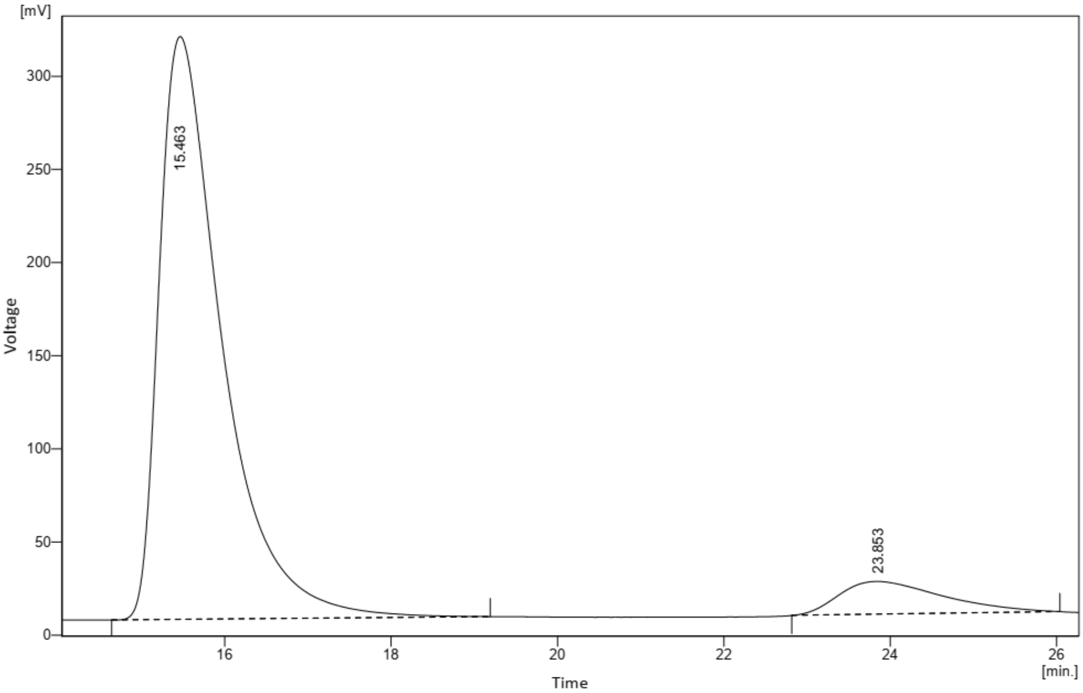


*Result Table - Calculation Method Uncal*

| | Reten. Time [min] | Area [mV.s] | Height [mV] | Area [%] | Height [%] | W05 [min] |
|---|---|---|---|---|---|---|
| 1 | 15.463 | 16462.682 | 312.884 | 91.5 | 94.7 | 0.77 |
| 2 | 23.853 | 1520.934 | 17.612 | 8.5 | 5.3 | 1.39 |
| | Total | 17983.616 | 330.496 | 100.0 | 100.0 | |

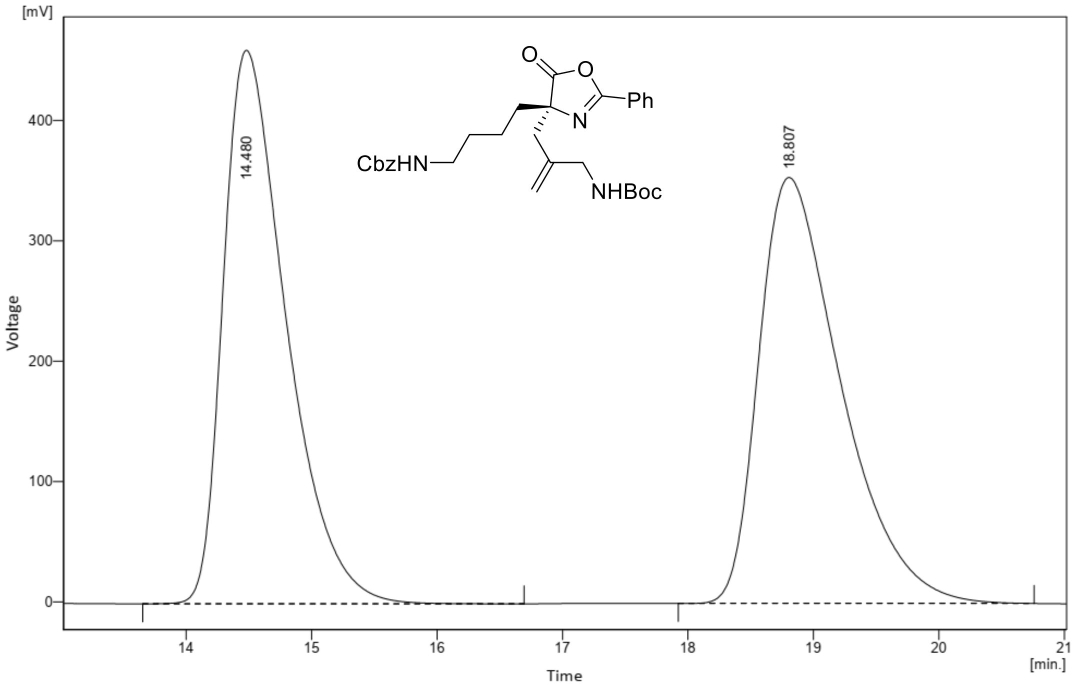


*Result Table - Calculation Method Uncal*

| | Reten. Time [min] | Area [mV.s] | Height [mV] | Area [%] | Height [%] | W05 [min] |
|---|---|---|---|---|---|---|
| 1 | 14.480 | 16036.370 | 459.934 | 49.9 | 56.5 | 0.53 |
| 2 | 18.807 | 16084.374 | 353.992 | 50.1 | 43.5 | 0.70 |
| | Total | 32120.744 | 813.926 | 100.0 | 100.0 | |

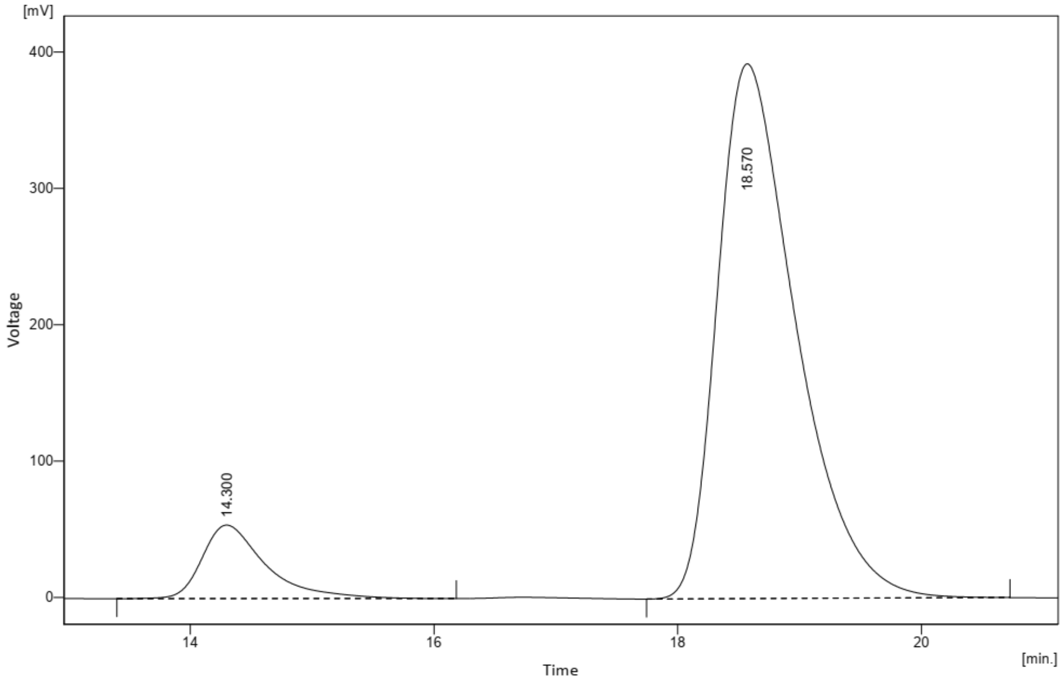


*Result Table - Calculation Method Uncal*

| | Reten. Time [min] | Area [mV.s] | Height [mV] | Area [%] | Height [%] | W05 [min] |
|---|---|---|---|---|---|---|
| 1 | 14.300 | 1961.340 | 53.998 | 10.0 | 12.1 | 0.52 |
| 2 | 18.570 | 17586.055 | 392.143 | 90.0 | 87.9 | 0.68 |
| | Total | 19547.395 | 446.141 | 100.0 | 100.0 | |

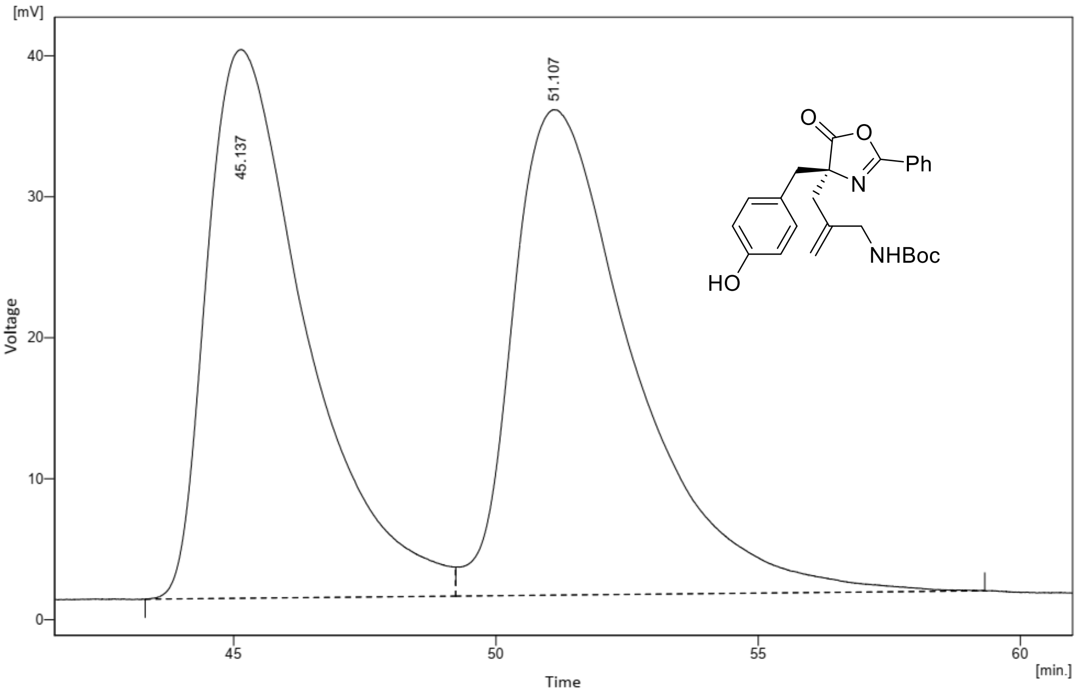


*Result Table - Calculation Method Uncal*

| | Reten. Time [min] | Area [mV.s] | Height [mV] | Area [%] | Height [%] | W05 [min] |
|---|---|---|---|---|---|---|
| 1 | 45.137 | 5168.248 | 38.918 | 48.2 | 53.1 | 1.95 |
| 2 | 51.107 | 5560.276 | 34.423 | 51.8 | 46.9 | 2.33 |
| | Total | 10728.524 | 73.341 | 100.0 | 100.0 | |

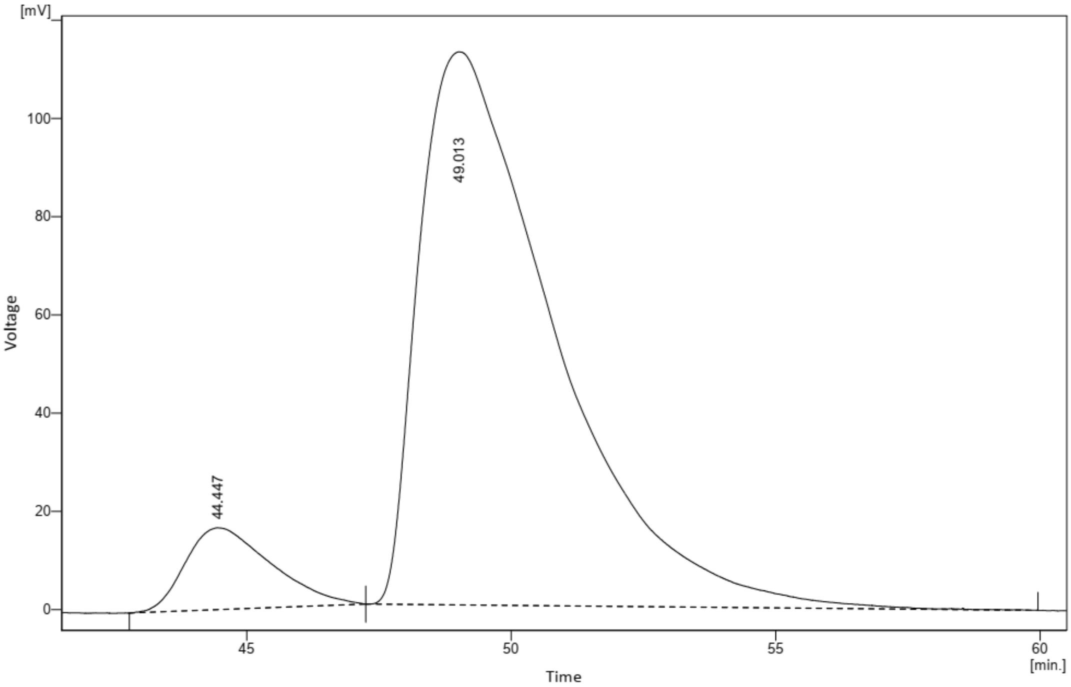


*Result Table - Calculation Method Uncal*

| | Reten. Time [min] | Area [mV.s] | Height [mV] | Area [%] | Height [%] | W05 [min] |
|---|---|---|---|---|---|---|
| 1 | 44.447 | 1879.213 | 16.693 | 8.5 | 12.9 | 1.82 |
| 2 | 49.013 | 20120.396 | 112.609 | 91.5 | 87.1 | 2.67 |
| | Total | 21999.609 | 129.302 | 100.0 | 100.0 | |

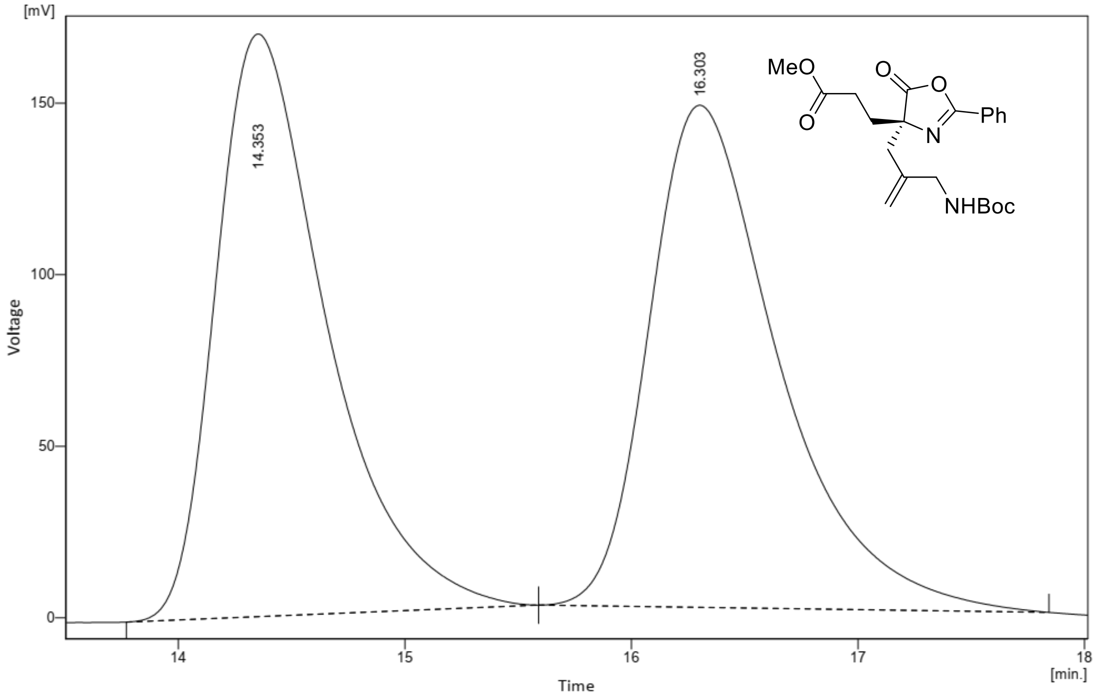


*Result Table - Calculation Method Uncal*

| | Reten. Time [min] | Area [mV.s] | Height [mV] | Area [%] | Height [%] | W05 [min] |
|---|---|---|---|---|---|---|
| 1 | 14.353 | 5791.240 | 169.779 | 49.9 | 53.7 | 0.51 |
| 2 | 16.303 | 5822.586 | 146.332 | 50.1 | 46.3 | 0.59 |
| | Total | 11613.826 | 316.111 | 100.0 | 100.0 | |

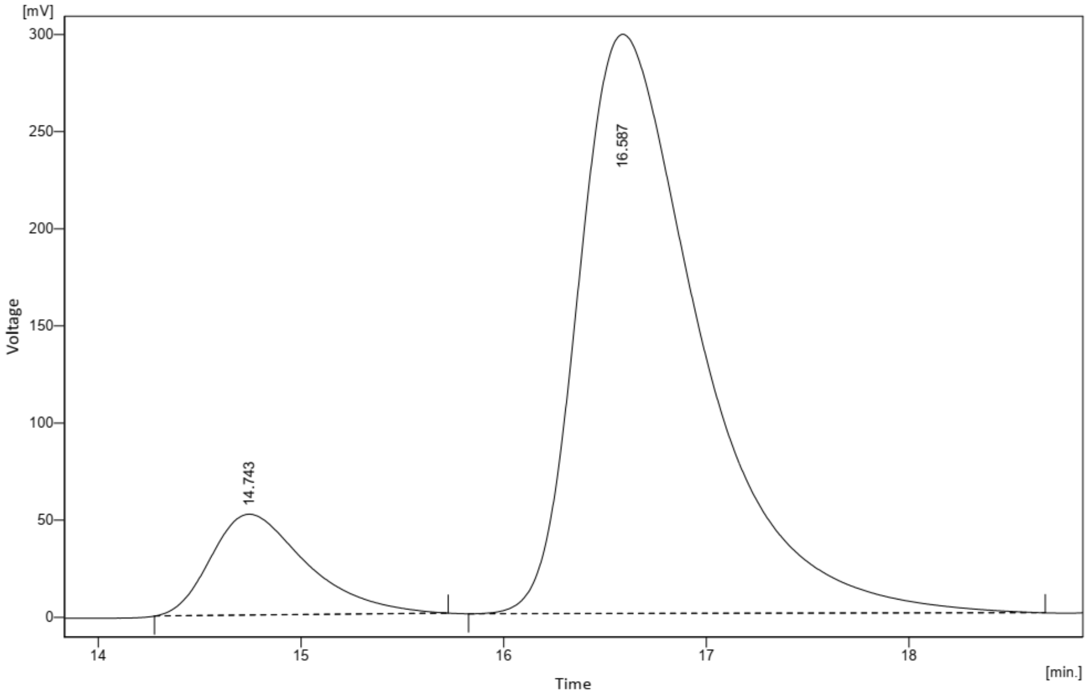


*Result Table - Calculation Method Uncal*

| | Reten. Time [min] | Area [mV.s] | Height [mV] | Area [%] | Height [%] | W05 [min] |
|---|---|---|---|---|---|---|
| 1 | 14.743 | 1710.315 | 51.974 | 12.1 | 14.8 | 0.50 |
| 2 | 16.587 | 12443.412 | 298.157 | 87.9 | 85.2 | 0.61 |
| | Total | 14153.727 | 350.131 | 100.0 | 100.0 | |

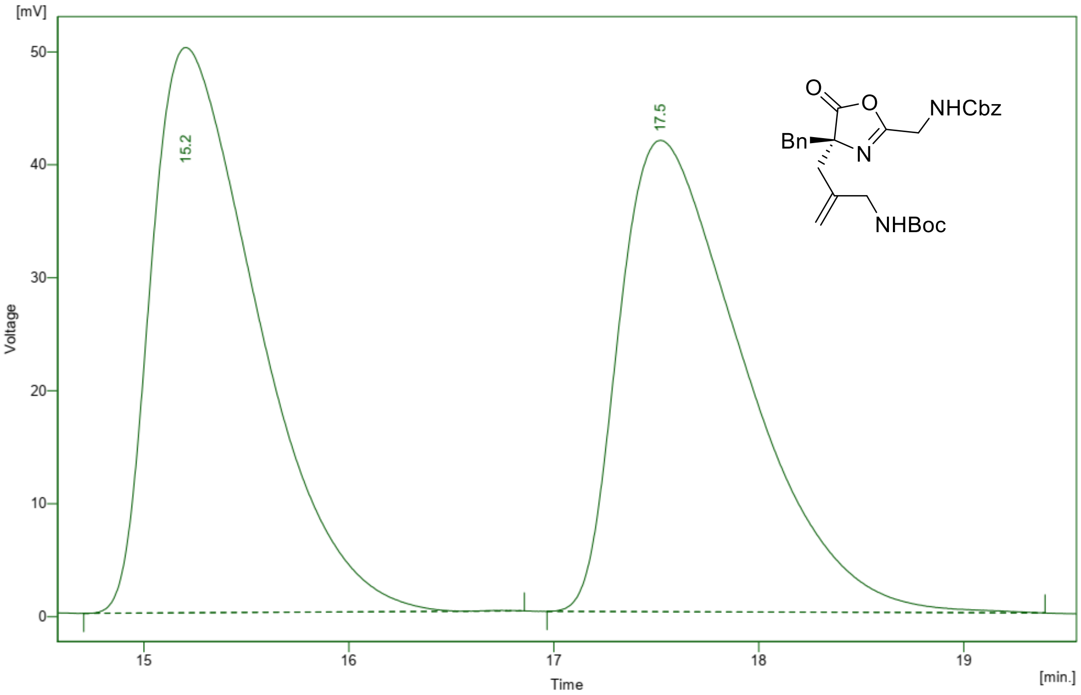


*Result Table - Calculation Method Uncal*

| | Reten. Time [min] | Area [mV.s] | Height [mV] | Area [%] | Height [%] | W05 [min] |
|---|---|---|---|---|---|---|
| 1 | 15.203 | 1813.368 | 50.046 | 50.5 | 54.5 | 0.57 |
| 2 | 17.520 | 1776.289 | 41.738 | 49.5 | 45.5 | 0.66 |
| | Total | 3589.658 | 91.784 | 100.0 | 100.0 | |

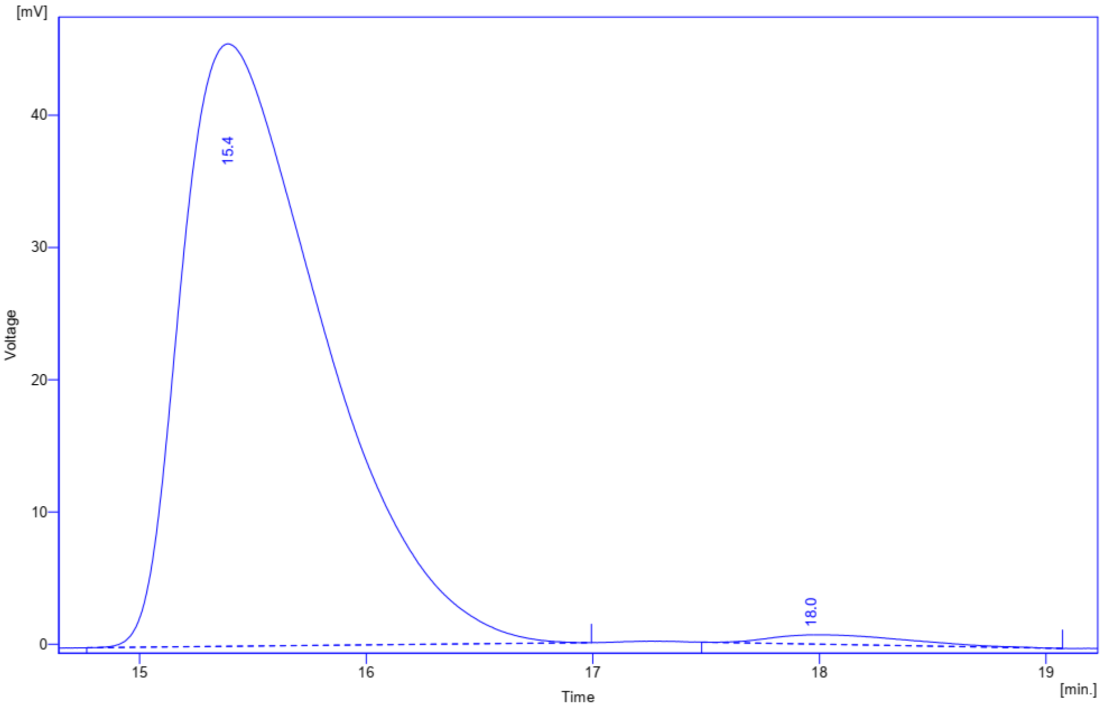


*Result Table - Calculation Method Uncal*

| | Reten. Time [min] | Area [mV.s] | Height [mV] | Area [%] | Height [%] | W05 [min] |
|---|---|---|---|---|---|---|
| 1 | 15.390 | 1978.594 | 45.556 | 98.5 | 98.5 | 0.68 |
| 2 | 17.963 | 30.842 | 0.708 | 1.5 | 1.5 | 0.62 |
| | Total | 2009.436 | 46.264 | 100.0 | 100.0 | |

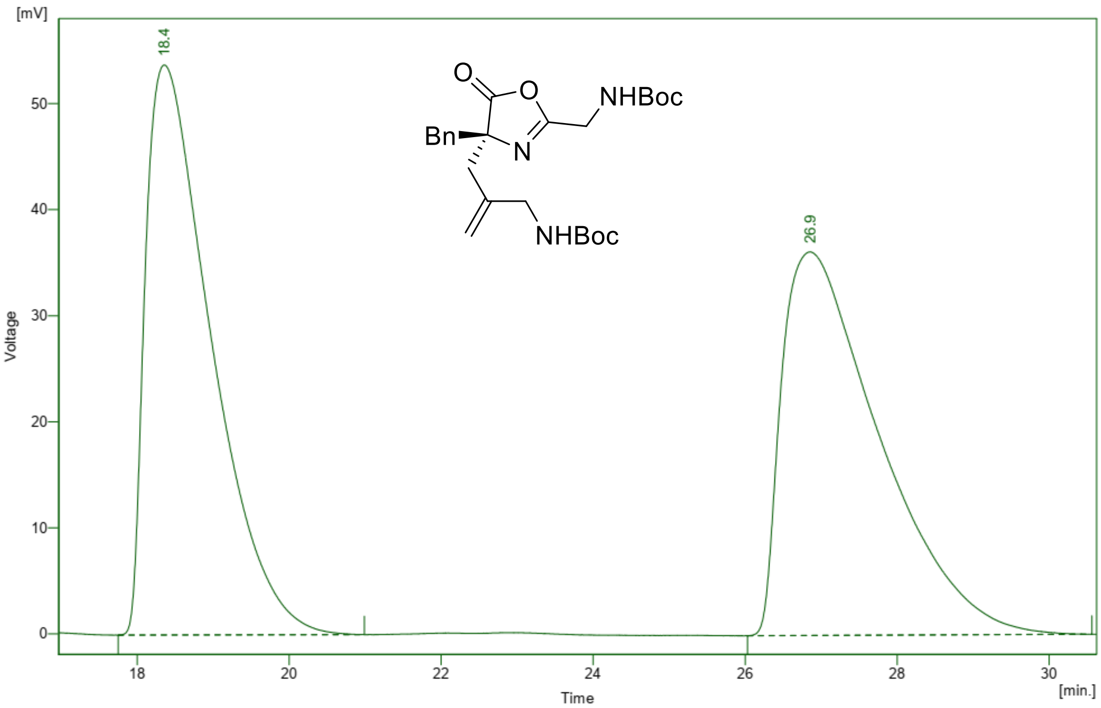


*Result Table - Calculation Method Uncal*

| | Reten. Time [min] | Area [mV.s] | Height [mV] | Area [%] | Height [%] | W05 [min] |
|---|---|---|---|---|---|---|
| 1 | 18.357 | 3227.753 | 53.761 | 50.0 | 59.8 | 0.93 |
| 2 | 26.857 | 3232.794 | 36.177 | 50.0 | 40.2 | 1.39 |
| | Total | 6460.546 | 89.938 | 100.0 | 100.0 | |

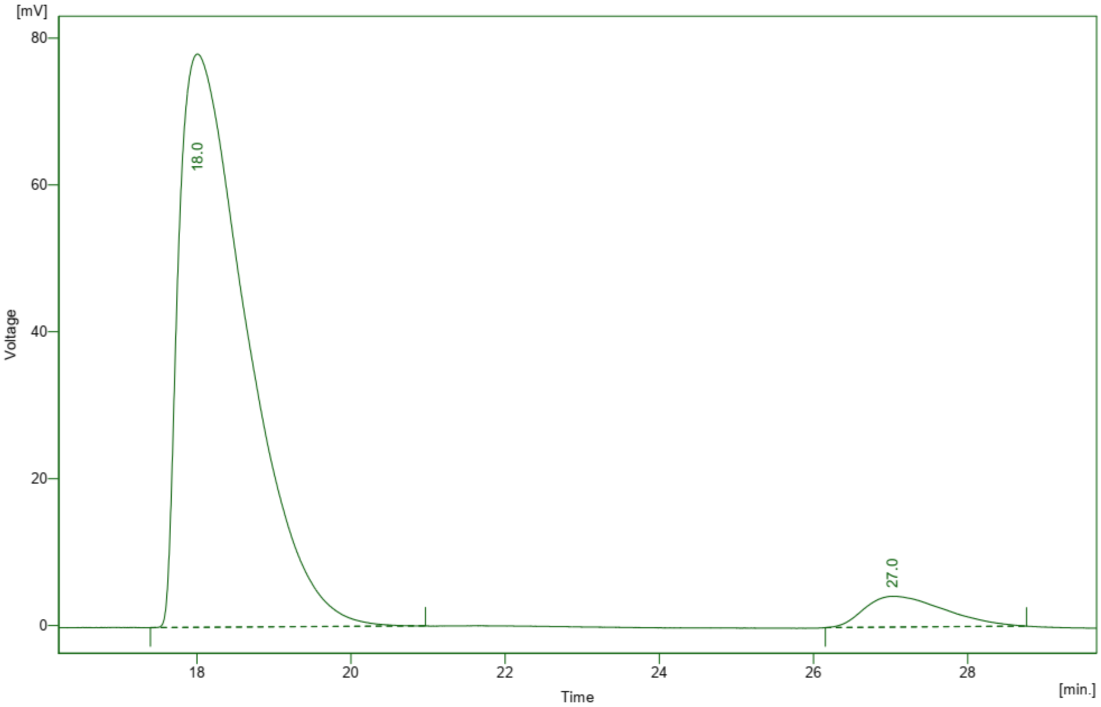


*Result Table - Calculation Method Uncal*

| | Reten. Time [min] | Area [mV.s] | Height [mV] | Area [%] | Height [%] | W05 [min] |
|---|---|---|---|---|---|---|
| 1 | 18.007 | 4785.101 | 78.098 | 94.0 | 94.9 | 0.95 |
| 2 | 27.023 | 304.291 | 4.224 | 6.0 | 5.1 | 1.17 |
| | Total | 5089.392 | 82.322 | 100.0 | 100.0 | |

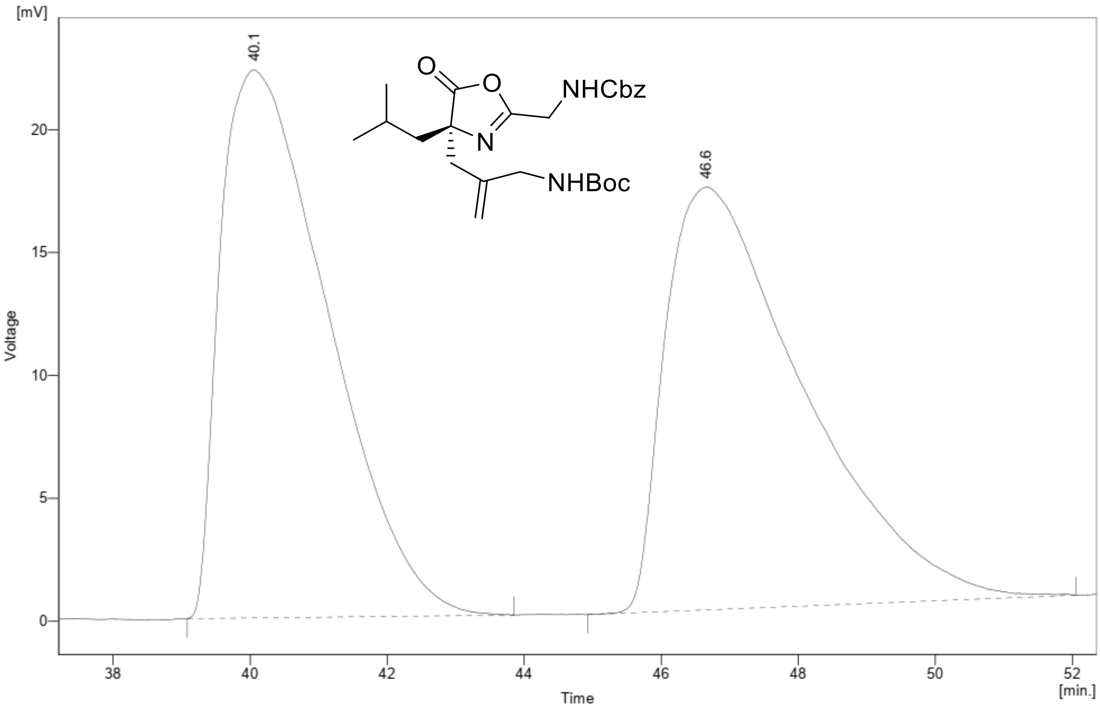


Result Table - Calculation Method Uncal

| | Reten. Time [min] | Area [mV.s] | Height [mV] | Area [%] | Height [%] | W05 [min] |
|---|---|---|---|---|---|---|
| 1 | 40.057 | 2431.961 | 22.305 | 50.1 | 56.5 | 1.77 |
| 2 | 46.647 | 2419.871 | 17.206 | 49.9 | 43.5 | 2.19 |
| | Total | 4851.832 | 39.512 | 100.0 | 100.0 | |

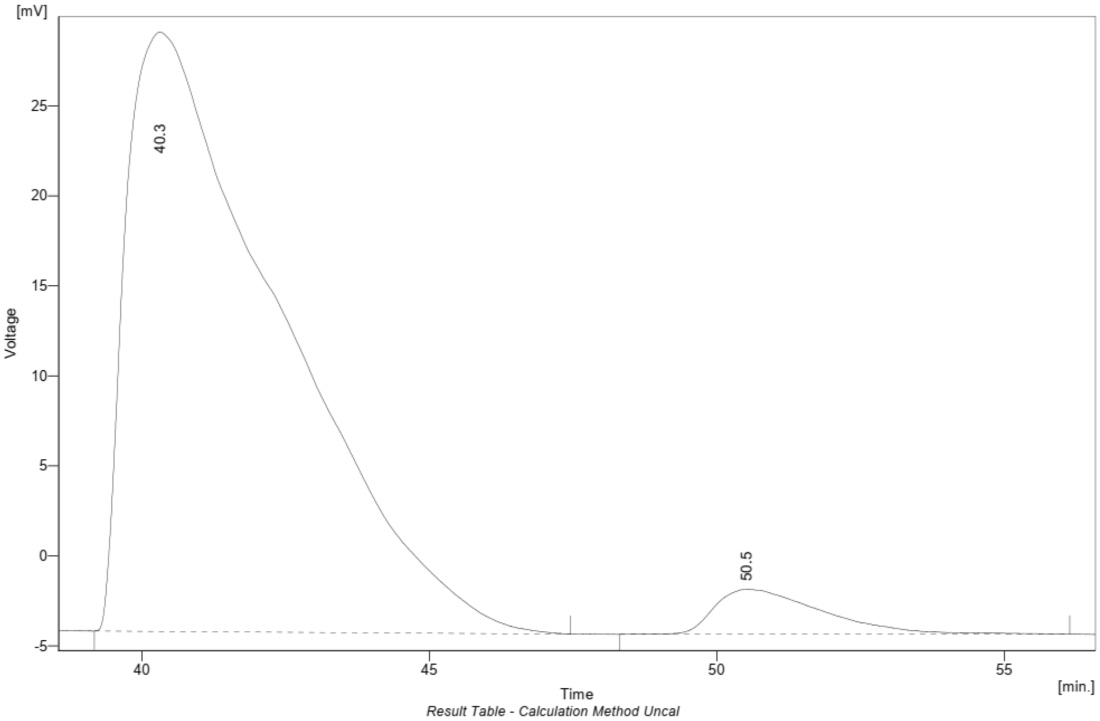


Result Table - Calculation Method Uncal

| | Reten. Time [min] | Area [mV.s] | Height [mV] | Area [%] | Height [%] | W05 [min] |
|---|---|---|---|---|---|---|
| 1 | 40.313 | 6238.968 | 33.328 | 95.0 | 93.0 | 3.00 |
| 2 | 50.510 | 326.399 | 2.510 | 5.0 | 7.0 | 1.99 |
| | Total | 6565.367 | 35.838 | 100.0 | 100.0 | |

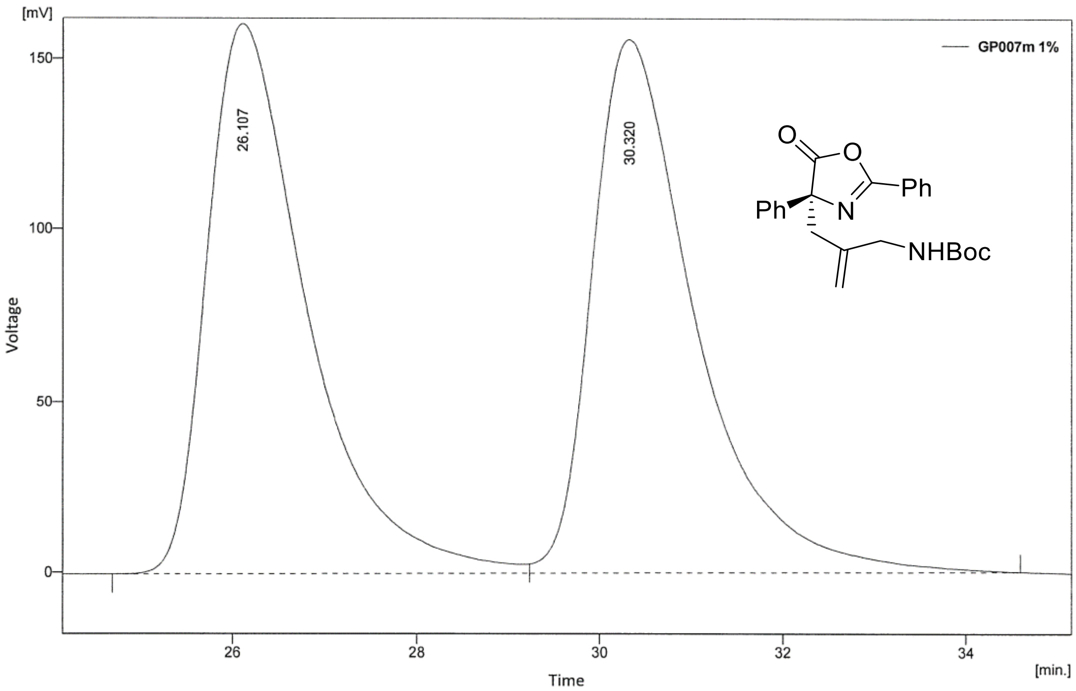


Result Table - Calculation Method Uncal

| | Reten. Time [min] | Area [mV.s] | Height [mV] | Area [%] | Height [%] | W05 [min] |
|---|---|---|---|---|---|---|
| 1 | 26.107 | 12196.083 | 160.629 | 49.9 | 50.7 | 1.11 |
| 2 | 30.320 | 12257.266 | 155.922 | 50.1 | 49.3 | 1.13 |
| | Total | 24453.350 | 316.551 | 100.0 | 100.0 | |

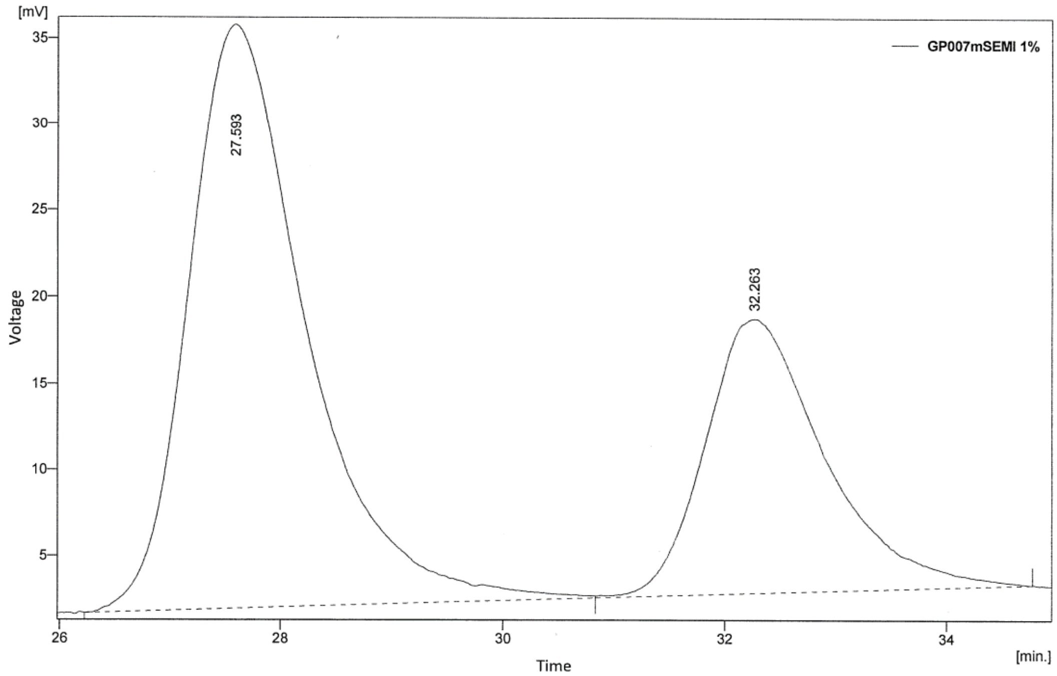


Result Table - Calculation Method Uncal

| | Reten. Time [min] | Area [mV.s] | Height [mV] | Area [%] | Height [%] | W05 [min] |
|---|---|---|---|---|---|---|
| 1 | 27.593 | 2486.201 | 33.823 | 67.6 | 68.0 | 1.07 |
| 2 | 32.263 | 1189.107 | 15.923 | 32.4 | 32.0 | 1.10 |
| | Total | 3675.307 | 49.746 | 100.0 | 100.0 | |

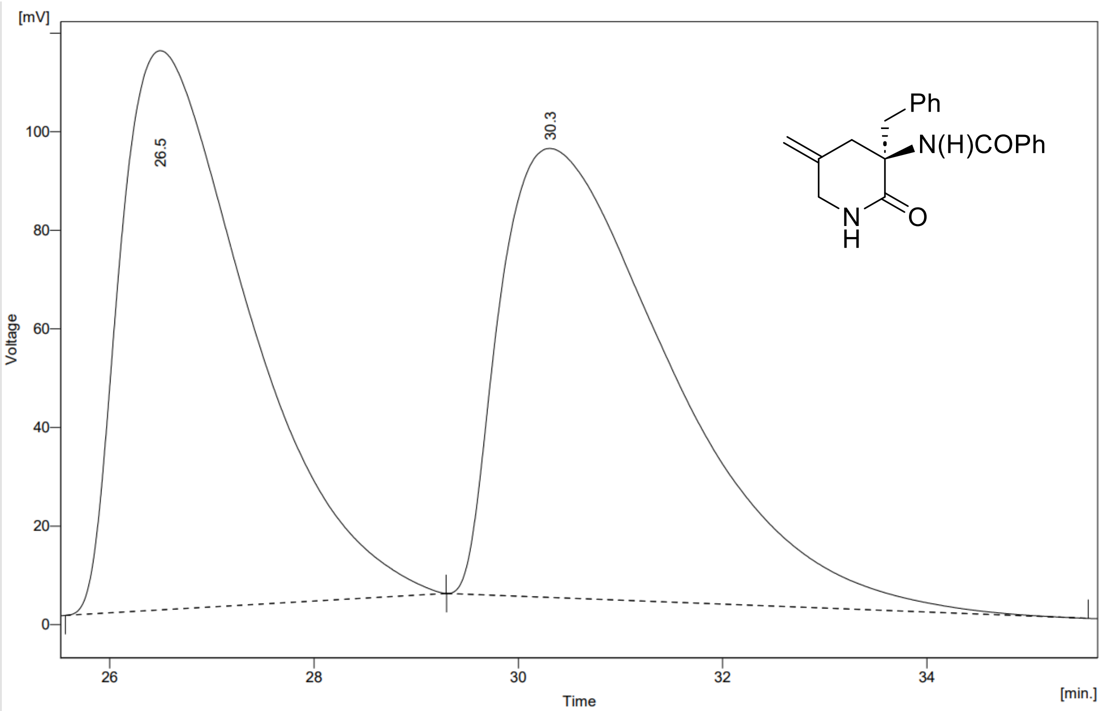


Result Table - Calculation Method Uncal

| | Reten. Time [min] | Area [mV.s] | Height [mV] | Area [%] | Height [%] | W05 [min] |
|---|---|---|---|---|---|---|
| 1 | 26.497 | 10001.141 | 113.455 | 48.3 | 55.5 | 1.38 |
| 2 | 30.313 | 10684.259 | 91.094 | 51.7 | 44.5 | 1.79 |
| | Total | 20685.400 | 204.549 | 100.0 | 100.0 | |

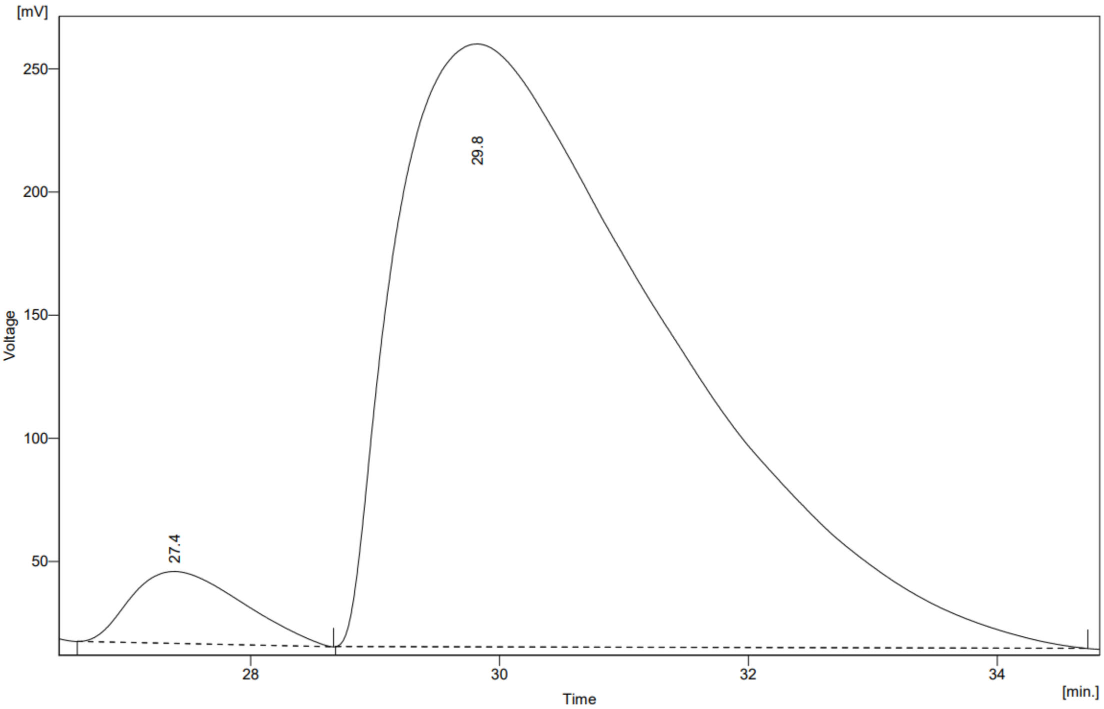


Result Table - Calculation Method Uncal

| | Reten. Time [min] | Area [mV.s] | Height [mV] | Area [%] | Height [%] | W05 [min] |
|---|---|---|---|---|---|---|
| 1 | 27.390 | 1828.369 | 29.257 | 4.7 | 10.7 | 1.02 |
| 2 | 29.823 | 37178.292 | 244.774 | 95.3 | 89.3 | 2.40 |
| | Total | 39006.661 | 274.031 | 100.0 | 100.0 | |

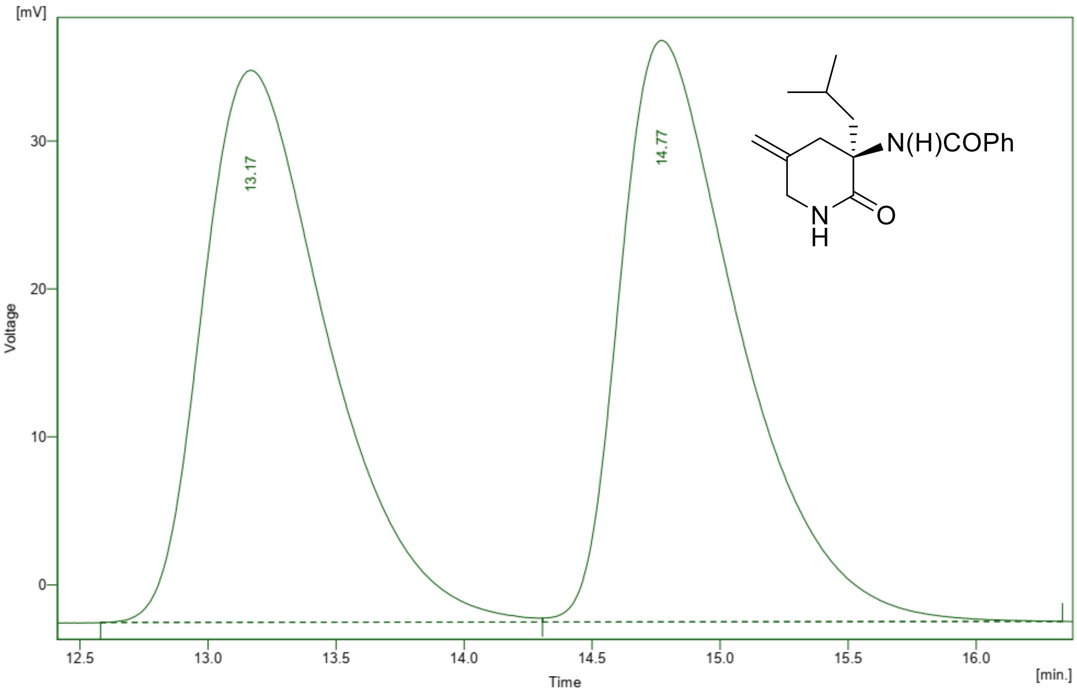


Result Table - Calculation Method Uncal

| | Reten. Time [min] | Area [mV.s] | Height [mV] | Area [%] | Height [%] | W05 [min] |
|---|---|---|---|---|---|---|
| 1 | 13.167 | 1266.759 | 37.316 | 49.9 | 48.7 | 0.52 |
| 2 | 14.773 | 1270.885 | 39.300 | 50.1 | 51.3 | 0.49 |
| | Total | 2537.644 | 76.615 | 100.0 | 100.0 | |

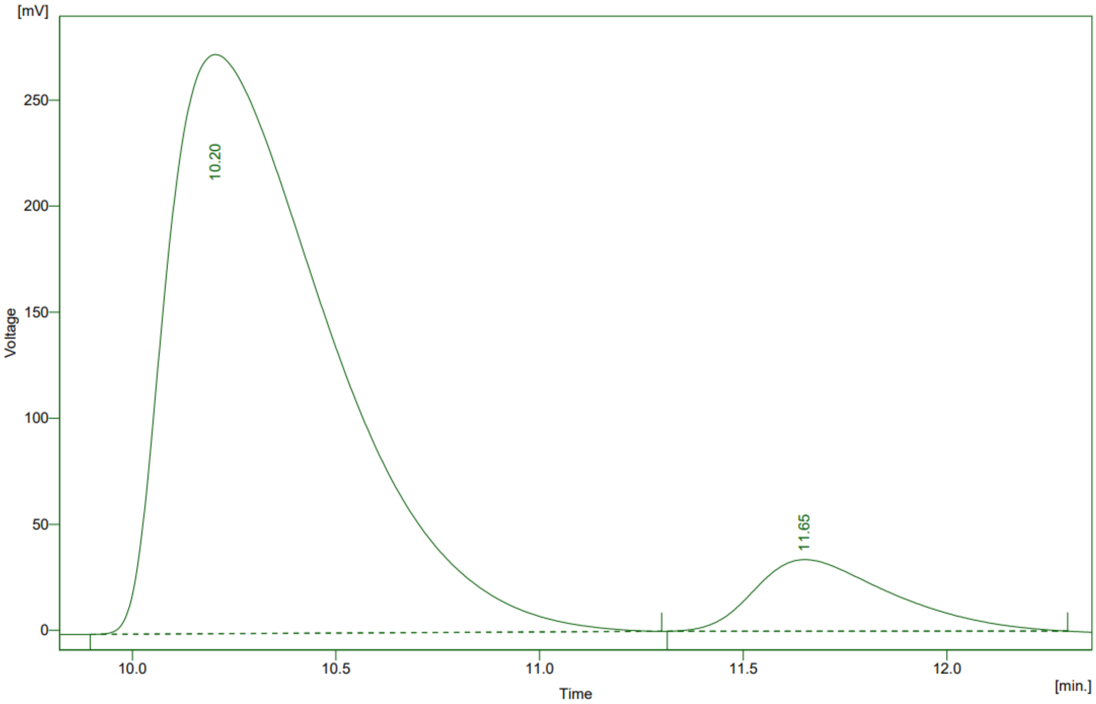


Result Table - Calculation Method Uncal

| | Reten. Time [min] | Area [mV.s] | Height [mV] | Area [%] | Height [%] | W05 [min] |
|---|---|---|---|---|---|---|
| 1 | 10.203 | 7556.713 | 273.106 | 90.5 | 89.0 | 0.43 |
| 2 | 11.650 | 791.978 | 33.791 | 9.5 | 11.0 | 0.36 |
| | Total | 8348.691 | 306.897 | 100.0 | 100.0 | |

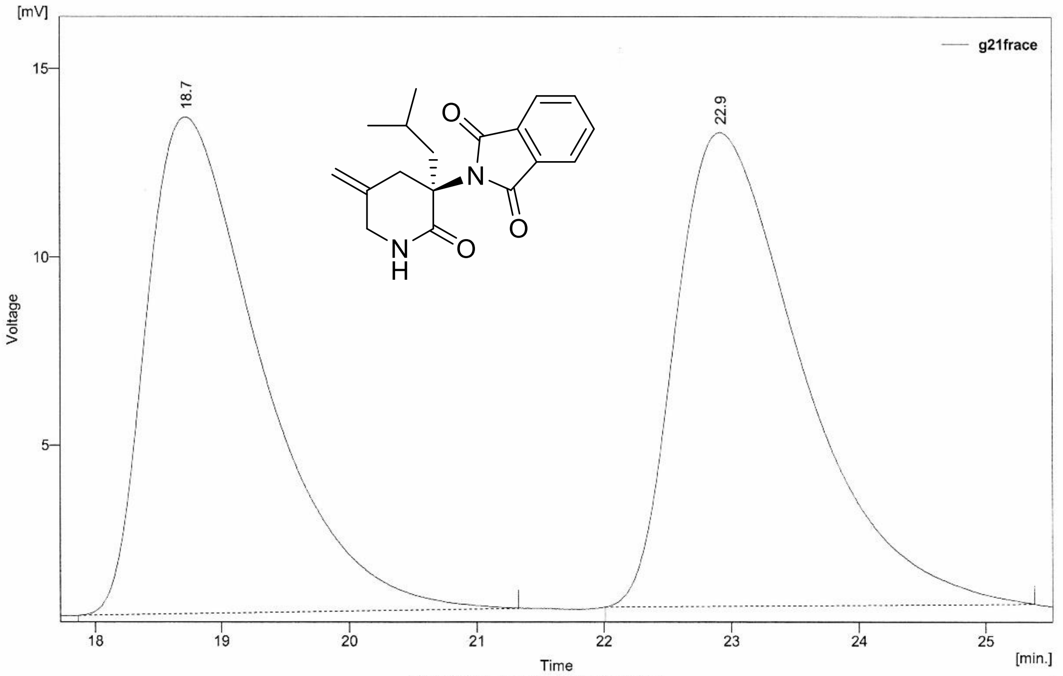


*Result Table - Calculation Method Uncal*

| | Reten. Time [min] | Area [mV.s] | Height [mV] | Area [%] | Height [%] | W05 [min] |
|---|---|---|---|---|---|---|
| 1 | 18.730 | 832.078 | 13.167 | 49.0 | 51.1 | 0.95 |
| 2 | 22.920 | 867.748 | 12.578 | 51.0 | 48.9 | 1.04 |
| | Total | 1699.826 | 25.745 | 100.0 | 100.0 | |

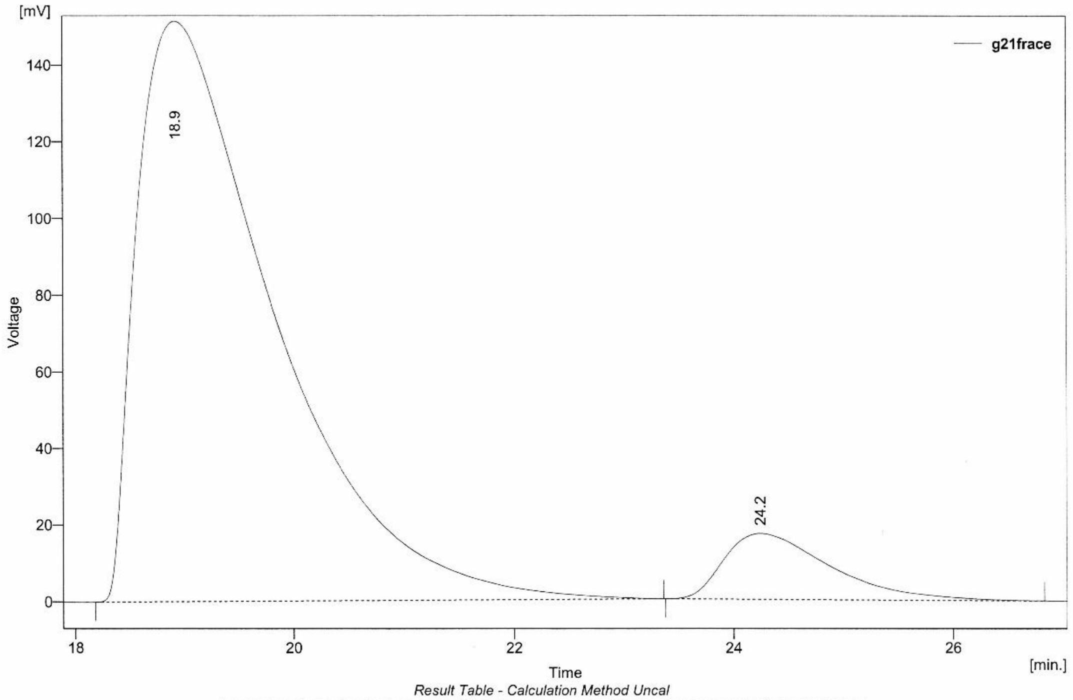


*Result Table - Calculation Method Uncal*

| | Reten. Time [min] | Area [mV.s] | Height [mV] | Area [%] | Height [%] | W05 [min] |
|---|---|---|---|---|---|---|
| 1 | 18.923 | 13249.768 | 151.483 | 92.0 | 89.8 | 1.32 |
| 2 | 24.240 | 1153.108 | 17.170 | 8.0 | 10.2 | 1.02 |
| | Total | 14402.876 | 168.653 | 100.0 | 100.0 | |

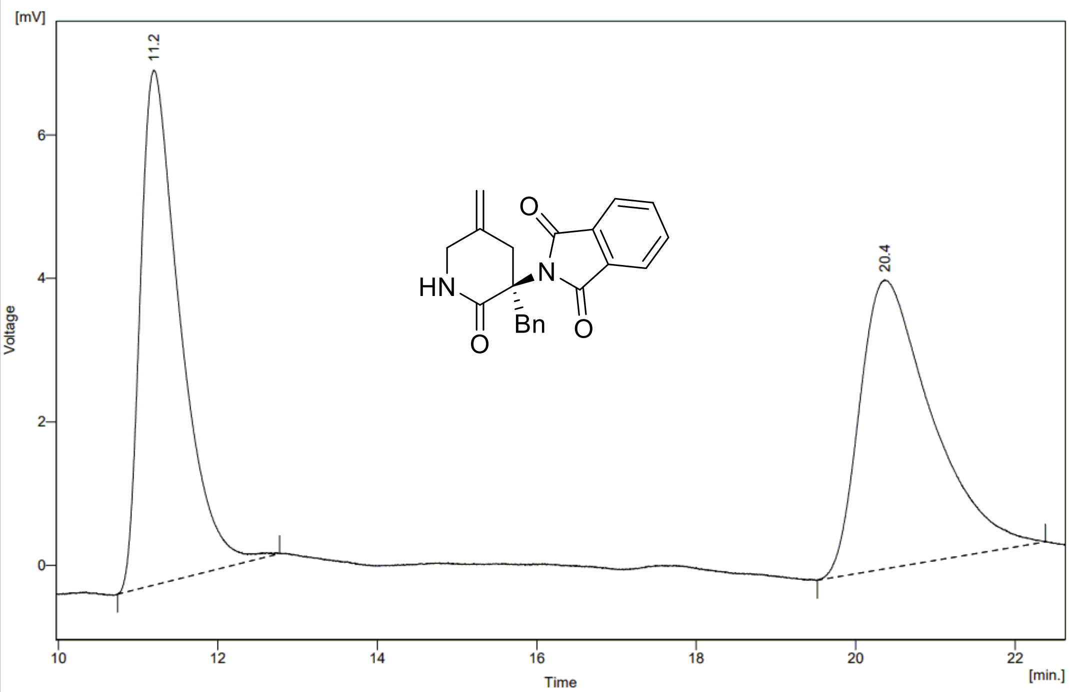


*Result Table - Calculation Method Uncal*

| | Reten. Time [min] | Area [mV.s] | Height [mV] | Area [%] | Height [%] | W05 [min] |
|---|---|---|---|---|---|---|
| 1 | 11.197 | 249.985 | 7.180 | 49.7 | 64.1 | 0.53 |
| 2 | 20.367 | 253.079 | 4.028 | 50.3 | 35.9 | 0.98 |
| | Total | 503.064 | 11.208 | 100.0 | 100.0 | |

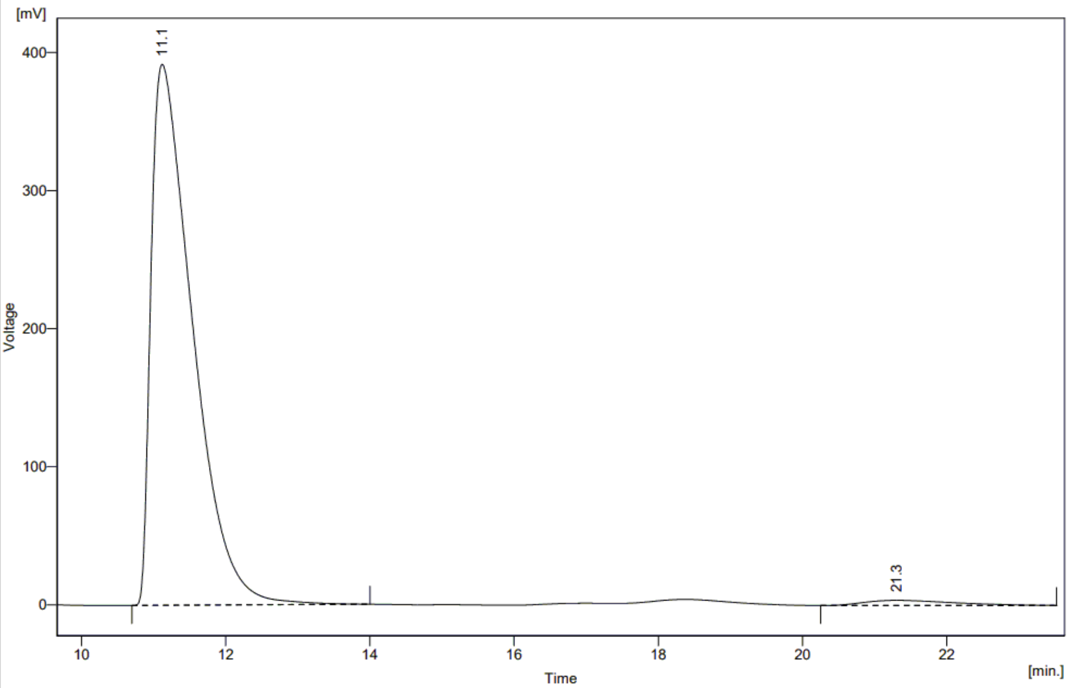


*Result Table - Calculation Method Uncal*

| | Reten. Time [min] | Area [mV.s] | Height [mV] | Area [%] | Height [%] | W05 [min] |
|---|---|---|---|---|---|---|
| 1 | 11.120 | 15651.256 | 391.826 | 98.0 | 99.1 | 0.62 |
| 2 | 21.303 | 325.742 | 3.758 | 2.0 | 0.9 | 1.38 |
| | Total | 15976.998 | 395.584 | 100.0 | 100.0 | |

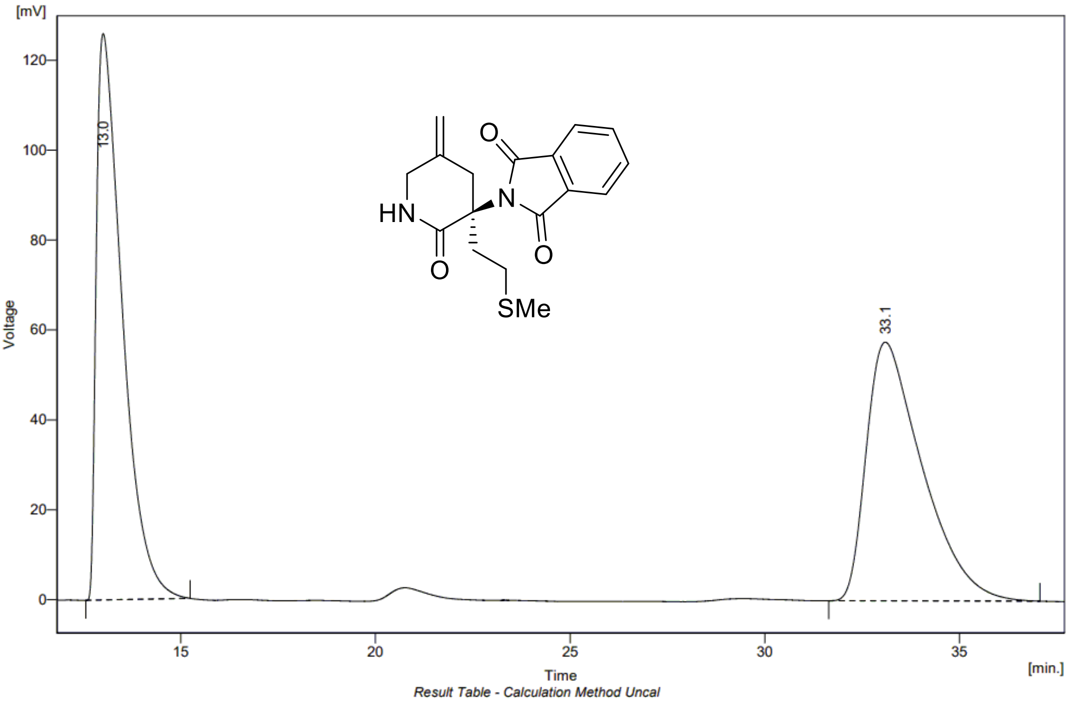


*Result Table - Calculation Method Uncal*

| | Reten. Time [min] | Area [mV.s] | Height [mV] | Area [%] | Height [%] | W05 [min] |
|---|---|---|---|---|---|---|
| 1 | 13.010 | 5835.593 | 126.056 | 50.3 | 68.6 | 0.71 |
| 2 | 33.100 | 5759.315 | 57.587 | 49.7 | 31.4 | 1.53 |
| | Total | 11594.909 | 183.642 | 100.0 | 100.0 | |

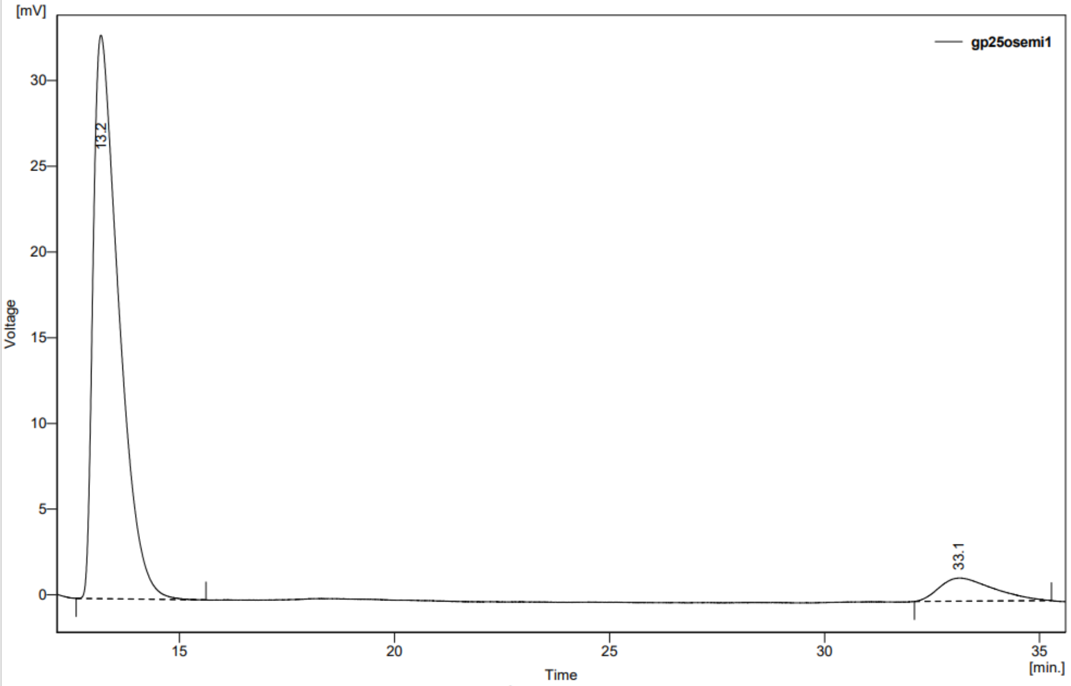


*Result Table - Calculation Method Uncal*

| | Reten. Time [min] | Area [mV.s] | Height [mV] | Area [%] | Height [%] | W05 [min] |
|---|---|---|---|---|---|---|
| 1 | 13.173 | 1362.148 | 32.868 | 92.0 | 96.0 | 0.64 |
| 2 | 33.117 | 118.925 | 1.357 | 8.0 | 4.0 | 1.40 |
| | Total | 1481.073 | 34.225 | 100.0 | 100.0 | |

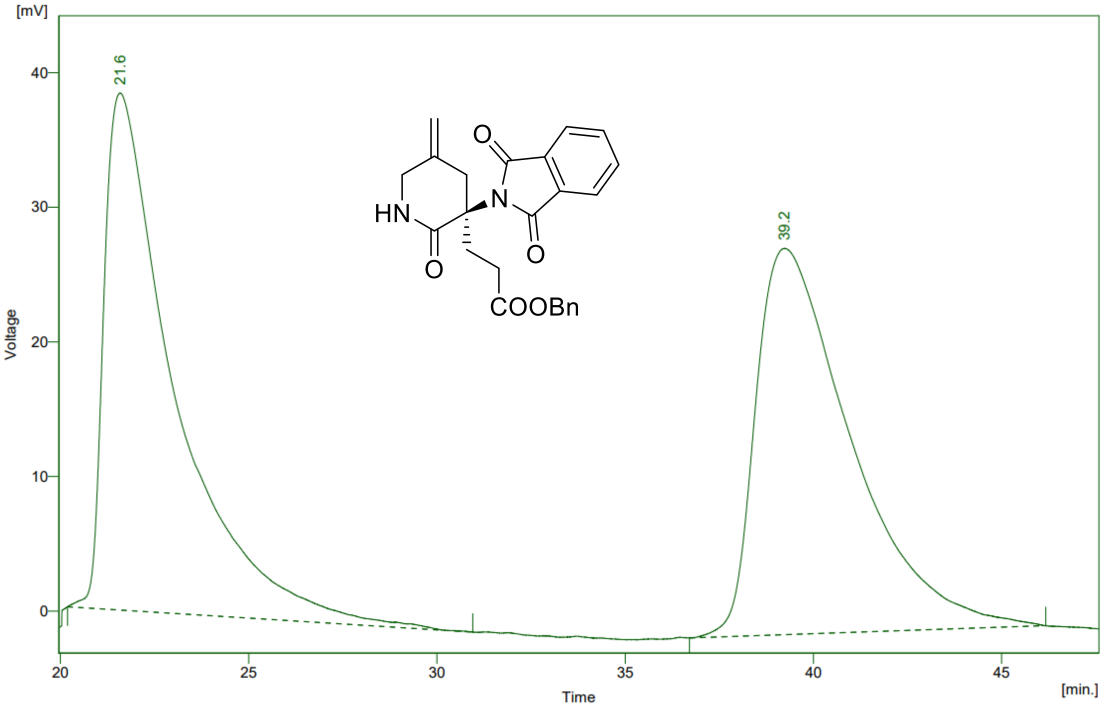


*Result Table - Calculation Method Uncal*

| | Reten. Time [min] | Area [mV.s] | Height [mV] | Area [%] | Height [%] | W05 [min] |
|---|---|---|---|---|---|---|
| 1 | 21.587 | 4991.681 | 38.437 | 49.7 | 57.3 | 1.69 |
| 2 | 39.223 | 5060.693 | 28.693 | 50.3 | 42.7 | 2.65 |
| | Total | 10052.373 | 67.130 | 100.0 | 100.0 | |

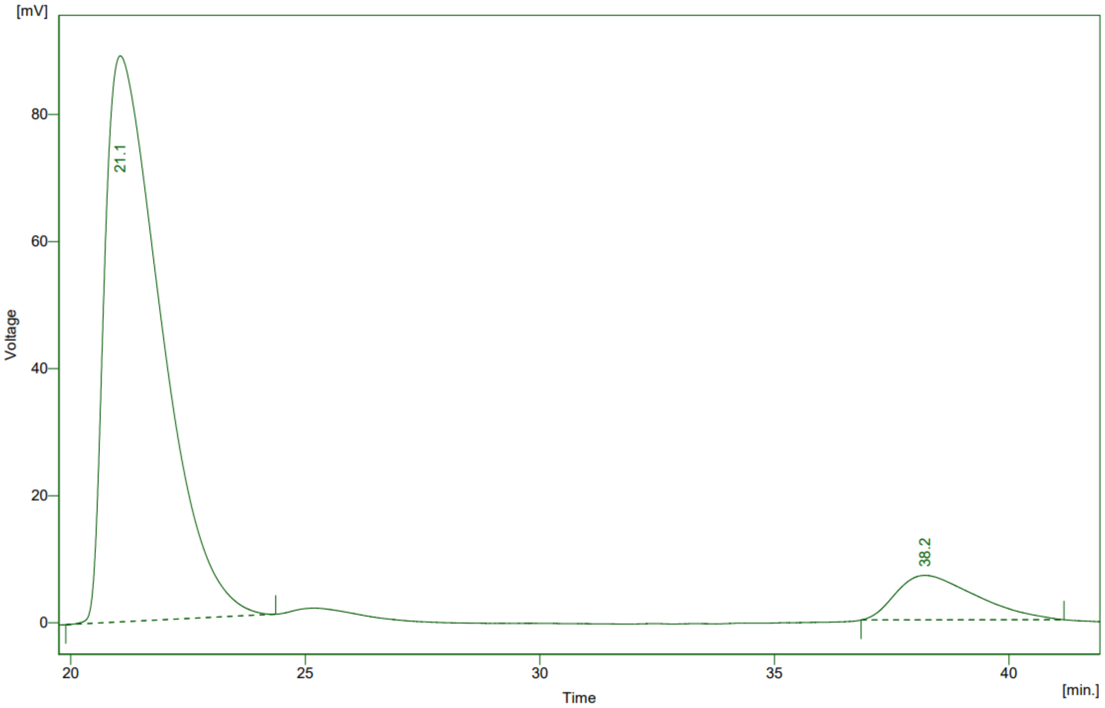


*Result Table - Calculation Method Uncal*

| | Reten. Time [min] | Area [mV.s] | Height [mV] | Area [%] | Height [%] | W05 [min] |
|---|---|---|---|---|---|---|
| 1 | 21.053 | 7458.846 | 89.130 | 90.0 | 92.7 | 1.31 |
| 2 | 38.207 | 833.335 | 7.012 | 10.0 | 7.3 | 1.90 |
| | Total | 8292.181 | 96.142 | 100.0 | 100.0 | |

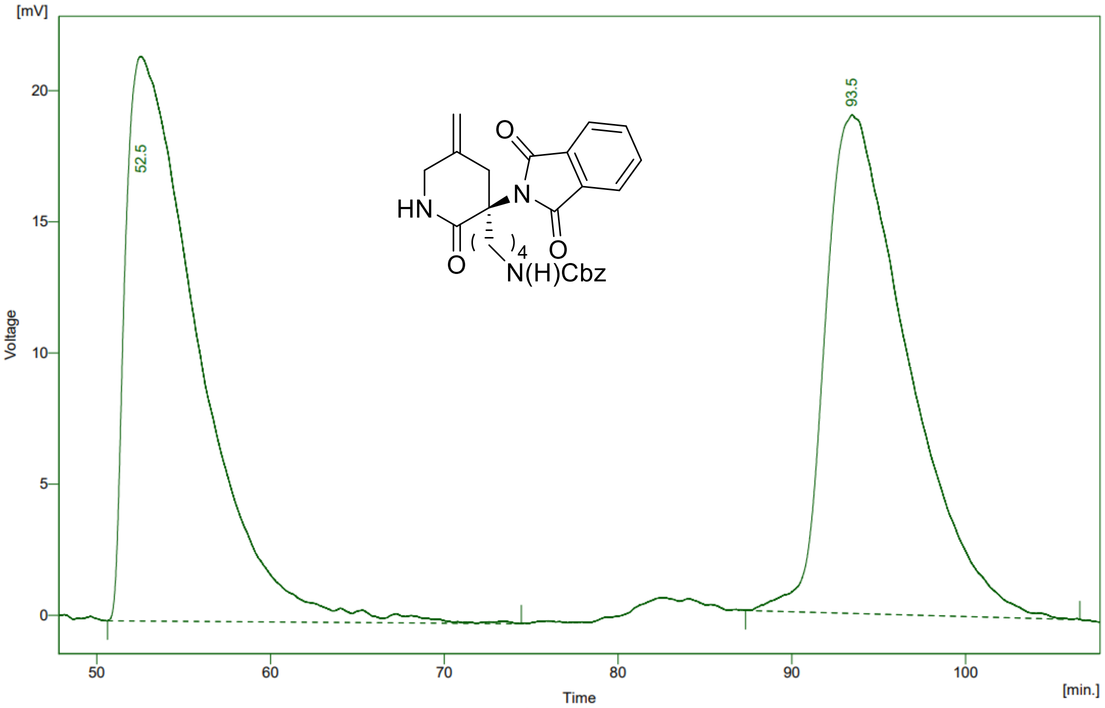


*Result Table - Calculation Method Uncal*

| | Reten. Time [min] | Area [mV.s] | Height [mV] | Area [%] | Height [%] | W05 [min] |
|---|---|---|---|---|---|---|
| 1 | 52.527 | 6282.501 | 21.533 | 50.0 | 53.1 | 4.28 |
| 2 | 93.450 | 6289.980 | 19.000 | 50.0 | 46.9 | 5.00 |
| | Total | 12572.481 | 40.533 | 100.0 | 100.0 | |

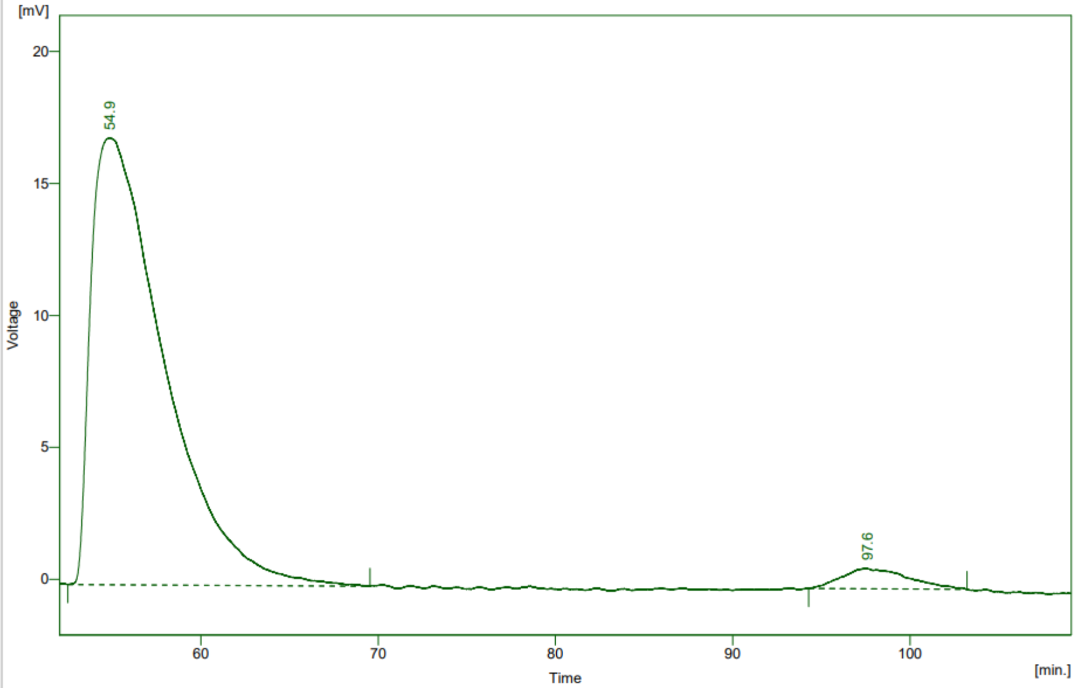


*Result Table - Calculation Method Uncal*

| | Reten. Time [min] | Area [mV.s] | Height [mV] | Area [%] | Height [%] | W05 [min] |
|---|---|---|---|---|---|---|
| 1 | 54.863 | 4866.038 | 16.940 | 96.0 | 95.7 | 4.30 |
| 2 | 97.607 | 202.465 | 0.764 | 4.0 | 4.3 | 4.14 |
| | Total | 5068.503 | 17.703 | 100.0 | 100.0 | |

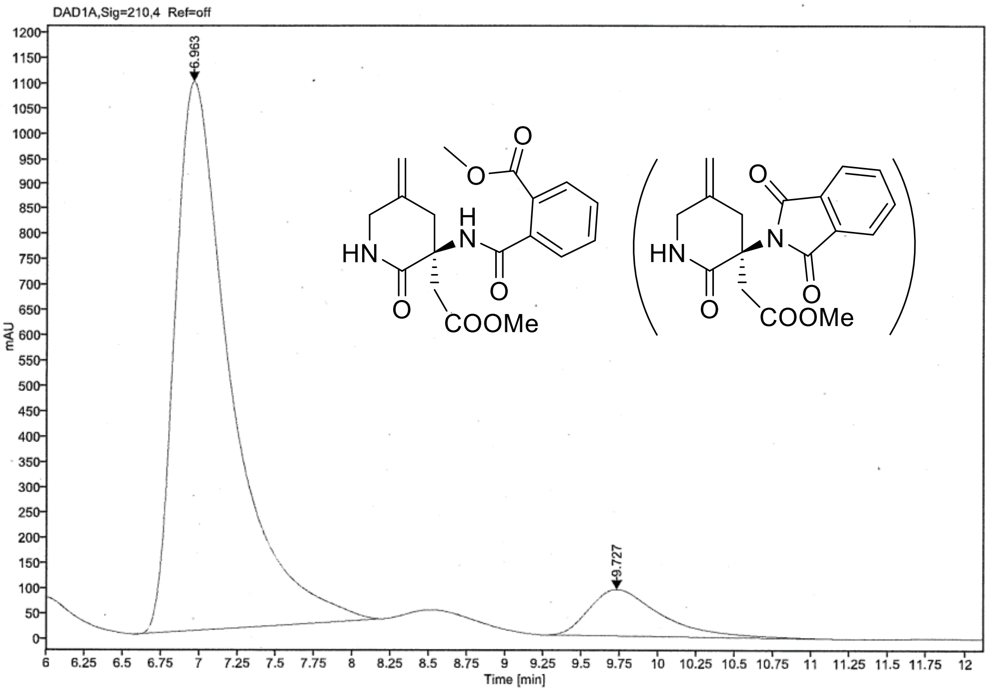


**Signal:** DAD1A,Sig=210,4 Ref=off

| RT [min] | Type | Width [min] | Area | Height | Area% | Name |
|---|---|---|---|---|---|---|
| 6.963 | MM m | 0.3719 | 27565.6416 | 1086.8924 | 90.1921 | |
| 9.727 | BB | 1.9854 | 2997.6160 | 92.0110 | 9.8079 | |
| | | Sum | 30563.2576 | | | |

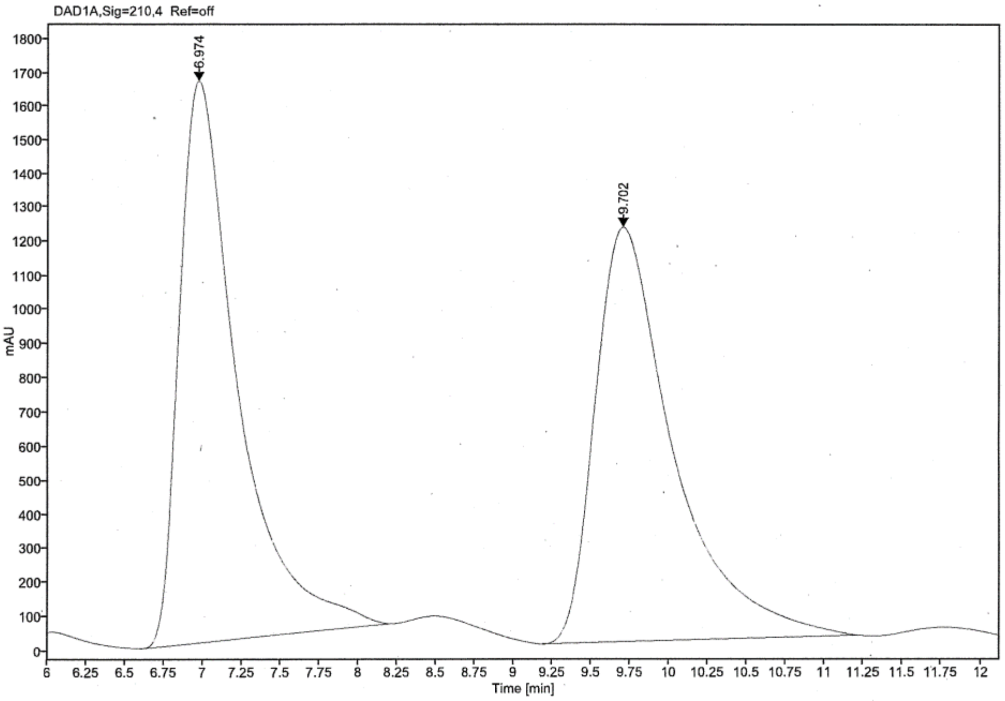


**Signal:** DAD1A,Sig=210,4 Ref=off

| RT [min] | Type | Width [min] | Area | Height | Area% | Name |
|---|---|---|---|---|---|---|
| 6.974 | VM m | 0.3837 | 42972.7625 | 1650.9311 | 50.2629 | |
| 9.702 | BM m | 0.5189 | 42523.2851 | 1213.2925 | 49.7371 | |
| | | Sum | 85496.0476 | | | |

**Details of the instrumentation used for crystal measurement studies**

Data for ojh419ncs_2022ncs0294 and 2022ncs0016z were collected at the National Crystallography Service, University of Southampton.[7]

For ojh419ncs_2022ncs0294, a suitable colourless needle crystal (0.19 × 0.025 × 0.02) mm was selected and mounted on a MiTeGen holder in perfluoro ether oil on a Rigaku 007HF diffractometer (Cu-Kα radiation) equipped with Arc)Sec VHF Varimax confocal mirrors and a UG2 goniometer and HyPix 6000HE detector and kept at 100(2) K during data collection.

In the case of 2022ncs0016z a suitable colourless needle-shaped crystal (0.21 × 0.02 × 0.01) mm was selected and mounted on a MiTeGen holder in perfluoro ether oil on a Rigaku 007HF diffractometer (Cu-Kα radiation) with HF Varimax confocal mirrors, an UG2 goniometer and HyPix 6000HE detector and kept at 100(2) K during data collection. The data were processed with CrysAlisPro.

Single crystal X-ray data for OJH420v_0m was collected in house on a Bruker D8 VENTURE diffractometer, equipped with a PHOTON 100 CMOS detector (Cu-Kα radiation). A suitable colourless plate-shaped crystal (0.5 x 0.098 x 0.06) mm was mounted in fomblin oil on a MiTiGen microloop and cooled in a stream of cold $N_2$ to 100 K. Data was corrected for absorption using empirical methods (SADABS)[8] based upon symmetry equivalent reflections combined with measurements at different azimuthal angles.

All crystal structures were solved and refined against $F^2$ values using ShelXT[9] for solution and ShelXL[10] for refinement accessed via the Olex2 program.[11] Non-H atoms were refined with anisotropic displacement parameters. Hydrogen atoms were added at calculated positions and refined with a riding model and isotropic displacement parameters fixed in magnitude relative to the attached carbon atoms.

**X-ray crystallographic analysis for compound 3 (OJH420v_0m)**

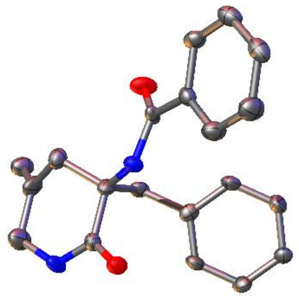

**Table 1 Crystal data and structure refinement for 3.**

| | |
|---|---|
| Identification code | OJH420v_0m |
| Empirical formula | $C_{20.25}H_{20.5}Cl_{0.5}N_2O_2$ |
| Formula weight | 341.61 |
| Temperature/K | 99.99 |
| Crystal system | monoclinic |
| Space group | C2 |
| a/Å | 13.6360(5) |
| b/Å | 13.6561(5) |
| c/Å | 20.1817(8) |
| α/° | 90 |
| β/° | 94.189(2) |
| γ/° | 90 |
| Volume/Å$^3$ | 3748.1(2) |
| Z | 8 |
| $\rho_{calc}$g/cm$^3$ | 1.211 |
| μ/mm$^{-1}$ | 1.261 |
| F(000) | 1444.0 |
| Crystal size/mm$^3$ | 0.5 × 0.098 × 0.06 |
| Radiation | CuKα (λ = 1.54178) |
| 2Θ range for data collection/° | 4.39 to 133.618 |
| Index ranges | $-14 \leq h \leq 16$, $-16 \leq k \leq 14$, $-24 \leq l \leq 23$ |
| Reflections collected | 27279 |
| Independent reflections | 6456 [$R_{int}$ = 0.0436, $R_{sigma}$ = 0.0360] |
| Data/restraints/parameters | 6456/3/463 |
| Goodness-of-fit on F$^2$ | 1.165 |
| Final R indexes [I>=2σ (I)] | $R_1$ = 0.0671, $wR_2$ = 0.1742 |
| Final R indexes [all data] | $R_1$ = 0.0682, $wR_2$ = 0.1750 |
| Largest diff. peak/hole / e Å$^{-3}$ | 0.49/-0.31 |
| Flack parameter | 0.095(14) |

**Table 2 Fractional Atomic Coordinates (×10[4]) and Equivalent Isotropic Displacement Parameters (Å[2]×10[3]) for 3. $U_{eq}$ is defined as 1/3 of the trace of the orthogonalised $U_{IJ}$ tensor.**

| Atom | *x* | *y* | *z* | U(eq) |
|---|---|---|---|---|
| O1 | 7828(3) | 5499(3) | 5854(2) | 30.8(9) |
| O2 | 8884(3) | 2187(3) | 5514(2) | 36.3(11) |
| N1 | 6990(4) | 5428(4) | 4857(3) | 35.3(13) |
| N2 | 8663(3) | 3792(3) | 5751(2) | 23.8(10) |
| C1 | 7552(4) | 5034(4) | 5359(3) | 26.8(13) |
| C2 | 7792(4) | 3937(4) | 5296(3) | 26.2(13) |
| C3 | 7997(5) | 3714(5) | 4571(3) | 31.5(14) |
| C4 | 7106(5) | 3975(5) | 4125(3) | 36.9(15) |
| C5 | 6790(6) | 5034(5) | 4181(3) | 43.8(18) |
| C6 | 9117(4) | 2917(4) | 5849(3) | 23.5(12) |
| C7 | 9928(4) | 2865(4) | 6390(3) | 23.9(12) |
| C8 | 10569(4) | 2066(4) | 6360(3) | 27.2(13) |
| C9 | 11329(4) | 1955(4) | 6848(3) | 32.0(14) |
| C10 | 11460(4) | 2617(5) | 7364(3) | 31.4(14) |
| C11 | 10829(5) | 3397(5) | 7393(3) | 38.6(15) |
| C12 | 10069(5) | 3535(5) | 6896(3) | 33.3(14) |
| C13 | 6914(4) | 3323(4) | 5495(3) | 27.7(13) |
| C14 | 6677(4) | 3412(4) | 6216(3) | 26.0(12) |
| C15 | 6094(4) | 4163(4) | 6421(3) | 28.7(13) |
| C16 | 5853(4) | 4225(4) | 7081(3) | 27.6(13) |
| C17 | 6217(4) | 3551(4) | 7539(3) | 25.0(12) |
| C18 | 6806(5) | 2788(5) | 7340(3) | 30.8(13) |
| C19 | 7035(5) | 2721(4) | 6683(3) | 32.7(14) |
| C20 | 6652(5) | 3359(5) | 3713(3) | 41.7(16) |
| O3 | 7179(3) | 7856(3) | 9224(2) | 25.6(9) |
| O4 | 6214(3) | 4471(3) | 9331(2) | 26.1(9) |
| N3 | 8116(4) | 7654(4) | 10185(3) | 26.3(11) |
| N4 | 6402(3) | 6110(3) | 9234(2) | 19.3(9) |
| C21 | 7518(4) | 7326(4) | 9676(3) | 20.3(11) |
| C22 | 7303(4) | 6217(4) | 9663(3) | 20.5(11) |
| C23 | 7183(4) | 5858(4) | 10379(3) | 21.0(11) |
| C24 | 8098(4) | 6110(5) | 10806(3) | 26.2(12) |
| C25 | 8343(5) | 7174(5) | 10826(3) | 33.7(14) |
| C26 | 5928(4) | 5266(4) | 9096(3) | 19.2(11) |
| C27 | 5031(4) | 5311(4) | 8625(3) | 18.4(10) |
| C28 | 4399(4) | 4512(4) | 8611(3) | 22.1(11) |
| C29 | 3557(4) | 4504(5) | 8180(3) | 31.0(14) |
| C30 | 3344(4) | 5278(5) | 7753(3) | 29.9(13) |
| C31 | 3969(4) | 6082(5) | 7768(3) | 31.9(14) |
| C32 | 4800(4) | 6101(4) | 8204(3) | 23.1(11) |
| C33 | 8178(4) | 5665(4) | 9392(3) | 20.7(11) |

**Table 2 Fractional Atomic Coordinates (×10$^4$) and Equivalent Isotropic Displacement Parameters (Å$^2$×10$^3$) for 3. $U_{eq}$ is defined as 1/3 of the trace of the orthogonalised $U_{IJ}$ tensor.**

| Atom | *x* | *y* | *z* | U(eq) |
|---|---|---|---|---|
| C34 | 8362(4) | 5846(4) | 8675(3) | 20.2(11) |
| C35 | 8896(4) | 6677(4) | 8490(3) | 23.9(12) |
| C36 | 9097(4) | 6808(4) | 7834(3) | 27.6(13) |
| C37 | 8777(4) | 6134(5) | 7353(3) | 28.1(13) |
| C38 | 8241(4) | 5326(5) | 7530(3) | 26.5(12) |
| C39 | 8051(4) | 5192(4) | 8189(3) | 22.6(11) |
| C40 | 8640(5) | 5437(5) | 11147(3) | 33.4(14) |
| Cl1 | 4490.6(12) | 8097.2(12) | 9333.6(9) | 41.2(4) |
| C41 | 5000 | 8768(8) | 10000 | 73(5) |

**Table 3 Anisotropic Displacement Parameters (Å$^2$×10$^3$) for 3. The Anisotropic displacement factor exponent takes the form: $-2\pi^2[h^2a^{*2}U_{11}+2hka^*b^*U_{12}+\ldots]$.**

| Atom | $U_{11}$ | $U_{22}$ | $U_{33}$ | $U_{23}$ | $U_{13}$ | $U_{12}$ |
|---|---|---|---|---|---|---|
| O1 | 42(2) | 24(2) | 26(2) | -4.0(17) | -1.9(18) | 5.7(18) |
| O2 | 38(2) | 25(2) | 43(3) | -15(2) | -10(2) | 4.0(19) |
| N1 | 58(4) | 22(3) | 25(3) | 0(2) | -6(2) | 11(2) |
| N2 | 28(2) | 17(2) | 26(2) | -2.0(18) | -2(2) | 2.0(18) |
| C1 | 34(3) | 25(3) | 22(3) | 3(2) | 3(2) | 3(2) |
| C2 | 24(3) | 31(3) | 23(3) | 0(2) | 1(2) | 9(2) |
| C3 | 33(3) | 38(4) | 23(3) | -1(2) | 1(2) | 1(3) |
| C4 | 47(4) | 44(4) | 19(3) | -10(3) | -1(3) | 8(3) |
| C5 | 62(5) | 39(4) | 28(3) | -4(3) | -11(3) | 11(3) |
| C6 | 39(3) | 16(3) | 15(3) | -1(2) | -3(2) | 0(2) |
| C7 | 25(3) | 23(3) | 24(3) | 5(2) | 2(2) | 1(2) |
| C8 | 31(3) | 19(3) | 32(3) | -5(2) | 5(2) | 0(2) |
| C9 | 28(3) | 24(3) | 44(4) | 7(3) | 3(3) | 2(2) |
| C10 | 25(3) | 32(3) | 37(4) | 12(3) | -1(3) | -2(2) |
| C11 | 45(4) | 36(4) | 34(3) | -9(3) | -4(3) | 4(3) |
| C12 | 36(3) | 27(3) | 36(3) | -6(3) | 0(3) | 7(3) |
| C13 | 38(3) | 19(3) | 26(3) | -9(2) | -5(2) | 3(2) |
| C14 | 22(3) | 29(3) | 27(3) | -7(2) | 0(2) | -3(2) |
| C15 | 36(3) | 18(3) | 31(3) | 1(2) | -4(2) | 8(2) |
| C16 | 27(3) | 25(3) | 32(3) | -1(2) | 9(2) | 3(2) |
| C17 | 31(3) | 18(3) | 26(3) | -4(2) | 2(2) | -2(2) |
| C18 | 37(3) | 27(3) | 28(3) | 5(2) | 1(3) | 6(2) |
| C19 | 44(4) | 21(3) | 32(3) | -1(2) | 0(3) | 13(3) |
| C20 | 51(4) | 40(4) | 33(3) | -8(3) | -6(3) | 12(3) |
| O3 | 26(2) | 16(2) | 34(2) | 4.4(16) | -0.2(17) | -1.1(15) |
| O4 | 28(2) | 8.4(18) | 40(2) | 2.2(16) | -9.2(17) | -1.3(15) |
| N3 | 33(3) | 13(3) | 32(3) | -7(2) | -2(2) | -4(2) |

**Table 3 Anisotropic Displacement Parameters (Å$^2$×10$^3$) for 3. The Anisotropic displacement factor exponent takes the form: $-2\pi^2[h^2a^{*2}U_{11}+2hka^*b^*U_{12}+\ldots]$.**

| Atom | $U_{11}$ | $U_{22}$ | $U_{33}$ | $U_{23}$ | $U_{13}$ | $U_{12}$ |
|---|---|---|---|---|---|---|
| N4 | 22(2) | 9(2) | 26(2) | -2.5(17) | -1.9(18) | 0.9(17) |
| C21 | 19(3) | 12(3) | 30(3) | -3(2) | 2(2) | 0(2) |
| C22 | 27(3) | 11(3) | 23(3) | 1(2) | -3(2) | 3(2) |
| C23 | 25(3) | 14(3) | 25(3) | -3(2) | 2(2) | -1(2) |
| C24 | 32(3) | 29(3) | 18(3) | -2(2) | 0(2) | -2(2) |
| C25 | 36(3) | 35(4) | 30(3) | -12(3) | -4(3) | -7(3) |
| C26 | 21(3) | 12(3) | 25(3) | 1(2) | 4(2) | 4(2) |
| C27 | 17(2) | 18(3) | 20(2) | -4(2) | 3(2) | 3(2) |
| C28 | 22(3) | 9(2) | 36(3) | 1(2) | 4(2) | 0(2) |
| C29 | 21(3) | 33(3) | 39(3) | -10(3) | 3(2) | -3(2) |
| C30 | 23(3) | 39(4) | 28(3) | -5(3) | -1(2) | 1(3) |
| C31 | 27(3) | 37(3) | 32(3) | 13(3) | 3(2) | 8(3) |
| C32 | 24(3) | 23(3) | 22(3) | -6(2) | 4(2) | 2(2) |
| C33 | 19(3) | 14(3) | 29(3) | -1(2) | -3(2) | -0.1(19) |
| C34 | 19(3) | 19(3) | 22(3) | 0(2) | -1(2) | 8(2) |
| C35 | 15(2) | 19(3) | 38(3) | -3(2) | 1(2) | -2(2) |
| C36 | 25(3) | 24(3) | 35(3) | 7(2) | 8(2) | 0(2) |
| C37 | 23(3) | 35(3) | 26(3) | 6(2) | 3(2) | 8(2) |
| C38 | 25(3) | 33(3) | 21(3) | -3(2) | -1(2) | 3(2) |
| C39 | 23(3) | 19(3) | 26(3) | 2(2) | 0(2) | 1(2) |
| C40 | 32(3) | 37(4) | 30(3) | 7(3) | -7(3) | -9(3) |
| Cl1 | 36.0(8) | 36.3(9) | 50.9(9) | -10.4(7) | 1.6(7) | -0.6(7) |
| C41 | 152(14) | 20(5) | 39(6) | 0 | -44(7) | 0 |

**Table 4 Bond Lengths for <u>3</u>.**

| Atom | Atom | Length/Å | Atom | Atom | Length/Å |
|---|---|---|---|---|---|
| O1 | C1 | 1.220(7) | O4 | C26 | 1.236(7) |
| O2 | C6 | 1.233(7) | N3 | C21 | 1.340(8) |
| N1 | C1 | 1.338(8) | N3 | C25 | 1.463(9) |
| N1 | C5 | 1.473(8) | N4 | C22 | 1.457(7) |
| N2 | C2 | 1.460(7) | N4 | C26 | 1.341(7) |
| N2 | C6 | 1.353(7) | C21 | C22 | 1.542(7) |
| C1 | C2 | 1.541(8) | C22 | C23 | 1.547(7) |
| C2 | C3 | 1.540(8) | C22 | C33 | 1.545(7) |
| C2 | C13 | 1.538(9) | C23 | C24 | 1.503(8) |
| C3 | C4 | 1.502(9) | C24 | C25 | 1.490(9) |
| C4 | C5 | 1.515(10) | C24 | C40 | 1.339(9) |
| C4 | C20 | 1.306(10) | C26 | C27 | 1.495(7) |
| C6 | C7 | 1.498(7) | C27 | C28 | 1.389(7) |
| C7 | C8 | 1.402(8) | C27 | C32 | 1.394(8) |

**Table 4 Bond Lengths for 3.**

| Atom | Atom | Length/Å | Atom | Atom | Length/Å |
|---|---|---|---|---|---|
| C7 | C12 | 1.376(8) | C28 | C29 | 1.390(8) |
| C8 | C9 | 1.386(9) | C29 | C30 | 1.382(9) |
| C9 | C10 | 1.380(10) | C30 | C31 | 1.389(9) |
| C10 | C11 | 1.373(9) | C31 | C32 | 1.383(8) |
| C11 | C12 | 1.400(9) | C33 | C34 | 1.508(8) |
| C13 | C14 | 1.518(8) | C34 | C35 | 1.413(8) |
| C14 | C15 | 1.379(8) | C34 | C39 | 1.371(8) |
| C14 | C19 | 1.396(8) | C35 | C36 | 1.384(9) |
| C15 | C16 | 1.397(8) | C36 | C37 | 1.385(9) |
| C16 | C17 | 1.372(8) | C37 | C38 | 1.385(9) |
| C17 | C18 | 1.392(8) | C38 | C39 | 1.386(8) |
| C18 | C19 | 1.389(9) | Cl1 | C41 | 1.730(6) |
| O3 | C21 | 1.228(7) | | | |

**Table 5 Bond Angles for 3.**

| Atom | Atom | Atom | Angle/° | Atom | Atom | Atom | Angle/° |
|---|---|---|---|---|---|---|---|
| C1 | N1 | C5 | 127.6(6) | C26 | N4 | C22 | 125.6(4) |
| C6 | N2 | C2 | 123.6(5) | O3 | C21 | N3 | 123.2(5) |
| O1 | C1 | N1 | 122.4(6) | O3 | C21 | C22 | 120.3(5) |
| O1 | C1 | C2 | 121.3(5) | N3 | C21 | C22 | 116.5(5) |
| N1 | C1 | C2 | 116.3(5) | N4 | C22 | C21 | 105.1(4) |
| N2 | C2 | C1 | 104.3(5) | N4 | C22 | C23 | 112.4(4) |
| N2 | C2 | C3 | 112.0(5) | N4 | C22 | C33 | 112.4(4) |
| N2 | C2 | C13 | 112.1(5) | C21 | C22 | C23 | 109.1(4) |
| C3 | C2 | C1 | 108.9(5) | C21 | C22 | C33 | 109.5(4) |
| C13 | C2 | C1 | 109.5(5) | C33 | C22 | C23 | 108.2(4) |
| C13 | C2 | C3 | 109.8(5) | C24 | C23 | C22 | 108.8(4) |
| C4 | C3 | C2 | 109.2(5) | C25 | C24 | C23 | 114.4(5) |
| C3 | C4 | C5 | 113.8(6) | C40 | C24 | C23 | 122.8(6) |
| C20 | C4 | C3 | 123.7(6) | C40 | C24 | C25 | 122.8(5) |
| C20 | C4 | C5 | 122.6(6) | N3 | C25 | C24 | 112.3(5) |
| N1 | C5 | C4 | 112.6(5) | O4 | C26 | N4 | 122.8(5) |
| O2 | C6 | N2 | 122.5(5) | O4 | C26 | C27 | 120.1(5) |
| O2 | C6 | C7 | 120.5(5) | N4 | C26 | C27 | 117.1(4) |
| N2 | C6 | C7 | 117.0(5) | C28 | C27 | C26 | 117.2(5) |
| C8 | C7 | C6 | 115.8(5) | C28 | C27 | C32 | 118.9(5) |
| C12 | C7 | C6 | 124.5(5) | C32 | C27 | C26 | 123.9(5) |
| C12 | C7 | C8 | 119.7(5) | C27 | C28 | C29 | 120.1(5) |
| C9 | C8 | C7 | 119.4(6) | C30 | C29 | C28 | 120.7(6) |
| C10 | C9 | C8 | 120.9(6) | C29 | C30 | C31 | 119.4(5) |
| C11 | C10 | C9 | 119.5(6) | C32 | C31 | C30 | 120.0(6) |

**Table 5 Bond Angles for 3.**

| Atom | Atom | Atom | Angle/° | Atom | Atom | Atom | Angle/° |
|---|---|---|---|---|---|---|---|
| C10 | C11 | C12 | 120.5(6) | C31 | C32 | C27 | 120.8(5) |
| C7 | C12 | C11 | 119.9(6) | C34 | C33 | C22 | 116.5(4) |
| C14 | C13 | C2 | 115.5(5) | C35 | C34 | C33 | 120.7(5) |
| C15 | C14 | C13 | 121.0(5) | C39 | C34 | C33 | 121.0(5) |
| C15 | C14 | C19 | 118.6(5) | C39 | C34 | C35 | 118.3(5) |
| C19 | C14 | C13 | 120.3(5) | C36 | C35 | C34 | 119.9(5) |
| C14 | C15 | C16 | 120.9(6) | C35 | C36 | C37 | 120.6(5) |
| C17 | C16 | C15 | 120.1(5) | C38 | C37 | C36 | 119.6(5) |
| C16 | C17 | C18 | 119.8(5) | C37 | C38 | C39 | 119.6(6) |
| C19 | C18 | C17 | 119.9(5) | C34 | C39 | C38 | 122.0(5) |
| C18 | C19 | C14 | 120.6(5) | Cl1[1] | C41 | Cl1 | 116.1(6) |
| C21 | N3 | C25 | 127.2(5) | | | | |

[1]1-X,+Y,2-Z

**Table 6 Hydrogen Bonds for 3.**

| D | H | A | d(D-H)/Å | d(H-A)/Å | d(D-A)/Å | D-H-A/° |
|---|---|---|---|---|---|---|
| N1 | H1 | O2[1] | 0.81(7) | 2.07(7) | 2.760(7) | 142(6) |
| N3 | H3 | O4[2] | 0.85(9) | 2.04(8) | 2.796(6) | 148(7) |

[1]3/2-X,1/2+Y,1-Z; [2]3/2-X,1/2+Y,2-Z

**Table 7 Torsion Angles for 3.**

| A | B | C | D | Angle/° | A | B | C | D | Angle/° |
|---|---|---|---|---|---|---|---|---|---|
| O1 | C1 | C2 | N2 | -23.3(7) | O3 | C21 | C22 | N4 | -22.8(7) |
| O1 | C1 | C2 | C3 | -143.1(6) | O3 | C21 | C22 | C23 | -143.6(5) |
| O1 | C1 | C2 | C13 | 96.8(7) | O3 | C21 | C22 | C33 | 98.1(6) |
| O2 | C6 | C7 | C8 | 18.0(8) | O4 | C26 | C27 | C28 | 17.6(7) |
| O2 | C6 | C7 | C12 | -161.8(6) | O4 | C26 | C27 | C32 | -162.2(5) |
| N1 | C1 | C2 | N2 | 159.8(5) | N3 | C21 | C22 | N4 | 159.9(5) |
| N1 | C1 | C2 | C3 | 40.0(7) | N3 | C21 | C22 | C23 | 39.1(6) |
| N1 | C1 | C2 | C13 | -80.1(7) | N3 | C21 | C22 | C33 | -79.2(6) |
| N2 | C2 | C3 | C4 | -174.3(5) | N4 | C22 | C23 | C24 | -173.0(4) |
| N2 | C2 | C13 | C14 | 50.4(7) | N4 | C22 | C33 | C34 | 50.0(6) |
| N2 | C6 | C7 | C8 | -162.0(5) | N4 | C26 | C27 | C28 | -163.7(5) |
| N2 | C6 | C7 | C12 | 18.2(8) | N4 | C26 | C27 | C32 | 16.5(7) |
| C1 | N1 | C5 | C4 | 13.2(11) | C21 | N3 | C25 | C24 | 18.4(9) |
| C1 | C2 | C3 | C4 | -59.4(7) | C21 | C22 | C23 | C24 | -56.8(6) |
| C1 | C2 | C13 | C14 | -64.9(6) | C21 | C22 | C33 | C34 | -66.4(6) |
| C2 | N2 | C6 | O2 | 7.0(9) | C22 | N4 | C26 | O4 | 0.6(8) |

**Table 7 Torsion Angles for 3.**

| A | B | C | D | Angle/° | A | B | C | D | Angle/° |
|---|---|---|---|---|---|---|---|---|---|
| C2 | N2 | C6 | C7 | -173.0(5) | C22 | N4 | C26 | C27 | -178.1(5) |
| C2 | C3 | C4 | C5 | 57.6(8) | C22 | C23 | C24 | C25 | 58.1(6) |
| C2 | C3 | C4 | C20 | -123.9(7) | C22 | C23 | C24 | C40 | -122.9(6) |
| C2 | C13 | C14 | C15 | 84.7(7) | C22 | C33 | C34 | C35 | 82.6(6) |
| C2 | C13 | C14 | C19 | -96.3(7) | C22 | C33 | C34 | C39 | -100.0(6) |
| C3 | C2 | C13 | C14 | 175.6(5) | C23 | C22 | C33 | C34 | 174.7(4) |
| C3 | C4 | C5 | N1 | -32.8(9) | C23 | C24 | C25 | N3 | -36.9(7) |
| C5 | N1 | C1 | O1 | 165.2(7) | C25 | N3 | C21 | O3 | 162.0(6) |
| C5 | N1 | C1 | C2 | -17.9(10) | C25 | N3 | C21 | C22 | -20.8(8) |
| C6 | N2 | C2 | C1 | 179.7(5) | C26 | N4 | C22 | C21 | -178.0(5) |
| C6 | N2 | C2 | C3 | -62.6(7) | C26 | N4 | C22 | C23 | -59.4(7) |
| C6 | N2 | C2 | C13 | 61.3(7) | C26 | N4 | C22 | C33 | 63.0(7) |
| C6 | C7 | C8 | C9 | -178.9(5) | C26 | C27 | C28 | C29 | -179.4(5) |
| C6 | C7 | C12 | C11 | 177.5(6) | C26 | C27 | C32 | C31 | 178.2(5) |
| C7 | C8 | C9 | C10 | 0.3(9) | C27 | C28 | C29 | C30 | 1.1(9) |
| C8 | C7 | C12 | C11 | -2.3(9) | C28 | C27 | C32 | C31 | -1.6(8) |
| C8 | C9 | C10 | C11 | -0.2(10) | C28 | C29 | C30 | C31 | -1.5(9) |
| C9 | C10 | C11 | C12 | -1.2(10) | C29 | C30 | C31 | C32 | 0.3(9) |
| C10 | C11 | C12 | C7 | 2.5(10) | C30 | C31 | C32 | C27 | 1.2(9) |
| C12 | C7 | C8 | C9 | 0.9(9) | C32 | C27 | C28 | C29 | 0.4(8) |
| C13 | C2 | C3 | C4 | 60.5(7) | C33 | C22 | C23 | C24 | 62.3(6) |
| C13 | C14 | C15 | C16 | 178.0(5) | C33 | C34 | C35 | C36 | 177.0(5) |
| C13 | C14 | C19 | C18 | -178.9(6) | C33 | C34 | C39 | C38 | -177.7(5) |
| C14 | C15 | C16 | C17 | 1.8(9) | C34 | C35 | C36 | C37 | 0.2(8) |
| C15 | C14 | C19 | C18 | 0.2(9) | C35 | C34 | C39 | C38 | -0.3(8) |
| C15 | C16 | C17 | C18 | -1.8(9) | C35 | C36 | C37 | C38 | 0.7(8) |
| C16 | C17 | C18 | C19 | 1.0(9) | C36 | C37 | C38 | C39 | -1.4(8) |
| C17 | C18 | C19 | C14 | -0.2(10) | C37 | C38 | C39 | C34 | 1.2(8) |
| C19 | C14 | C15 | C16 | -1.0(9) | C39 | C34 | C35 | C36 | -0.5(8) |
| C20 | C4 | C5 | N1 | 148.7(7) | C40 | C24 | C25 | N3 | 144.2(6) |

**Table 8 Hydrogen Atom Coordinates (Å×10$^4$) and Isotropic Displacement Parameters (Å$^2$×10$^3$) for 3.**

| Atom | *x* | *y* | *z* | U(eq) |
|---|---|---|---|---|
| H1 | 6850(50) | 5990(60) | 4920(30) | 26(18) |
| H2 | 8850(30) | 4370(20) | 6030(20) | 0(11) |
| H3A | 8569.51 | 4099.83 | 4445.48 | 38 |
| H3B | 8151.26 | 3010.65 | 4522.75 | 38 |
| H5A | 7142.32 | 5435.17 | 3865.85 | 53 |
| H5B | 6077.35 | 5085.27 | 4053.2 | 53 |
| H8 | 10483.34 | 1605.3 | 6008.16 | 33 |

**Table 8 Hydrogen Atom Coordinates (Å×10[4]) and Isotropic Displacement Parameters (Å[2]×10[3]) for 3.**

| Atom | *x* | *y* | *z* | U(eq) |
|---|---|---|---|---|
| H9 | 11766.9 | 1416.32 | 6827.9 | 38 |
| H10 | 11981.92 | 2532.84 | 7697.66 | 38 |
| H11 | 10908.53 | 3846.81 | 7751.78 | 46 |
| H12 | 9651.83 | 4090.91 | 6909.57 | 40 |
| H13A | 6325.4 | 3518.69 | 5209.29 | 33 |
| H13B | 7047.9 | 2626.58 | 5400.43 | 33 |
| H15 | 5854.61 | 4643.58 | 6110.38 | 34 |
| H16 | 5435.25 | 4735.75 | 7212.16 | 33 |
| H17 | 6067.02 | 3604.25 | 7989.69 | 30 |
| H18 | 7051.26 | 2313.94 | 7654.72 | 37 |
| H19 | 7438.61 | 2201.06 | 6549.25 | 39 |
| H20A | 6882.71 | 2705.29 | 3684.71 | 50 |
| H20B | 6090.27 | 3564.96 | 3442.6 | 50 |
| H3 | 8140(50) | 8270(70) | 10220(40) | 40(20) |
| H4 | 6140(40) | 6710(30) | 9010(30) | 19(15) |
| H23A | 6605.59 | 6176.78 | 10557.47 | 25 |
| H23B | 7076.95 | 5140.78 | 10380.14 | 25 |
| H25A | 7967.69 | 7496.73 | 11166.8 | 40 |
| H25B | 9051.57 | 7254.39 | 10958.5 | 40 |
| H28 | 4543.94 | 3970.23 | 8897.12 | 27 |
| H29 | 3122.44 | 3959.47 | 8179.1 | 37 |
| H30 | 2774.95 | 5260.93 | 7452.23 | 36 |
| H31 | 3826.78 | 6619.34 | 7477.33 | 38 |
| H32 | 5217.52 | 6658.1 | 8217.31 | 28 |
| H33A | 8781.4 | 5844.22 | 9668.86 | 25 |
| H33B | 8071.61 | 4954.52 | 9451 | 25 |
| H35 | 9116.43 | 7144.58 | 8816.18 | 29 |
| H36 | 9458.84 | 7366.89 | 7712.14 | 33 |
| H37 | 8924.57 | 6225.16 | 6904.24 | 34 |
| H38 | 8005.8 | 4867.24 | 7202.6 | 32 |
| H39 | 7693.71 | 4628.78 | 8308.03 | 27 |
| H40A | 9207.51 | 5630.06 | 11417.22 | 40 |
| H40B | 8458 | 4765.75 | 11120.2 | 40 |
| H41A | 5517.18 | 9197.45 | 9838.95 | 87 |
| H41B | 4482.8 | 9197.43 | 10161.06 | 87 |

**Table 9 Atomic Occupancy for 3**

| Atom | *Occupancy* | Atom | *Occupancy* | Atom | *Occupancy* |
|---|---|---|---|---|---|
| H41A | 0.5 | H41B | 0.5 | | |

**Table 10 Solvent masks information for 3.**

| Number | X | Y | Z | Volume | Electron count | Content |
|---|---|---|---|---|---|---|
| 1 | 0.000 | 0.012 | 0.500 | 15.1 | 0.0 | |
| 2 | 0.000 | 0.605 | 0.500 | 151.9 | 54.1 | 1 hexane |
| 3 | 0.000 | 0.694 | 0.000 | 7.2 | 0.0 | |
| 4 | 0.500 | 0.105 | 0.500 | 151.9 | 54.0 | 1 hexane |
| 5 | 0.500 | 0.194 | 0.000 | 7.2 | 0.0 | |
| 6 | 0.500 | 0.512 | 0.500 | 15.1 | 0.0 | |

**Crystal structure determination of 3**

**Crystal Data** for $C_{20.25}H_{20.5}Cl_{0.5}N_2O_2$ ($M$ =341.61 g/mol): monoclinic, space group C2 (no. 5), $a$ = 13.6360(5) Å, $b$ = 13.6561(5) Å, $c$ = 20.1817(8) Å, $\beta$ = 94.189(2)°, $V$ = 3748.1(2) Å$^3$, $Z$ = 8, $T$ = 99.99 K, μ(CuKα) = 1.261 mm$^{-1}$, $Dcalc$ = 1.211 g/cm$^3$, 27279 reflections measured (4.39° ≤ 2Θ ≤ 133.618°), 6456 unique ($R_{int}$ = 0.0436, $R_{sigma}$ = 0.0360) which were used in all calculations. The final $R_1$ was 0.0671 (I > 2σ(I)) and $wR_2$ was 0.1750 (all data).

**X-ray crystallographic analysis for compound 5 (2022ncs0016z)**

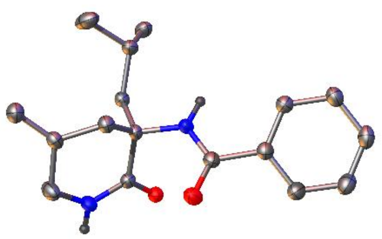

**Table 1 Crystal data and structure refinement for 5.**

| | |
|---|---|
| Identification code | 2022ncs0016z |
| Empirical formula | $C_{17}H_{22}N_2O_2$ |
| Formula weight | 286.36 |
| Temperature/K | 100(2) |
| Crystal system | orthorhombic |
| Space group | $P2_12_12_1$ |
| a/Å | 8.04670(10) |
| b/Å | 11.0689(2) |
| c/Å | 17.3352(4) |
| α/° | 90 |
| β/° | 90 |
| γ/° | 90 |
| Volume/Å$^3$ | 1544.01(5) |

| | |
|---|---|
| Z | 4 |
| $\rho_{calc}$g/cm$^3$ | 1.232 |
| μ/mm$^{-1}$ | 0.647 |
| F(000) | 616.0 |
| Crystal size/mm$^3$ | 0.21 × 0.02 × 0.01 |
| Radiation | Cu Kα (λ = 1.54178) |
| 2Θ range for data collection/° | 9.48 to 153.196 |
| Index ranges | -6 ≤ h ≤ 9, -13 ≤ k ≤ 13, -21 ≤ l ≤ 21 |
| Reflections collected | 22416 |
| Independent reflections | 3130 [$R_{int}$ = 0.0514, $R_{sigma}$ = 0.0263] |
| Data/restraints/parameters | 3130/0/192 |
| Goodness-of-fit on F$^2$ | 1.077 |
| Final R indexes [I>=2σ (I)] | $R_1$ = 0.0407, w$R_2$ = 0.0960 |
| Final R indexes [all data] | $R_1$ = 0.0471, w$R_2$ = 0.1009 |
| Largest diff. peak/hole / e Å$^{-3}$ | 0.25/-0.24 |
| Flack parameter | 0.21(11) |

**Table 2 Fractional Atomic Coordinates (×10$^4$) and Equivalent Isotropic Displacement Parameters (Å$^2$×10$^3$) for 5. $U_{eq}$ is defined as 1/3 of the trace of the orthogonalised $U_{IJ}$ tensor.**

| Atom | *x* | *y* | *z* | U(eq) |
|---|---|---|---|---|
| O1 | 5347(2) | 7635.3(15) | 5011.0(12) | 26.4(4) |
| O2 | 4763(2) | 8089.0(17) | 6742.9(11) | 30.9(4) |
| N1 | 7249(3) | 6401(2) | 5544.7(13) | 27.9(5) |
| N2 | 2977(3) | 6903.7(19) | 6062.9(13) | 25.1(5) |
| C1 | 5696(3) | 6777(2) | 5450.3(16) | 25.0(5) |
| C2 | 4277(3) | 6055(2) | 5826.0(15) | 23.6(5) |
| C3 | 4880(3) | 5326(2) | 6526.9(16) | 27.5(6) |
| C4 | 6435(3) | 4643(2) | 6335.6(16) | 26.7(6) |
| C5 | 7812(3) | 5419(3) | 6046.7(19) | 36.8(7) |
| C6 | 3357(3) | 7888(2) | 6496.4(16) | 25.6(5) |
| C7 | 1927(3) | 8722(2) | 6661.4(15) | 26.4(5) |
| C8 | 2253(4) | 9953(2) | 6753.0(16) | 30.9(6) |
| C9 | 968(4) | 10739(3) | 6931.1(18) | 34.9(6) |
| C10 | -635(4) | 10312(3) | 7034.2(17) | 34.6(7) |
| C11 | -968(4) | 9084(3) | 6947.6(17) | 31.8(6) |
| C12 | 317(3) | 8306(2) | 6757.8(16) | 28.4(6) |
| C13 | 3596(3) | 5260(2) | 5165.9(16) | 25.7(6) |
| C14 | 2028(3) | 4497(2) | 5326.7(17) | 28.6(6) |
| C15 | 2424(4) | 3201(3) | 5580(2) | 47.6(8) |
| C16 | 964(4) | 4448(2) | 4601.5(19) | 35.6(7) |

**Table 2 Fractional Atomic Coordinates (×10$^4$) and Equivalent Isotropic Displacement Parameters (Å$^2$×10$^3$) for 5. $U_{eq}$ is defined as 1/3 of the trace of the orthogonalised $U_{IJ}$ tensor.**

| Atom | *x* | *y* | *z* | U(eq) |
|---|---|---|---|---|
| C17 | 6646(4) | 3468(2) | 6446.3(18) | 33.4(6) |

**Table 3 Anisotropic Displacement Parameters (Å$^2$×10$^3$) for 5. The Anisotropic displacement factor exponent takes the form: $-2\pi^2[h^2a^{*2}U_{11}+2hka^*b^*U_{12}+\ldots]$.**

| Atom | $U_{11}$ | $U_{22}$ | $U_{33}$ | $U_{23}$ | $U_{13}$ | $U_{12}$ |
|---|---|---|---|---|---|---|
| O1 | 22.3(9) | 22.8(8) | 34.1(10) | 2.9(7) | 0.1(8) | 0.4(7) |
| O2 | 23.4(10) | 32.0(9) | 37.4(11) | -3.9(8) | -0.5(8) | -2.2(8) |
| N1 | 18.9(11) | 27.2(11) | 37.5(13) | 5.7(10) | 1.6(9) | -0.6(9) |
| N2 | 17.5(10) | 21.5(10) | 36.4(12) | -2.3(9) | 0.6(9) | -0.5(8) |
| C1 | 22.6(13) | 20.9(11) | 31.4(14) | -1.0(11) | -0.7(11) | -0.4(10) |
| C2 | 17.3(12) | 20.5(11) | 33.0(15) | -0.2(10) | 1.2(10) | 2.2(10) |
| C3 | 23.6(13) | 25.5(12) | 33.5(15) | 1.3(11) | 1.2(11) | 1.6(11) |
| C4 | 19.8(12) | 29.4(13) | 30.8(14) | 0.7(11) | -2.0(10) | 1.4(11) |
| C5 | 22.6(13) | 38.2(15) | 49.7(18) | 14.2(14) | -3.1(12) | 1.5(12) |
| C6 | 22.1(13) | 24.0(12) | 30.6(14) | 1.8(11) | 2.4(11) | -0.8(10) |
| C7 | 25.4(13) | 24.9(12) | 28.9(13) | 0.3(10) | 1.4(11) | 1.3(11) |
| C8 | 29.9(14) | 26.7(13) | 36.0(15) | -2.3(11) | 4.1(12) | -1.6(11) |
| C9 | 42.5(16) | 24.8(13) | 37.4(16) | -0.1(12) | 5.2(13) | 3.0(12) |
| C10 | 35.1(16) | 31.0(14) | 37.6(16) | -0.8(12) | 2.3(12) | 11.7(13) |
| C11 | 24.4(14) | 34.1(14) | 37.1(16) | -2.5(12) | 2.2(12) | 3.8(12) |
| C12 | 26.3(13) | 25.5(12) | 33.5(15) | -0.9(11) | 1.4(11) | -0.1(11) |
| C13 | 20.3(12) | 24.2(12) | 32.6(14) | -0.5(11) | 0.1(10) | 0.8(10) |
| C14 | 22.5(13) | 24.5(13) | 38.7(15) | -2.4(11) | 0.7(11) | -2.6(11) |
| C15 | 45.2(19) | 31.7(15) | 66(2) | 11.9(15) | -17.3(17) | -12.1(14) |
| C16 | 30.5(14) | 24.3(13) | 52.0(18) | -0.5(13) | -7.2(13) | -3.0(11) |
| C17 | 29.5(14) | 29.8(14) | 41.0(16) | 1.5(12) | -1.2(13) | 4.2(12) |

**Table 4 Bond Lengths for 5.**

| Atom | Atom | Length/Å | Atom | Atom | Length/Å |
|---|---|---|---|---|---|
| O1 | C1 | 1.250(3) | C4 | C17 | 1.326(4) |
| O2 | C6 | 1.230(3) | C6 | C7 | 1.503(4) |
| N1 | C1 | 1.327(3) | C7 | C8 | 1.397(4) |
| N1 | C5 | 1.464(3) | C7 | C12 | 1.385(4) |
| N2 | C2 | 1.464(3) | C8 | C9 | 1.386(4) |
| N2 | C6 | 1.358(3) | C9 | C10 | 1.385(4) |
| C1 | C2 | 1.538(3) | C10 | C11 | 1.394(4) |
| C2 | C3 | 1.537(4) | C11 | C12 | 1.385(4) |
| C2 | C13 | 1.545(4) | C13 | C14 | 1.543(3) |

**Table 4 Bond Lengths for 5.**

| Atom | Atom | Length/Å | Atom | Atom | Length/Å |
|---|---|---|---|---|---|
| C3 | C4 | 1.499(4) | C14 | C15 | 1.533(4) |
| C4 | C5 | 1.488(4) | C14 | C16 | 1.522(4) |

**Table 5 Bond Angles for 5.**

| Atom | Atom | Atom | Angle/° | Atom | Atom | Atom | Angle/° |
|---|---|---|---|---|---|---|---|
| C1 | N1 | C5 | 126.7(2) | O2 | C6 | N2 | 123.0(2) |
| C6 | N2 | C2 | 120.6(2) | O2 | C6 | C7 | 121.8(2) |
| O1 | C1 | N1 | 121.7(2) | N2 | C6 | C7 | 115.2(2) |
| O1 | C1 | C2 | 119.1(2) | C8 | C7 | C6 | 118.5(2) |
| N1 | C1 | C2 | 118.9(2) | C12 | C7 | C6 | 122.3(2) |
| N2 | C2 | C1 | 108.43(19) | C12 | C7 | C8 | 119.1(3) |
| N2 | C2 | C3 | 109.9(2) | C9 | C8 | C7 | 119.8(3) |
| N2 | C2 | C13 | 108.7(2) | C10 | C9 | C8 | 120.6(3) |
| C1 | C2 | C13 | 104.2(2) | C9 | C10 | C11 | 119.9(3) |
| C3 | C2 | C1 | 111.9(2) | C12 | C11 | C10 | 119.2(3) |
| C3 | C2 | C13 | 113.5(2) | C7 | C12 | C11 | 121.3(2) |
| C4 | C3 | C2 | 110.7(2) | C14 | C13 | C2 | 117.9(2) |
| C5 | C4 | C3 | 113.9(2) | C15 | C14 | C13 | 113.2(2) |
| C17 | C4 | C3 | 124.7(3) | C16 | C14 | C13 | 109.3(2) |
| C17 | C4 | C5 | 121.3(3) | C16 | C14 | C15 | 108.7(2) |
| N1 | C5 | C4 | 113.5(2) | | | | |

**Table 6 Hydrogen Bonds for 5.**

| D | H | A | d(D-H)/Å | d(H-A)/Å | d(D-A)/Å | D-H-A/° |
|---|---|---|---|---|---|---|
| N1 | H1 | O1[1] | 0.88 | 2.04 | 2.878(3) | 157.7 |
| N2 | H2 | O1[2] | 0.88 | 2.17 | 2.864(3) | 135.9 |

[1]1/2+X,3/2-Y,1-Z; [2]-1/2+X,3/2-Y,1-Z

**Table 7 Torsion Angles for 5.**

| A | B | C | D | Angle/° | A | B | C | D | Angle/° |
|---|---|---|---|---|---|---|---|---|---|
| O1 | C1 | C2 | N2 | -40.5(3) | C2 | C13 | C14 | C15 | -94.5(3) |
| O1 | C1 | C2 | C3 | -161.8(2) | C2 | C13 | C14 | C16 | 144.3(2) |
| O1 | C1 | C2 | C13 | 75.2(3) | C3 | C2 | C13 | C14 | 63.8(3) |
| O2 | C6 | C7 | C8 | 30.9(4) | C3 | C4 | C5 | N1 | -37.9(3) |
| O2 | C6 | C7 | C12 | -146.2(3) | C5 | N1 | C1 | O1 | 179.3(3) |
| N1 | C1 | C2 | N2 | 145.7(2) | C5 | N1 | C1 | C2 | -7.1(4) |
| N1 | C1 | C2 | C3 | 24.4(3) | C6 | N2 | C2 | C1 | -51.4(3) |

**Table 7 Torsion Angles for 5.**

| A | B | C | D | Angle/° | A | B | C | D | Angle/° |
|---|---|---|---|---|---|---|---|---|---|
| N1 | C1 | C2 | C13 | -98.7(3) | C6 | N2 | C2 | C3 | 71.2(3) |
| N2 | C2 | C3 | C4 | -168.3(2) | C6 | N2 | C2 | C13 | -164.1(2) |
| N2 | C2 | C13 | C14 | -58.8(3) | C6 | C7 | C8 | C9 | -177.8(3) |
| N2 | C6 | C7 | C8 | -149.4(3) | C6 | C7 | C12 | C11 | 176.7(3) |
| N2 | C6 | C7 | C12 | 33.4(4) | C7 | C8 | C9 | C10 | 1.3(4) |
| C1 | N1 | C5 | C4 | 13.3(4) | C8 | C7 | C12 | C11 | -0.4(4) |
| C1 | C2 | C3 | C4 | -47.8(3) | C8 | C9 | C10 | C11 | -0.9(5) |
| C1 | C2 | C13 | C14 | -174.3(2) | C9 | C10 | C11 | C12 | -0.1(5) |
| C2 | N2 | C6 | O2 | -3.7(4) | C10 | C11 | C12 | C7 | 0.7(4) |
| C2 | N2 | C6 | C7 | 176.6(2) | C12 | C7 | C8 | C9 | -0.6(4) |
| C2 | C3 | C4 | C5 | 56.1(3) | C13 | C2 | C3 | C4 | 69.8(3) |
| C2 | C3 | C4 | C17 | -127.5(3) | C17 | C4 | C5 | N1 | 145.6(3) |

**Table 8 Hydrogen Atom Coordinates (Å×10$^4$) and Isotropic Displacement Parameters (Å$^2$×10$^3$) for 5.**

| Atom | *x* | *y* | *z* | U(eq) |
|---|---|---|---|---|
| H1 | 8019.59 | 6780.16 | 5278.07 | 33 |
| H2 | 1940.77 | 6774.81 | 5923.15 | 30 |
| H3A | 5098.7 | 5881.52 | 6963.04 | 33 |
| H3B | 4002.91 | 4752.18 | 6687.46 | 33 |
| H5A | 8407.72 | 5770.04 | 6493.34 | 44 |
| H5B | 8609.69 | 4909.28 | 5758.53 | 44 |
| H8 | 3353 | 10251.16 | 6693.46 | 37 |
| H9 | 1188.27 | 11578.13 | 6982.92 | 42 |
| H10 | -1505.14 | 10855.37 | 7163.8 | 42 |
| H11 | -2063.98 | 8784.02 | 7017.8 | 38 |
| H12 | 89.41 | 7470.03 | 6692.42 | 34 |
| H13A | 3353.73 | 5793.07 | 4721.47 | 31 |
| H13B | 4492.74 | 4702.2 | 5004.31 | 31 |
| H14 | 1374.07 | 4898.58 | 5744.74 | 34 |
| H15A | 3101.64 | 2805.71 | 5183.39 | 71 |
| H15B | 1386.33 | 2751.59 | 5649.32 | 71 |
| H15C | 3038.67 | 3218.84 | 6067.59 | 71 |
| H16A | 613.16 | 5267.13 | 4461.73 | 53 |
| H16B | -18.7 | 3947.1 | 4697.92 | 53 |
| H16C | 1611.6 | 4098.31 | 4178.08 | 53 |
| H17A | 7691.73 | 3105.36 | 6340.81 | 40 |
| H17B | 5751.51 | 2988.64 | 6631.16 | 40 |

**Crystal structure determination of 5**

**Crystal Data** for $C_{17}H_{22}N_2O_2$ ($M$ =286.36 g/mol): orthorhombic, space group $P2_12_12_1$ (no. 19), $a$ = 8.04670(10) Å, $b$ = 11.0689(2) Å, $c$ = 17.3352(4) Å, $V$ = 1544.01(5) Å$^3$, $Z$ = 4, $T$ = 100(2) K, μ(Cu Kα) = 0.647 mm$^{-1}$, $Dcalc$ = 1.232 g/cm$^3$, 22416 reflections measured (9.48° ≤ 2Θ ≤ 153.196°), 3130 unique ($R_{int}$ = 0.0514, $R_{sigma}$ = 0.0263) which were used in all calculations. The final $R_1$ was 0.0407 (I > 2σ(I)) and $wR_2$ was 0.1009 (all data).

## X-ray crystallographic analysis for compound 27 (ojh419ncs_2022ncs0294_1a)

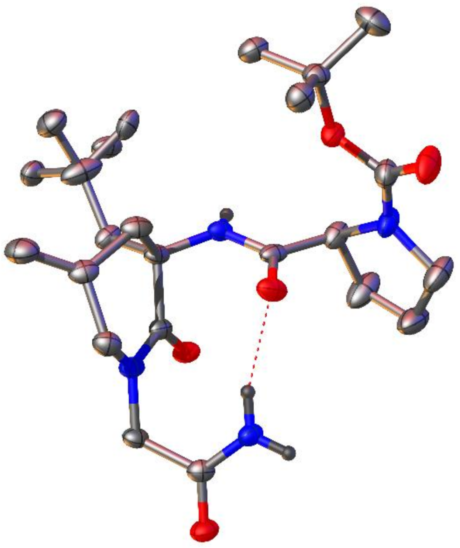

**Table 1 Crystal data and structure refinement for 27.**

| | |
|---|---|
| Identification code | ojh419ncs_2022ncs0294_1a |
| Empirical formula | $C_{22}H_{36}N_4O_5$ |
| Formula weight | 436.55 |
| Temperature/K | 100.00(10) |
| Crystal system | orthorhombic |
| Space group | $P2_12_12_1$ |
| a/Å | 10.3188(3) |
| b/Å | 14.5578(5) |
| c/Å | 16.1464(4) |
| α/° | 90 |
| β/° | 90 |
| γ/° | 90 |
| Volume/Å$^3$ | 2425.50(12) |
| Z | 4 |
| $\rho_{calc}$g/cm$^3$ | 1.195 |
| μ/mm$^{-1}$ | 0.695 |
| F(000) | 944.0 |
| Crystal size/mm$^3$ | 0.19 × 0.025 × 0.02 |
| Radiation | Cu Kα (λ = 1.54178) |
| 2Θ range for data collection/° | 8.178 to 136.476 |
| Index ranges | -12 ≤ h ≤ 12, -11 ≤ k ≤ 17, -18 ≤ l ≤ 19 |
| Reflections collected | 18854 |

| | |
|---|---|
| Independent reflections | 4443 [$R_{int}$ = 0.0292, $R_{sigma}$ = 0.0252] |
| Data/restraints/parameters | 4443/7/318 |
| Goodness-of-fit on $F^2$ | 1.022 |
| Final R indexes [I>=2σ (I)] | $R_1$ = 0.0442, $wR_2$ = 0.1086 |
| Final R indexes [all data] | $R_1$ = 0.0474, $wR_2$ = 0.1110 |
| Largest diff. peak/hole / e Å$^{-3}$ | 0.52/-0.51 |
| Flack parameter | 0.11(6) |

**Table 2 Fractional Atomic Coordinates (×10$^4$) and Equivalent Isotropic Displacement Parameters (Å$^2$×10$^3$) for 27. $U_{eq}$ is defined as 1/3 of the trace of the orthogonalised $U_{IJ}$ tensor.**

| Atom | *x* | *y* | *z* | U(eq) |
|---|---|---|---|---|
| O1 | 3269(2) | 6966.3(16) | 4973.2(12) | 28.1(5) |
| O2 | 131(2) | 6112.9(17) | 6294.7(13) | 32.8(5) |
| O3 | 1410(2) | 7154.4(17) | 3396.2(13) | 33.7(5) |
| O4 | 3(2) | 8500.9(19) | 1359.8(15) | 45.4(7) |
| O5 | 2114(2) | 8064.1(16) | 1552.7(12) | 28.4(5) |
| N1 | 2450(2) | 5573.5(19) | 4673.4(14) | 24.4(5) |
| N2 | 170(2) | 6578.3(19) | 4956.0(16) | 25.9(6) |
| N3 | 3593(2) | 7312.3(19) | 3310.3(15) | 25.1(5) |
| N4 | 1111(3) | 8822(2) | 2542.1(17) | 33.2(7) |
| C1 | 3132(3) | 6331(2) | 4472.5(17) | 23.8(6) |
| C2 | 3824(3) | 6386(2) | 3621.1(17) | 24.6(6) |
| C3 | 3325(3) | 5671(2) | 2986.1(18) | 29.6(7) |
| C4 | 3042(3) | 4774(2) | 3399.4(18) | 28.2(7) |
| C5 | 2061(3) | 4866(2) | 4075.7(19) | 29.0(7) |
| C6 | 1852(3) | 5552(2) | 5489.9(17) | 26.3(6) |
| C7 | 631(3) | 6120(2) | 5600.0(18) | 25.3(6) |
| C8 | 2368(3) | 7640(2) | 3268.2(17) | 27.7(7) |
| C9 | 2232(3) | 8656(2) | 3076.2(19) | 31.9(7) |
| C10 | 1893(4) | 9218(3) | 3852(3) | 54.9(12) |
| C11 | 482(4) | 9354(4) | 3827(2) | 50.8(11) |
| C12 | 23(4) | 9258(3) | 2967(2) | 49.1(10) |
| C13 | 980(3) | 8458(2) | 1780(2) | 32.3(7) |
| C14 | 2226(3) | 7595(2) | 753.8(19) | 28.9(7) |
| C15 | 1265(3) | 6801(2) | 715(2) | 32.8(7) |
| C16 | 3610(3) | 7239(2) | 781(2) | 33.1(7) |
| C17 | 2059(4) | 8272(3) | 45(2) | 43.7(9) |
| C18 | 5276(3) | 6235(2) | 3834.3(19) | 28.8(7) |
| C19 | 6266(4) | 6065(3) | 3164(2) | 52.4(11) |
| C20A | 7587(4) | 5851(3) | 3586(3) | 37.2(12) |
| C20B | 6608(17) | 5578(11) | 2529(9) | 41(4) |
| C21A | 6371(4) | 6795(3) | 2582(3) | 35.6(12) |
| C21B | 7324(13) | 6982(11) | 3216(9) | 38(4) |

**Table 2 Fractional Atomic Coordinates (×10$^4$) and Equivalent Isotropic Displacement Parameters (Å$^2$×10$^3$) for 27. $U_{eq}$ is defined as 1/3 of the trace of the orthogonalised $U_{IJ}$ tensor.**

| Atom | *x* | *y* | *z* | U(eq) |
|---|---|---|---|---|
| C22 | 3576(4) | 3985(3) | 3197(2) | 39.7(8) |

**Table 3 Anisotropic Displacement Parameters (Å$^2$×10$^3$) for 27. The Anisotropic displacement factor exponent takes the form: $-2\pi^2[h^2a^{*2}U_{11}+2hka^*b^*U_{12}+\ldots]$.**

| Atom | $U_{11}$ | $U_{22}$ | $U_{33}$ | $U_{23}$ | $U_{13}$ | $U_{12}$ |
|---|---|---|---|---|---|---|
| O1 | 27.6(11) | 36.4(12) | 20.3(10) | -4.3(10) | 1.2(8) | -2.4(9) |
| O2 | 31.2(12) | 44.4(13) | 22.7(11) | -0.2(10) | 5.0(9) | 4.1(10) |
| O3 | 20.4(11) | 55.2(15) | 25.5(11) | 8.5(10) | -2.6(9) | -2.3(10) |
| O4 | 38.5(14) | 57.4(16) | 40.3(14) | -13.4(12) | -19.6(12) | 22.1(12) |
| O5 | 27.9(11) | 35.1(11) | 22.2(10) | -2.8(9) | -4.6(8) | 7.0(9) |
| N1 | 21.1(12) | 35.1(14) | 16.9(11) | -0.4(10) | -0.5(10) | 0.7(11) |
| N2 | 20.5(13) | 35.4(14) | 21.9(13) | -1.3(11) | 0.9(11) | 2.9(11) |
| N3 | 18.6(12) | 36.9(15) | 19.9(12) | 1.8(11) | 0.1(10) | -1.5(11) |
| N4 | 27.4(14) | 39.6(16) | 32.5(14) | -8.4(12) | -6.2(12) | 14.4(12) |
| C1 | 17.6(13) | 35.2(17) | 18.5(13) | 1.7(13) | -2.4(11) | 2.7(12) |
| C2 | 21.8(14) | 35.3(17) | 16.9(13) | 0.4(12) | -1.0(11) | -1.6(12) |
| C3 | 26.8(15) | 44.6(19) | 17.4(14) | -3.5(14) | -2.4(12) | -6.7(14) |
| C4 | 27.1(15) | 38.4(18) | 19.3(14) | -7.1(13) | -2.8(12) | -4.3(13) |
| C5 | 27.5(16) | 34.4(17) | 25.1(15) | 0.9(13) | -0.8(12) | -4.4(14) |
| C6 | 25.6(15) | 35.3(17) | 17.9(14) | 3.4(13) | 0.2(12) | -1.3(13) |
| C7 | 22.1(14) | 32.0(16) | 21.9(14) | -2.0(13) | -0.6(12) | -5.5(13) |
| C8 | 20.6(14) | 47.7(19) | 14.9(13) | -0.4(13) | -0.7(11) | 1.5(14) |
| C9 | 26.1(16) | 41.1(19) | 28.7(16) | -11.0(14) | -6.6(13) | 6.5(14) |
| C10 | 41(2) | 76(3) | 48(2) | -33(2) | -10.4(19) | 16(2) |
| C11 | 41(2) | 80(3) | 31.7(19) | -6(2) | 2.9(16) | 18(2) |
| C12 | 34.7(19) | 63(3) | 50(2) | -25(2) | -5.2(18) | 18.7(19) |
| C13 | 33.7(17) | 32.4(17) | 30.8(17) | -2.9(14) | -4.8(14) | 10.2(14) |
| C14 | 31.0(16) | 34.8(17) | 20.8(15) | -1.9(13) | -0.4(12) | 0.4(14) |
| C15 | 29.0(16) | 36.3(18) | 33.2(17) | -3.2(14) | -1.8(13) | 0.3(14) |
| C16 | 27.8(16) | 41.7(19) | 29.7(17) | -7.2(14) | 3.4(13) | -0.1(15) |
| C17 | 57(2) | 45(2) | 28.5(17) | 8.1(16) | -5.5(17) | -7.2(18) |
| C18 | 22.0(15) | 39.5(18) | 24.9(15) | -9.0(14) | -3.3(12) | 3.5(13) |
| C19 | 31.1(19) | 90(3) | 36(2) | -24(2) | 3.5(16) | -4(2) |
| C20A | 28(2) | 48(3) | 36(2) | 3(2) | 1.8(19) | 11.7(19) |
| C20B | 43(9) | 37(9) | 43(9) | -11(7) | 9(7) | 0(7) |
| C21A | 24(2) | 37(3) | 46(3) | -8(2) | -1.7(19) | 9.4(19) |
| C21B | 22(7) | 63(11) | 31(7) | 3(7) | 4(6) | 1(7) |
| C22 | 49(2) | 43(2) | 27.0(17) | -5.3(15) | 6.2(15) | -4.5(17) |

**Table 4 Bond Lengths for 27.**

| Atom | Atom | Length/Å | Atom | Atom | Length/Å |
|---|---|---|---|---|---|
| O1 | C1 | 1.237(4) | C2 | C18 | 1.553(4) |
| O2 | C7 | 1.235(4) | C3 | C4 | 1.495(5) |
| O3 | C8 | 1.232(4) | C4 | C5 | 1.495(4) |
| O4 | C13 | 1.217(4) | C4 | C22 | 1.316(5) |
| O5 | C13 | 1.353(4) | C6 | C7 | 1.517(4) |
| O5 | C14 | 1.464(4) | C8 | C9 | 1.518(5) |
| N1 | C1 | 1.348(4) | C9 | C10 | 1.536(5) |
| N1 | C5 | 1.468(4) | C10 | C11 | 1.470(6) |
| N1 | C6 | 1.456(4) | C11 | C12 | 1.473(5) |
| N2 | C7 | 1.324(4) | C14 | C15 | 1.525(5) |
| N3 | C2 | 1.458(4) | C14 | C16 | 1.519(5) |
| N3 | C8 | 1.353(4) | C14 | C17 | 1.519(5) |
| N4 | C9 | 1.463(4) | C18 | C19 | 1.509(5) |
| N4 | C12 | 1.462(4) | C19 | C20A | 1.555(6) |
| N4 | C13 | 1.347(4) | C19 | C20B | 1.296(13) |
| C1 | C2 | 1.551(4) | C19 | C21A | 1.422(6) |
| C2 | C3 | 1.549(4) | C19 | C21B | 1.727(14) |

**Table 5 Bond Angles for 27.**

| Atom | Atom | Atom | Angle/° | Atom | Atom | Atom | Angle/° |
|---|---|---|---|---|---|---|---|
| C13 | O5 | C14 | 120.3(2) | O3 | C8 | N3 | 122.6(3) |
| C1 | N1 | C5 | 124.0(2) | O3 | C8 | C9 | 121.3(3) |
| C1 | N1 | C6 | 117.2(3) | N3 | C8 | C9 | 116.1(3) |
| C6 | N1 | C5 | 117.7(2) | N4 | C9 | C8 | 110.7(3) |
| C8 | N3 | C2 | 119.7(3) | N4 | C9 | C10 | 102.3(3) |
| C12 | N4 | C9 | 113.7(3) | C8 | C9 | C10 | 111.9(3) |
| C13 | N4 | C9 | 123.6(3) | C11 | C10 | C9 | 106.0(3) |
| C13 | N4 | C12 | 121.5(3) | C10 | C11 | C12 | 109.4(3) |
| O1 | C1 | N1 | 120.9(3) | N4 | C12 | C11 | 103.7(3) |
| O1 | C1 | C2 | 119.2(3) | O4 | C13 | O5 | 125.9(3) |
| N1 | C1 | C2 | 119.8(3) | O4 | C13 | N4 | 124.9(3) |
| N3 | C2 | C1 | 106.1(2) | N4 | C13 | O5 | 109.1(3) |
| N3 | C2 | C3 | 109.8(2) | O5 | C14 | C15 | 109.8(3) |
| N3 | C2 | C18 | 111.4(3) | O5 | C14 | C16 | 102.0(2) |
| C1 | C2 | C18 | 103.9(2) | O5 | C14 | C17 | 110.6(3) |
| C3 | C2 | C1 | 113.5(3) | C16 | C14 | C15 | 110.7(3) |
| C3 | C2 | C18 | 111.9(3) | C17 | C14 | C15 | 112.8(3) |
| C4 | C3 | C2 | 110.9(2) | C17 | C14 | C16 | 110.4(3) |
| C5 | C4 | C3 | 112.3(3) | C19 | C18 | C2 | 121.1(3) |
| C22 | C4 | C3 | 124.8(3) | C18 | C19 | C20A | 108.2(3) |
| C22 | C4 | C5 | 122.9(3) | C18 | C19 | C21B | 105.4(6) |

**Table 5 Bond Angles for 27.**

| Atom | Atom | Atom | Angle/° | Atom | Atom | Atom | Angle/° |
|---|---|---|---|---|---|---|---|
| N1 | C5 | C4 | 111.0(3) | C20B | C19 | C18 | 147.3(8) |
| N1 | C6 | C7 | 116.5(2) | C20B | C19 | C21B | 106.8(9) |
| O2 | C7 | N2 | 124.6(3) | C21A | C19 | C18 | 113.7(4) |
| O2 | C7 | C6 | 116.7(3) | C21A | C19 | C20A | 111.8(4) |
| N2 | C7 | C6 | 118.7(3) | | | | |

**Table 6 Hydrogen Bonds for 27.**

| D | H | A | d(D-H)/Å | d(H-A)/Å | d(D-A)/Å | D-H-A/° |
|---|---|---|---|---|---|---|
| N2 | H2A | O1[1] | 0.85(3) | 2.07(3) | 2.890(4) | 162(4) |
| N3 | H3 | O2[2] | 0.87(4) | 2.04(4) | 2.860(4) | 157(4) |

[1]-1/2+X,3/2-Y,1-Z; [2]1/2+X,3/2-Y,1-Z

**Table 7 Torsion Angles for 27.**

| A | B | C | D | Angle/° | A | B | C | D | Angle/° |
|---|---|---|---|---|---|---|---|---|---|
| O1 | C1 | C2 | N3 | -45.9(3) | C5 | N1 | C1 | O1 | 169.3(3) |
| O1 | C1 | C2 | C3 | -166.6(3) | C5 | N1 | C1 | C2 | -14.3(4) |
| O1 | C1 | C2 | C18 | 71.7(3) | C5 | N1 | C6 | C7 | -91.8(3) |
| O3 | C8 | C9 | N4 | -38.6(4) | C6 | N1 | C1 | O1 | 1.9(4) |
| O3 | C8 | C9 | C10 | 74.8(4) | C6 | N1 | C1 | C2 | 178.4(3) |
| N1 | C1 | C2 | N3 | 137.6(3) | C6 | N1 | C5 | C4 | -160.7(3) |
| N1 | C1 | C2 | C3 | 16.9(4) | C8 | N3 | C2 | C1 | -52.8(3) |
| N1 | C1 | C2 | C18 | -104.8(3) | C8 | N3 | C2 | C3 | 70.3(3) |
| N1 | C6 | C7 | O2 | -179.1(3) | C8 | N3 | C2 | C18 | -165.2(3) |
| N1 | C6 | C7 | N2 | 1.4(4) | C8 | C9 | C10 | C11 | -98.1(4) |
| N3 | C2 | C3 | C4 | -156.9(3) | C9 | N4 | C12 | C11 | -1.2(5) |
| N3 | C2 | C18 | C19 | -77.5(4) | C9 | N4 | C13 | O4 | 171.6(4) |
| N3 | C8 | C9 | N4 | 143.0(3) | C9 | N4 | C13 | O5 | -10.2(5) |
| N3 | C8 | C9 | C10 | -103.6(3) | C9 | C10 | C11 | C12 | -22.8(6) |
| N4 | C9 | C10 | C11 | 20.5(5) | C10 | C11 | C12 | N4 | 15.1(5) |
| C1 | N1 | C5 | C4 | 32.0(4) | C12 | N4 | C9 | C8 | 107.4(3) |
| C1 | N1 | C6 | C7 | 76.4(3) | C12 | N4 | C9 | C10 | -12.0(4) |
| C1 | C2 | C3 | C4 | -38.3(4) | C12 | N4 | C13 | O4 | 4.6(6) |
| C1 | C2 | C18 | C19 | 168.7(3) | C12 | N4 | C13 | O5 | -177.2(3) |
| C2 | N3 | C8 | O3 | -8.2(4) | C13 | O5 | C14 | C15 | -60.4(4) |
| C2 | N3 | C8 | C9 | 170.1(2) | C13 | O5 | C14 | C16 | -177.9(3) |
| C2 | C3 | C4 | C5 | 58.3(3) | C13 | O5 | C14 | C17 | 64.6(4) |
| C2 | C3 | C4 | C22 | -122.2(4) | C13 | N4 | C9 | C8 | -60.5(4) |
| C2 | C18 | C19 | C20A | -174.7(3) | C13 | N4 | C9 | C10 | -179.9(4) |
| C2 | C18 | C19 | C20B | -54.1(18) | C13 | N4 | C12 | C11 | 167.0(4) |

**Table 7 Torsion Angles for 27.**

| A | B | C | D | Angle/° | A | B | C | D | Angle/° |
|---|---|---|---|---|---|---|---|---|---|
| C2 | C18 | C19 | C21A | 60.4(5) | C14 | O5 | C13 | O4 | -3.3(5) |
| C2 | C18 | C19 | C21B | 116.9(6) | C14 | O5 | C13 | N4 | 178.5(3) |
| C3 | C2 | C18 | C19 | 45.9(4) | C18 | C2 | C3 | C4 | 78.9(3) |
| C3 | C4 | C5 | N1 | -53.9(3) | C22 | C4 | C5 | N1 | 126.7(3) |

**Table 8 Hydrogen Atom Coordinates (Å×10[4]) and Isotropic Displacement Parameters (Å[2]×10[3]) for 27.**

| Atom | *x* | *y* | *z* | U(eq) |
|---|---|---|---|---|
| H2A | -500(30) | 6910(30) | 5020(20) | 34(10) |
| H2B | 550(40) | 6590(30) | 4480(20) | 33(10) |
| H3 | 4200(40) | 7730(30) | 3320(30) | 45(12) |
| H3A | 2543.59 | 5901 | 2724.3 | 36 |
| H3B | 3973.06 | 5580.56 | 2558.6 | 36 |
| H5A | 1227.93 | 5026.53 | 3838.27 | 35 |
| H5B | 1967.81 | 4281.66 | 4358.6 | 35 |
| H6A | 2486.91 | 5761.01 | 5890.97 | 32 |
| H6B | 1647.78 | 4918.55 | 5622.09 | 32 |
| H9 | 3024.74 | 8891.3 | 2817.67 | 38 |
| H10A | 2140.81 | 8888.04 | 4349.02 | 66 |
| H10B | 2338.52 | 9804.47 | 3844.47 | 66 |
| H11A | 267.88 | 9960.25 | 4034.59 | 61 |
| H11B | 58.81 | 8902.22 | 4175.88 | 61 |
| H12A | -746.61 | 8876.34 | 2941.03 | 59 |
| H12B | -171.2 | 9853.35 | 2727.06 | 59 |
| H15A | 1383.3 | 6410.91 | 1188.47 | 49 |
| H15B | 1409.8 | 6453.02 | 218.45 | 49 |
| H15C | 397.4 | 7038.61 | 713.72 | 49 |
| H16A | 4195.18 | 7744.35 | 856.23 | 50 |
| H16B | 3806.32 | 6931.54 | 269.83 | 50 |
| H16C | 3701.11 | 6815.59 | 1232.75 | 50 |
| H17A | 1203.57 | 8529.38 | 64.75 | 66 |
| H17B | 2180.3 | 7958.68 | -472.16 | 66 |
| H17C | 2688.6 | 8753.93 | 96.2 | 66 |
| H18A | 5561.55 | 6770.64 | 4141.58 | 35 |
| H18B | 5318.39 | 5717.92 | 4212.14 | 35 |
| H19 | 5998.19 | 5513.81 | 2860.1 | 63 |
| H19A | 6805.81 | 5697.3 | 3537.27 | 63 |
| H20A | 7491.14 | 5331.31 | 3945.97 | 56 |
| H20B | 7861.32 | 6374.58 | 3901.94 | 56 |
| H20C | 8222.54 | 5716.36 | 3169.01 | 56 |
| H20D | 7360.93 | 5221.64 | 2666.23 | 61 |

**Table 8 Hydrogen Atom Coordinates (Å×10[4]) and Isotropic Displacement Parameters (Å[2]×10[3]) for 27.**

| Atom | *x* | *y* | *z* | U(eq) |
|---|---|---|---|---|
| H20E | 6804.16 | 5976.87 | 2072.6 | 61 |
| H20F | 5912.01 | 5173.51 | 2377.49 | 61 |
| H21A | 7014.96 | 6643.7 | 2175.23 | 53 |
| H21B | 6617.76 | 7348.09 | 2864.36 | 53 |
| H21C | 5550.38 | 6885.22 | 2313.73 | 53 |
| H21D | 7930.07 | 6884.63 | 3658.09 | 58 |
| H21E | 6845.42 | 7536.77 | 3316.83 | 58 |
| H21F | 7784.12 | 7035.87 | 2701.34 | 58 |
| H22A | 3346.12 | 3451.98 | 3478.54 | 48 |
| H22B | 4182.43 | 3959.48 | 2771.27 | 48 |

**Table 9 Atomic Occupancy for 27.**

| Atom | *Occupancy* | Atom | *Occupancy* | Atom | *Occupancy* |
|---|---|---|---|---|---|
| H19 | 0.772(7) | H19A | 0.228(7) | C20A | 0.772(7) |
| H20A | 0.772(7) | H20B | 0.772(7) | H20C | 0.772(7) |
| C20B | 0.228(7) | H20D | 0.228(7) | H20E | 0.228(7) |
| H20F | 0.228(7) | C21A | 0.772(7) | H21A | 0.772(7) |
| H21B | 0.772(7) | H21C | 0.772(7) | C21B | 0.228(7) |
| H21D | 0.228(7) | H21E | 0.228(7) | H21F | 0.228(7) |

**Crystal structure determination of 27**

**Crystal Data** for $C_{22}H_{36}N_4O_5$ ($M$ =436.55 g/mol): orthorhombic, space group $P2_12_12_1$ (no. 19), $a$ = 10.3188(3) Å, $b$ = 14.5578(5) Å, $c$ = 16.1464(4) Å, $V$ = 2425.50(12) Å$^3$, $Z$ = 4, $T$ = 100.00(10) K, μ(Cu Kα) = 0.695 mm$^{-1}$, $Dcalc$ = 1.195 g/cm$^3$, 18854 reflections measured (8.178° ≤ 2Θ ≤ 136.476°), 4443 unique ($R_{int}$ = 0.0292, $R_{sigma}$ = 0.0252) which were used in all calculations. The final $R_1$ was 0.0442 (I > 2σ(I)) and $wR_2$ was 0.1110 (all data).

**References**


1. (a) B. D. W. Allen, M. J. Connolly and J. P. A. Harrity, *Chem. Eur. J.*, 2016, **22**, 13000–13003; (b) García-Vázquez, V.; Hoteite, L.; Lakeland, C. P.; Watson, D. W.; Harrity, J. P. A. *Org. Lett.,* **2021**, *23*, 2811-2815.
2. Melhado, A. D.; Luparia, M.; Toste, F. D. *J. Am. Chem. Soc.* **2007,** *129*, 12638-12639.
3. Zhang, H.; Yang, Z.; Zhao, B. N.; Li, G. *J. Org. Chem.* **2018,** *83*, 644-655.
4. Chen, F. M.; Benoiton, N. L. *Int. J. Peptide and Protein Res.* **1987**, *30*, 683-688.
5. Latorre, A.; Sáez, J. A.; Rodriguez, S.; Gonzalez, F. V. *Tetrahedron* **2014**, *70*, 97-102.
6. Kimura, M.; Tamaki, T.; Nakata, M.; Tohyama, K.; Tamaru, Y. *Angew. Chem. Int. Ed.* **2008**, *47*, 5803-5805.
7. Coles, S. J.; Allan, D. R.; Beavers, C. M.; Teat, S. J.; Holgate, S. J.W. (2020) Leading edge chemical crystallography service provision and its impact on crystallographic data science in the twenty-first century. In, Structure and Bonding. Berlin, Heidelberg. Springer, pp. 1-72. (doi:10.1007/430_2020_63).
8. (a) Krause, L.; Herbst-Irmer, R.; Sheldrick, G. M.; Stalke, D. *J. Appl. Cryst.* **2015**, *48*, 3–10; (b) Blessing, R. H. *Acta Crystallogr.* **1995**, *A51*, 33–38.
9. Sheldrick, G. M. *Acta Crystallogr.* **2015**, *A71*, 3–8.
10. Sheldrick, G. M. *Acta Crystallogr.* **2015**, *C71*, 3–8.
11. Dolomanov, O. V.; Bourhis, L. J.; Gildea, R. J.; Howard, J. A. K.; Puschmann, H. *J. Appl. Cryst.* **2009**, *42*, 339–341.
